# Structure-Activity Relationships of Benzothiazole-Based Hsp90 C-Terminal-Domain Inhibitors

**DOI:** 10.3390/pharmaceutics13081283

**Published:** 2021-08-17

**Authors:** Jaka Dernovšek, Živa Zajec, Martina Durcik, Lucija Peterlin Mašič, Martina Gobec, Nace Zidar, Tihomir Tomašič

**Affiliations:** Faculty of Pharmacy, University of Ljubljana, Aškerčeva Cesta 7, 1000 Ljubljana, Slovenia; jaka.dernovsek@ffa.uni-lj.si (J.D.); ziva.zajec@ffa.uni-lj.si (Ž.Z.); martina.durcik@ffa.uni-lj.si (M.D.); lucija.peterlinmasic@ffa.uni-lj.si (L.P.M.); martina.gobec@ffa.uni-lj.si (M.G.); nace.zidar@ffa.uni-lj.si (N.Z.)

**Keywords:** allosteric, Hsp90, benzothiazole, cancer, inhibitor, cancer therapy

## Abstract

Heat shock protein 90 (Hsp90) is a chaperone responsible for the maturation of many cancer-related proteins, and is therefore an important target for the design of new anticancer agents. Several Hsp90 N-terminal domain inhibitors have been evaluated in clinical trials, but none have been approved as cancer therapies. This is partly due to induction of the heat shock response, which can be avoided using Hsp90 C-terminal-domain (CTD) inhibition. Several structural features have been shown to be useful in the design of Hsp90 CTD inhibitors, including an aromatic ring, a cationic center and the benzothiazole moiety. This study established a previously unknown link between these structural motifs. Using ligand-based design methodologies and structure-based pharmacophore models, a library of 29 benzothiazole-based Hsp90 CTD inhibitors was prepared, and their antiproliferative activities were evaluated in MCF-7 breast cancer cells. Several showed low-micromolar IC_50_, with the most potent being compounds **5g** and **9i** (IC_50_, 2.8 ± 0.1, 3.9 ± 0.1 μM, respectively). Based on these results, a ligand-based structure–activity relationship model was built, and molecular dynamics simulation was performed to elaborate the binding mode of compound **9i**. Moreover, compound **9i** showed degradation of Hsp90 client proteins and no induction of the heat shock response.

## 1. Introduction

Heat shock protein 90 (Hsp90) is a chaperone that consists of four highly conserved isoforms: inducible Hsp90α and constitutively expressed Hsp90β (both localized in the cytosol), mitochondrial TRAP-1, and Grp94 (localized in the endoplasmic reticulum) [1,2,3]. To carry out their functions, all four Hsp90 isoforms are obligate homodimers [3]. Each monomeric unit of Hsp90 consists of an ATP-hydrolyzing N-terminal domain (NTD), which is connected via a charged linker to the middle domain and C-terminal domain (CTD) [2,4,5]. This last is responsible for dimerization [5,6], and it forms a secondary nucleotide binding pocket without ATPase activity. This secondary binding site only becomes available upon the binding of ATP to the NTD [7,8].

Together with its co-chaperones, Hsp90 is responsible for ensuring proteostasis, by guaranteeing correct protein folding and maturation, along with preventing aggregation of its client proteins [5,9,10]. In a healthy human cell, Hsp90 represents 1% to 2% of all the protein, making it one of the most abundant cellular proteins [11]. Although Hsp90 is vital for the functionality of more than 400 proteins [12,13], its abundance still provides a functional reserve and allows cells to function even when Hsp90 is downregulated [14].

Furthermore, the expression of Hsp90 is increased in various cancers due to the stress conditions and oxygen deprivation in the tumor environment [13,15]. Therefore, although mutations in Hsp90 itself are scarce [13], this chaperone is involved in all 10 hallmarks of cancer [6,12]. The protein clients of Hsp90 include protein kinases (e.g., Akt, Cdk4), transcription factors (e.g., p53, Hif1), E3 ubiquitin ligases, and steroid hormone receptors, all of which are essential for cancer pathogenesis [2,16]. Under malignant transformation, the oncogenic Hsp90 client proteins become even more dependent on Hsp90 to maintain a suitable conformation for their function [17]. Consequently, Hsp90 has been intensively studied for the development of anticancer agents since the early 1990s [13,18,19,20].

Since the discovery of the first Hsp90 NTD inhibitor geldanamycin in 1994 [19], at least 18 Hsp90 inhibitors that target the NTD have entered clinical trials. Unfortunately, none of these have been successful to date [13,21], due to various toxicities and strong activation of the heat shock response (HSR) [13]. The HSR is particularly problematic because the upregulation of different heat shock proteins that are controlled by heat shock factor 1 (HSF1) leads to cytoprotective effects, thus counteracting any treatment effects [13,21,22,23]. Therefore, investigations have shifted toward the development of NTD isoform-selective inhibitors [12], protein–protein interaction inhibitors [24], and CTD allosteric modulators of Hsp90 [12,25], which do not induce the HSR.

**Figure 1:**
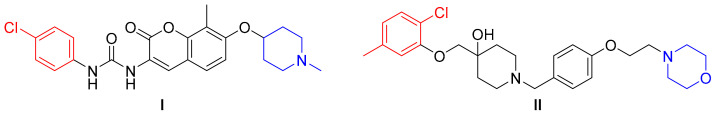
Two known Hsp90 C-terminal-domain inhibitors, (**I**) and (**II**), that feature a cationic center and aromatic ring at the opposite ends of their structures [30,32].

The first Hsp90 CTD inhibitor discovered was the coumarin antibiotic novobiocin [26], which was originally developed as a bacterial DNA gyrase inhibitor [27]. All of the subsequent ligands have contributed to the definition of the structure–activity relationships (SARs) necessary for Hsp90 CTD inhibition. Many of the resulting compounds (e.g., see Figure 1) have highlighted the importance of the cationic center at a sufficient distance from the aromatic ring, while the linker is important for molecular rigidity and provides an opportunity for hydrogen bond formation with the Hsp90 CTD [28,29,30,31,32]. From a series of novobiocin core analogs, a distance from 7.7 Å to 12.1 Å between the *N*-methylpiperidine and the biaryl side chain was shown to be optimal for Hsp90 CTD inhibition [33].

Although the Hsp90 CTD is characterized by X-ray crystallography and cryo-electron microscopy, the allosteric binding site has not been specified. Therefore, the exact binding mode of Hsp90 CTD inhibitors remains challenging to determine. Nevertheless, a structure-based study using the cryo-electron microscopy structure of Hsp90β confirmed the importance of the basic center and the aromatic ring at the appropriate distance [32]. On the other hand, the benzothiazole has been shown to be a useful structural feature for Hsp90 inhibitors [34] that target the CTD [35]. However, no connections between the CTD SARs and the benzothiazole ring as a central scaffold have been established to date.

Therefore, we conducted this SAR study using computational and biological methods of evaluation to investigate benzothiazole-based Hsp90 inhibitors that also feature characteristic structural properties of other CTD inhibitors.

## 2. Materials and Methods

### 2.1. Synthetic Procedures and Analytical Data

Reagents and solvents for synthesis were purchased from Enamine Ltd. (Kyiv, Ukraine), Apollo Scientific Ltd. (Stockport, UK), Sigma-Aldrich (St. Louis, MO, USA), TCI (Tokyo, Japan), and Fluorochem Ltd. (Derbyshire, UK), and were not further purified. Analytical thin-layer chromatography was performed on silica gel aluminum sheets (0.20 mm; 60 F254; Merck, Darmstadt, Germany). Column chromatography was performed using silica gel 60 (particle size, 230–400 mesh). ^1^H, ^13^C and ^19^F NMR spectra were recorded on a 400 MHz NMR spectrometer (Bruker Advance 3, Bruker, Billerica, MA, USA). The splitting patterns were designated as follows: s, singlet; d, doublet; dd, double doublet; td, triple doublet; t, triplet; dt, double triplet; ddd, double of doublet of doublet; q, quartet; p, pentet; and m, multiplet. The purities of the prepared compounds were monitored by liquid chromatography–mass spectrometry that was performed using method A (see below) on a 1260 Infinity II LC system (Agilent Technologies, Santa Clara, CA, USA), which was equipped with a quaternary pump and a wavelength detector. The system was coupled to the mass spectrometry (Expression CMS^L^; Advion Inc., Ithaca, NY, USA). The high-resolution mass spectra (HRMS) (Exactive Plus Orbitrap mass spectrometer; Thermo Scientific Inc., Waltham, MA, USA) used optical rotation detection at λ = 589 nm (Polarimeter Model 241; Perkin Elmer, Boston, MA, USA).

Method A: A C18 column was used (Waters xBridge BEH; 4.6 mm × 150 mm, 3.5 µm) at 40 °C. The flow rate was of the mobile phase was 1.5 mL/min, the injection volume was 10 µL, and the products were detected at 254 nm. Solvent A comprised 1% CH_3_CN and 0.1% HCOOH in double-distilled H_2_O; Solvent B comprised CH_3_CN. The following elution gradient was used: 0→1 min, 25% B; 1→6 min, 25%→98% B; 6→6.5 min, 98% B; 6.5→7 min, 98%→25% B; 7→10 min, 25% B.

Detailed chemical synthesis procedures and chemical analysis results of all intermediates and final compounds **5a–m**, **8k–n**, **9a–j**, **10** and **14** are described in Appendix A and Supplementary Materials. 

### 2.2. Docking

The software FRED (OEDOCKING 3.3.0.2: OpenEye Scientific Software, Santa Fe, NM, USA, http://www.eyesopen.com, accessed on 1 July 2021) [36,37] was used for docking experiments at the Hsp90β CTD binding site [32] (PDB entry: 5FWK) [38]. The binding site for docking experiments was created using MAKE RECEPTOR (Release 3.2.0.2, OpenEye Scientific Software, Inc., Santa Fe, NM, USA; www.eyesopen.com, accessed on 1 July 2021). The grid box with the following dimensions: 21.67 Å × 24.67 Å × 16.00 Å and the volume of 8551 Å^3^ was automatically generated around the coumarin-based Hsp90 CTD inhibitor and was not adjusted. The “Molecular” method was used for “Cavity detection”, and the outer and inner contours were automatically calculated with the “Balanced” settings. The inner contours were disabled. A library of a maximum of 200 conformations per ligand was created using OMEGA (Release 3.3.1.2, OpenEye Scientific Software, Inc., Santa Fe, NM, USA; www.eyesopen.com, accessed on 1 July 2021). The library of ligand conformers was then rigidly docked to the prepared Hsp90 CTD binding site using FRED (default settings). Docking poses were scored and ranked using Chemgauss4 scoring function. The results were visualized and analyzed with VIDA (version 4.3.0.4, OpenEye Scientific Software, Inc., Santa Fe, NM, USA, www.eyesopen.com, accessed on 1 July 2021).

### 2.3. Molecular Dynamics Simulations

MD simulations of the Hsp90-**9i** complex were performed using NAMD package (version 2.9) [39] and the CHARMM22 force field [40,41]. The ParamChem tool [42,43,44] was used to estimate the molecular mechanics parameters for compound **9i**. Removal of potential steric clashes and optimization of the atomic coordinates of the Hsp90β-**9i** docking complex were first performed by steepest descent and adopted basis Newton−Raphson energy minimizations (10,000 steps each). The structure of the Hsp90-**9i** complex embedded in a box of TIP3P water molecules and neutralized by the addition of NaCl was prepared using psfgen in VMD (version 1.9.1) [45]. The MD simulation was carried out in the NPT ensemble using the periodic boundary conditions. The Langevin dynamics and Langevin piston methods were used for temperature (300 K) and pressure (1 atm) control, respectively. Short-range and long-range forces were calculated every 1 and 2 time steps, respectively, with a time step of 2.0 ps. The smooth particle mesh Ewald method [46] was used to calculate the electrostatic interactions. The short-range interactions were cut off at 12 Å. All of the chemical bonds between hydrogen and the heavy atoms were held fixed, using the SHAKE algorithm [47]. The simulation consisted of three consecutive steps: (i) solvent equilibration for 1 ns with ligand and protein constrained harmonically around the initial structure; (ii) equilibration of the complete system for 1 ns with ligand and protein released; and (iii) an unconstrained 500 ns production run. For structure-based pharmacophore modeling, 2500 frames from the production run were saved separately and used for interaction analysis.

### 2.4. Structure-Based Pharmacophore Modeling

The 500 ns MD trajectory of Hsp90β dimer (PDB Entry: 5FWK) [38] in complex with compound **9i** was used for chemical feature interaction analysis, using LigandScout 4.4 Expert [48], which resulted in 2500 structure-based pharmacophore models.

### 2.5. MTS Assay

The compounds were evaluated for their antiproliferative activities against the MCF-7 (ATCC HTB-22, adherent cells isolated form 69 years old white female) breast cancer cell line, using an MTS (Promega, Madison, WI, USA) assay, according to the manufacturer’s instructions. The MCF-7 cell line was obtained from American Type Culture Collection (ATCC, Manassas, VA, USA). The cells were cultured in Dubecco’s modified Eagle’s medium (Sigma-Aldrich, St. Louis, MO, USA), which was supplemented with 10% fetal bovine serum (Gibco, Thermo Fisher Scientific, Waltham, MA, USA), 100 U/mL penicillin (Sigma-Aldrich, St. Louis, MO, USA), 100 µg/mL streptomycin (Sigma-Aldrich, St. Louis, MO, USA), and 2 mM l-glutamine (Sigma-Aldrich, St. Louis, MO, USA). MCF-7 cells were incubated in a 5% CO_2_ atmosphere at 37 °C. The cells were plated in 96-well plates at a density of 2000 cells per well. Afterwards, the cells were incubated for 24 h, and then treated with the final compounds, positive control (1 µM 17-DMAG) or vehicle control (0.5% DMSO). After the 72 h incubation, CellTiter96 Aqueous One Solution Reagent (10 µL; Promega, Madison, WI, USA) was added to each well, and the cells were incubated for an additional 3 h. Then the absorbance was measured using a microplate reader (Synergy 4 Hybrid; BioTek, Winooski, VT, USA). Independent experiments were repeated two times, each performed in triplicate. The statistically significant differences (*p* < 0.05) were calculated between the treated groups and DMSO, using two-tailed Welch’s *t*-tests. The IC_50_ values were determined using GraphPad Prism 8.0 software (San Diego, CA, USA), and represent the concentration at which a compound produced a half-maximal response; these are given as means from the independent measurements.

### 2.6. Western Blotting of MCF-7 Cells

MCF-7 cells were cultured as previously described. The cells were treated with 10 µM and 1 µM compound **9i**, 0.5 µM 17-DMAG (positive control) and 0.5% DMSO (vehicle) and incubated for 24 h. After these incubations, the cells were rinsed with 1 × DPBS (Gibco, Thermo Fisher Scientific, Waltham, MA, USA) and then lysed with RIPA buffer (50 mM Tris-HCl, pH 7.4, 150 mM NaCl, 1% NP-40, 0.5% sodium deoxycholate, 1 mM EDTA), containing 1:100 Halt protease inhibitor cocktail (Thermo Fisher Scientific, Waltham, MA, USA) and 1:100 Halt phosphatase inhibitor cocktail (Thermo Fisher Scientific, Waltham, MA, USA). Cell lysates were sonicated and then centrifuged at 15,000 rpm for 20 min at 4 °C. The supernatants were collected, and the protein concentrations were determined using the DC protein assay (Bio-Rad, Hercules, CA, USA). Equal amounts of protein (30 µg) were separated, using SDS PAGE (10% acrylamide/bisacrylamide gels,), electrophoresed at 80 V for 15 min, then at 130 V for 60 min, and then transferred onto nitrocellulose membranes using a dry blotting system (iBlot 2; Thermo Fisher Scientific, Waltham, MA, USA). Nonspecific binding sites were blocked in 5% bovine serum albumin for 1 h at room temperature, prior to exposure to the primary antibody solutions. Membranes were incubated with the primary antibodies overnight at 4 °C, and then with the secondary antibodies for 1 h at room temperature. The primary antibodies used in these experiments included anti-Hsp90 rabbit mAb (1:1000, Cell Signaling, Danvers, MA, USA), Hsp70 Mouse mAb (1:1000, Cell Signaling, Danvers, MA, USA), anti-c-Raf rabbit mAb (1:1000, Cell Signaling, Danvers, MA, USA), anti-Akt rabbit mAb (1:1000, Cell Signaling, Danvers, MA, USA), anti-estrogen receptor α mAb mouse (1:1000, Santa Cruz Biotechnology, Dallas, TX, USA). β-Actin Mouse mAb (1:5000, Sigma-Aldrich, St. Louis, MO, USA). The secondary antibodies used was an anti-IgG, Hrp-linked rabbit antibody (1:10,000, Cell Signaling, Danvers, MA, USA) and anti-IgG, Hrp-linked mouse antibody (1:10,000 Cell Signaling, Danvers, MA, USA). Blots were visualized, using UVITEC Cambridge Imaging System (UVITEC, Cambridge, UK).

## 3. Results and Discussion

### 3.1. Design

**Figure 2:**
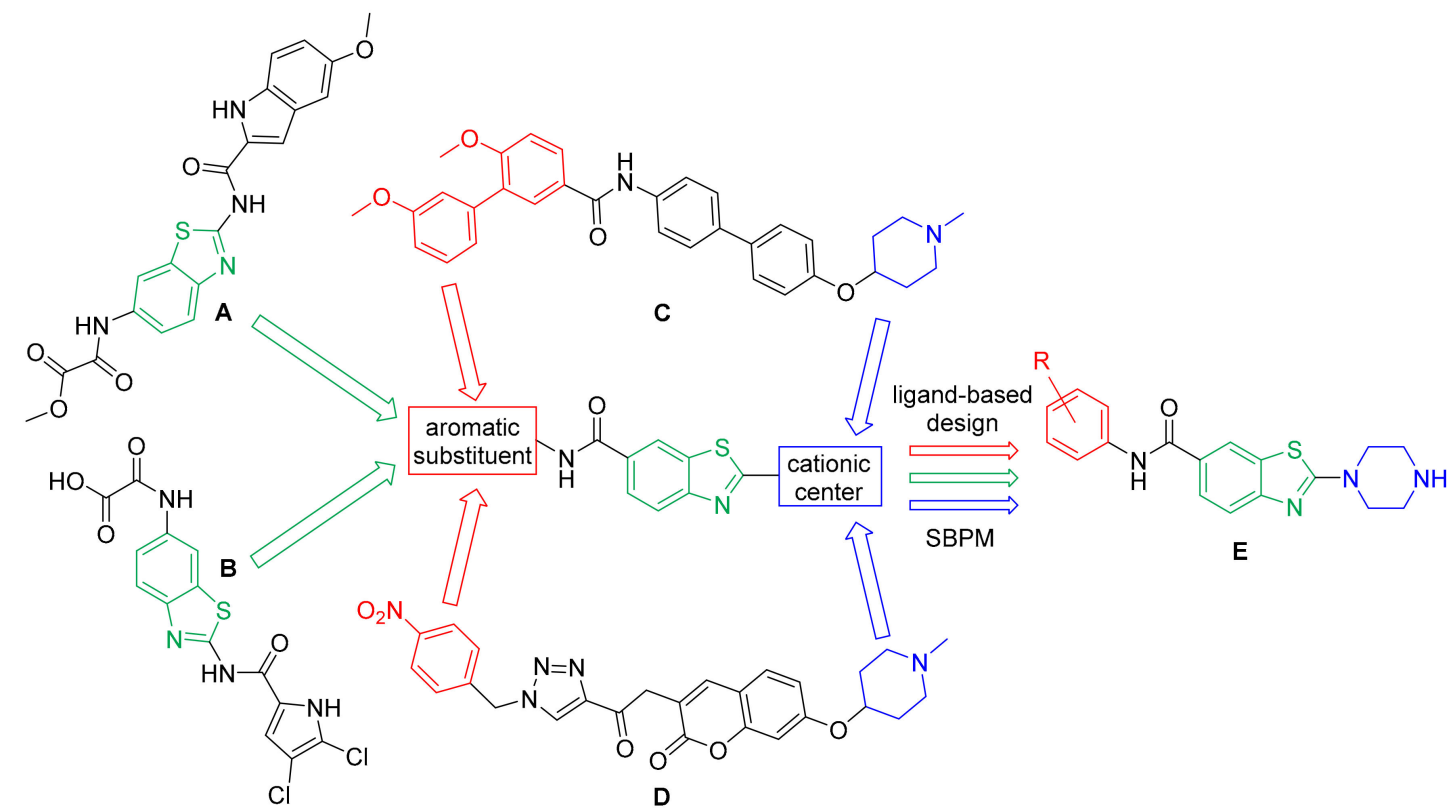
The design strategy started with the known benzothiazole-based Hsp90 C-terminal-domain (CTD) inhibitors **A** [35] and **B** [34]. These were combined with the well-established general structure–activity relationships of Hsp90 CTD inhibitors represented by compounds **C** [29] and **D** [28]. A new compound of class **E** was also designed and included.

In the absence of a co-crystal structure of Hsp90 CTD in complex with a CTD inhibitor, we used a ligand-based design methodology (Figure 2) supported by structure-based pharmacophore models (SBPMs) derived from molecular dynamics (MD) simulations from our previous study [32]. Our aim was to prepare a focused library of benzothiazole-based Hsp90 CTD inhibitors, and to establish the SARs for their Hsp90 inhibition. The benzothiazole moiety was selected as a suitable central scaffold that offers the possibility for the attachment of aromatic substituents and basic amines to positions 2 and 6, which are characteristic for Hsp90 CTD inhibitors (Figure 1). In contrast to the already known benzothiazoles **A** and **B** (Figure 2) [34,35], we decided to introduce aromatic groups at position 6 of the benzothiazole moiety and to replace the amide bond at position 2 with an amine directly linked to the benzothiazole ring (Figure 2). This amine would serve as an attachment point for the various linkers to the basic center. Furthermore, a different orientation of the amide bond was introduced at position 6, in contrast to the already known inhibitors [34,35].

**Figure 3:**
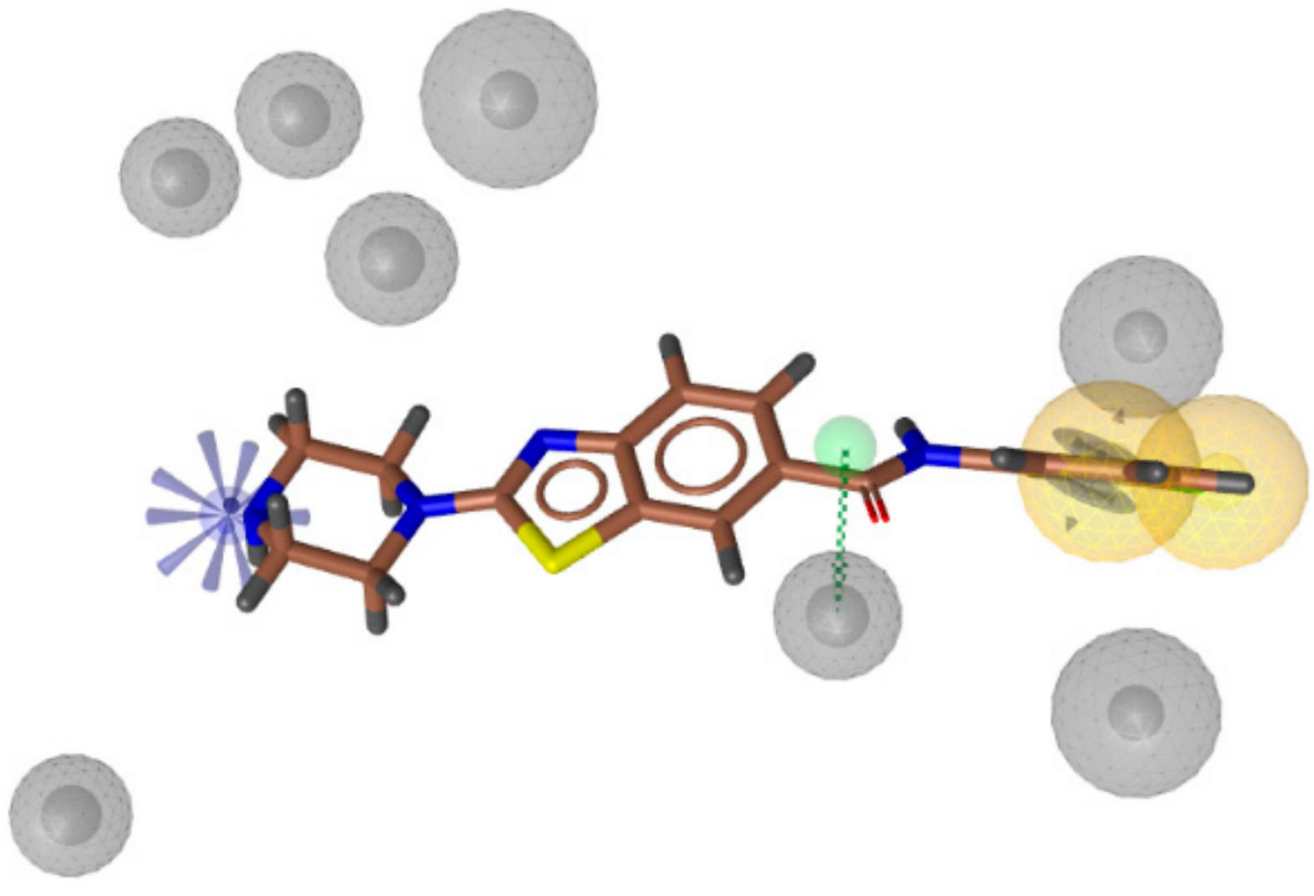
Alignment of a representative compound (R, 3-Cl) of the general structure **E** (brown sticks) with the three-dimensional structure-based pharmacophore model derived from molecular dynamics simulation of a coumarin-based Hsp90 CTD inhibitor in complex with Hsp90β [32]. The pharmacophores included two with hydrophobic features (yellow spheres), one with an aromatic (blue disc), a hydrogen bond (H-bond) donor (green arrow, indicating the defined direction for H-bonding), and a positive ionizable (blue star). The exclusion volumes (grey spheres) define restricted regions based on the shape of the aligned molecules.

To confirm this design strategy, the library of benzothiazoles designed using the strategy shown in Figure 2 was screened against the SBPMs (Figure 3) derived from the MD simulation of a coumarin-based Hsp90 CTD inhibitor in complex with Hsp90β [32]. This pharmacophore model shown in Figure 3 consisted of two hydrophobic features (yellow spheres): one with an aromatic ring feature on one side (blue disc) and a positive ionizable feature on the opposite side (blue star). For screening, these features were marked as essential, while the hydrogen bond donor feature (Figure 3, green arrow) was marked as optional. This last feature is coumarin-ring-specific and not critical for Hsp90 CTD inhibition. Screening was performed for a small library of compounds with the general structure of **E** (Figure 2) and with different substituents on the phenyl ring as R (i.e., H, 3-Cl, 4-Cl, 3-OH, 4-OH). This identified compounds with R as 3-Cl (Figure 3, compound **5b**) or 4-Cl as promising candidates for Hsp90 CTD inhibition, as they provided a good fit to the model. 

From the alignment in Figure 3, it can be seen that further optimization of the distance between the aromatic ring and the basic center was possible. Together with the variation in the phenyl ring substituents, this might provide improved interactions within the binding pocket, and thus we synthesized a focused library of new Hsp90 CTD inhibitors following the design strategy shown in Figure 2.

### 3.2. Synthesis

**Scheme 1:**
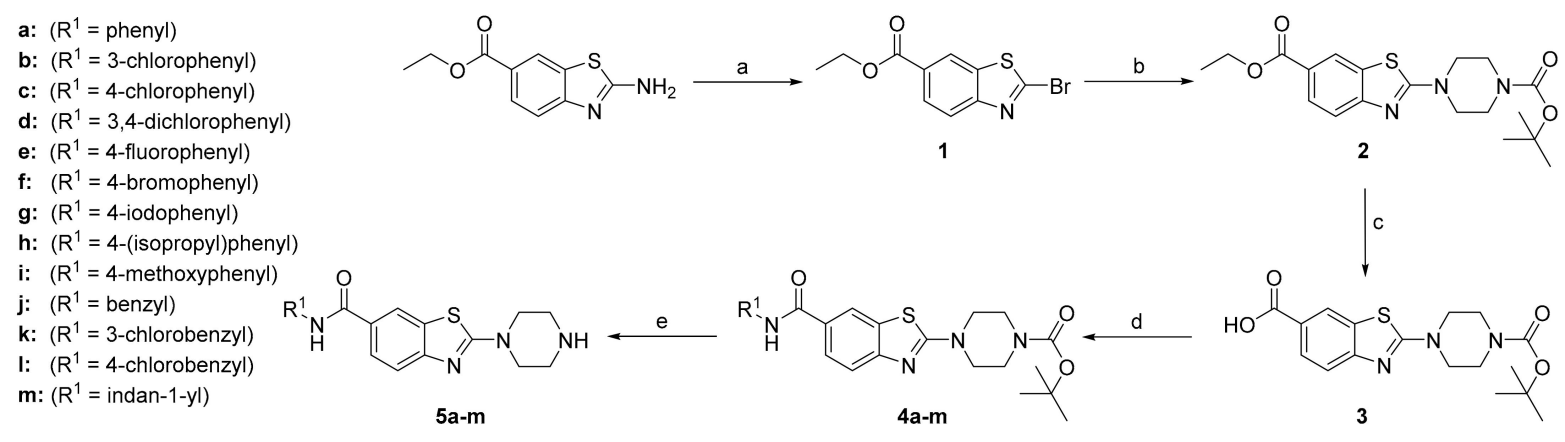
Reagents and conditions: (**a**) CuBr_2_, *t*-BuONO, MeCN, 0 °C to r.t., 2 h; (**b**) 1-Boc-piperazine, THF, r.t., overnight; (**c**) 1 M NaOH, 96% EtOH, 100 °C, 2 h; (**d**) (i) EDC, HOBt, DIPEA, DMF, r.t. 20 min; (ii) respective amine, 1–3 days; (**e**) CF_3_COOH, DCM, r.t., 1–3 days.

To evaluate the impact of the phenyl ring substitutions and variations on the inhibitory activity, compounds **5a–m** were synthesized as shown in Scheme 1. In the first step, a Sandmeyer reaction was performed to generate the 2-bromo derivative **1**, followed by nucleophilic aromatic substitution with 1-Boc protected piperazine, to yield compound **2**. Next, the ethyl ester was hydrolyzed with 1 M NaOH to produce carboxylic acid **3**. In the penultimate step, an aromatic ring was introduced at position 6 through amide coupling, using 1-ethyl-3-(3-dimethylaminopropyl)carbodiimide (EDC) and hydroxybenzotriazole (HOBt) as coupling reagents, to prepare compounds **4a–m**. The final compounds **5a–m** were synthesized by acidolysis of **4a–m**, using trifluoroacetic acid in dichloromethane (DCM).

**Scheme 2:**
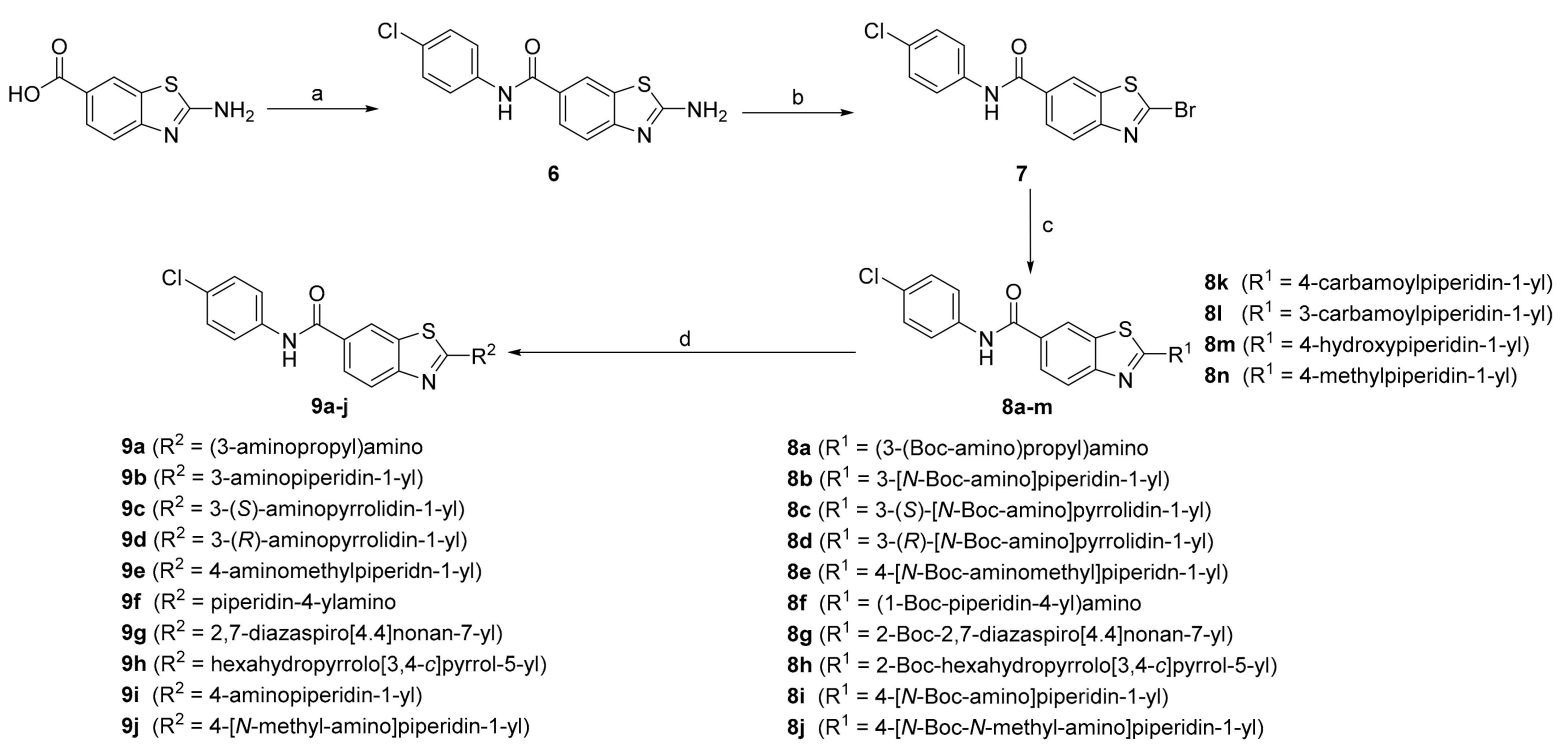
Reagents and conditions: (**a**) (i) EDC, HOBt, DIPEA, DMF, r.t. 20 min; (ii) 4-chloroaniline, r.t., overnight; (**b**) CuBr_2_, *t*-BuONO, MeCN, 0 °C to r.t., overnight; (**c**) respective amine, Et_3_N, THF, r.t., overnight; (**d**) CF_3_COOH, DCM, r.t., 1–3 days.

To investigate the SARs at position 2 of the benzothiazole core (Scheme 2), 2-aminobenzo[*d*]thiazole-6-carboxylic acid was used as the starting point for the synthesis. First, 4-chloroaniline was coupled to the carboxylic acid at position 6 of the benzothiazole to provide **6**. Subsequently, a Sandmeyer reaction was carried out to substitute the aromatic amine at position 2 with bromine (**7**). Aromatic nucleophilic substitution was then used to introduce various amines at position 2 of the benzothiazole to generate the final compounds without cationic centers, **8k–n**. This reaction was also used to prepare compounds **8a–j** with Boc protected amines, which were then Boc-deprotected by acidolysis to synthesize the final products **9a–j**.

**Scheme 3:**
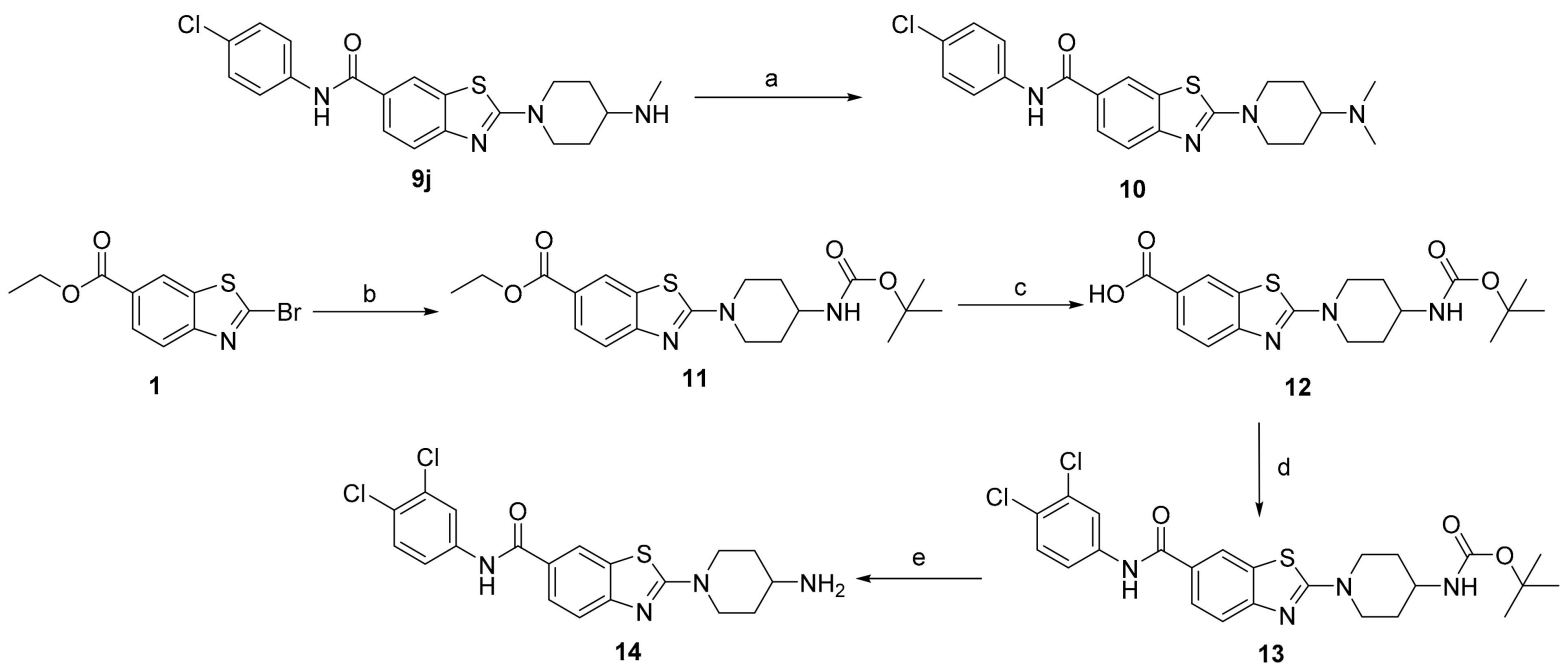
Reagents and conditions: (**a**) (i) formaldehyde, DCM:MeOH = 1:1, r.t., 15 min; (ii) NaCNBH_3_, r.t., overnight; (**b**) 4-(*N*-Boc-amino)piperidine, THF, from r.t. to 70 °C, overnight; (**c**) 1 M NaOH, 96% EtOH, 100 °C, 2 h; (**d**) (i) EDC, HOBt, DIPEA, DMF, r.t., 20 min; (ii) respective amine, 1–3 days; (**e**) CF_3_COOH, DCM, r.t., 1–3 days.

To further establish the SARs, two additional analogs of compound **9i** were prepared as well as the monomethylated analog **9j**. From **9j**, a dimethylated analog **10** was synthesized using reductive amination, as shown in Scheme 3. Compound **14** was synthesized from compound **1** as shown in Scheme 3. First the 4-(*N*-Boc-amino)piperidine was introduced at position 2 of the benzothiazole ring to generate compound **11**. Then, the ester was hydrolyzed to prepare compound **12**, to which the 3,4-dichloroaniline was introduced using amide coupling, to generate compound **13**. Ultimately, the Boc protection group was removed by acidolysis to yield the final compound **14**.

### 3.3. Biological Evaluation

All of the final compounds were tested for antiproliferative activities against the MCF-7 breast cancer cell line, using the MTS assay. The results obtained from the introduction of the changes in the substituents at position 6 are shown in Table 1. The unsubstituted benzene ring at this position provided the moderately active compound **5a** (IC_50_, 18.9 ± 0.4 μM), even though it lacked an additional hydrophobic substituent that is relevant for binding according to the pharmacophore model shown in Figure 3. Therefore, the introduction of a chlorine atom at the *meta* and/or *para* positions of the phenyl ring was designed to form additional hydrophobic interactions with the hydrophobic pocket of the proposed CTD binding site. Both substitution positions resulted in compounds **5b–d** with similar potencies. The possibility of compounds to form a halogen bond was investigated by introducing fluorine (**5e**), bromine (**5f**), and iodine (**5g**) at the *para* position of the phenyl ring. The gradual increase in activity from **5c** to the most potent compound **5g** (IC_50_, 2.8 ± 0.1 μM) correlated with the formation of halogen bonds by the individual atoms; however, the increase in activity might also be attributed to improved steric fit with the larger iodine substituent in **5g**. This last is also in agreement with compound **5h**, which suggests that there might be additional unused space in this part of the binding pocket. The introduction of the polar 4-methoxy group (**5i**) or increasing the distance between the basic center and aromatic moiety by introducing additional methylene groups (**5j**–**m**), did not further improve the activity with respect to **5a**.

As chlorine is a more drug-like element than iodine and bromine [49], and as compound **5c** showed promising activity in the cell-based assay, *para*-chlorophenyl was chosen as a suitable substituent on position 6 for further SAR exploration of position 2 on the benzothiazole ring. The antiproliferative activities against the MCF-7 cell line of these compounds are shown in Table 2.

First, the importance of the cationic center in the compounds was evaluated. This was because the previously developed benzothiazole-based Hsp90 CTD inhibitors did not contain a cationic center in their structure [34,35]. For this purpose, variously substituted piperidines were introduced at position 2 of the benzothiazole ring. The antiproliferative IC_50_ values for 4-methyl- (**8n**), 4-hydroxy- (**8m**), 4-carbamoyl- (**8k**) and 3-carbamoyl- (**8l**) piperidines were all >50 μM. Therefore, these compounds were considered inactive, and the importance of the cationic center for this inhibitor class was confirmed.

Next, the piperazine ring at position 2 of compound **5c** was replaced by 1,3-diaminopropane (**9a**). This change resulted in an expected decrease in activity (IC_50_, 8.3 ± 2.4 μM), as the propyl chain is more flexible than piperazine. In compounds **9b–9d**, the substituents in position **2** maintained approximately the same spacing of two carbon atoms between the two nitrogen atoms as in piperazine, which had a very limited effect on the activity (IC_50_, from 5.5 ± 0.7 to 7.1 ± 1.4 μM). The reduced potency of compound **9e** (IC_50_, 11.4 ± 0.8 μM) with a longer 4-aminomethyl-piperidine substituent highlights the importance of an appropriate distance between the basic center and the aromatic ring, as shown in the pharmacophore model in Figure 3. The optimal distance between the aromatic ring at position 6 of the benzothiazole and the cationic center appears to have been achieved with compounds **9g–9i**. Although the substituents of these inhibitors differed, the distances to the cationic center were similar. As compound **9i** was the most potent (IC_50_, 3.9 ± 0.1 μM), it was selected for further SAR investigations.

First, *N-*methyl (**9j**) and *N-*dimethyl (**10**) analogs of the primary amine of **9i** were prepared and tested. The results, presented in Table 3 show that neither modification resulted in improved activity. The monomethylated analog **9j** showed an almost unchanged potency (IC_50_, 4.2 ± 0.9 μM) with respect to **9i**, with a slight decrease in activity for the tertiary amine **10** (IC_50_, 5.3 ± 1.1 μM). This was not in agreement with our expectations, as many previously developed potent Hsp90 CTD inhibitors have contained a tertiary amine moiety in their structure [28,29,31]. In addition, compound **14** (Table 3) was synthesized as an analog of compound **5d**. Interestingly, with compound **14**, the antiproliferative activity was half that of **9i** (IC_50_, 7.4 ± 0.5 vs. 3.9 ± 0.1 μM, respectively).

**Figure 4:**
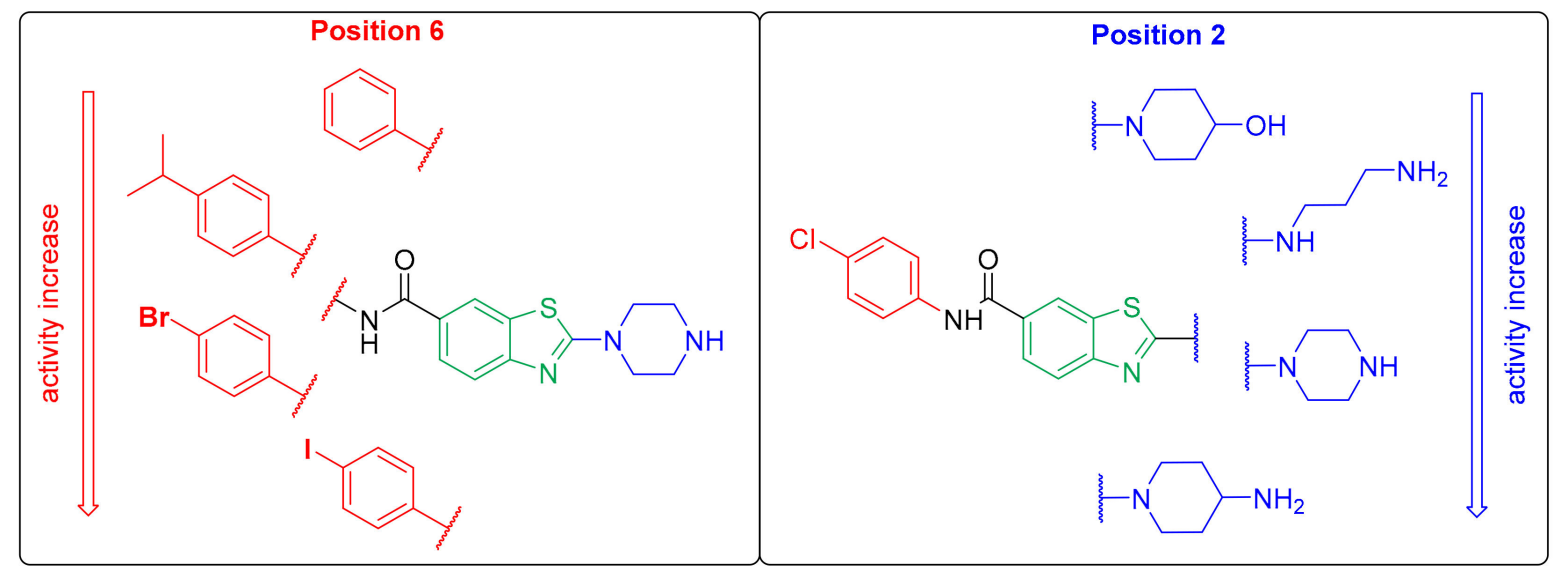
Structure–activity relationships of the benzothiazole based Hsp90 C-terminal-domain inhibitors.

Overall, the results of the biological evaluation of the antiproliferative activities against the MCF-7 cell line established the general SARs for this set of compounds. As shown in Figure 4, the cationic center at position 2 is obligatory, and its correct distance from the aromatic ring at position 6 is also very important.

**Figure 5:**
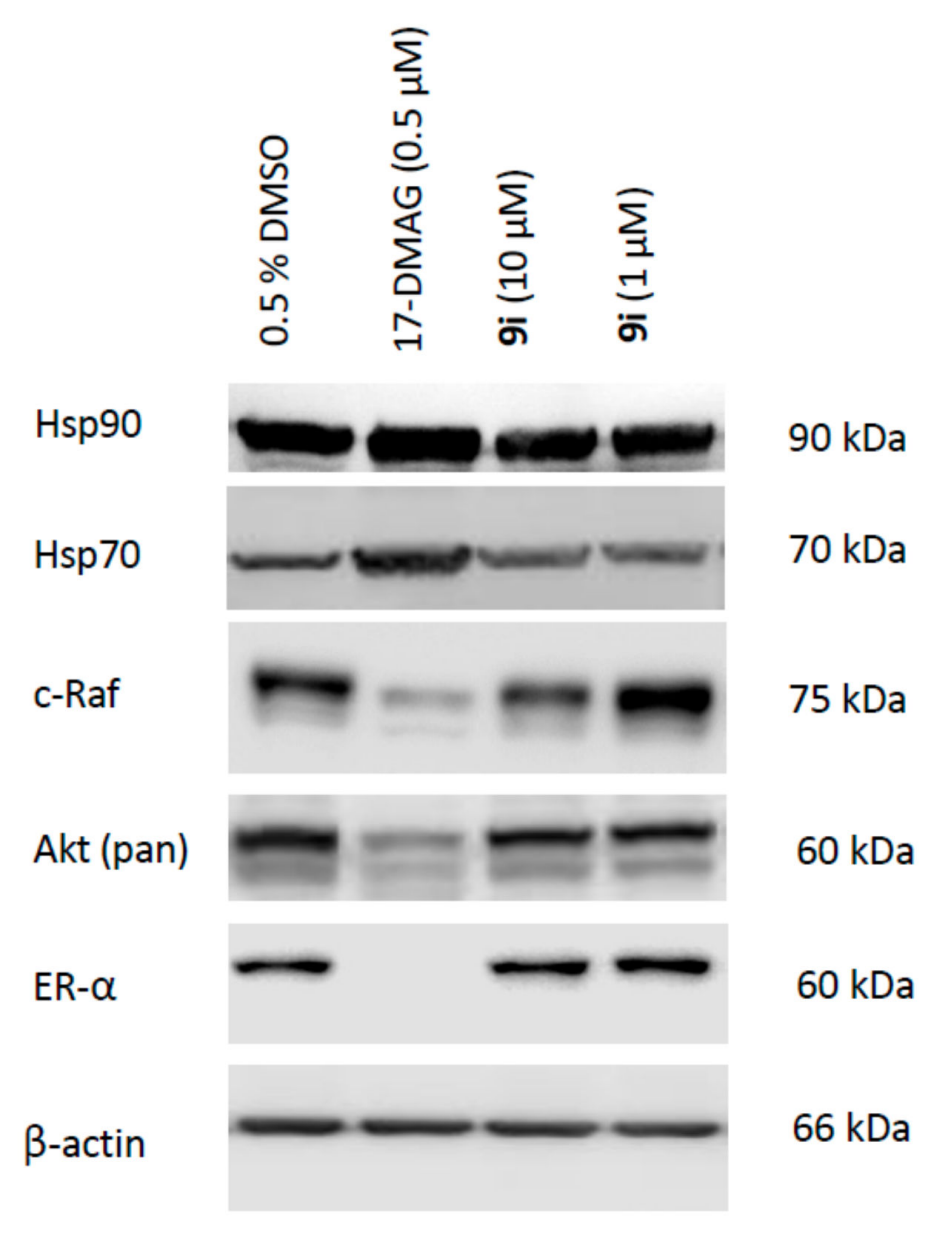
Representative Western blotting for the MCF-7 breast cancer cell line for Hsp90, Hsp70, Akt, c-Raf, and estrogen receptor alpha (ER-α) after 24 h incubation with compound **9i**, 17-DMAG (positive control) and 0.5% DMSO (vehicle).

To confirm that these inhibitors alter the biological activity of Hsp90, the expression levels of some of its client proteins that are relevant to cancer pathogenesis were examined using Western blotting: Akt, c-Raf, and estrogen receptor alpha (ERα). In addition, the expression levels of Hsp90 and Hsp70 in the treated cells were monitored to confirm that no HSR induction occurs, as non-induction of HSR is one of the main advantages of Hsp90 CTD inhibitors compared to ATP-competitive isoform non-selective inhibitors. As shown in Figure 5, Western blotting of lysates from the MCF-7 cells after treatment with compound **9i** confirmed that the oncogenic proteins Akt, c-Raf, and ERα were downregulated. At the same time, compound **9i** did not induce the HSR, as Hsp90 and Hsp70 were not upregulated. On the contrary, a known Hsp90 NTD inhibitor 17-DMAG that was used as a positive control resulted in significant upregulation of these heat shock proteins. These data confirmed that this new class of inhibitor modulates the activity of Hsp90 in a CTD allosteric manner, similar to the previously reported benzothiazole-based CTD inhibitors [35]. 

### 3.4. Molecular Modeling

**Figure 6:**
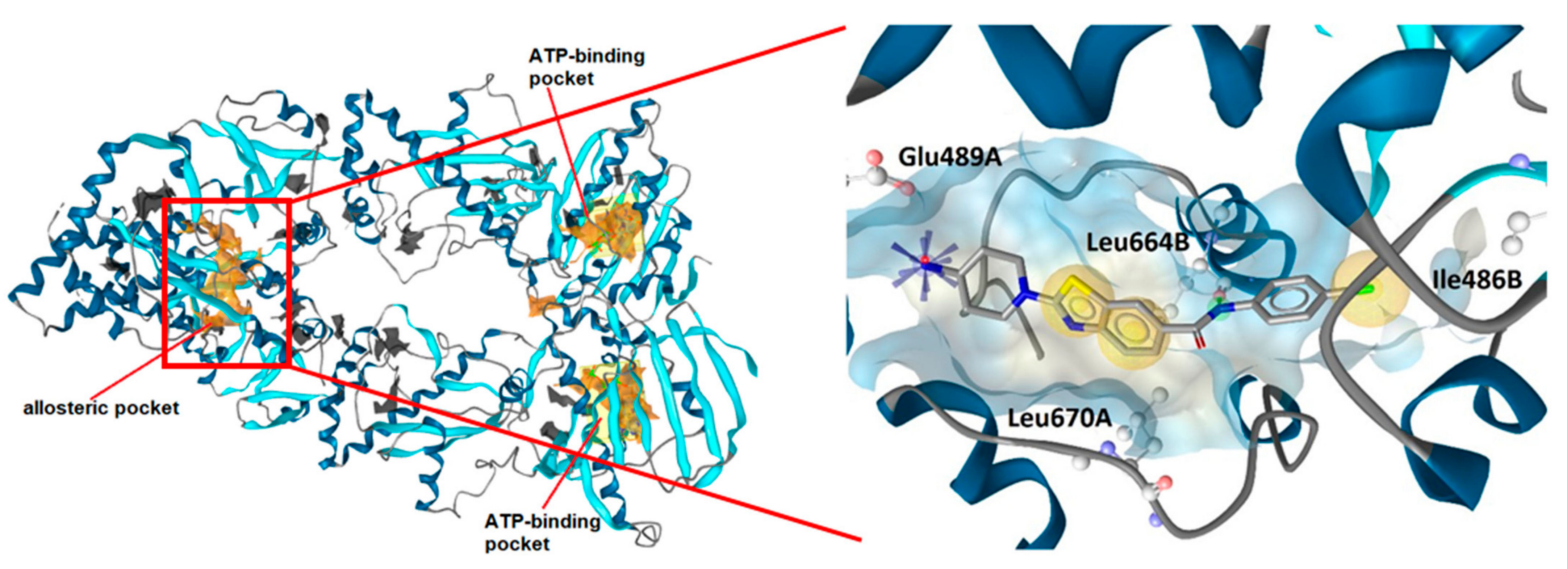
Interactions of compound **9i** with the Hsp90 C-terminal-domain binding site. The pharmacophore features are hydrophobics (yellow spheres), hydrogen bond donor (green arrow), and positive ionizable (blue star). For clarity, only the amino acids that interact with **9i** are shown.

To study the possible binding mode of these benzothiazole-based Hsp90 CTD inhibitors, as **9i** was one of the most potent compounds, it was docked in the Hsp90β CTD binding site in the conformation from the MD simulation trajectory from which the SBPM used for alignment in Figure 3 was derived [32]. From the analysis of the binding mode (Figure 6), it is apparent that the cationic center at position 2 of the benzothiazole forms an ionic interaction with the Glu489A side chain, while the benzothiazole scaffold forms hydrophobic interactions with the Leu664B and Leu670A side chains. The amide NH group forms a hydrogen bond with the Leu664B backbone carbonyl group, and the 4-chlorophenyl ring interacts with the Ile486B side chain. Eight out of ten highest ranked docking poses of **9i** were predicted to adopt similar orientation in the binding site and form similar interactions.

**Figure 7:**
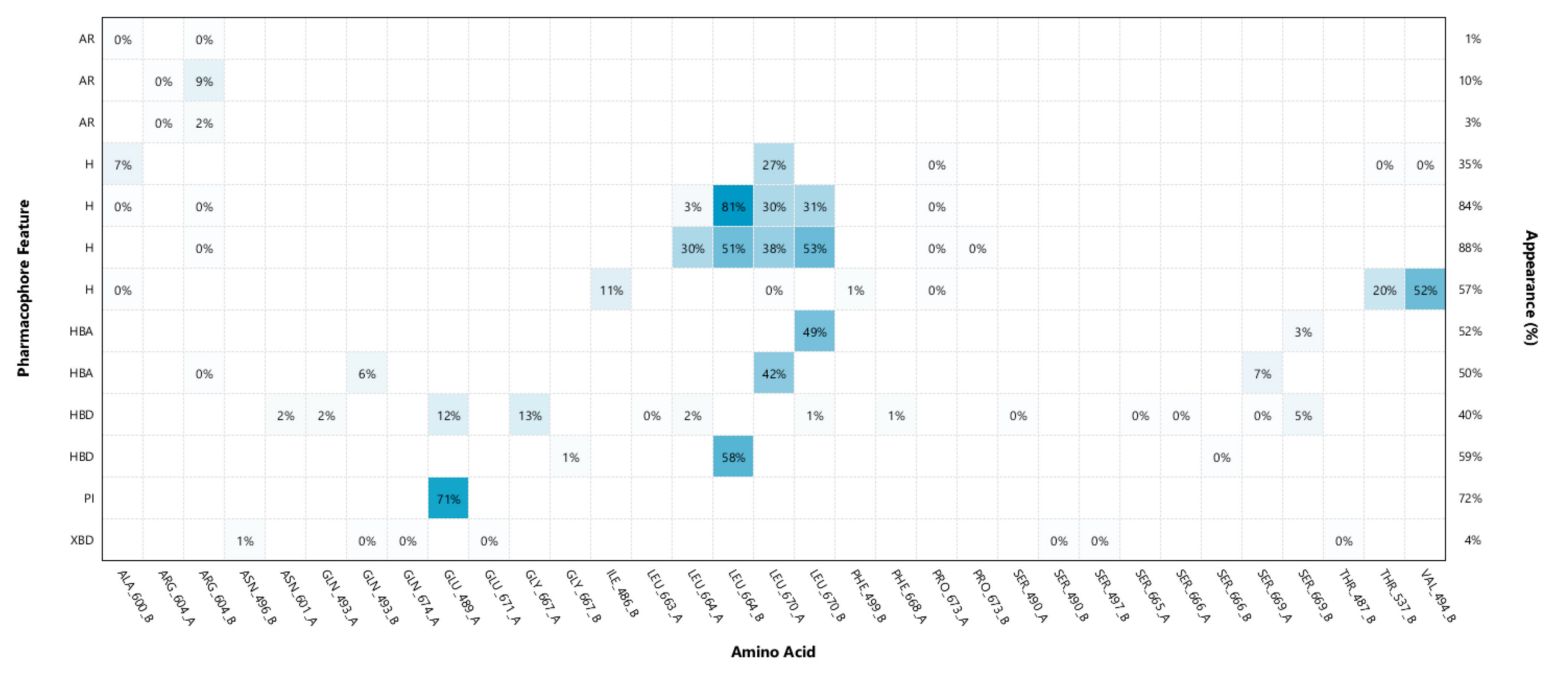
MD Interaction map obtained by analyzing interactions of compound **9i** with Hsp90 CTD binding site residues in the 500 ns MD simulation trajectory. Amino acid name and numbering is shown on x-axis, pharmacophore feature type on the left y-axis (H—hydrophobic, HBA—hydrogen bond acceptor, HBD—hydrogen bond donor, PI—positive ionizable, XBD—halogen bond), % appearance on the right y-axis.

**Figure 8:**
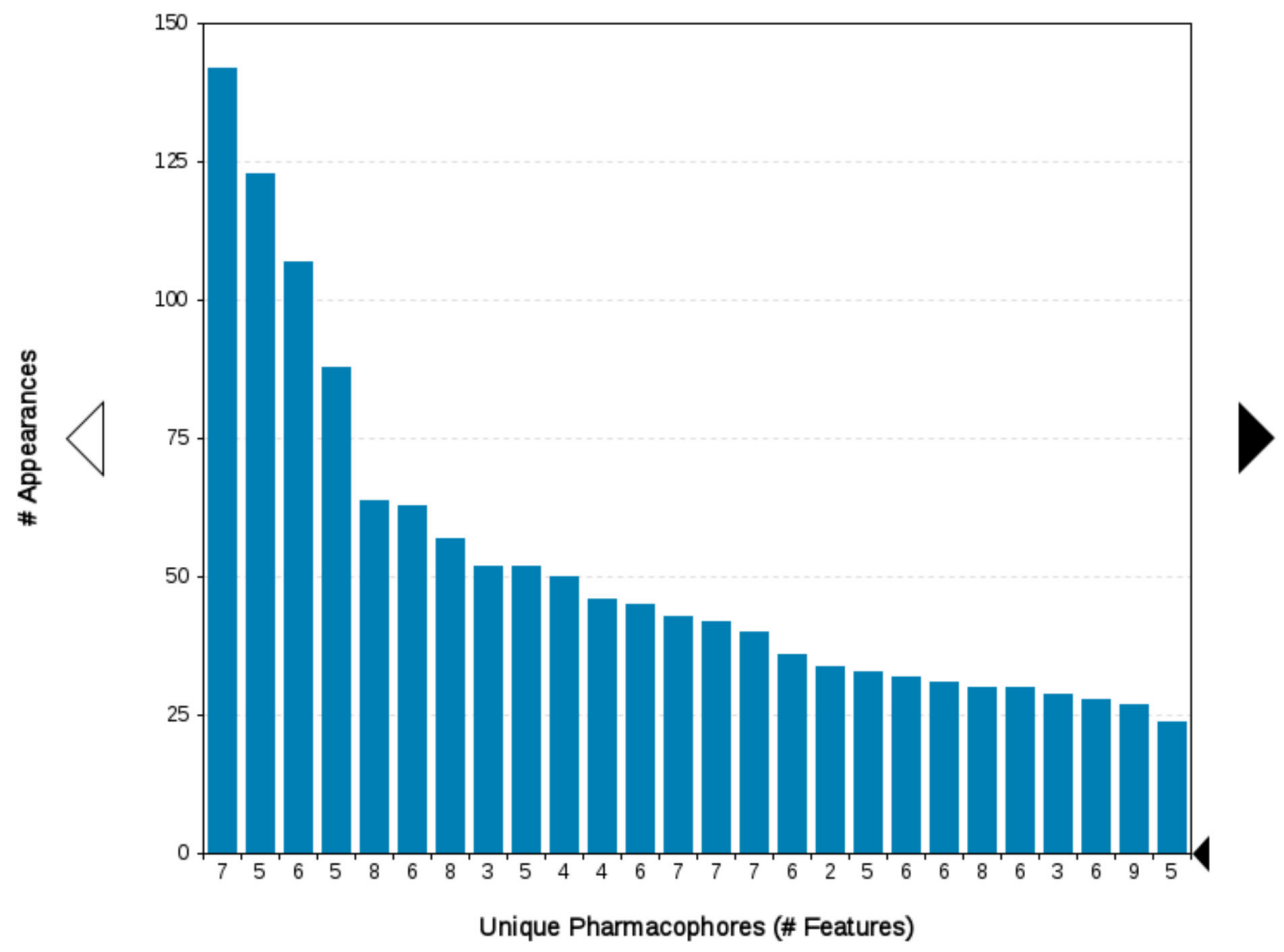
Plot of the most frequent unique structure-based pharmacophore models derived from the molecular dynamics simulations of the Hsp90 C-terminal domain in complex with **9i**. The numbers below the bars indicate the numbers of interaction features observed during molecular dynamics simulation for the pharmacophore models.

**Figure 9:**
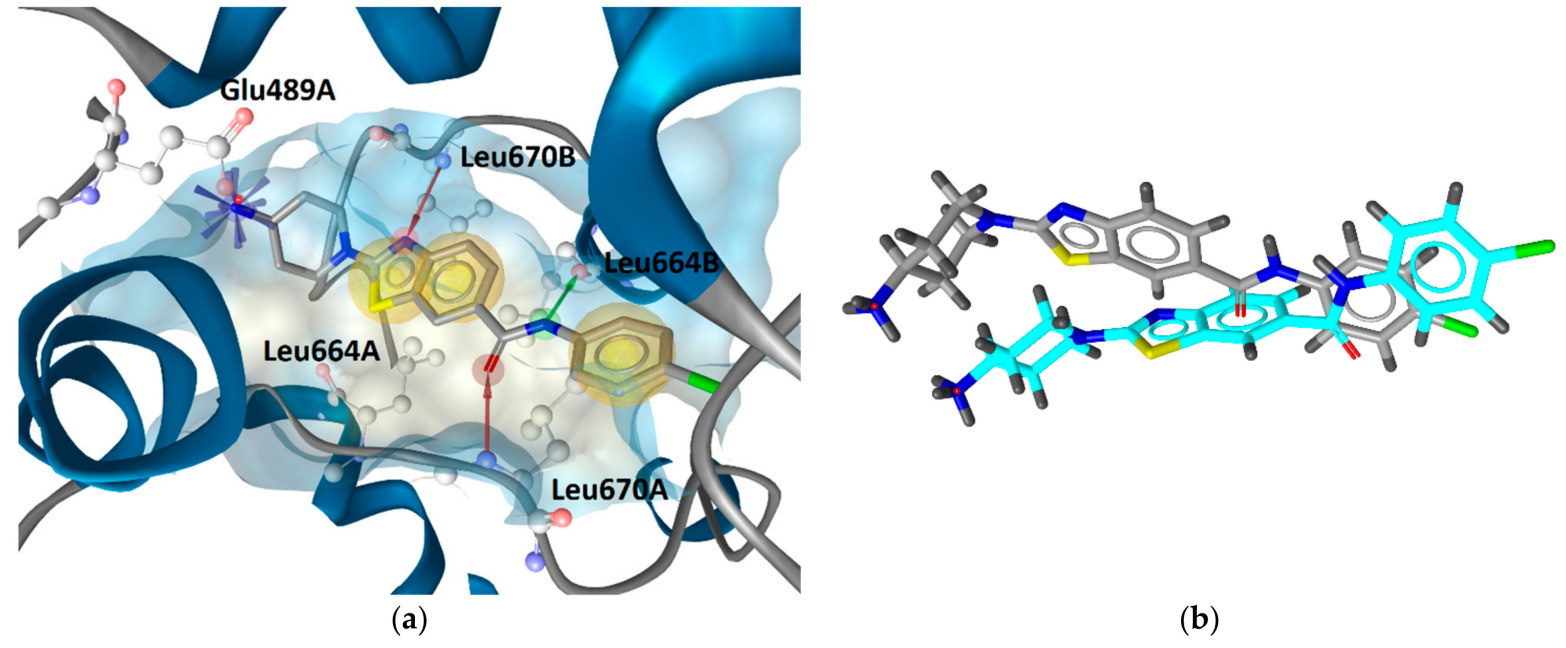
(**a**) The most frequently occurring interactions and binding modes of compound **9i** in the Hsp90 C-terminal-domain binding site during the 500 ns molecular dynamics simulation. The pharmacophore features are as follows: hydrophobics (yellow spheres), hydrogen bond donor (green arrow), hydrogen bond acceptor (red arrow), and positive ionizable (blue star). For clarity, only the amino acids that interact with **9i** are shown. (**b**) Superposition of the docking pose (in cyan) and the MD conformation (in grey) of **9i** representing the most frequently appearing pharmacophore model.

In the absence of any co-crystal structure of the Hsp90–CTD inhibitor complex, we further validated the proposed binding mode of **9i** using 500 ns MD simulations. The interaction features between **9i** and the allosteric Hsp90 CTD binding site during the MD simulation were analyzed using the MD analysis tool in LigandScout 4.4 Expert. The MD interaction map in Figure 7 shows the percentage appearance of each pharmacophore feature during the MD trajectory and the amino acid residues associated with these features. The most conserved interactions during the MD simulation are ionic interactions with the Glu489A side chain (71%), hydrophobic interactions with Leu664B (81%), Leu670B (53%) and hydrogen bonds with Leu664B (58%), Leu670A (42%) and Leu670B (49%). Figure 8 shows the plot of the most frequently appearing unique SBPMs, in terms of the total number of interaction features for each SBPM versus the frequency (#Appearances). The most frequent model (seen 141 times) showed 7 interaction features, including a positive ionizable feature associated with the primary amine and Glu489A, hydrophobic interactions with Leu664A, Leu670A, Leu664B, and Leu670B, and hydrogen bonds with Leu664B, Leu670A, and Leu670B (Figure 9). This pharmacophore model frequently appeared between 280 ns and 500 ns of the MD simulation. The second most frequent model (seen 123 times) was the same as the docking model shown in Figure 6 and is represented in the first part of the MD simulation (until 140 ns). However, the binding pose of **9i** does not significantly change between the docking pose and poses derived from the MD simulation trajectory (Figure 9).

The Hsp90 CTD binding site of **9i** partially overlaps with the previously studied binding sites [30,50]. The amino acid residues Glu489 (Glu497 in Hsp90α), Ser669 (Ser677 in Hsp90α), and Leu664 (Leu672 in Hsp90α) that interact with **9i** (Figure 7) also form ionic interactions and hydrogen bonds with bisphenol derivative [50] and novobiocin analogues [30]. In addition, **9i** forms hydrophobic contacts with Ile486 (Ile494 in Hsp90α) (Figure 7), which was identified by protein NMR spectroscopy studies as important for the binding of novobiocin analogues KU-32 and KU-596 to Hsp90 CTD [51]. However, this binding site does not overlap with that of the peptide-based inhibitor AX [52].

## 4. Conclusions

A focused library of novel benzothiazole-based Hsp90 CTD inhibitors was designed by combining ligand-based and structure-based pharmacophore modeling. To develop the SARs of this new Hsp90 CTD inhibitor class, a series of 2,6-disubstituted benzothiazoles was synthesized and biologically evaluated. The most potent compounds showed low micromolar antiproliferative activities against the MCF-7 breast cancer cell line. Compound **9i** showed dose-dependent degradation of Hsp90 client proteins and did not induce the HSR, which is a characteristic feature of Hsp90 CTD inhibitors. In the absence of a crystal structure of a Hsp90-CTD inhibitor complex, the binding mode of **9i** was investigated through a combination of docking and MD simulations in conjunction with structure-based pharmacophore modeling, which were consistent with the observed SARs. Analysis of the binding interactions of **9i** in the Hsp90 CTD binding site during MD simulation revealed conserved interactions with several amino acid residues that can be used for the design of novel Hsp90 CTD inhibitors. The results of this study highlight the benzothiazole moiety as a suitable scaffold for the design of Hsp90 CTD inhibitors with antiproliferative activities and provide the basis for structure-based optimization toward more potent compounds.

## Figures and Tables

**Table 1:** IC_50_ values for the antiproliferative activities of the synthesized benzothiazoles **5a**–**m** in the MCF-7 breast cancer cell line: exploration of the structure–activity relationships at position 6 of the benzothiazole moiety.

Compound	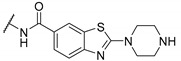	IC_50_ (μM)
**17-DMAG** *		0.9 ± 0.1
**5a**	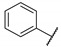	18.9 ± 0.4
**5b**	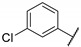	6.5 ± 0.1
**5c**	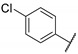	7.2 ± 0.0
**5d**	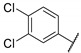	6.3 ± 0.5
**5e**	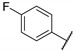	17.7 ± 3.7
**5f**	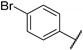	4.3 ± 0.0
**5g**	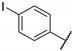	2.8 ± 0.1
**5h**	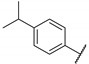	6.6 ± 0.4
**5i**	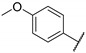	22.3 ± 5.1
**5j**	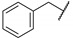	35.4 ± 8.6
**5k**	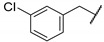	13.3 ± 1.5
**5l**	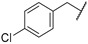	15.0 ± 0.1
**5m**	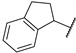	20.1 ± 0.2

* **17-DMAG**, 17-dimethylaminoethylamino-17-demethoxygeldanamycin (Hsp90 N-terminal-domain inhibitor used as a positive control in all experiments).

**Table 2:** IC_50_ values for the antiproliferative activities of the synthesized benzothiazoles **8k–n** and **9a–i** in the MCF-7 breast cancer cell line: exploration of the structure–activity relationships at position 2 of the benzothiazole moiety.

Compound	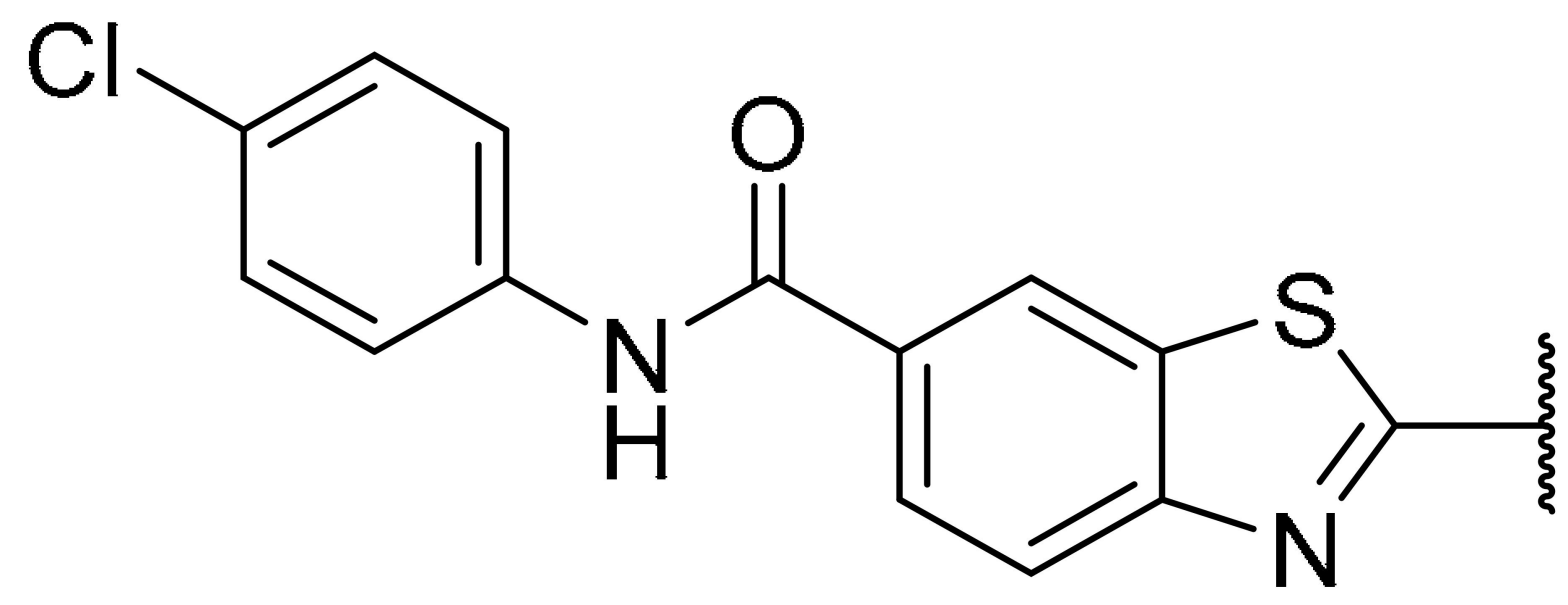	IC_50_ (μM)
**8k**	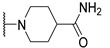	>50
**8l**	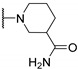	>50
**8m**	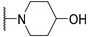	>50
**8n**	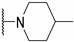	>50
**9a**	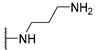	8.3 ± 2.4
**9b**	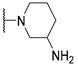	6.7 ± 0.1
**9c**	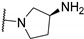	5.5 ± 0.7
**9d**	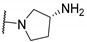	7.1 ± 1.4
**9e**	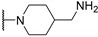	11.4 ± 0.8
**9f**	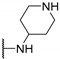	6.2 ± 0.1
**9g**	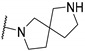	4.2 ± 1.0
**9h**	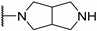	4.1 ± 0.3
**9i**	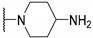	3.9 ± 0.1

**Table 3:** IC_50_ values for the antiproliferative activities of the **9i** analogs evaluated in the MCF-7 breast cancer cell line.

Compound	Structure	IC_50_ (μM)
**9j**	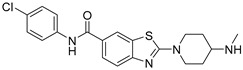	4.2 ± 1.0
**10**	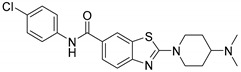	5.3 ± 1.1
**14**	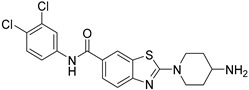	7.4 ± 0.5

## Data Availability

The data presented in this study are available on request from the corresponding author.

## Supplementary Materials

The following are available online at https://www.mdpi.com/article/10.3390/pharmaceutics13081283/s1, Figure S1. 1H NMR spectrum of compound **1**, Figure S2. 13C NMR spectrum of compound **1**, Figure S3. 1H NMR spectrum of compound **2**, Figure S4. 13C NMR spectrum of compound **2**, Figure S5. 1H NMR spectrum of compound **3**, Figure S6. 13C NMR spectrum of compound **3**, Figure S7. 1H NMR spectrum of compound **4c**, Figure S8. 1H NMR spectrum of compound **5a**, Figure S9. 13C NMR spectrum of compound **5a**, Figure S10. HPLC chromatogram for compound **5a**, Figure S11. 1H NMR spectrum of compound **5b**, Figure S12. 13C NMR spectrum of compound **5b**, Figure S13. HPLC chromatogram for compound **5b**, Figure S14. 1H NMR spectrum of compound **5c**, Figure S15. 13C NMR spectrum of compound **5c**, Figure S16. HPLC chromatogram for compound **5c**, Figure S17. 1H NMR spectrum of compound **5d**, Figure S18. 13C NMR spectrum of compound **5d**, Figure S19. HPLC chromatogram for compound **5d**, Figure S20. 1H NMR spectrum of compound **5e**, Figure S21. 19F NMR spectrum of compound **5e**, Figure S22. 13C NMR spectrum of compound **5e**, Figure S23. HPLC chromatogram for compound **5e**, Figure S24. 1H NMR spectrum of compound **5f**, Figure S25. 13C NMR spectrum of compound **5f**, Figure S26. HPLC chromatogram for compound **5f**, Figure S27. 1H NMR spectrum of compound **5g**, Figure S28. 13C NMR spectrum of compound **5g**, Figure S29. HPLC chromatogram for compound **5g**, Figure S30. 1H NMR spectrum of compound **5h**, Figure S31. 13C NMR spectrum of compound **5h**, Figure S32. HPLC chromatogram for compound **5h**, Figure S33. 1H NMR spectrum of compound **5i**, Figure S34. 13C NMR spectrum of compound **5i**, Figure S35. HPLC chromatogram for compound **5i**, Figure S36. 1H NMR spectrum of compound **5j**, Figure S37. 13C NMR spectrum of compound **5j**, Figure S38. HPLC chromatogram for compound **5j**, Figure S39. 1H NMR spectrum of compound **5k**, Figure S40. 13C NMR spectrum of compound **5k**, Figure S41. HPLC chromatogram for compound **5k**, Figure S42. 1H NMR spectrum of compound **5l**, Figure S43. 13C NMR spectrum of compound **5l**, Figure S44. HPLC chromatogram for compound **5l**, Figure S45. 1H NMR spectrum of compound **5m**, Figure S46. 13C NMR spectrum of compound **5m**, Figure S47. HPLC chromatogram for compound **5m**, Figure S48. 1H NMR spectrum of compound **6**, Figure S49. 13C NMR spectrum of compound **6**, Figure S50. 1H NMR spectrum of compound **7**, Figure S51. 13C NMR spectrum of compound **7**, Figure S52. 1H NMR spectrum of compound **8i**, Figure S53. 13C NMR spectrum of compound **8i**, Figure S54 1H NMR spectrum of compound **8k**, Figure S55. 13C NMR spectrum of compound **8k**, Figure S56. HPLC chromatogram for compound **8k**, Figure S57 1H NMR spectrum of compound **8l**, Figure S58. 13C NMR spectrum of compound **8l**, Figure S59. HPLC chromatogram for compound **8l**, Figure S60 1H NMR spectrum of compound **8m**, Figure S61. 13C NMR spectrum of compound **8m**, Figure S62. HPLC chromatogram for compound **8m**, Figure S63 1H NMR spectrum of compound **8n**, Figure S64. 13C NMR spectrum of compound **8n**, Figure S65. HPLC chromatogram for compound **8n**, Figure S66 1H NMR spectrum of compound **9a**, Figure S67. 13C NMR spectrum of compound **9a**, Figure S68. HPLC chromatogram for compound **9a**, Figure S69 1H NMR spectrum of compound **9b**, Figure S70. 13C NMR spectrum of compound **9b**, Figure S71. HPLC chromatogram for compound **9b**, Figure S72 1H NMR spectrum of compound **9c**, Figure S73. 13C NMR spectrum of compound **9c**, Figure S74. HPLC chromatogram for compound **9c**, Figure S75 1H NMR spectrum of compound **9d**, Figure S76. 13C NMR spectrum of compound **9d**, Figure S77. HPLC chromatogram for compound **9d**, Figure S78 1H NMR spectrum of compound **9e**, Figure S79. 13C NMR spectrum of compound **9e**, Figure S80. HPLC chromatogram for compound **9e**, Figure S81 1H NMR spectrum of compound **9f**, Figure S82. 13C NMR spectrum of compound **9f**, Figure S83. HPLC chromatogram for compound **9f**, Figure S84 1H NMR spectrum of compound **9g**, Figure S85. 13C NMR spectrum of compound **9g**, Figure S86. HPLC chromatogram for compound **9g**, Figure S87 1H NMR spectrum of compound **9h**, Figure S88. 13C NMR spectrum of compound **9h**, Figure S89. HPLC chromatogram for compound **9h**, Figure S90 1H NMR spectrum of compound **9i**, Figure S91. 13C NMR spectrum of compound **9i**, Figure S92. HPLC chromatogram for compound **9i**, Figure S93 1H NMR spectrum of compound **9j**, Figure S94. 13C NMR spectrum of compound **9j**, Figure S95. HPLC chromatogram for compound **9j**, Figure S96 1H NMR spectrum of compound **10**, Figure S97. 13C NMR spectrum of compound **10**, Figure S98. HPLC chromatogram for compound **10**, Figure S99 1H NMR spectrum of compound **14**, Figure S100. 13C NMR spectrum of compound **14**, Figure S101. HPLC chromatogram for compound **14**.

## Appendix A

**Ethyl 2-bromobenzo[*d*]thiazole-6-carboxylate (1):** Ethyl 2-aminobenzo[*d*]thiazole-6-carboxylate (4.00 g, 18.00 mmol) and CuBr_2_ (8.04 g, 35.99 mmol) were dissolved in acetonitrile (90 mL). *tert*-Butyl nitrite (4.28 mL, 35.99 mmol, 867 mg mL^−1^) was added in an ice bath, and the reaction mixture was stirred for 1 h at room temperature. The solvent was then removed under reduced pressure, and the residue was taken up in ethyl acetate (150 mL) and saturated NH_4_Cl (50 mL). The organic phase was additionally washed with saturated NH_4_Cl (2 × 50 mL) and brine (50 mL). The organic phase was dried over Na_2_SO_4_, filtered, and the solvent was evaporated under reduced pressure. Yield: 3.95 g (77%); brown amorphous powder; R_f_ (DCM:MeOH = 30:1) = 0.74; ^1^H NMR (400 MHz, CDCl_3_, 25 °C, TMS): δ = 8.55 (d, *J* = 1.5 Hz, 1H, Ar-*H*_7_), 8.16 (dd, *J*_1_ = 8.5 Hz, *J*_2_ = 1.5 Hz, 1H, Ar-*H*_5_), 8.02 (d, *J* = 8.5 Hz, 1H, Ar-*H*_4_), 4.43 (q, *J* = 7.1 Hz, 2H, COO-C*H*_2_-CH_3_), 1.43 ppm (t, *J* = 7.1 Hz, 3H, COO-CH_2_-C*H*_3_); ^13^C NMR (101 MHz, CDCl_3_, 25 °C, TMS): δ = 165.8, 155.1, 142.4, 137.3, 127.9, 127.8, 122.9, 122.5, 61.5, 14.4 ppm; LC–MS (ESI+): *m/z* 285.9 [M+H]^+^ (calcd. *m/z* = 284.9 for C_10_H_8_BrNO_2_S).

**Ethyl 2-(4-Boc-piperazin-1-yl)benzo[*d*]thiazole-6-carboxylate (2):** Ethyl 2-bromobenzo[*d*]thiazole-6-carboxylate (1.98 g, 6.91 mmol) and 1-Boc-piperazine (3.22 g, 17.3 mmol) were dissolved in THF (100 mL). The reaction mixture was then stirred overnight at room temperature. The precipitate formed was pressure filtered off, and the solvent was evaporated under reduced pressure. The residue was taken up in ethyl acetate (100 mL) and washed with 1% (*w*/*v*) citric acid (3 × 50 mL) and saturated NaCl (50 mL). The organic phase was dried over Na_2_SO_4_, filtered, and the solvent was evaporated under reduced pressure. Yield: 2.63 g (97%); yellow amorphous powder; R_f_(EtOAc:Hex = 1:3) = 0.29; ^1^H NMR (400 MHz, CDCl_3_, 25 °C, TMS): δ = 8.32 (d, *J* = 1.5 Hz, 1H, Ar-*H*_7_), 8.01 (dd, *J*_1_ = 8.5 Hz, *J*_2_ = 1.8 Hz, 1H, Ar-*H*_5_), 7.53 (d, *J* = 8.5 Hz, 1H, Ar-*H*_4_), 4.38 (q, *J* = 7.1 Hz, 2H, COO-C*H*_2_-CH_3_), 3.69–3.63 (m, 4H, 2 × piperazine-C*H*_2_), 3.63–3.56 (m, 4H, 2 × piperazine-C*H*_2_), 1.49 (s, 9H, COC(C*H*_3_)_3_), 1.40 ppm (t, *J* = 7.1 Hz, 3H, COO-CH_2_-C*H*_3_); ^13^C NMR (101 MHz, CDCl_3_, 25 °C, TMS): δ = 170.7, 166.4, 156.3, 154.5, 130.6, 128.0, 123.7, 122.8, 118.5, 80.6 (2C), 60.9, 48.2 (2C), 28.4 (3C), 14.4 ppm; LC–MS (ESI+): *m/z* 392.1 [M+H]^+^ (calcd. *m/z* = 391.2 for C_19_H_25_N_3_O_4_S).

**2-(4-Boc-piperazin-1-yl)benzo[*d*]thiazole-6-carboxylic acid (3):** Compound **2** (2.60 g, 6.64 mmol) was suspended in EtOH (96%, 50 mL) and NaOH_(aq)_ (33.2 mL, 2 M, 66.4 mmol) was added. The mixture was heated to 100 °C and left to stir for 1 h. Afterwards, the reaction mixture was cooled and acidified to pH 3, using 2 M HCl_(aq)_. The precipitate that formed was then filtered off under pressure and left to dry. Yield: 2.25 g (93%); yellow amorphous powder; R_f_(EtOAc:Hex = 1:1) = 0,16; ^1^H NMR (400 MHz, CDCl_3_, 25 °C, TMS): δ = 8.39 (d, *J* = 1.6 Hz, 1H, Ar-*H*_7_), 8.08 (dd, *J*_1_ = 8.5 Hz, *J*_2_ = 1.6 Hz, 1H, Ar-*H*_5_), 7.58 (d, *J* = 8.5 Hz, 1H, Ar-*H*_4_), 3.73–3.66 (m, 4H, 2 × piperazine-C*H*_2_), 3.65–3.58 (m, 4H, 2 × piperazine-C*H*_2_), 1.50 ppm (s, 9H, COC(C*H*_3_)_3_); ^13^C NMR (101 MHz, [D_6_]DMSO, 25 °C, TMS): δ = 170.9, 167.6, 156.5, 154.2, 130.9, 128.1, 123.8, 123.6, 118.4, 79.8 (2C), 48.2 (2C), 28.5 ppm (3C); LC–MS (ESI+): *m/z* 364.1 [M+H]^+^ (calcd. *m/z* = 363.1 for C_17_H_21_N_3_O_4_S).

**General procedure for amide coupling (4a–m)**

The amide coupling reactions were performed under an argon atmosphere. 2-Substituted benzo[*d*]thiazole-6-carboxylic acid (1 equiv) was dissolved in DMF. EDC (1.2 equiv), HOBT (1.3 equiv) and DIPEA (2.5 equiv) were added in an ice bath. After 20 min, the respective amine (1.2 equiv) was added, and the reaction mixture was stirred for 1–3 days at room temperature. When the activated ester that formed with HOBt was too stable to further react with the respective aniline at r.t. (according to liquid chromatography–mass spectrometry analysis), the temperature of the reaction mixture was increased to 70 °C (for **4b–d**, **4g**) and stirred overnight. The solvent was then removed under reduced pressure, and the residue was taken up in ethyl acetate (~100 mL) and washed with 1 M NaOH (3 × 50 mL), 1% (*w*/*v*) citric acid (3 × 50 mL) and with saturated NaCl (50 mL). The organic phase was dried over Na_2_SO_4_, filtered, and the solvent was evaporated under reduced pressure. The crude product was purified with precipitation from a mixture of ethyl acetate and hexane.

**4-(6-(Phenylcarbamoyl)benzo[*d*]thiazol-2-yl)-1-Boc-piperazine (4a)**: Yield: 216 mg (49%); yellow amorphous powder; R_f_(EtOAc:Hex = 2:1) = 0.54; ^1^H NMR (400 MHz, [D_6_]DMSO, 25 °C, TMS): δ = 10.18 (s, 1H, Ar-N*H*-COR), 8.41 (d, *J* = 1.8 Hz, 1H, Ar-*H*_7_), 7.91 (dd, *J*_1_ = 8.5 Hz, *J*_2_ = 1.8 Hz, 1H, Ar-*H*_5_), 7.78 (d, *J* = 7.6 Hz, 2H, 2 × Ar-*H*), 7.55 (d, *J* = 8.5 Hz, 1H, Ar-*H*_4_), 7.35 (t, *J* = 7.9 Hz, 2H, 2 × Ar-*H*), 7.09 (t, *J* = 7.4 Hz, 1H, Ar-*H*), 3.69–3.58 (m, 4H, 2 × piperazine-C*H*_2_), 3.55–3.45 (m, 4H, 2 × piperazine-C*H*_2_), 1.44 ppm (s, 9H, COC(C*H*_3_)_3_); ^13^C (101 MHz, [D_6_]DMSO, 25 °C, TMS): δ = 170.4, 165.5, 155.5, 154.3, 139.9, 130.8, 129.0 (2C), 128.2, 126.6, 123.9, 121.7, 120.7 (2C), 118.3, 79.8 (2C), 48.2 (2C), 28.5 ppm (3C); LC–MS (ESI+): *m/z* 439.1 [M+H]^+^ (calcd. *m/z* = 438.2 for C_23_H_26_N_4_O_3_S).

**4-(6-((3-Chlorophenyl)carbamoyl)benzo[*d*]thiazol-2-yl)-1-Boc-piperazine (4b):** Yield: 67 mg (15%); light pink amorphous powder; R_f_(EtOAc:Hex = 2:1) = 0.61; ^1^H NMR (400 MHz, [D_6_]DMSO, 25 °C, TMS): δ = 10.34 (s, 1H, Ar-N*H*-COR), 8.41 (d, *J* = 1.9 Hz, 1H, Ar-*H*_7_), 7.98 (t, *J* = 2.0 Hz, 1H, Ar-*H*), 7.91 (dd, *J*_1_ = 8.5 Hz, *J*_2_ = 1.9 Hz, 1H, Btz-*H*_5_), 7.71 (ddd, *J*_1_ = 8.2 Hz, *J*_2_ = 2.0 Hz, *J_3_* = 0.9 Hz, 1H, Ar-*H*), 7.56 (d, *J* = 8.5 Hz, 1H, Ar-*H*_4_), 7.38 (t, *J* = 8.2 Hz, 1H, Ar-*H*), 7.15 (ddd, *J*_1_ = 8.2 Hz, *J*_2_ = 2.0 Hz, *J_3_* = 0.9 Hz, 1H, Ar-*H*), 3.65–3.60 (m, 4H, 2 × piperazine-C*H*_2_), 3.53–3.47 (m, 4H, 2 × piperazine-C*H*_2_), 1.44 ppm (s, 9H, COC(C*H*_3_)_3_); LC–MS (ESI+): *m/z* 472.8 [M+H]^+^ (calcd. *m/z* = 471.1 for C_24_H_26_Cl_2_N_4_O_3_S).

**4-(6-((4-Chlorophenyl)carbamoyl)benzo[*d*]thiazol-2-yl)-1-Boc-piperazine (4c):** Yield: 258 mg (48%); off-white amorphous powder; R_f_(EtOAc:Hex = 1:2) = 0.39; ^1^H NMR (400 MHz, [D_6_]DMSO, 25 °C, TMS): δ = 10.31 (s, 1H, Ar-N*H*-COR), 8.40 (d, *J* = 1.8 Hz, 1H, Ar-*H*_7_), 7.91 (dd, *J*_1_ = 8.5, *J*_2_ = 1.8 Hz, 1H, Ar-*H*_5_), 7.82 (d, *J* = 8.9 Hz, 2H, 2 × Ar-*H*), 7.55 (d, *J* = 8.4 Hz, 1H, Ar-*H*_4_), 7.41 (d, *J* = 8.9 Hz, 2H, 2 × Ar-*H*), 3.69–3.58 (m, 4H, 2 × piperazine-C*H*_2_), 3.54–3.46 (m, 4H, 2 × piperazine-C*H*_2_), 1.43 ppm (s, 9H, COC(C*H*_3_)_3_); LC–MS (ESI+): *m/z* 473.1 [M+H]^+^ (calcd. *m/z* = 472.1 for C_23_H_25_ClN_4_O_3_S).

**4-(6-((3,4-Dichlorophenyl)carbamoyl)benzo[*d*]thiazol-2-yl)-1-Boc-piperazine (4d):** Yield: 274 mg (20%); off-white amorphous powder; R_f_(EtOAc:Hex = 1:2) = 0,15; ^1^H NMR (400 MHz, CDCl_3_, 25 °C, TMS): δ = 8.21 (d, *J* = 1.8 Hz, 1H, Ar-*H*_7_), 7.91 (d, *J* = 2.5 Hz, 1H, Ar-*H*_20_), 7.84 (s, 1H, Ar-N*H*-COR), 7.74 (dd, *J*_1_ = 8.5 Hz, *J*_2_ = 1.8 Hz, 1H, Ar-*H*_5_), 7.58 (d, *J* = 8.5 Hz, 1H, Ar-*H*_4_), 7.48 (dd, *J*_1_ = 8.7, *J*_2_ = 2.5 Hz, 1H, Ar-*H_24_*), 7.42 (d, *J* = 8.7 Hz, 1H, Ar-*H*_23_), 3.70–3.64 (m, 6.5, 3.6 Hz, 4H, 2 × piperazine-C*H*_2_), 3.63–3.58 (m, 4H, 2 × piperazine-C*H*_2_), 1.50 ppm (s, 9H, COC(C*H*_3_)_3_); LC–MS (ESI+): *m/z* 506.9 [M+H]^+^ (calcd. *m/z* = 506,1 for C_23_H_24_Cl_2_N_4_O_3_S).

**4-(6-((4-Fluorophenyl)carbamoyl)benzo[*d*]thiazol-2-yl)-1-Boc-piperazine (4e):** Yield: 151 mg (59%); off-white amorphous powder; R_f_(EtOAc:Hex = 2:1) = 0.43; ^1^H NMR (400 MHz, [D_6_]DMSO, 25 °C, TMS): δ = 10.24 (s, 1H, Ar-N*H*-COR), 8.41 (d, *J* = 1.6 Hz, 1H, Ar-*H*_7_), 7.91 (dd, *J*_1_ = 8.5 Hz, *J*_2_ = 1.6 Hz, 1H, Ar-*H*_5_), 7.83–7.76 (m, 2H, 2 × Ar-*H*), 7.55 (d, *J* = 8.5 Hz, 1H, Ar-*H*_4_), 7.20 (t, *J* = 8.9 Hz, 2H, 2 × Ar-*H*), 3.70–3.58 (m, 4H, 2 × piperazine-C*H*_2_), 3.56–3.46 (m, 4H, 2 × piperazine-C*H*_2_), 1.44 ppm (s, 9H, COC(C*H*_3_)_3_); ^19^F NMR (376 MHz, [D_6_]DMSO, 25 °C, TMS): δ = -119.20 ppm; ^13^C (101 MHz, [D_6_]DMSO, 25 °C, TMS): δ = 170.4, 165.4, 158.6 (d, *J* = 239.9 Hz), 155.5, 154.2, 136.2, 136.2, 130.8, 128.0, 126.5, 122.5, 122.5, 121.7, 118.3, 115.7, 115.5, 79.8 (2C), 48.2 (2C), 28.5 ppm (3C); LC–MS (ESI+): *m/z* 457.1 [M+H]^+^ (calcd. *m/z* = 456.2 for C_23_H_25_FN_4_O_3_S).

**4-(6-((4-Bromophenyl)carbamoyl)benzo[*d*]thiazol-2-yl)-1-Boc-piperazine (4f):** Yield: 203 mg (58%); yellow amorphous powder; R_f_(EtOAc:Hex = 2:1) = 0.46; ^1^H NMR (400 MHz, [D_6_]DMSO, 25 °C, TMS): δ = 10.30 (s, 1H, Ar-N*H*-COR), 8.40 (d, *J* = 1.9 Hz, 1H, Ar-*H*_7_), 7.91 (dd, *J*_1_ = 8.5 Hz, *J*_2_ = 1.9 Hz, 1H, Ar-*H*_5_), 7.81–7.73 (m, 2H, 2 × Ar-*H*), 7.58–7.49 (m, 3H, 3 × Ar-*H*), 3.68–3.57 (m, 4H, 2 × piperazine-C*H*_2_), 3.56–3.45 (m, 4H, 2 × piperazine-C*H*_2_), 1.43 ppm (s, 9H, COC(C*H*_3_)_3_); LC–MS (ESI+): *m/z* 571.4 [M+H]^+^ (calcd. *m/z* = 516.1 for C_23_H_25_BrN_4_O_3_S).

**4-(6-((4-Iodophenyl)carbamoyl)benzo[*d*]thiazol-2-yl)-1-Boc-piperazine (4g):** Yield: 77 mg (20%); yellow amorphous powder; R_f_(EtOAc:Hex = 2:1) = 0.58; ^1^H NMR (400 MHz, [D_6_]DMSO, 25 °C, TMS): δ = 10.27 (s, 1H, Ar-N*H*-COR), 8.40 (d, *J* = 1.9 Hz, 1H, Ar-*H*_7_), 7.90 (dd, *J*_1_ = 8.5 Hz, *J*_2_ = 1.9 Hz, 1H, Ar-*H*_5_), 7.72–7.66 (m, 2H, 2 × Ar-*H*), 7.66–7.60 (m, 2H, 2 × Ar-*H*), 7.54 (d, *J* = 8.5 Hz, 1H, Ar-*H*_4_), 3.66–3.58 (m, 4H, 2 × piperazine-C*H*_2_), 3.54–3.45 (m, 4H, 2 × piperazine-C*H*_2_), 1.43 ppm (s, 9H, COC(C*H*_3_)_3_); LC–MS (ESI+): *m/z* 565.0 [M+H]^+^ (calcd. *m/z* = 564.0 for C_23_H_25_IN_4_O_3_S).

**4-(6-((4-Isopropylphenyl)carbamoyl)benzo[*d*]thiazol-2-yl)-1-Boc-piperazine (4h):** Yield: 151 mg (50%); off-white amorphous powder; R_f_(EtOAc:Hex = 2:1) = 0.63; ^1^H NMR (400 MHz, [D_6_]DMSO, 25 °C, TMS): δ = 10.10 (s, 1H, Ar-N*H*-COR), 8.40 (d, *J* = 1.8 Hz, 1H, Ar-*H*_7_), 7.91 (dd, *J*_1_ = 8.5 Hz, *J*_2_ = 1.8 Hz, 1H, Ar-*H*_5_), 7.68 (d, *J* = 8.5 Hz, 2H, 2 × Ar-*H*), 7.54 (d, *J* = 8.5 Hz, 1H, Ar-*H*_4_), 7.21 (d, *J* = 8.5 Hz, 2H, 2 × Ar-*H*), 3.68–3.59 (m, 4H, 2 × piperazine-C*H*_2_), 3.54–3.47 (m, 4H, 2 × piperazine-C*H*_2_), 2.86 (dt, *J*_1_ = 13.7 Hz, *J*_2_ = 6.9 Hz, 1H, Ar-C*H*-(CH_3_)_2_), 1.44 (s, 9H, COC(C*H*_3_)_3_), 1.20 ppm (d, *J* = 6.9 Hz, 6H, Ar-CH-(C*H*_3_)_2_); LC–MS (ESI+): *m/z* 481.1 [M+H]^+^ (calcd. *m/z* = 480.2 for C_26_H_32_N_4_O_3_S).

**4-(6-((4-(Methylhydroxy)phenyl)carbamoyl)benzo[*d*]thiazol-2-yl)-1-Boc-piperazine (4i):** Yield: 333 mg (79%); yellow amorphous powder; R_f_(EtOAc:Hex = 2:1) = 0.58; ^1^H NMR (400 MHz, [D_6_]DMSO, 25 °C, TMS): δ = 10.08 (s, 1H, Ar-N*H*-COR), 8.40 (d, *J* = 1.8 Hz, 1H, Ar-*H*_7_), 7.91 (dd, *J*_1_ = 8.5 Hz, *J*_2_ = 1.8 Hz, 1H, Ar-*H*_5_), 7.73–7.64 (m, 2H, 2 × Ar-*H*), 7.53 (d, *J* = 8.5 Hz, 1H¸, Ar-*H*_4_), 6.98–6.87 (m, 2H, 2 × Ar-*H*), 3.74 (s, 3H, Ar-O-C*H*_3_), 3.66–3.58 (m, 4H, 2 × piperazine-C*H*_2_), 3.54–3.46 (m, 4H, 2 × piperazine-C*H*_2_), 1.44 ppm (s, 9H, COC(C*H*_3_)_3_); LC–MS (ESI+): *m/z* 469.2 [M+H]^+^ (calcd. *m/z* = 468.2 for C_24_H_28_N_4_O_4_S).

**4-(6-(Benzylcarbamoyl)benzo[*d*]thiazol-2-yl)-1-Boc-piperazine (4j):** Yield: 265 mg (71%); yellow amorphous powder; R_f_(EtOAc:Hex = 2:1) = 0.33; ^1^H NMR (400 MHz, [D_6_]DMSO, 25 °C, TMS): δ = 8.96 (t, *J* = 6.0 Hz, 1H, Ar-CH_2_-N*H*-COR), 8.32 (d, *J* = 1.8 Hz, 1H, Ar-*H*_7_), 7.84 (dd, *J*_1_ = 8.5, *J*_2_ = 1.8 Hz, 1H, Ar-*H*_5_), 7.49 (d, *J* = 8.5 Hz, 1H, Ar-*H*_4_), 7.35–7.29 (m, 4H, 4 × Ar-*H*), 7.27–7.21 (m, 1H, Ar-*H*), 4.48 (d, *J* = 6.0 Hz, 2H, Ar-C*H*_2_-NHCO), 3.61 (m, 4H, 2 × piperazine-C*H*_2_), 3.54–3.44 (m, 4H, 2 × piperazine-C*H*_2_), 1.43 ppm (s, 9H, COC(C*H*_3_)_3_); LC–MS (ESI+): *m/z* 452.7 [M+H]^+^ (calcd. *m/z* = 452.2 for C_24_H_26_Cl_2_N_4_O_3_S).

**4-(6-((3-Chlorobenzyl)carbamoyl)benzo[*d*]thiazol-2-yl)1-Boc-piperazine (4k):** Yield: 196 mg (64%); white amorphous powder; R_f_(EtOAc:Hex = 1:1) = 0.19; ^1^H NMR (400 MHz, [D_6_]DMSO, 25 °C, TMS): δ = 9.01 (t, *J* = 6.0 Hz, 1H, CH_2_-N*H*-COR), 8.33 (d, *J* = 1.8 Hz, 1H, Ar-*H*_7_), 7.84 (dd, *J*_1_ = 8.5 Hz, *J*_2_ = 1.8 Hz, 1H, Ar-*H*_5_), 7.50 (d, *J* = 8.5 Hz, 1H, Ar-*H*_4_), 7.39–7.34 (m, 2H, 2 × Ar-*H*), 7.33–7.27 (m, 2H, 2 × Ar-*H*), 4.48 (d, *J* = 6.0 Hz, 2H, RCO-NH-C*H*_2_-Ph), 3.64–3.58 (m, 4H, 2 × piperazine-C*H*_2_), 3.55–3.44 (m, 4H, 2 × piperazine-C*H*_2_), 1,43 ppm (s, 9H, COC(C*H*_3_)_3_); LC–MS (ESI+): *m/z* 487.1 [M+H]^+^ (calcd. *m/z* = 486.1 for C_24_H_27_ClN_4_O_3_S).

**4-(6-((4-Chlorobenzyl)carbamoyl)benzo[*d*]thiazol-2-yl)-1-Boc-piperazine (4l):** Yield: 200 mg (65%); yellow amorphous powder; R_f_(EtOAc:Hex = 1:1) = 0.19; ^1^H NMR (400 MHz, [D_6_]DMSO, 25 °C, TMS): δ = 8.99 (t, *J* = 5.9 Hz, 1H, CH_2_-N*H*-COR), 8.32 (d, *J* = 1.7 Hz, 1H, Ar-*H*_7_), 7.83 (dd, *J*_1_ = 8.5 Hz, *J*_2_ = 1.7 Hz, 1H, Ar-*H*_5_), 7.49 (d, *J* = 8.5 Hz, 1H, Ar-*H*_4_), 7.42–7.31 (m, 4H, 4 × Ar-*H*), 4.46 (d, *J* = 5.9 Hz, 2H, RCO-NH-C*H*_2_-Ph), 3.66–3.56 (m, 4H, 2 × piperazine-C*H*_2_), 3.53–3.43 (m, 4H, 2 × piperazine-C*H*_2_), 1.43 ppm (s, 9H, COC(C*H*_3_)_3_); ^13^C (101 MHz, [D_6_]DMSO, 25 °C, TMS): δ = 170.2, 166.3, 155.2, 154.2, 139.4, 131.7, 130.8, 129.6 (2C), 128.7 (2C), 127.6, 126.0, 121.3, 118.3, 79.8, 48.2 (2C), 42.5 (2C), 28.5 ppm (3C); LC–MS (ESI+): *m/z* 487.4 [M+H]^+^ (calcd. *m/z* = 486.1 for C_24_H_27_ClN_4_O_3_S).

**4-(6-((Indane-1-yl)carbamoyl)benzo[*d*]thiazol-2-yl)-1-Boc-piperazine (4m):** Yield: 165 mg (63%); white amorphous powder; R_f_(EtOAc:Hex = 2:1) = 0.39; ^1^H NMR (400 MHz, [D_6_]DMSO, 25 °C, TMS): δ = 8.68 (d, *J* = 8.3 Hz, 1H, CH-N*H*-COR), 8.35 (d, *J* = 1.6 Hz, 1H, Ar-*H*), 7.87 (dd, *J*_1_ = 8.5 Hz, *J*_2_ = 1.6 Hz, 1H, Ar-*H*), 7.48 (d, *J* = 8.5 Hz, 1H, Ar-*H*), 7.32–7.13 (m, 4H, 4 × Ar-*H*), 5.56 (dd, *J*_1_ = 16.1 Hz, *J*_2_ = 8.3 Hz, 1H, indane-*H*), 3.66–3.55 (m, 1H, indane-*H*), 3.53–3.46 (m, 4H, 2 × piperazine-C*H*_2_), 3.05–2.95 (m, 4H, 2 × piperazine-C*H*_2_), 2.91–2.79 (m, 1H, indane-*H*), 2.05–1.92 ppm (m, 1H, indane-*H*), not visible (1H, indane-*H*); LC–MS (ESI+): *m/z* 479.1 [M+H]^+^ (calcd. *m/z* = 478.2 for C_26_H_30_N_4_O_3_S).

**General procedure for acidolysis used for the synthesis of compounds 5a-m, 9a-j and 14**

4-Boc-protected benzothiazole (1 equiv) was dissolved in dichloromethane. Trifluoroacetic acid (25 equiv) was added, and the reaction mixture was stirred overnight at room temperature. Afterwards, the reaction mixture was diluted with dichloromethane to 25 mL and washed with 1 M NaOH (3 × 25 mL) and saturated NaCl (25 mL). The organic phase was dried over Na_2_SO_4_, filtered, and the solvent was evaporated under reduced pressure.

**4-(6-(Phenylcarbamoyl)benzo[*d*]thiazol-2-yl)piperazine (5a)**: Yield: 91 mg (74%); off-white amorphous powder; R_f_(DCM:MeOH = 9:1) = 0.0; ^1^H NMR (400 MHz, [D_6_]DMSO, 25 °C, TMS): δ = 10.16 (s, 1H, Ar-N*H*-COR), 8.38 (d, *J* = 1.8 Hz, 1H, Ar-*H*_7_), 7.90 (dd, *J*_1_ = 8.5 Hz, *J*_2_ = 1.8 Hz, 1H, Ar-*H*_5_), 7.82–7.75 (m, 2H, 2 × Ar-*H*), 7.51 (d, *J* = 8.5 Hz, 1H, Ar-*H*_4_), 7.39–7.30 (m, 2H, 2 × Ar-*H*), 7.13–7.04 (m, 1H, Ar-*H*), 3.58–3.48 (m, 4H, 2 × piperazine-C*H*_2_), 2.87–2.76 ppm (m, 4H, 2 × piperazine-C*H*_2_); ^13^C (101 MHz, [D_6_]DMSO, 25 °C, TMS): δ = 170.6, 165.5, 155.7, 139.9, 130.6, 129.0 (2C), 127.8, 126.5, 123.9, 121.5, 120.7 (2C), 118.1, 49.9 (2C), 45.5 ppm (2C); HRMS (ESI+) *m*/*z* calcd. calcd. for C_18_H_18_N_4_OS+H^+^: 339.1274 [*M*+H]^+^: found 339.1269; HPLC: t_r_ = 2.27 min (97.4% at 254 nm).

**4-(6-((3-Chlorophenyl)carbamoyl)benzo[*d*]thiazol-2-yl)piperazine (5b):** Yield: 43 mg (91%); off-white amorphous powder; R_f_(DCM:MeOH = 9:1) = 0.07; ^1^H NMR (400 MHz, [D_6_]DMSO, 25 °C, TMS): δ = 10.32 (s, 1H, Ar-N*H*-COR), 8.38 (d, *J* = 1.9 Hz, 1H, Ar-*H*_7_), 7.98 (t, *J* = 2.0 Hz, 1H, Ar-*H*), 7.90 (dd, *J*_1_ = 8.5 Hz, *J*_2_ = 1.9 Hz, 1H, Ar-*H*_5_), 7.71 (ddd, *J*_1_ = 8.1 Hz, *J*_2_ = 2.0 Hz, *J_3_* = 0.9 Hz, 1H, Ar-*H*), 7.52 (d, *J* = 8.5 Hz, 1H, Ar-*H*_4_), 7.38 (t, *J* = 8.1 Hz, 1H, Ar-*H*), 7.14 (ddd, *J*_1_ = 8.1 Hz, *J*_2_ = 2.0 Hz, *J_3_* = 0.9 Hz, 1H, Ar-*H*), 3.58–3.51 (m, 4H, 2 × piperazine-C*H*_2_), 2.85–2.79 ppm (m, 4H, 2 × piperazine-C*H*_2_), not visible (N*H*); ^13^C (101 MHz, [D_6_]DMSO, 25 °C, TMS): δ = 170.7, 165.8, 155.9, 141.4, 133.4, 130.8, 130.6, 127.3, 126.6, 123.5, 121.7, 120.0, 119.0, 118.1, 49.9 (2C), 45.5 (2C) ppm; HRMS (ESI+) *m*/*z* calcd. calcd. for C_18_H_17_ClN_4_OS+H^+^: 373.0884 [*M*+H]^+^: found 373.0880; HPLC: t_r_ = 5.31 min (95.5% at 254 nm).

**4-(6-((4-Chlorophenyl)carbamoyl)benzo[*d*]thiazol-2-yl)piperazine (5c):** Yield: 61 mg (31%); white powder; R_f_(DCM:MeOH = 9:1) = 0.09; ^1^H NMR (400 MHz, [D_6_]DMSO, 25 °C, TMS): δ = 10.29 (s, 1H, Ar-N*H*-COR), 8.38 (d, *J* = 1.8 Hz, 1H, Ar-*H*_7_), 7.89 (dd, *J*_1_ = 8.5, *J*_2_ = 1.8 Hz, 1H, Ar-*H*_5_), 7.85–7.78 (m, 2H, 2 × Ar-*H*), 7.51 (d, *J* = 8.5 Hz, 1H, Ar-*H*_4_), 7.44–7.37 (m, 2H, 2 × Ar-*H*), 3.57–3.50 (m, 4H, 2 × piperazine-C*H*_2_), 2.84–2.78 ppm (m, 4H, 2 × piperazine-C*H*_2_), not visible (N*H*); ^13^C (101 MHz, [D_6_]DMSO, 25 °C, TMS): δ = 170.7, 165.6, 155.8, 138.9, 130.6, 129.0 (2C), 127.5, 127.4, 126.6, 122.2 (2C), 121.6, 118.1, 49.9 (2C), 45.5 ppm (2C); HRMS (ESI+) *m*/*z* calcd. calcd. for C_18_H_17_ClN_4_OS+H^+^: 373.0884 [*M*+H]^+^: found 373.0878; HPLC: t_r_ = 5.27 min (97.2% at 254 nm).

**4-(6-((3,4-Dichlorophenyl)carbamoyl)benzo[*d*]thiazol-2-yl)piperazine (5d):** Yield: 118 mg (68%); off-white amorphous powder; R_f_(EtOAc:Hex = 1:1) = 0; ^1^H NMR (400 MHz, CDCl_3_, 25 °C, TMS): δ = 8.20 (d, *J* = 1.9 Hz, 1H, Ar-*H*_7_), 7.90 (d, *J* = 2.4 Hz, 1H, Ar-*H*_20_), 7.79 (s, 1H, Ar-N*H*-COR), 7.73 (dd, *J*_1_ = 8.5 Hz, *J*_2_ = 1.9 Hz, 1H, Ar-*H*_5_), 7.57 (d, *J* = 8.5 Hz, 1H, Ar-*H*_4_), 7.48 (dd, *J*_1_ = 8.7 Hz, *J*_2_ = 2.4 Hz, 1H, Ar-*H_24_*), 7.42 (d, *J* = 8.7 Hz, 1H, Ar-*H*_23_), 3.70–3.65 (m, 4H, 2 × piperazine-C*H*_2_), 3.06–2.99 ppm (m, 4H, 2 × piperazine-C*H*_2_), not visible (N*H*); ^13^C NMR (101 MHz, [D_6_]DMSO, 25 °C, TMS): δ = 170.8, 165.8, 156.0, 140.1, 131.3, 131.0, 130.7, 127.1, 126.7, 125.2, 121.7, 121.7, 120.6, 118.1, 49.9 (2C), 45.5 ppm (2C); HRMS (ESI+) *m*/*z* calcd. calcd. for C_18_H_16_Cl_2_N_4_OS+H^+^: 407.0495 [*M*+H]^+^: found 407.0487; HPLC: t_r_ = 6.57 min (95.3% at 254 nm).

**4-(6-((4-Fluorophenyl)carbamoyl)benzo[*d*]thiazol-2-yl)piperazine (5e):** Yield: 74 mg (95%); white amorphous powder; R_f_(DCM:MeOH = 9:1) = 0.05; ^1^H NMR (400 MHz, [D_6_]DMSO, 25 °C, TMS): δ = 10.22 (s, 1H, Ar-N*H*-COR), 8.38 (d, *J* = 1.8 Hz, 1H, Ar-*H*_7_), 7.90 (dd, *J*_1_ = 8.5 Hz, *J*_2_ = 1.8 Hz, 1H, Ar-*H*_5_), 7.84–7.74 (m, 2H, 2 × Ar-*H*), 7.52 (d, *J* = 8.5 Hz, 1H, Ar-*H*_4_), 7.24–7.14 (m, 2H, 2 × Ar-*H*), 3.59–3.50 (m, 4H, 2 × piperazine-C*H*_2_), 2.83 ppm (m, 4.3 Hz, 4H, 2 × piperazine-C*H*_2_), not visible (N*H*); ^19^F NMR (376 MHz, [D_6_]DMSO, 25 °C, TMS): δ = -119.26 ppm; ^13^C (101 MHz, [D_6_]DMSO, 25 °C, TMS): δ = 170.6, 165.4, 158.60 (d, *J* = 239.8 Hz), 155.7, 136.2 (d, *J* = 2.6 Hz), 130.6, 127.6, 126.5, 122.5, 122.4, 121.5, 118.1, 115.7, 115.5, 49.9 (2C), 45.5 ppm (2C); HRMS (ESI+) *m*/*z* calcd. calcd. for C_18_H_17_FN_4_OS+H^+^: 357.1180 [*M*+H]^+^: found 357.1173; HPLC: t_r_ = 3.27 min (99.0% at 254 nm).

**4-(6-((4-Bromophenyl)carbamoyl)benzo[*d*]thiazol-2-yl)piperazine (5f):** Yield: 70 mg (72%); off-white amorphous powder; R_f_(DCM:MeOH = 9:1) = 0.05; ^1^H NMR (400 MHz, [D_6_]DMSO, 25 °C, TMS): δ = 10.28 (s, 1H, Ar-N*H*-COR), 8.38 (d, *J* = 1.8 Hz, 1H, Ar-*H*_7_), 7.89 (dd, *J*_1_ = 8.5 Hz, *J*_2_ = 1.8 Hz, 1H, Ar-*H*_5_), 7.79–7.73 (m, 2H, 2 × Ar-*H*), 7.56–7.47 (m, 3H, 3 × Ar-*H*), 3.59–3.48 (m, 4H, 2 × piperazine-C*H*_2_), 2.86–2.77 ppm (m, 4H, 2 × piperazine-C*H*_2_), not visible (N*H*); ^13^C (101 MHz, [D_6_]DMSO, 25 °C, TMS): δ = 170.7, 165.6, 155.8, 139.3, 131.9 (2C), 130.6, 127.5, 126.6, 122.6 (2C), 121.6, 118.1, 115.5, 49.9 (2C), 45.5 ppm (2C); HRMS (ESI+) *m*/*z* calcd. calcd. for C_18_H_17_BrN_4_OS+H^+^: 417.0379 [*M*+H]^+^: found 417.0372; HPLC: t_r_ = 5.45 min (96.5% at 254 nm).

**4-(6-((4-Iodophenyl)carbamoyl)benzo[*d*]thiazol-2-yl)piperazine (5g):** Yield: 44 mg (83%); light yellow amorphous powder; R_f_(DCM:MeOH = 9:1) = 0.09; ^1^H NMR (400 MHz, [D_6_]DMSO, 25 °C, TMS): δ = 10.25 (s, 1H, Ar-N*H*-COR), 8.37 (s, 1H, Ar-*H*), 7.89 (d, *J* = 8.5 Hz, 1H, Ar-*H*), 7.71–7.60 (m, 4H, 4 × Ar-*H*), 7.50 (d, *J* = 8.1 Hz, 1H, Ar-*H*), 3.57–3.51 (m, 4H, 2 × piperazine-C*H*_2_), 2.84–2.79 ppm (m, 4H, 2 × piperazine-C*H*_2_), not visible (N*H*); ^13^C (101 MHz, [D_6_]DMSO, 25 °C, TMS): δ = 170.6, 165.6, 155.8, 139.8, 137.7 (2C), 130.6, 127.5, 126.6, 122.8 (2C), 121.6, 118.1, 87.4, 49.8 (2C), 45.5 ppm (2C); HRMS (ESI+) *m*/*z* calcd. for C_18_H_17_IN_4_OS+H^+^: 465.0234 [*M*+H]^+^: found 465.0241; HPLC: t_r_ = 5.03 min (98.7% at 254 nm).

**4-(6-((4-Isopropylphenyl)carbamoyl)benzo[*d*]thiazol-2-yl)piperazine (5h):** Yield: 44 mg (80%); off-white amorphous powder; R_f_(DCM:MeOH = 9:1) = 0.06; ^1^H NMR (400 MHz, [D_6_]DMSO, 25 °C, TMS): δ = 10.08 (s, 1H, Ar-N*H*-COR), 8.37 (d, *J* = 1.8 Hz, 1H, Ar-*H*_7_), 7.89 (dd, *J*_1_ = 8.5 Hz, *J*_2_ = 1.8 Hz, 1H, Ar-*H*_5_), 7.68 (d, *J* = 8.6 Hz, 2H, 2 × Ar-*H*), 7.50 (d, *J* = 8.5 Hz, 1H, Ar-*H*_4_), 7.21 (d, *J* = 8.6 Hz, 2H, 2 × Ar-*H*), 3.58–3.47 (m, 4H, 2 × piperazine-C*H*_2_), 2.90–2.78 (m, 5H, Ar-C*H*-(CH_3_)_2_ + 2 × piperazine-C*H*_2_), 1.20 ppm (d, *J* = 6.9 Hz, 6H, Ar-CH-(C*H*_3_)_2_); ^13^C (101 MHz, [D_6_]DMSO, 25 °C, TMS): δ = 170.5, 165.3, 155.6, 143.9, 137.6, 130.5, 127.9, 126.7 (2C), 126.5, 121.5, 120.8 (2C), 118.0, 49.9 (2C), 45.5 (2C), 33.4, 24.5 ppm (2C); HRMS (ESI+) *m*/*z* calcd. for C_21_H_24_N_4_OS+H^+^: 381.1744 [*M*+H]^+^: found 381.1737; HPLC: t_r_ = 5.12 min (99.0% at 254 nm).

**4-(6-((4-(Methylhydroxy)phenyl)carbamoyl)benzo[*d*]thiazol-2-yl)piperazine (5i):** Yield: 43 mg (91%); off-white amorphous powder; R_f_(DCM:MeOH = 9:1) = 0.08; ^1^H NMR (400 MHz, [D_6_]DMSO, 25 °C, TMS): δ = 10.04 (s, 1H, Ar-N*H*-COR), 8.36 (d, *J* = 1.8 Hz, 1H, Ar-*H*_7_), 7.89 (dd, *J*_1_ = 8.5 Hz, *J*_2_ = 1.8 Hz, 1H, Ar-*H*_5_), 7.71–7.63 (m, 2H, 2 × Ar-*H*), 7.50 (d, *J* = 8.5 Hz, 1H, Ar-*H*_4_), 6.95–6.89 (m, 2H, 2 × Ar-*H*), 3.74 (s, 3H, Ar-O-C*H*_3_), 3.58–3.48 (m, 4H, 2 × piperazine-C*H*_2_), 2.85–2.78 ppm (m, 4H, 2 × piperazine-C*H*_2_), not visible (N*H*); ^13^C (101 MHz, [D_6_]DMSO, 25 °C, TMS): δ = 170.5, 165.1, 155.9, 155.6, 132.9, 130.6, 128.0, 125.9, 122.3 (2C), 121.4, 118.0, 114.2 (2C), 55.6, 49.9 (2C), 45.5 ppm (2C); HRMS (ESI+) *m*/*z* calcd. for C_19_H_20_N_4_O_2_S+H^+^: 369.1380 [*M*+H]^+^: found 369.1374; HPLC: t_r_ = 3.42 min (95.0% at 254 nm).

**4-(6-((Benzyl)carbamoyl)benzo[*d*]thiazol-2-yl)piperazine (5j):** Yield: 96 mg (81%); light pink amorphous powder; R_f_(DCM:MeOH = 9:1) = 0.13; ^1^H NMR (400 MHz, [D_6_]DMSO, 25 °C, TMS): δ = 8.95 (t, *J* = 6.0 Hz, 1H, Ar-CH_2_-N*H*-COR), 8.30 (d, *J* = 1.8 Hz, 1H, Ar-*H*_7_), 7.83 (dd, *J*_1_ = 8.5 Hz, *J*_2_ = 1.8 Hz, 1H, Ar-*H*_5_), 7.45 (d, *J* = 8.5 Hz, 1H, Ar-*H*_4_), 7.35–7.29 (m, 4H, 4 × Ar-*H*), 7.26–7.20 (m, 1H, Ar-*H*), 4.48 (d, *J* = 6.0 Hz, 2H, Ar-C*H*_2_-NHCO), 3.56–3.48 (m, 4H, 2 × piperazine-C*H*_2_), 2.84–2.76 ppm (m, 4H, 2 × piperazine-C*H*_2_), not visible (N*H*); ^13^C (101 MHz, [D_6_]DMSO, 25 °C, TMS): δ = 170.3, 166.3, 155.3, 140.3, 130.6, 128.7 (2C), 127.7 (2C), 127.6, 127.2, 126.0, 121.1, 118.1, 49.3 (2C), 45.1 (2C), 43.1 ppm; HRMS (ESI+) *m*/*z* calcd. for C_19_H_20_N_4_OS+H^+^: 353.1430 [*M*+H]^+^: found 353.1424; HPLC: t_r_ = 3.43 min (95.3% at 254 nm).

**4-(6-((3-Chlorobenzyl)carbamoyl)benzo[*d*]thiazol-2-yl)piperazine (5k):** Yield: 48 mg (74%); white amorphous powder; R_f_(DCM:MeOH = 9:1) = 0.06; ^1^H NMR (400 MHz, [D_6_]DMSO, 25 °C, TMS): δ = 8.99 (t, *J* = 6.0 Hz, 1H, CH_2_-N*H*-COR), 8.30 (d, *J* = 1.9 Hz, 1H, Ar-*H*_7_), 7.82 (dd, *J*_1_ = 8.5 Hz, *J*_2_ = 1.9 Hz, 1H, Ar-*H*_5_), 7.46 (d, *J* = 8.5 Hz, 1H, Ar-*H*_4_), 7.39–7.33 (m, 2H, 2 × Ar-*H*), 7.33–7.27 (m, 2H, 2 × Ar-*H*), 4.47 (d, *J* = 6.0 Hz, 2H), 3.56–3.48 (m, 4H, 2 × piperazine-C*H*_2_), 2.85–2.76 ppm (m, 4H, 2 × piperazine-C*H*_2_), not visible (N*H*); ^13^C (101 MHz, [D_6_]DMSO, 25 °C, TMS): δ = 170.4, 166.4, 155.5, 142.9, 133.4, 130.6, 130.6, 127.5, 127.2, 127.1, 126.4, 126.0, 121.1, 118.1, 49.8 (2C), 45.5 (2C), 42.7 ppm; HRMS (ESI+) *m*/*z* calcd. for C_19_H_19_ClN_4_OS+H^+^: 387.1041 [*M*+H]^+^: found 387.1032; HPLC: t_r_ = 4.26 min (98.0% at 254 nm).

**4-(6-((4-Chlorobenzyl)carbamoyl)benzo[*d*]thiazol-2-yl)piperazine (5l):** Yield: 59 mg (92%); off-white amorphous powder; R_f_(DCM:MeOH = 9:1) = 0.07; ^1^H NMR (400 MHz, [D_6_]DMSO, 25 °C, TMS): δ = 8.98 (t, *J* = 6.0 Hz, 1H, t, *J* = 5.9 Hz, 1H, CH_2_-N*H*-COR), 8.29 (d, *J* = 1.8 Hz, 1H, Ar-*H*_7_), 7.82 (dd, *J*_1_ = 8.5 Hz, *J*_2_ = 1.8 Hz, 1H, Ar-*H*_5_), 7.46 (d, *J* = 8.5 Hz, 1H, Ar-*H*_4_), 7.41–7.30 (m, 4H, 4 × Ar-*H*), 4.46 (d, *J* = 6.0 Hz, 2H, RCO-NH-C*H*_2_-Ph), 3.57–3.46 (m, 4H, 2 × piperazine-C*H*_2_), 2.86–2.77 (m, 4H, 2 × piperazine-C*H*_2_), 2.77–2.69 ppm (m, 1H, N*H*); ^13^C (101 MHz, [D_6_]DMSO, 25 °C, TMS): δ = 170.4, 166.3, 155.4, 139.4, 131.7, 130.6, 129.6 (2C), 128.7 (2C), 127.3, 126.0, 121.1, 118.0, 49.7 (2C), 45.4 (2C), 42.5 ppm; HRMS (ESI+) *m*/*z* calcd. for C_19_H_19_ClN_4_OS+H^+^: 387.1041 [*M*+H]^+^: found 387.1034; HPLC: t_r_ = 4.15 min (97.7% at 254 nm).

**4-(6-((Indane-1-yl)carbamoyl)benzo[*d*]thiazol-2-yl)piperazine (5m):** Yield: 52 mg (83%); white amorphous powder; R_f_(DCM:MeOH = 9:1) = 0.07; ^1^H NMR (400 MHz, [D_6_]DMSO, 25 °C, TMS): δ = 8.66 (d, *J* = 8.3 Hz, 1H, CH-N*H*-CO), 8.32 (d, *J* = 1.7 Hz, 1H, Ar-*H*_7_), 7.86 (dd, *J*_1_ = 8.5 Hz, *J*_2_ = 1.7 Hz, 1H, Ar-*H*_5_), 7.44 (d, *J* = 8.5 Hz, 1H, Ar-*H*_4_), 7.29–7.14 (m, 4H, 4 × Ar-*H*), 5.56 (q, *J* = 8.3 Hz, 1H, indane-*H*), 3.55–3.48 (m, 4H, 2 × piperazine-C*H*_2_), 3.00 (ddd, *J*_1_ = 15.6 Hz, *J*_2_ = 8.8 Hz, *J_3_* = 2.9 Hz, 1H, indane-*H*), 2.90–2.77 (m, 5H, 2 × piperazine-C*H_2_,* and indane-*H*), 2.47–2.40 (m, 1H), 2.05–1.92 ppm (m, 1H, indane-*H*), not visible (N*H*); ^13^C (101 MHz, [D_6_]DMSO, 25 °C, TMS): δ = 170.3, 166.2, 155.3, 144.8, 143.4, 130.4, 127.8, 127.5, 126.8, 126.2, 124.9, 124.5, 121.2, 118.0, 54.7, 49.8 (2C), 45.5 (2C), 33.2, 30.3 ppm; HRMS (ESI+) *m*/*z* calcd. for C_21_H_22_N_4_OS+H^+^: 379.1587 [*M*+H]^+^: found 379.1579; HPLC: t_r_ = 4.22 min (96.6% at 254 nm).

**2-Amino-*N*-(4-chlorophenyl)benzo[*d*]thiazole-6-carboxamide (6)**: The reaction was performed under an argon atmosphere. 2-Aminobenzo[*d*]thiazole-6-carboxylic acid (2.13 g, 11.0 mmol) was dissolved in DMF (40 mL). EDC (2.04 g, 13.2 mmol), HOBt (1.93 g, 14.3 mmol) and *N-*methylmorpholine (2.86 mL, 27.4 mmol) were added in an ice bath. After 20 min, 4-chloroaniline (1.82 g, 14.3 mmol) was added to the reaction mixture, which was then left to stir for 2 days at room temperature. The solvent was removed under reduced pressure, and the residue was taken up in ethyl acetate (200 mL) and washed with 1 M NaOH (3 × 75 mL), 1% (*w*/*v*) citric acid (3 × 75 mL) and with saturated NaCl (100 mL). The organic phase was dried over Na_2_SO_4_, filtered, and the solvent was evaporated under reduced pressure. The product was purified with precipitation from a mixture of ethyl acetate and hexane. Yield: 1.82 g (55%); off-white amorphous powder; R_f_(DCM:MeOH = 9:1) = 0.14; ^1^H NMR (400 MHz, [D_6_]DMSO, 25 °C, TMS): δ = 10.25 (s, 1H, Ar-N*H*-COR) 8.29 (d, *J* = 1.7 Hz, 1H, Ar-N*H*-COR), 7.87–7.80 (m, 5H, 5 × Ar-*H*), 7.45–7.37 ppm (m, 3H, Ar-*H* and N*H*_2_); ^13^C (101 MHz, [D_6_]DMSO, 25 °C, TMS): δ = 169.3, 165.7, 156.2, 138.9, 131.4, 129.0 (2C), 127.4; 127.4, 126.2, 122.1 (2C), 121.3, 117.4 ppm; LC–MS (ESI+): *m/z* 345.1 [M+H+CH_3_CN]^+^ (calcd. *m/z* = 303.0 for C_14_H_10_ClN_3_OS).

**2-Bromo-*N*-(4-chlorophenyl)benzo[*d*]thiazole-6-carboxamide (7)**: Compound **6** (1.80 g, 5.93 mmol) and CuBr_2_ (2.65 g, 11.9 mmol) were dissolved in acetonitrile (90 mL). *tert*-Butyl nitrite (1.41 mL, 11.9 mmol, 867 mg/mL) was added in an ice bath, and the reaction mixture was stirred overnight at room temperature. The solvent was then removed under reduced pressure, and the residue was taken up in ethyl acetate (150 mL) and saturated NH_4_Cl (50 mL). The precipitate that formed was filtered off under pressure. The organic phase was additionally washed with saturated NH_4_Cl (3 × 75 mL) and saturated NaCl. The organic phase was dried over Na_2_SO_4_, filtered, and the solvent was evaporated under reduced pressure. Yield: 1.80 g (83%); orange amorphous powder; R_f_(EtOAc:Hex = 1:2) = 0.34; ^1^H NMR (400 MHz, [D_6_]DMSO, 25 °C, TMS): δ = 10.58 (s, 1H, Ar-N*H*-COR), 8.73–8.70 (m, 1H, Ar-*H*), 8.16–8.12 (m, 1H, Ar-*H*), 8.09 (m, 1H, Ar-*H*), 7.87–7.80 (m, 2H, 2 × Ar-*H*), 7.48–7.40 ppm (m, 2H, 2 × Ar-*H*); ^13^C (101 MHz, [D_6_]DMSO, 25 °C, TMS): δ = 165.4, 154.2, 143.2, 138.5, 137.4, 132.6, 129.1 (2C), 127.9, 126.8, 122.7, 122.5, 122.3 ppm (2C); LC–MS (ESI-): *m/z* 365.0 [M-H]^-^ (calcd. *m/z* = 365.9 for C_14_H_8_BrClN_2_OS).

**General procedure for the synthesis of compounds 8a-n**

The reaction was performed under an argon atmosphere. Compound **7** (1 equiv) was dissolved in THF, and triethylamine was added (2.5 equiv). The corresponding amine (1.25 equiv) was added to the reaction mixture, and the solution was stirred overnight at room temperature. The precipitate formed was filtered off. The solvent was then evaporated under reduced pressure, and the residue was dissolved in ethyl acetate (50–75 mL) and 1% (*w*/*v*) citric acid (30 mL). The organic phase was additionally washed with 1% (*w*/*v*) citric acid (2 × 30 mL) and saturated NaCl (30 mL). The organic phase was then dried over Na_2_SO_4_, filtered, and the solvent was evaporated under reduced pressure. The products were purified using column chromatography.

**3-*N*-Boc-1-*N*-(6-((4-Chlorophenyl)carbamoyl)benzo[*d*]thiazol-2-yl)1,3-diaminopropane (8a):** The product was purified using column chromatography (mobile phase: DCM:MeOH = 25:1). Yield: 159 mg (42%); white amorphous powder; R_f_(DCM:MeOH = 20:1) = 0.19; ^1^H NMR (400 MHz, [D_6_]DMSO, 25 °C, TMS): δ = 10.26 (s, 1H, Ar-N*H*-COR), 8.34 (t, *J* = 5.3 Hz, 1H, Ar-N*H*-CH_2_), 8.29 (d, *J* = 1.7 Hz, 1H, Ar-*H*), 7.89–7.79 (m, 3H, 3 × Ar-*H*), 7.46 (d, *J* = 8.4 Hz, 1H, Ar-*H*), 7.43–7.38 (m, 2H, 2 × Ar-*H*), 6.91 (t, *J* = 5.3 Hz, 1H, CO-N*H*-CH_2_), 3.40 (dd, *J*_1_ = 12.6 Hz, *J*_2_ = 6.7 Hz, 2H, C*H*_2_), 3.02 (dd, *J*_1_ = 12.6 Hz, *J*_2_ = 6.7 Hz, 2H, C*H*_2_), 1.72 (p, *J* = 6.7 Hz, 2H, C*H*_2_), 1.38 ppm (s, 9H, COC(C*H*_3_)_3_); LC–MS (ESI+): *m/z* 461.2 [M+H]^+^ (calcd. *m/z* = 460.1 for C_22_H_25_N_4_O_3_S).

**3-(*N*-Boc-Amino)-1-(6-((4-chlorophenyl)carbamoyl)benzo[*d*]thiazol-2-yl)piperidine (8b):** The product was purified using column chromatography (mobile phase: DCM:MeOH = 40:1). Yield: 108 mg (33%); light orange amorphous powder; R_f_(DCM:MeOH = 30:1) = 0.08; ^1^H NMR (400 MHz, [D_6_]DMSO, 25 °C, TMS): δ = 10.28 (s, 1H, Ar-N*H*-COR), 8.36 (d, *J* = 1.8 Hz, 1H, Ar-*H*_7_), 7.89 (dd, *J*_1_ = 8.5 Hz, *J*_2_ = 1.8 Hz, 1H, Ar-*H*_5_), 7.86–7.78 (m, 2H, 2 × Ar-*H*), 7.51 (d, *J* = 8.5 Hz, 1H, Ar-*H*_4_), 7.43–7.37 (m, 2H, 2 × Ar-*H*), 7.08 (d, *J* = 7.1 Hz, 1H, CH-N*H*-Boc), 4.05–3.97 (m, 1H, piperidine-*H*), 3.84–3.74 (m, 1H, piperidine-*H*), 3.53–3.42 (m, 1H, piperidine-*H*), 3.11 (dd, *J*_1_ = 12.6 Hz, *J*_2_ = 9.1 Hz, 1H, piperidine-*H*), 1.92–1.78 (m, 2H, 2 × piperidine-*H*), 1.63–1.32 ppm (m, 11H, 2 × piperidine-*H* and COC(C*H*_3_)_3_); LC–MS (ESI+): *m/z* 487.4 [M+H]^+^ (calcd. *m/z* = 486.1 for C_24_H_27_ClN_4_O_3_S).

**3-(*S*)-(*N*-Boc-Amino)-1-(6-((4-chlorophenyl)carbamoyl)benzo[*d*]thiazol-2-yl)pyrrolidine (8c):** The product was purified using column chromatography (mobile phase: DCM:MeOH = 40:1). Yield: 179 mg (56%); white amorphous powder; R_f_(DCM:MeOH = 9:1) = 0.32; ^1^H NMR (400 MHz, [D_6_]DMSO, 25 °C, TMS): δ = 10.28 (s, 1H, Ar-N*H*-COR), 8.38 (d, *J* = 1.9 Hz, 1H, Ar-*H*_7_), 7.89 (dd, *J*_1_ = 8.5 Hz, *J*_2_ = 1.9 Hz, 1H, Ar-*H*_5_), 7.85–7.79 (m, 2H, 2 × Ar-*H*), 7.54 (d, *J* = 8.5 Hz, 1H, Ar-*H*_4_), 7.44–7.38 (m, 2H, 2 × Ar-*H*), 7.37–7.33 (m, 1H, CH-N*H*-Boc), 4.24–4.14 (m, 1H, pyrrolidine-*H*), 3.79–3.51 (m, 3H, 3 × pyrrolidine-*H*), 3.43–3.36 (m, 1H, pyrrolidine-*H*), 2.28–2.17 (m, 1H, pyrrolidine-*H*), 2.02–1.91 (m, 1H, pyrrolidine-*H*), 1.40 ppm (s, 9H, COC(C*H*_3_)_3_); [α]_D_^25^ = 9.5 (c = 0.86 in DMF); LC–MS (ESI+): *m/z* 473.3 [M+H]^+^ (calcd. *m/z* = 472.1 for C_23_H_25_ClN_4_O_3_S).

**3-(*R*)-(*N*-Boc-Amino)-1-(6-((4-chlorophenyl)carbamoyl)benzo[*d*]thiazol-2-yl)pyrrolidine (8d):** The product was purified using column chromatography (mobile phase: DCM:MeOH = 30:1). Yield: 130 mg (67%); white amorphous powder; R_f_(DCM:MeOH = 9:1) = 0.50; ^1^H NMR (400 MHz, [D_6_]DMSO, 25 °C, TMS): δ = 10.28 (s, 1H, Ar-N*H*-COR), 8.38 (d, *J* = 1.9 Hz, 1H, Ar-*H*_7_), 7.89 (dd, *J*_1_ = 8.5 Hz, *J*_2_ = 1.9 Hz, 1H, Ar-*H*_5_), 7.85–7.79 (m, 2H, 2 × Ar-*H*), 7.54 (d, *J* = 8.5 Hz, 1H, Ar-*H*_4_), 7.45–7.38 (m, 2H, 2 × Ar-*H*), 7.35 (d, *J* = 6.3 Hz, 1H, CH-N*H*-Boc), 4.22–4.15 (m, 1H, pyrrolidine-*H*), 3.78–3.51 (m, 3H, 3 × pyrrolidine-*H*), 3.45–3.36 (m, 1H, pyrrolidine-*H*), 2.28–2.17 (m, 1H, pyrrolidine-*H*), 2.01–1.92 (m, 1H, pyrrolidine-*H*), 1.40 ppm (s, 9H, COC(C*H*_3_)_3_); [α]_D_^25^ = − 9.5 (c = 0,69 in DMF); LC–MS (ESI+): *m/z* 473.0 [M+H]^+^ (calcd. *m/z* = 472.1 for C_23_H_25_ClN_4_O_3_S).

**4-(*N*-Boc-Aminomethyl)-1-(6-((4-chlorophenyl)carbamoyl)benzo[*d*]thiazol-2-yl)piperidine (8e):** The product was purified using column chromatography (mobile phase: EtOAc:Hex = 1:1). Yield: 67 mg (20%); white amorphous powder; R_f_(EtOAc:Hex = 2:1) = 0.34; ^1^H NMR (400 MHz, [D_6_]DMSO, 25 °C, TMS): δ = 10.28 (s, 1H, Ar-N*H*-COR), 8.37 (d, *J* = 1.9 Hz, 1H, Ar-*H*_7_), 7.89 (dd, *J*_1_ = 8.5 Hz, *J*_2_ = 1.9 Hz, 1H, Ar-*H*_5_), 7.84–7.79 (m, 2H, 2 × Ar-*H*), 7.50 (d, *J* = 8.5 Hz, 1H, Ar-*H*_4_), 7.43–7.37 (m, 2H, 2 × Ar-*H*), 6.96 (t, *J* = 5.8 Hz, 1H, CH_2_-N*H*-Boc), 4.15–4.00 (m, 2H, 2 × methylpiperdine-*H*), 3.24–3.13 (m, 2H, 2 × methylpiperdine-*H*), 2.86 (t, *J* = 6.2 Hz, 2H, 2 × methylpiperdine-*H*), 1.80–1.64 (m, 3H, 3 × methylpiperdine-*H*), 1.38 (s, 9H, COC(C*H*_3_)_3_), 1.27–1.11 ppm (m, 2H, 2 × methylpiperdine-*H*); LC–MS (ESI): *m/z* 501.4 [M+H]^+^ (calcd. *m/z* = 500.2 for C_25_H_29_ClN_4_O_3_S).

**1-Boc-4-((6-((4-Chlorophenyl)carbamoyl)benzo[*d*]thiazol-2-yl)amino)piperidine (8f):** The product was purified using column chromatography (mobile phase: DCM:MeOH = 20:1). Yield: 49 mg (15%); white amorphous powder; R_f_(DCM:MeOH = 20:1) = 0.21; ^1^H NMR (400 MHz, [D_6_]DMSO, 25 °C, TMS): δ = 10.26 (s, 1H, Ar-N*H*-COR), 8.38 (d, *J* = 7.4 Hz, 1H, Ar-N*H*-CH), 8.29 (d, *J* = 1.8 Hz, 1H, Ar-*H*), 7.88–7.78 (m, 3H, 3 × Ar-*H*), 7.47 (d, *J* = 8.5 Hz, 1H, Ar-*H*), 7.44–7.36 (m, 2H, 2 × Ar-*H*), 4.02–3.81 (m, 3H, 3 × piperidine-*H*), 3.05–2.88 (m, 2H, 2 × piperidine-*H*), 2.01–1.93 (m, 2H, 2 × piperidine-*H*), 1.45–1.30 ppm (m, 11H, COC(C*H*_3_)*_3_*+ 2 × piperidine-*H*); LC–MS (ESI+): *m/z* 487.1 [M+H]^+^ (calcd. *m/z* = 486.1 for C_24_H_27_ClN_4_O_3_S).

**2-Boc-7-(6-((4-Chlorophenyl)carbamoyl)benzo[*d*]thiazol-2-yl)-2,7-diazaspiro[4.4]nonane (8g):** The product was purified using column chromatography (mobile phase: DCM:MeOH = 20:1). Yield: 141 mg (51%); off-white amorphous powder; R_f_(DCM:MeOH = 9:1) = 0.44; ^1^H NMR (400 MHz, [D_6_]DMSO, 25 °C, TMS): δ = 10.28 (s, 1H, Ar-N*H*-COR), 8.39 (d, *J* = 1.9 Hz, 1H, Ar-*H*_7_), 7.90 (dd, *J*_1_ = 8.5 Hz, *J*_2_ = 1.9 Hz, 1H, Ar-*H*_5_), 7.86–7.79 (m, 2H, 2 × Ar-*H*), 7.53 (d, *J* = 8.5 Hz, 1H, Ar-*H*_4_), 7.44–7.36 (m, 2H, 2 × Ar-*H*), 3.65 (s, 2H, C*H*_2_), 3.52 (s, 2H, C*H*_2_), 2.08–2.01 (m, 2H, C*H*_2_), 1.97–1.84 (m, 2H, C*H*_2_), 1,40 ppm (s, 9H, COC(C*H*_3_)_3_). covered with solvent (2 × C*H*_2_); LC–MS (ESI+): *m/z* 513.2 [M+H]^+^ (calcd. *m/z* = 512.2 for C_26_H_29_ClN_4_O_3_S).

**2-Boc-5-(6-((4-Chlorophenyl)carbamoyl)benzo[*d*]thiazol-2-yl)hexahydropyrrolo[3,4-c]pyrrole (8h):** The product was purified using column chromatography (mobile phase: DCM:MeOH = 20:1). Yield: 66 mg (24%); white amorphous powder; R_f_(DCM:MeOH = 20:1) = 0.14; ^1^H NMR (400 MHz, [D_6_]DMSO, 25 °C, TMS): δ = 10.28 (s, 1H, Ar-N*H*-COR), 8.39 (d, *J* = 1.9 Hz, 1H, Ar-*H*_7_), 7.90 (dd, *J*_1_ = 8.5 Hz, *J*_2_ = 1.9 Hz, 1H, Ar-*H*_5_), 7.85–7.78 (m, 2H, 2 × Ar-*H*), 7.54 (d, *J* = 8.5 Hz, 1H, Ar-*H*_4_), 7.43–7.37 (m, 2H, 2 × Ar-*H*), 3.83–3.73 (m, 2H, 2 × C*H*), 3.61–3.50 (m, 2H, 2 × C*H*), 3.49–3.39 (m, 2H, 2 × C*H*), 3.26–3.18 (m, 2H, 2 × C*H*), 3.12–3.01 (m, 2H, 2 × C*H*), 1.39 ppm (s, 9H, COC(C*H*_3_)_3_); LC–MS (ESI+): *m/z* 499.0 [M+H]^+^ (calcd. *m/z* = 498.1 for C_25_H_27_ClN_4_O_3_S).

**4-(*N*-Boc-Amino)-1-(6-((4-chlorophenyl)carbamoyl)benzo[*d*]thiazol-2-yl)piperidine (8i):** The product was purified using column chromatography (mobile phase: DCM:MeOH = 30:1). Yield: 75 mg (28%); white amorphous powder; R_f_(DCM:MeOH = 30:1) = 0.28; ^1^H NMR (400 MHz, [D_6_]DMSO, 25 °C, TMS): δ = 10.28 (s, 1H, Ar-N*H*-COR), 8.37 (d, *J* = 1.9 Hz, 1H, Ar-*H*_7_), 7.89 (dd, *J*_1_ = 8.5 Hz, *J*_2_ = 1.9 Hz, 1H, Ar-*H*_5_), 7.86–7.78 (m, 2H, 2 × Ar-*H*), 7.51 (d, *J* = 8.5 Hz, 1H, Ar-*H*_4_), 7.43–7.37 (m, 2H, 2 × Ar-*H*), 6.95 (d, *J* = 7.9 Hz, 1H, CH-N*H*-Boc), 4.02 (d, *J* = 13.5 Hz, 2H, 2 × piperidine-*H*), 3.64–3.52 (m, 1H, piperidine-*H*), 3.31–3.26 (m, 2H, 2 × piperidine-*H*), 1.93–1.79 (m, 2H, 2 × piperidine-*H*), 1.50–1.41 (m, 2H, 2 × piperidine-*H*), 1.39 ppm (s, 9H, COC(C*H*_3_)_3_); ^13^C (101 MHz, [D_6_]DMSO, 25 °C, TMS): δ = 168.9, 164.5, 154.9, 154.2, 137.8, 129.9, 127.9 (2C), 126.4, 126.4, 125.5, 121.1 (2C), 120.5, 116.9, 77.1, 46.6 (2C), 46.2, 30.5 (2C), 27.6 ppm (3C); LC–MS (ESI+): *m/z* 487.1 [M+H]^+^ (calcd. *m/z* = 486.1 for C_24_H_27_ClN_4_O_3_S).

**4-(*N*-Boc-*N*-Methylamino)-1-(6-((4-chlorophenyl)carbamoyl)benzo[*d*]thiazol-2-yl)piperidine (8j):** The product was purified using column chromatography (mobile phase: DCM:MeOH = 20:1). Yield: 155 mg (50%); off-white amorphous powder; R_f_(DCM:MeOH = 20:1) = 0.12; ^1^H NMR (400 MHz, [D_6_]DMSO, 25 °C, TMS): δ = 10.29 (s, 1H, Ar-N*H*-COR), 8.38 (d, *J* = 1.9 Hz, 1H, Ar-*H*_7_), 7.90 (dd, *J*_1_ = 8.5 Hz, *J*_2_ = 1.9 Hz, 1H, Ar-*H*_5_), 7.85–7.79 (m, 2H, 2 × Ar-*H*), 7.52 (d, *J* = 8.5 Hz, 1H, Ar-*H*_4_), 7.44–7.36 (m, 2H, 2 × Ar-*H*), 4.24–3.96 (m, 3H, 3 × piperidine-*H*), 3.31–3.23 (m, 2H, 2 × piperidine-*H*), 2.67 (s, 3H, N-C*H*_3_) 1.83–1.65 (m, 4H, 4 × piperidine-*H*), 1.41 ppm (s, 9H, COC(C*H*_3_)_3_); LC–MS (ESI+): *m/z* 501.2 [M+H]^+^ (calcd. *m/z* = 500.2 for C_25_H_29_ClN_4_O_3_S).

**2-(4-Carbamoylpiperidin-1-yl)-*N*-(4-chlorophenyl)benzo[*d*]thiazole-6-carboxamide (8k):** The product was purified using column chromatography (mobile phase: DCM:MeOH = 17.5:1). Yield: 31 mg (23%); white amorphous powder; R_f_(DCM:MeOH = 17:1) = 0.08; ^1^H NMR (400 MHz, [D_6_]DMSO, 25 °C, TMS): δ = 10.29 (s, 1H, Ar-N*H*-COR), 8.38 (d, *J* = 1.9 Hz, 1H, Ar-*H*_7_), 7.90 (dd, *J*_1_ = 8.5 Hz, *J*_2_ = 1.9 Hz, 1H, Ar-*H*_5_), 7.85–7.79 (m, 2H, 2 × Ar-*H*), 7.52 (d, *J* = 8.5 Hz, 1H, Ar-*H*_4_), 7.43–7.38 (m, 2H, 2 × Ar-*H*), 7.36 (s, 1H, CH-CON*H_a_*), 6.87 (s, 1H, CH-CON*H_b_*), 4.08 (d, *J* = 13.0 Hz, 2H, 2 × piperidine-*H*), 3.29–3.21 (m, 2H, 2 × piperidine-*H*), 2.45–2.39 (m, 1H, piperidine-*H*), 1.90–1.79 (m, 2H, 2 × piperidine-*H*), 1.68–1.56 ppm (m, 2H, 2 × piperidine-*H*); ^13^C (101 MHz, [D_6_]DMSO, 25 °C, TMS): δ = 176.1, 170.2, 165.6, 155.9, 138.9, 130.8, 129.0 (2C), 127.5, 127.4, 126.6, 122.2 (2C), 121.6, 118.0, 48.4, 41.5 (2C), 28.2 ppm (2C); HRMS (ESI+) *m*/*z* calcd. for C_20_H_19_ClN_4_O_2_S+H^+^: 415.0990 [*M*+H]^+^: found 415.0966; HPLC: t_r_ = 5.41 min (99.0% at 254 nm).

**2-(3-Carbamoylpiperidin-1-yl)-*N*-(4-chlorophenyl)benzo[*d*]thiazole-6-carboxamide (8l):** The product was purified using column chromatography (mobile phase: DCM:MeOH = 15:1). Yield: 73 mg (65%); white amorphous powder; R_f_(DCM:MeOH = 9:1) = 0.27; ^1^H NMR (400 MHz, [D_6_]DMSO, 25 °C, TMS): δ = 10.29 (s, 1H, Ar-N*H*-COR), 8.38 (s, 1H, Ar-*H*), 7.90 (d, *J* = 8.5 Hz, 1H, Ar-*H*), 7.82 (d, *J* = 8.4 Hz, 2H, 2 × Ar-*H*), 7.52 (d, *J* = 8.5 Hz, 1H, Ar-*H*), 7.46 (s, 1H, CO-N*H_a_*), 7.41 (d, *J* = 8.4 Hz, 2H, 2 × Ar-*H*), 6.98 (s, 1H, CO-N*H_b_*), 4.15–4.07 (m, 1H, piperidine-*H*), 4.00–3.93 (m, 1H, piperidine- *H*), 3.30–3.19 (m, 2H, 2 × piperidine-*H*), 2.00–1.92 (m, 1H, piperidine- *H*), 1.85–1.77 (m, 1H, piperidine- *H*), 1.71–1.50 ppm (m, 3H, 3 × piperidine-*H*); ^13^C (101 MHz, [D_6_]DMSO, 25 °C, TMS): δ = 174.8, 170.2, 165.6, 155.9, 138.9, 130.8, 129.0 (2C), 127.5, 127.4, 126.6, 122.2 (2C), 121.6, 118.0, 51.0, 49.2, 41.8, 27.8, 24.3 ppm; HRMS (ESI+) *m*/*z* calcd. for C_20_H_19_ClN_4_O_2_S+H^+^: 415.0990 [*M*+H]^+^: found 415.0985; HPLC: t_r_ = 5.54 min (99.9% at 254 nm).

**2-(4-Hydroxypiperidin-1-yl)-*N*-(4-chlorophenyl)benzo[*d*]thiazole-6-carboxamide (8m):** The product was purified using column chromatography (mobile phase: EtOAc:Hex = 2:1). Yield: 49 mg (46%); white amorphous powder; R_f_(DCM:MeOH = 20:1) = 0.07; ^1^H NMR (400 MHz, [D_6_]DMSO, 25 °C, TMS): δ = 10.28 (s, 1H, Ar-N*H*-COR), 8.37 (d, *J* = 1.8 Hz, 1H, Ar-*H*_7_), 7.89 (dd, *J*_1_ = 8.5 Hz, *J*_2_ = 1.8 Hz, 1H, Ar-*H*_5_), 7.86–7.79 (m, 2H, 2 × Ar-*H*), 7.50 (d, *J* = 8.5 Hz, 1H, Ar-*H*_4_), 7.44–7.37 (m, 2H, 2 × Ar-*H*), 4.88 (d, *J* = 4.1 Hz, 1H, CH-O*H*), 3.94–3.83 (m, 2H, 2 × piperidine-*H*), 3.84–3.76 (m, 1H, piperidine-*H*), 3.46–3.37 (m, 2H, 2 × piperidine-*H*), 1.93–1.80 (m, 2H, 2 × piperidine-*H*), 1.56–1.41 ppm (m, 2H, 2 × piperidine-*H*); ^13^C (101 MHz, [D_6_]DMSO, 25 °C, TMS): δ = 170.1, 165.6, 156.0, 138.9, 130.9, 129.0 (2C), 127.4, 126.6, 122.2 (2C), 121.6, 118.0, 65.5, 46.41 (2C), 33.81 (2C) ppm; HRMS (ESI+) *m*/*z* calcd. for C_19_H_18_ClN_3_O_2_S+H^+^: 388.0881 [*M*+H]^+^: found 388.0885; HPLC: t_r_ = 6.00 min (96.2% at 254 nm).

**2-(4-Methylpiperidin-1-yl)-*N*-(4-chlorophenyl)benzo[*d*]thiazole-6-carboxamide (8n):** The product was purified using column chromatography (mobile phase: DCM:MeOH = 67:1). Yield: 27 mg (29%); white amorphous powder; R_f_(DCM:MeOH = 66:1) = 0.12; ^1^H NMR (400 MHz, [D_6_]DMSO, 25 °C, TMS): δ = 10.28 (s, 1H, Ar-N*H*-COR), 8.37 (d, *J* = 1.9 Hz, 1H, Ar-*H*_7_), 7.89 (dd, *J*_1_ = 8.5 Hz, *J*_2_ = 1.9 Hz, 1H, Ar-*H*_5_), 7.85–7.78 (m, 2H, 2 × Ar-*H*), 7.50 (d, *J* = 8.5 Hz, 1H, Ar-*H*_4_), 7.44–7.38 (m, 2H, 2 × Ar-*H*), 4.11–4.02 (m, 2H, 2 × piperidine-*H*), 3.25–3.17 (m, 2H, 2 × piperidine-*H*), 1.80–1.62 (m, 3H, 3 × piperidine-*H*), 1.28–1.14 (m, 2H, 2 × piperidine-*H*), 0.96 ppm (d, *J* = 6.4 Hz, 3H, C*H*_3_); ^13^C (101 MHz, [D_6_]DMSO, 25 °C, TMS): δ = 170.1, 165.6, 156.0, 138.9, 130.8, 128.9 (2C), 127.4, 127.4, 126.6, 122.2 (2C), 121.5, 117.9, 49.0 (2C), 33.5 (2C), 30.5, 22.0 ppm; HRMS (ESI+) *m*/*z* calcd. for C_20_H_20_N_3_OS+H^+^: 386.1088 [*M*+H]^+^: found 386.1083; HPLC: t_r_ = 7.49 min (99.4% at 254 nm).

**General procedure for the synthesis of compounds 9a-j**

Acidolysis was performed in the same manner as described for the synthesis of compounds **5a–m**.

**1-*N*-(6-((4-Chlorophenyl)carbamoyl)benzo[*d*]thiazol-2-yl)1,3-diaminopropane (9a):** Yield: 29 mg (62%); off-white amorphous powder; R_f_(DCM:MeOH = 9:1) = 0.0; ^1^H NMR (400 MHz, [D_6_]DMSO, 25 °C, TMS): δ = 10.25 (s, 1H, Ar-N*H*-COR), 8.28 (d, *J* = 1.8 Hz, 1H, Ar-*H*), 7.88–7.78 (m, 3H, 3 × Ar-*H*), 7.48–7.36 (m, 3H, 3 × Ar-*H*), 3.44 (t, *J* = 6.8 Hz, 2H, N-C*H*_2_-CH_2_), 2.63 (t, *J* = 6.8 Hz, 2H, N-C*H*_2_-CH_2_), 1.66 ppm (p, *J* = 6.8 Hz, 2H), not visible (N*H* and N*H*_2_); ^13^C (101 MHz, [D_6_]DMSO, 25 °C, TMS): δ = 168.8, 165.7, 156.0, 138.9, 130.7, 128.9 (2C), 127.4, 127.2, 126.2, 122.1 (2C), 121.3, 117.5, 42.4 (2C), 33.0 ppm; HRMS (ESI+) *m*/*z* calcd. for C_17_H_17_ClN_4_OS+H^+^: 361.0884 [*M*+H]^+^: found 361.0877; HPLC: t_r_ = 4.40 min (99.8% at 254 nm).

**3-Amino-1-(6-((4-chlorophenyl)carbamoyl)benzo[*d*]thiazol-2-yl)piperidine (9b):** Yield: 39 mg (89%); off-white amorphous powder; R_f_(DCM:MeOH = 4:1) = 0.0; ^1^H NMR (400 MHz, [D_6_]DMSO, 25 °C, TMS): δ = 10.27 (s, 1H, Ar-N*H*-COR), 8.36 (d, *J* = 1.8 Hz, 1H, Ar-*H*_7_), 7.89 (dd, *J*_1_ = 8.5 Hz, *J*_2_ = 1.8 Hz, 1H, Ar-*H*_5_), 7.85–7.78 (m, 2H, 2 × Ar-*H*), 7.49 (d, *J* = 8.5 Hz, 1H, Ar-*H*_4_), 7.43–7.38 (m, 2H, 2 × Ar-*H*), 3.95 (dd, *J*_1_ = 29.1 Hz, *J*_2_ = 12.8 Hz, 2H, CH-N*H*_2_), 3.23–3.14 (m, 1H, piperidine-*H*), 2.93–2.86 (m, 1H, piperidine-*H*), 2.79–2.70 (m, 1H, piperidine-*H*), 1.93–1.84 (m, 1H, piperidine-*H*), 1.83–1.70 (m, 3H, 3 × piperidine-*H*), 1.60–1.46 (m, 1H, piperidine-*H*), 1.34–1.21 ppm (m, 1H, piperidine-*H*); ^13^C (101 MHz, [D_6_]DMSO, 25 °C, TMS): δ = 170.2, 165.6, 156.0, 138.9, 130.8, 129.0 (2C), 127.4, 127.3, 126.6, 122.2 (2C), 121.5, 117.9, 57.1, 48.9, 47.9, 33.7, 23.7 ppm; HRMS (ESI+) *m*/*z* calcd. for C_19_H_19_ClN_4_OS+H^+^: 387.1041 [*M*+H]^+^: found 387.1017; HPLC: t_r_ = 5.43 min (97.9% at 254 nm).

**3-(*S*)-Amino-1-(6-((4-chlorophenyl)carbamoyl)benzo[*d*]thiazol-2-yl)pyrrolidine (9c):** Yield: 47 mg (80%); off-white amorphous powder; R_f_(DCM:MeOH = 9:1) = 0.03; ^1^H NMR (400 MHz, [D_6_]DMSO, 25 °C, TMS): δ = 10.27 (s, 1H, Ar-N*H*-COR), 8.37 (d, *J* = 1.8 Hz, 1H, Ar-*H*_7_), 7.88 (dd, *J*_1_ = 8.5 Hz, *J*_2_ = 1.9 Hz, 1H, Ar-*H*_5_), 7.84–7.78 (m, 2H, 2 × Ar-*H*), 7.52 (d, *J* = 8.5 Hz, 1H, Ar-*H*_4_), 7.44–7.37 (m, 2H, 2 × Ar-*H*), 3.73–3.46 (m, 4H, CH-N*H*_2_ + 2 × pyrrolidine-*H*), 3.29–3.15 (m, 1H, pyrrolidine-*H*), 2.17–2.04 (m, 1H, pyrrolidine-*H*), 1.90–1.74 ppm (m, 3H, 3 × pyrrolidine-*H*); ^13^C (101 MHz, [D_6_]DMSO, 25 °C, TMS): δ = 167.0, 165.7, 156.4, 138.9, 130.9, 129.0 (2C), 127.4, 126.9, 126.5, 122.2 (2C), 121.7, 117.7, 58.3, 51.4, 48.5, 34.5 ppm; [α]_D_^25^ = 11.5 (c = 0.77 in DMF); HRMS (ESI+) *m*/*z* calcd. for C_18_H_17_ClN_4_OS+H^+^: 373.0884 [*M*+H]^+^: found 373.0878; HPLC: t_r_ = 4.41 min (98.9% at 254 nm).

**3-(*R*)-Amino-1-(6-((4-chlorophenyl)carbamoyl)benzo[*d*]thiazol-2-yl)pyrrolidine (9d):** Yield: 41 mg (75%); off-white amorphous powder; R_f_(DCM:MeOH = 9:1) = 0.0; ^1^H NMR (400 MHz, [D_6_]DMSO, 25 °C, TMS): δ = 10.27 (s, 1H, Ar-N*H*-COR), 8.37 (d, *J* = 1.8 Hz, 1H, Ar-*H*_7_), 7.88 (dd, *J*_1_ = 8.5 Hz, *J*_2_ = 1.9 Hz, 1H, Ar-*H*_5_), 7.85–7.79 (m, 2H, 2 × Ar-*H*), 7.52 (d, *J* = 8.5 Hz, 1H, Ar-*H*_4_), 7.44–7.37 (m, 2H, 2 × Ar-*H*), 3.73–3.48 (m, 4H, CH-N*H*_2_ and 2 × pyrrolidine-*H*), 3.27–3.18 (m, 1H, pyrrolidine-*H*), 2.16–2.07 (m, 1H, pyrrolidine-*H*), 1.96–1.74 ppm (m, 3H, 3 × pyrrolidine-*H*); ^13^C (101 MHz, [D_6_]DMSO, 25 °C, TMS): δ = 167.0, 165.7, 156.4, 138.9, 130.9, 129.0 (2C), 127.4, 126.9, 126.5, 122.2 (2C), 121.7, 117.7, 58.2, 51.4, 48.5, 34.4 ppm; [α]_D_^25^ = - 11.5 (c = 0.77 in DMF);HRMS (ESI+) *m*/*z* calcd. for C_18_H_17_ClN_4_OS+H^+^: 373.0884 [*M*+H]^+^: found 373.0879; HPLC: t_r_ = 4.41 min (98.7% at 254 nm).

**4-Aminomethyl-1-(6-((4-chlorophenyl)carbamoyl)benzo[*d*]thiazol-2-yl)piperidin (9e):** Yield: 45 mg (94%); off-white amorphous powder; R_f_(DCM:MeOH = 9:1) = 0.0; ^1^H NMR (400 MHz, [D_6_]DMSO, 25 °C, TMS): δ = 10.28 (s, 1H, Ar-N*H*-COR), 8.36 (d, *J* = 1.8 Hz, 1H, Ar-*H*_7_), 7.89 (dd, *J*_1_ = 8.5 Hz, *J*_2_ = 1.8 Hz, 1H, Ar-*H*_5_), 7.84–7.80 (m, 2H, 2 × Ar-*H*), 7.50 (d, *J* = 8.5 Hz, 1H, Ar-*H*_4_), 7.43–7.38 (m, 2H, 2 × Ar-*H*), 4.13–4.05 (m, 2H, CH_2_-N*H*_2_), 3.19 (td, *J*_1_ =12.7 Hz, *J*_2_ = 2.6 Hz, 2H, CH-C*H*_2_-NH_2_), 2.47–2.44 (m, 2H, 2 × piperidine-*H*), 1.87–1.80 (m, 2H, 2 × piperidine-*H*), 1.59–1.40 (m, 3H, 3 × piperidine-*H*), 1.26–1.12 ppm (m, 2H, 2 × piperidine-*H*); ^13^C (101 MHz, [D_6_]DMSO, 25 °C, TMS): δ = 170.2, 165.6, 156.1, 138.9, 130.8, 129.0 (2C), 127.4, 127.4, 126.6, 122.2 (2C), 121.5, 117.9, 49.0 (2C), 47.7, 29.5 ppm (3C); HRMS (ESI+) *m*/*z* calcd. for C_20_H_21_ClN_4_OS+H^+^: 401.1197 [*M*+H]^+^: found 401.1192; HPLC: t_r_ = 5.49 min (97.7% at 254 nm).

**4-((6-((4-Chlorophenyl)carbamoyl)benzo[*d*]thiazol-2-yl)amino)piperidine (9f):** Yield: 39 mg (82%); off-white amorphous powder; R_f_(DCM:MeOH = 9:1) = 0.0; ^1^H NMR (400 MHz, [D_6_]DMSO, 25 °C, TMS): δ = 10.24 (s, 1H, Ar-N*H*-COR), 8.34 (d, *J* = 7.3 Hz, 1H, Ar-N*H*-CH), 8.27 (d, *J* = 1.7 Hz, 1H, Ar-*H*), 7.87–7.79 (m, 3H, 3 × Ar-*H*), 7.45 (d, *J* = 8.4 Hz, 1H, Ar-*H*), 7.42–7.37 (m, 2H, 2 × Ar-*H*), 3.82–3.76 (m, 1H, piperidine-*H*), 2.99–2.90 (m, 2H, 2 × piperidine-*H*), 1.98–1.88 (m, 2H, 2 × piperidine-*H*), 1.34 ppm (ddd, *J*_1_ = 14.9 Hz, *J*_2_ = 11.7 Hz, *J_3_* = 3.8 Hz, 2H, 2 × piperidine-*H*), not visible (2 × piperidine-*H* and N*H*); ^13^C (101 MHz, [D_6_]DMSO, 25 °C, TMS): δ = 167.6, 165.7, 156.1, 138.9, 130.7, 128.9 (2C), 127.4, 127.2, 126.2, 122.1 (2C), 121.3, 117.5, 52.5, 45.3 (2C), 33.4 ppm (2C); HRMS (ESI+) *m*/*z* calcd. for C_19_H_19_ClN_4_OS+H^+^: 387.1041 [*M*+H]^+^: found 387.1033; HPLC: t_r_ = 4.45 min (95.1% at 254 nm).

**7-(6-((4-Chlorophenyl)carbamoyl)benzo[*d*]thiazol-2-yl)-2,7-diazaspiro[4.4]nonane (9g):** Yield: 68 mg (85%); off-white amorphous powder; R_f_(DCM:MeOH = 9:1) = 0.0; ^1^H NMR (400 MHz, [D_6_]DMSO, 25 °C, TMS): δ = 10.27 (s, 1H, Ar-N*H*-COR), 8.39 (d, *J* = 1.8 Hz, 1H, Ar-*H*_7_), 7.89 (dd, *J*_1_ = 8.5 Hz, *J*_2_ = 1.8 Hz, 1H, Ar-*H*_5_), 7.84–7.79 (m, 2H, 2 × Ar-*H*), 7.52 (d, *J* = 8.5 Hz, 1H, Ar-*H*_4_), 7.43–7.37 (m, 2H, 2 × Ar-*H*), 3.69–3.40 (m, 4H, 2 × C*H*_2_), 2.94–2.86 (m, 2H, C*H*_2_), 2.81–2.70 (m, 2H, C*H*_2_), 2.07–1.97 (m, 2H, C*H*_2_), 1.81–1.68 ppm (m, 2H, C*H*_2_), not visible (N*H*); ^13^C (101 MHz, [D_6_]DMSO, 25 °C, TMS): δ = 166.9, 165.7, 156.3, 138.9, 130.9, 128.9 (2C), 127.4, 127.0, 126.6, 122.2 (2C), 121.7, 117.8, 59.3, 55.6, 49.9, 49.4, 45.8, 35.9, 35.5 ppm; HRMS (ESI+) *m*/*z* calcd. for C_21_H_21_ClN_4_OS+H^+^: 413.1197 [*M*+H]^+^: found 413.1190; HPLC: t_r_ = 4.59 min (97.0% at 254 nm).

**5-(6-((4-Chlorophenyl)carbamoyl)benzo[*d*]thiazol-2-yl)hexahydropyrrolo[3,4-*c*]pyrrole (9h):** Yield: 30 mg (70%); off-white amorphous powder; R_f_(DCM:MeOH = 9:1) = 0.0; ^1^H NMR (400 MHz, [D_6_]DMSO, 25 °C, TMS): δ = 10.28 (s, 1H, Ar-N*H*-COR), 8.38 (d, *J* = 1.9 Hz, 1H, Ar-*H*_7_), 7.89 (dd, *J*_1_ = 8.5 Hz, *J*_2_ = 1.9 Hz, 1H, Ar-*H*_5_), 7.85–7.79 (m, 2H, 2 × Ar-*H*), 7.53 (d, *J* = 8.5 Hz, 1H, Ar-*H*_4_), 7.43–7.38 (m, 2H, 2 × Ar-*H*), 3.82–3.75 (m, 2H, 2 × C*H*), 3.39–3.37 (m, 2H, 2 × C*H* ), 2.96–2.88 (m, 4H, 4 × C*H*), 2.74–2.69 ppm (m, 2H, 2 × C*H*), not visible (N*H*); ^13^C (101 MHz, [D_6_]DMSO, 25 °C, TMS): δ = 166.6, 165.7, 156.2, 138.9, 131.1, 128.9 (2C), 127.4, 127.1, 126.5, 122.2 (2C), 121.7, 117.9, 55.3 (2C), 53.2 (2C), 43.8 ppm (2C); HRMS (ESI+) *m*/*z* calcd. for C_20_H_19_ClN_4_OS+H^+^: 399.1040 [*M*+H]^+^: found 399.1034; HPLC: t_r_ = 4.47 min (98.8% at 254 nm).

**4-Amino-1-(6-((4-chlorophenyl)carbamoyl)benzo[*d*]thiazol-2-yl)piperidine (9i):** Yield: 32 mg (73%); off-white amorphous powder; R_f_(DCM:MeOH = 9:1) = 0.03; ^1^H NMR (400 MHz, [D_6_]DMSO, 25 °C, TMS): δ = 10.28 (s, 1H, Ar-N*H*-COR), 8.37 (d, *J* = 1.8 Hz, 1H, Ar-*H*_7_), 7.89 (dd, *J*_1_ = 8.5 Hz, *J*_2_ = 1.8 Hz, 1H, Ar-*H*_5_), 7.85–7.79 (m, 2H, 2 × Ar-*H*), 7.50 (d, *J* = 8.5 Hz, 1H, Ar-*H*_4_), 7.43–7.37 (m, 2H, 2 × Ar-*H*), 3.99 (d, *J* = 13.1 Hz, 2H, CH-N*H*_2_), 3.30–3.23 (m, 2H, 2 × piperidine-*H*), 2.91–2.83 (m, 1H, piperidine-*H*), 1.87–1.64 (m, 4H, 4 × piperidine-*H*), 1.37–1.23 ppm (m, 2H, 2 × piperidine-*H*); ^13^C (101 MHz, [D_6_]DMSO, 25 °C, TMS): δ = 170.1, 165.6, 156.1, 138.9, 130.9, 128.9 (2C), 127.4, 127.4, 126.6, 122.2 (2C), 121.5, 117.9, 48.0, 47.6 (2C), 34.8 ppm(2C); HRMS (ESI+) *m*/*z* calcd. for C_19_H_18_ClN_3_O_2_S+H^+^: 387.1041 [*M*+H]^+^: found 387.1033; HPLC: t_r_ = 4.54 min (98.9% at 254 nm).

**4-(*N*-Methylamino)-1-(6-((4-chlorophenyl)carbamoyl)benzo[*d*]thiazol-2-yl)piperidine (9j):** Yield: 89 mg (74%); off-white amorphous powder; R_f_(DCM:MeOH = 9:1) = 0.0; ^1^H NMR (400 MHz, [D_6_]DMSO, 25 °C, TMS): δ = 10.28 (s, 1H, Ar-N*H*-COR), 8.37 (d, *J* = 1.8 Hz, 1H, Ar-*H*_7_), 7.89 (dd, *J*_1_ = 8.5 Hz, *J*_2_ = 1.8 Hz, 1H, Ar-*H*_5_), 7.85–7.78 (m, 2H, 2 × Ar-*H*), 7.50 (d, *J* = 8.5 Hz, 1H, Ar-*H*_4_), 7.44–7.37 (m, 2H, 2 × Ar-*H*), 3.93–4.00 (m, 2H, 2 × piperidine-*H*), 3.32–3.26 (m, 2H, 2 × piperidine-*H*), 2.63–2.54 (m, 1H, piperidine-*H*), 2.30 (s, 3H, NH-C*H*_3_), 1.97–1.86 (m, 2H, 2 × piperidine-*H*), 1.83–1.67 (m, 1H, N*H*), 1.39–1.28 ppm (m, 2H, 2 × piperidine-*H*); ^13^C (101 MHz, [D_6_]DMSO, 25 °C, TMS): δ = 170.1, 165.6, 156.0, 138.9, 130.9, 128.9 (2C), 127.4, 127.4, 126.6, 122.2 (2C), 121.5, 118.0, 55.7, 47.3 (2C), 33.7, 31.3 ppm (2C); HRMS (ESI) *m*/*z* calcd. for C_20_H_21_ClN_4_OS+H^+^: 401.1197 [*M*+H]^+^: found 401.1192; HPLC: t_r_ = 4.53 min (99.6% at 254 nm).

**4-(*N*,*N*-Dimethylamino)-1-(6-((4-chlorophenyl)carbamoyl)benzo[*d*]thiazol-2-yl)piperidine (10):** Compound **9j** (60 mg, 0.15 mmol) was dissolved in a mixture of DCM (5 mL) and MeOH (5 mL). Formaldehyde_(aq)_ (56 μL, 37% [w/w], 1.08 g/mL, 0.75 mmol) and acetic acid (8.6 μL, 100%, 1.05 g/mL, 0.15 mmol) were added. After 2 h, NaCNBH_3_ (15 mg, 0.24 mmol) was added. The reaction mixture was stirred overnight at room temperature. The solvent was evaporated under reduced pressure and the residue was taken up in DCM (30 mL). The organic phase was washed with 1 M NaOH (3 × 25 mL), saturated NaCl (25 mL) and dried over Na_2_SO_4_. The solvent was evaporated under reduced pressure. Yield: 56 mg (90%); white amorphous powder; R_f_(DCM:MeOH = 9:1) = 0.03; ^1^H NMR (400 MHz, [D_6_]DMSO, 25 °C, TMS): δ = 10.29 (s, 1H, Ar-N*H*-COR), 8.38 (d, *J* = 1.9 Hz, 1H, Ar-*H*_7_), 7.90 (dd, *J*_1_ = 8.5 Hz, *J*_2_ = 1.9 Hz, 1H, Ar-*H*_5_), 7.88–7.78 (m, 2H, 2 × Ar-*H*), 7.51 (d, *J* = 8.5 Hz, 1H, Ar-*H*_4_), 7.44–7.37 (m, 2H, 2 × Ar-*H*), 4.08 (d, *J* = 13 Hz, 2H, 2 × piperidine-*H*), 3.29–3.16 (m, 2 × piperidine-*H*), 2.20 (s, 6H, N(C*H*_3_)_2_), 1.92–1.84 (m, 2H, 2 × piperidine-*H*), 1.55–1.42 ppm (m, 2H, 2 × piperidine-*H*), covered with solvent (1H, piperidine-*H*); ^13^C (101 MHz, [D_6_]DMSO, 25 °C, TMS): δ = 170.0, 165.6, 156.0, 138.9, 130.9, 128.9 (2C), 127.5, 127.4, 126.6, 122.2 (2C), 121.6, 118.0, 61.1, 48.0 (2C), 41.9 (2C), 27.9 ppm (2C); HRMS (ESI+) *m*/*z* calcd. for C_21_H_23_ClN_4_OS+H^+^: 415.1354 [*M*+H]^+^: found 415.1346; HPLC: t_r_ = 5.02 min (96.9% at 254 nm).

**Ethyl 2-(4-(*N*-Boc-amino)piperidin-1-yl)benzo[*d*]thiazole-6-carboxylate (11):** Ethyl 2-bromobenzo[d]thiazole-6-carboxylate (750 mg, 2.62 mmol) and 4-(*N*-Boc-amino)piperidine (1.31 g, 6.55 mmol) were dissolved in THF (100 mL). The reaction mixture was then stirred at r.t. overnight. The precipitate was filtered off and the solvent was evaporated under reduced pressure. The residue was taken up in ethyl acetate (100 mL) and washed with 1% (*w*/*v*) citric acid (3 × 50 mL) and saturated NaCl (50 mL). The organic phase was dried over Na_2_SO_4_, filtered and the solvent was evaporated under reduced pressure. Yield: 1.05 g (99%); yellow amorphous powder; R_f_(EtOAc:Hex = 1:2) = 0.16; ^1^H NMR (400 MHz, CDCl_3_, 25 °C, TMS): δ = 8.30 (d, *J* = 1.8 Hz, 1H, Ar-*H*_7_), 8.00 (dd, *J*_1_ = 8.5 Hz, *J*_2_ = 1.8 Hz, 1H, Ar-*H*_5_), 7.51 (d, *J* = 8.5 Hz, 1H, Ar-*H*_4_), 4.52–4.44 (m, 1H, CH-N*H*-CO), 4.37 (q, *J* = 7.1 Hz, 2H, COO-C*H*_2_-CH_3_), 4.19–4.10 (m, 2H, 2× piperidine-*H*), 3.82–3.70 (m, 1H, piperidine-*H*), 3.34–3.24 (m, 2H, 2× piperidine-*H*), 2.14–2.06 (m, 2H, 2× piperidine-*H*), 1.54–1.49 (m, 2H, 2× piperidine-*H*), 1.46 (s, 9H, COC(C*H*_3_)_3_), 1.40 ppm (t, *J* = 7.1 Hz, 3H, COO-CH_2_-C*H*_3_); LC–MS (ESI+): *m/z* 406.3 [M+H]+ (calcd. *m/z* = 405.2 for C_20_H_27_N_3_O_4_S).

**2-(4-(*N*-Boc-Amino)piperidin-1-yl)benzo[*d*]thiazole-6-carboxylic acid (12):** Compound **11** (1.00 g, 2.47 mmol) was suspended in ethanol (96%, 50 mL). Then, 2 M NaOH_(aq)_ (12.3 mL, 24.6 mmol) was added. The mixture was heated to 100 °C and left to stir for 1 h. After that, the reaction mixture was cooled and acidified to pH 3 using 2 M HCl_(aq)_. The precipitated product was then filtered off under pressure. Yield: 258 mg (93%); yellow amorphous powder; R_f_(DCM:MeOH = 9:1) = 0.34; ^1^H NMR (400 MHz, [D_6_]DMSO, 25 °C, TMS): δ = 12.71 (s, 1H, COO*H*), 8.35 (d, *J* = 1.7 Hz, 1H, Ar-*H*_7_), 7.84 (dd, *J*_1_ = 8.5 Hz, *J*_2_ = 1.7 Hz, 1H, Ar-*H*_5_), 7.45 (d, *J* = 8.5 Hz, 1H, Ar-*H*_4_), 6.94 (d, *J* = 7.9 Hz, 1H, CH-N*H*-CO), 4.06–3.96 (m, *J* = 12.9 Hz, 2H, 2× piperidine-*H*), 3.68–3.51 (m, 1H, C*H-*NH), 1.86 (m, 2H, 2× piperidine-*H*), 1.50–1.33 ppm (m, 11H, 2× piperidine-*H* and COC(C*H*_3_)_3_), not visible (2× piperidine-*H*); LC–MS (ESI+): *m/z* 378.0 [M+H]^+^ (calcd. *m/z* = 377.1 for C_18_H_23_N_3_O_4_S).

**The amide coupling** procedure was used for the synthesis of **4-(*N*-Boc-amino)-1-(6-((3,4-dichlorophenyl)carbamoyl)benzo[*d*]thiazol-2-yl)piperidine (13):** Yield: 258 mg (48%); off-white amorphous powder; R_f_(EtOAc:Hex = 1:2) = 0.39; ^1^H NMR (400 MHz, [D_6_]DMSO, 25 °C, TMS): δ = 10.31 (s, 1H, Ar-N*H*-COR), 8.40 (d, *J* = 1.8 Hz, 1H, Ar-*H*_7_), 7.91 (dd, *J*_1_ = 8.5, *J*_2_ = 1.8 Hz, 1H, Ar-*H*_5_), 7.82 (d, *J* = 8.9 Hz, 2H, 2 × Ar-*H*), 7.55 (d, *J* = 8.5 Hz, 1H, Ar-*H*_4_), 7.41 (d, *J* = 8.9 Hz, 2H, 2 × Ar-*H*), 3.69–3.58 (m, 4H, 2 × piperazine-C*H*_2_), 3.54–3.46 (m, 4H, 2 × piperazine-C*H*_2_), 1.43 ppm (s, 9H, COC(C*H*_3_)_3_); LC–MS (ESI+): *m/z* 473.1 [M+H]^+^ (calcd. *m/z* = 472.1 for C_23_H_25_ClN_4_O_3_S).

**The general procedure for the preparation of compounds 5a****–m** was used for the synthesis of **4-amino-1-(6-((3,4-dichlorophenyl)carbamoyl)benzo[*d*]thiazol-2-yl)piperidine (14)**: Yield: 220 mg (91%); off-white amorphous powder; R_f_(DCM:MeOH = 9:1) = 0.02; ^1^H NMR (400 MHz, [D_6_]DMSO, 25 °C, TMS): δ = 10.47 (s, 1H, Ar-N*H*-COR), 8.40 (d, *J* = 1.8 Hz, 1H, Ar-*H*_7_), 8.19 (d, *J* = 2.4 Hz, 1H, Ar-*H*_20_), 7.91 (dd, *J*_1_ = 8.5, *J*_2_ = 1.8 Hz, 1H, Ar-*H*_5_), 7.79 (dd, *J* = 8.9, 2.4 Hz, 1H, Ar-*H_24_*), 7.61 (d, *J* = 8.9 Hz, 1H, Ar-*H*_23_), 7.50 (d, *J* = 8.5 Hz, 1H, Ar-*H*_4_), 3.99 (d, *J* = 13.2 Hz, 2H, CH-N*H*_2_), 3.33–3.20 (m, 2H, 2 × piperidin-*H*) 2.91–2.83 (m, 1H, C*H*-NH_2_), 1.88–1.78 (m, 2H, 2 × piperidin-*H*), 1.88–1.58 (m, 4H, 4 × piperidin-*H*), 1.37–1.25 ppm (m, 2H, 2 × piperidin-*H*); ^13^C (101 MHz, [D_6_]DMSO, 25 °C, TMS): δ = 170.2, 165.8, 156.3, 140.2, 131.2, 130.9 (2C), 126.9, 126.8, 125.2, 121.8 (2C), 120.7, 117.9, 48.0 (2C), 47.6 (2C), 34.8 ppm; HRMS (ESI+) *m*/*z* calcd. for C_19_H_18_Cl_2_N_4_OS+H^+^: 421.0651 [*M*+H]^+^: found 421.0645; HPLC: t_r_ = 6.31 min (95.1% at 254 nm).
