# Supplementary material for: Structure-Activity Relationships of Benzothiazole-Based Hsp90 C-Terminal-Domain Inhibitors
Source: Pharmaceutics. 2021 Aug 17;13(8):1283. doi: 10.3390/pharmaceutics13081283 (PMC8400049; doi:10.3390/pharmaceutics13081283)
Supplement: Supplementary file 1 [file pharmaceutics-13-01283-s001.zip › pharmaceutics-1335106-supplementary.pdf]

pharmaceutics

MDPI

Supplementary Materials: Structure–Activity Relationships of Benzothiazole-Based Hsp90 C-Terminal-Domain Inhibitors

Jaka Dernovšek, Živa Zajec, Martina Durcik, Lucija Peterlin Mašič, Martina Gobec, Nace Zidar and Tihomir Tomašič

Representative ^{1}H NMR, ^{13}C NMR spectra and HPLC chromatograms

^{1}H NMR (400 MHz, $CDCl_3$, 25 °C, TMS): δ = 8.55 (d, *J* = 1.5 Hz, 1H, Ar-H_7), 8.16 (dd, J_1 = 8.5 Hz, J_2 = 1.5 Hz, 1H, Ar-H_5), 8.02 (d, *J* = 8.5 Hz, 1H, Ar-H_4), 4.43 (q, *J* = 7.1 Hz, 2H, COO-CH_2-CH_3), 1.43 ppm (t, *J* = 7.1 Hz, 3H, COO-CH_2-CH_3)

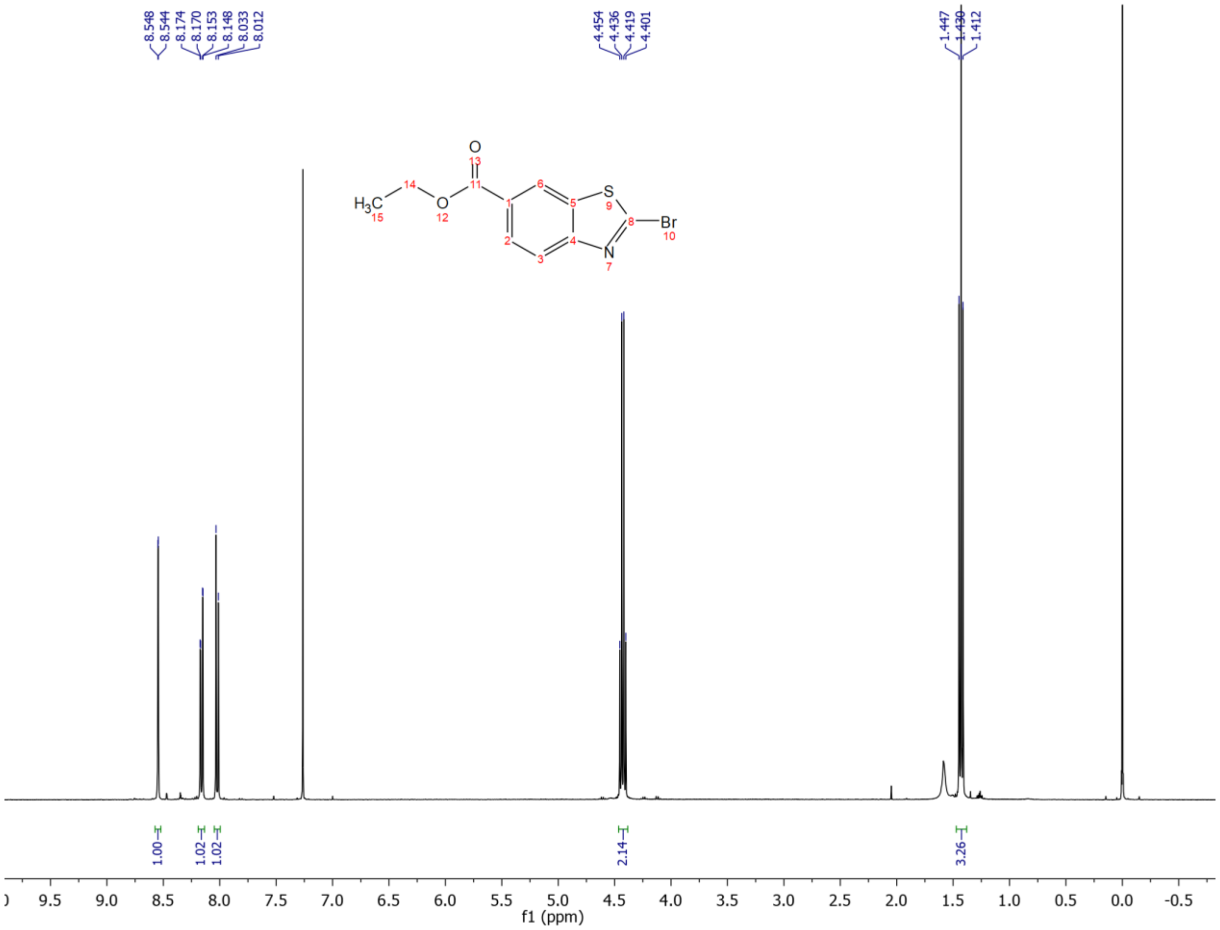

Figure S1. ^{1}H NMR spectrum of compound **1**.

^{13}C NMR (101 MHz, $CDCl_3$, 25 °C, TMS): δ = 165.8, 155.1, 142.4, 137.3, 127.9, 127.8, 122.9, 122.5, 61.5, 14.4 ppm.

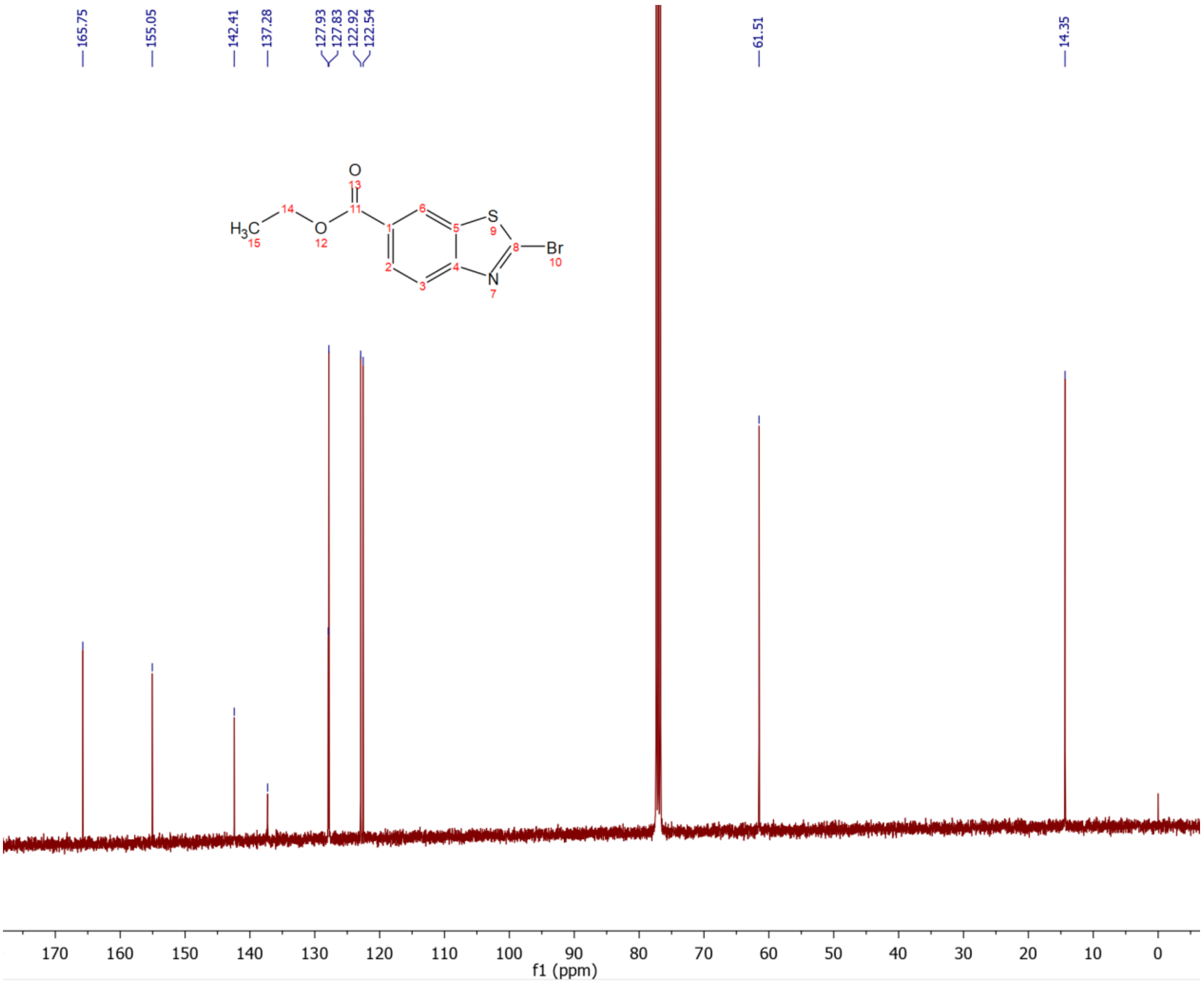

Figure S2. ^{13}C NMR spectrum of compound **1**.

^{1}H NMR (400 MHz, $CDCl_3$, 25°C, TMS): δ = 8.32 (d, J = 1.5 Hz, 1H, Ar-H_7), 8.01 (dd, J_1 = 8.5 Hz, J_2 = 1.8 Hz, 1H, Ar-H_5), 7.53 (d, J = 8.5 Hz, 1H, Ar-H_4), 4.38 (q, J = 7.1 Hz, 2H, COO-CH_2-CH_3), 3.69 – 3.63 (m, 4H, 2 × piperazine-CH_2), 3.63 – 3.56 (m, 4H, 2 × piperazine-CH_2), 1.49 (s, 9H, COC(CH_3)$_3$), 1.40 ppm (t, J = 7.1 Hz, 3H, COO-CH_2-CH_3)

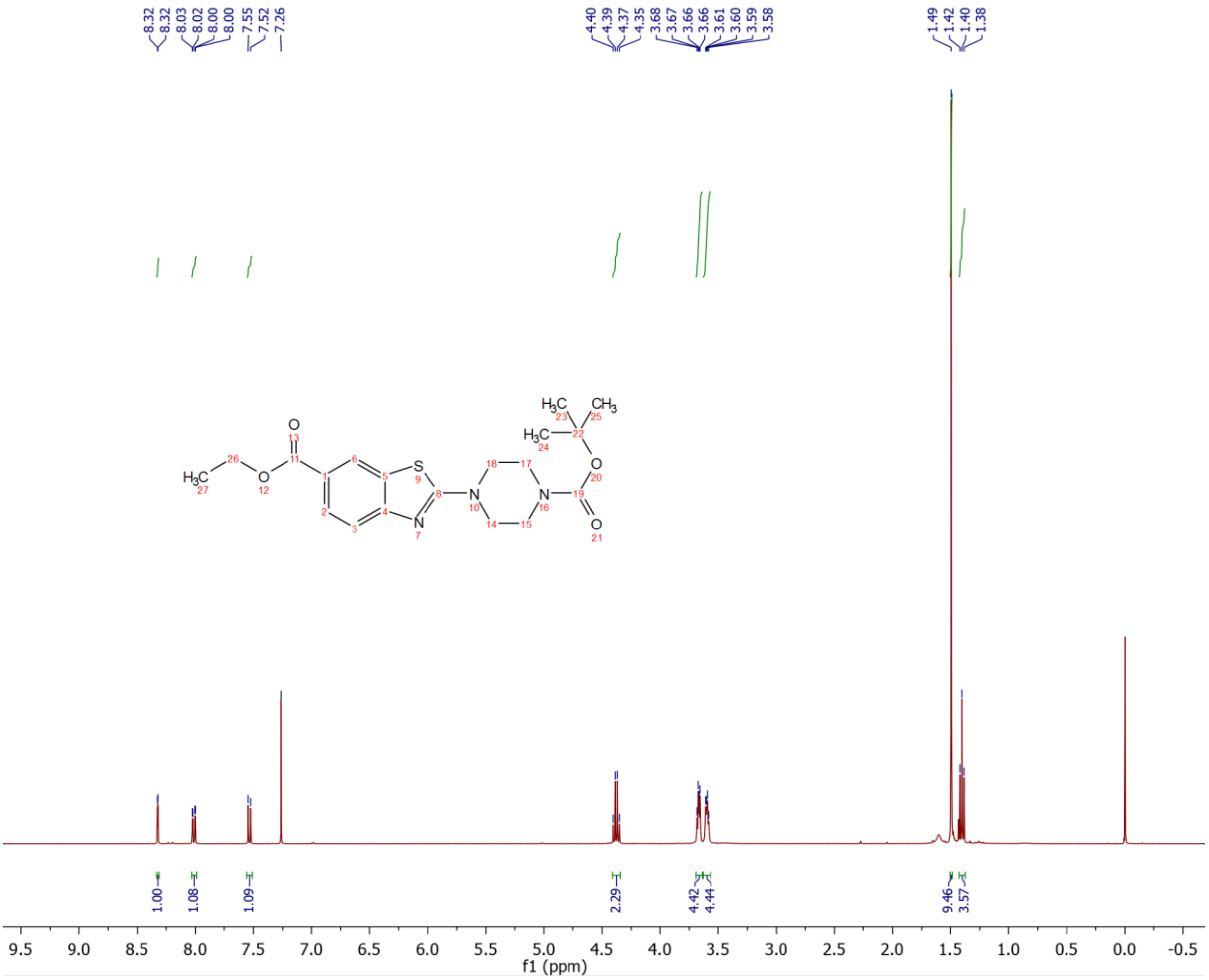

Figure S3. ^{1}H NMR spectrum of compound **2**.

^{13}C NMR (101 MHz, $CDCl_3$, 25°C, TMS): δ = 170.7, 166.4, 156.3, 154.5, 130.6, 128.0, 123.7, 122.8, 118.5, 80.6 (2C), 60.9, 48.2 (2C), 28.4 (3C), 14.4 ppm

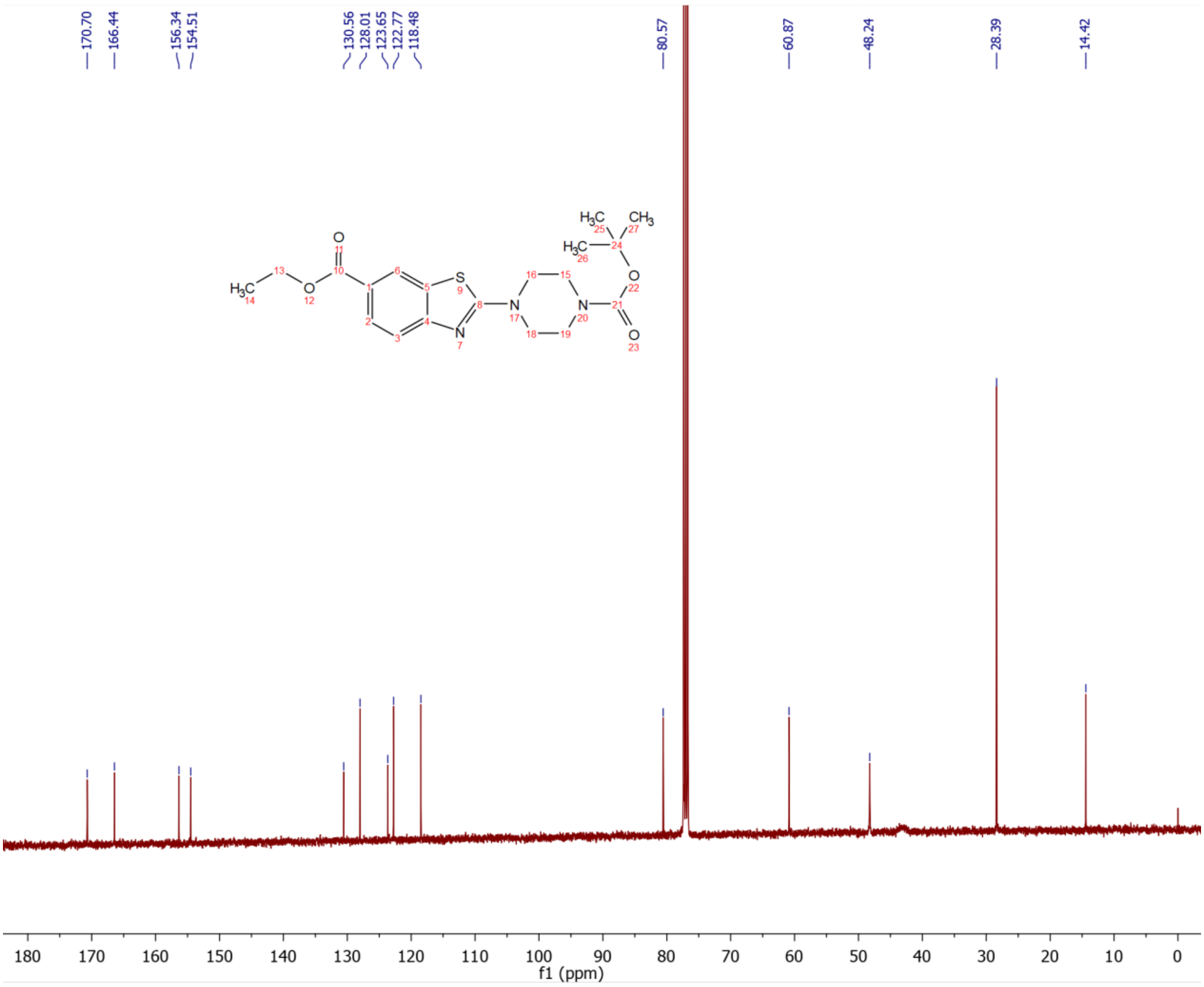

Figure S4. ^{13}C NMR spectrum of compound **2**.

^{1}H NMR (400 MHz, $CDCl_3$, 25°C, TMS): δ = 8.39 (d, *J* = 1.6 Hz, 1H, Ar-H_7), 8.08 (dd, J_1 = 8.5 Hz, J_2 = 1.6 Hz, 1H, Ar-H_5), 7.58 (d, *J* = 8.5 Hz, 1H, Ar-H_4), 3.73 – 3.66 (m, 4H, 2 × piperazine-CH_2), 3.65 – 3.58 (m, 4H, 2 × piperazine-CH_2), 1.50 ppm (s, 9H, COC(CH_3)$_3$)

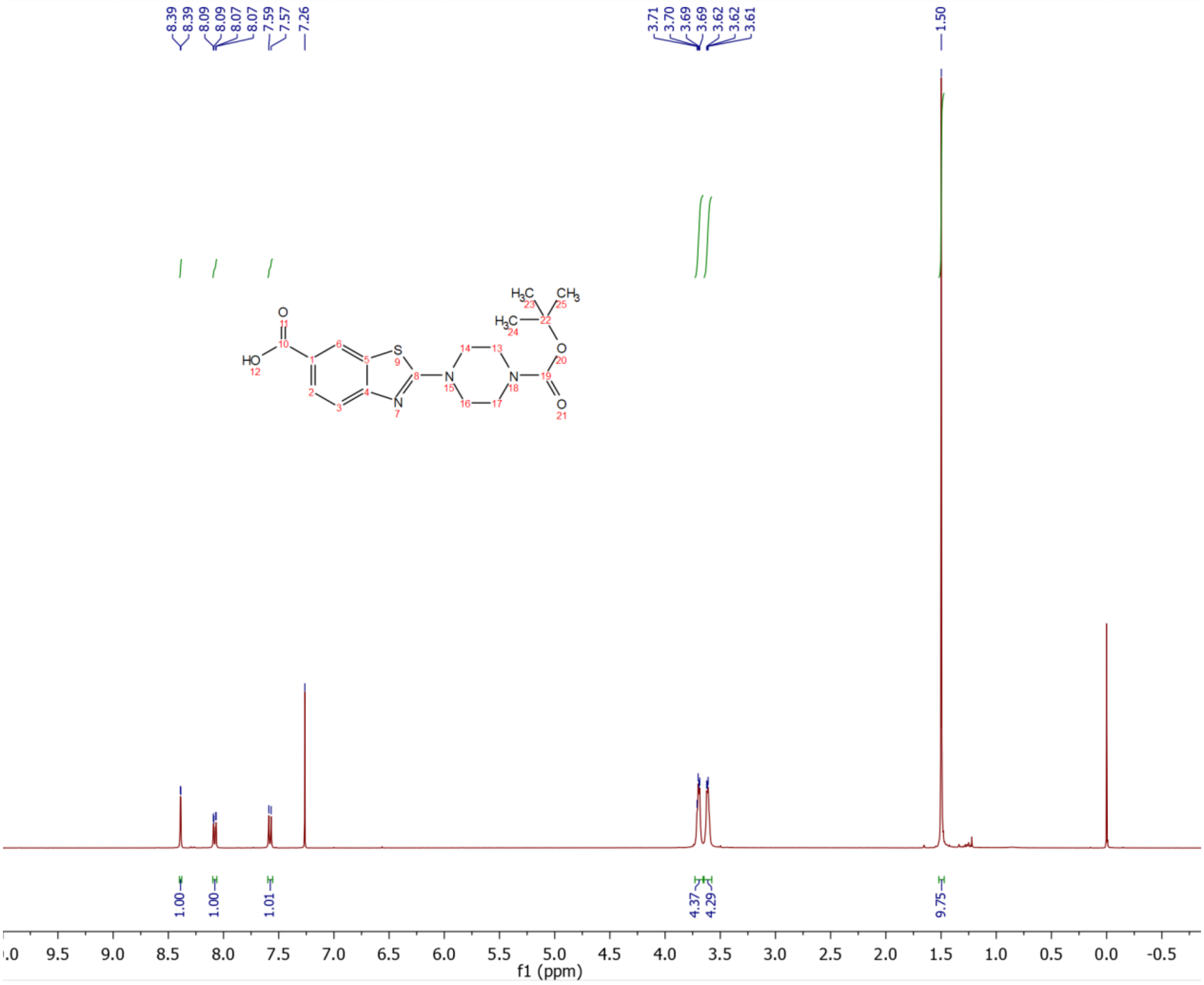

Figure S5. ^{13}H NMR spectrum of compound **3**.

^{13}C NMR (101 MHz, [D$_6$]DMSO, 25°C, TMS): δ = 170.9, 167.6, 156.5, 154.2, 130.9, 128.1, 123.8, 123.6, 118.4, 79.8 (2C), 48.2 (2C), 28.5 (3C) ppm

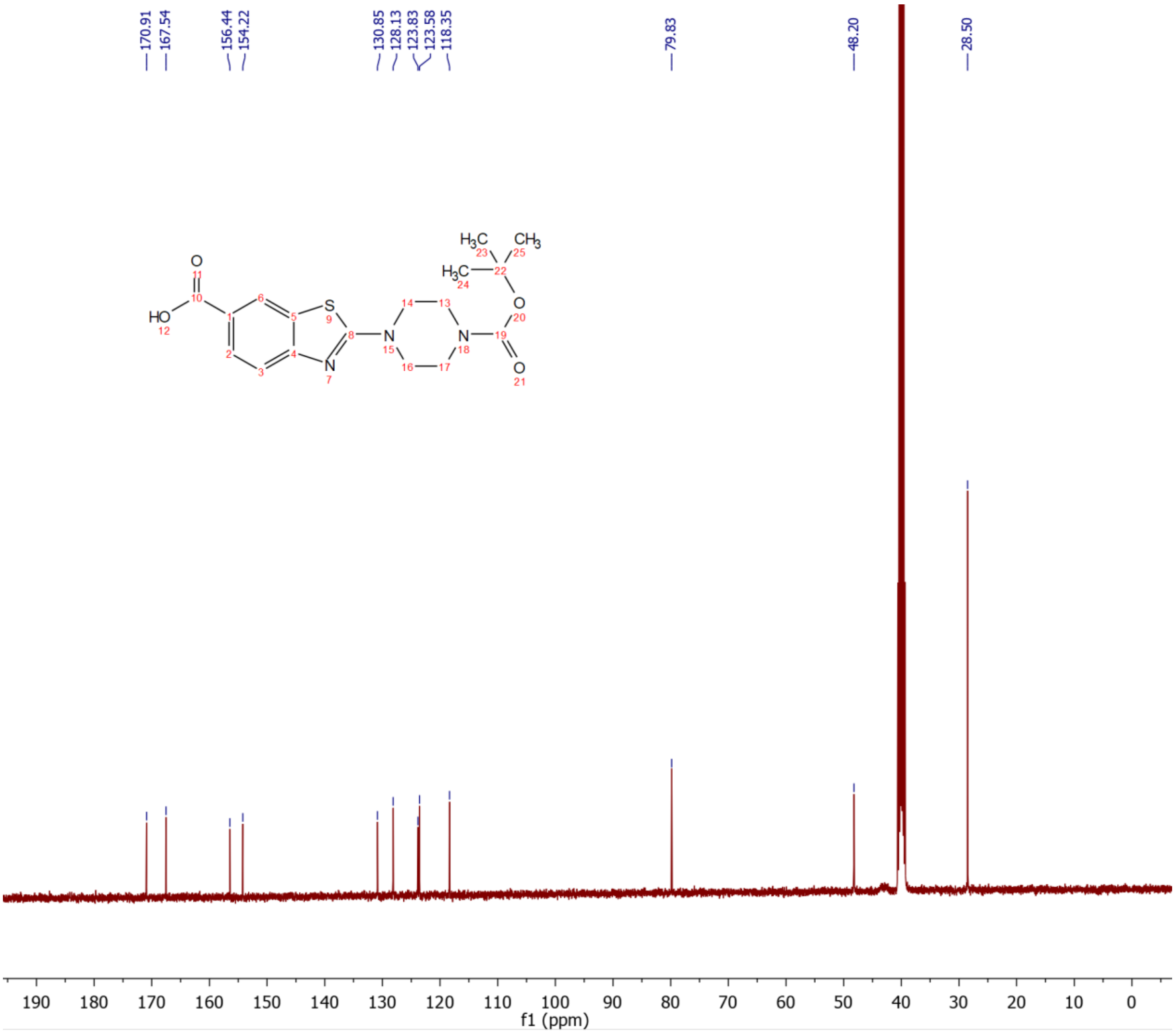

Figure S6. ^{13}C NMR spectrum of compound **3**.

^{1}H NMR (400 MHz, [D$_6$]DMSO, 25°C, TMS): δ = 10.31 (s, 1H, Ar-N*H*-COR), 8.40 (d, *J* = 1.8 Hz, 1H, Ar-*H*$_7$), 7.91 (dd, *J*$_1$ = 8.5, *J*$_2$ = 1.8 Hz, 1H, Ar-*H*$_5$), 7.82 (d, *J* = 8.9 Hz, 2H, 2 × Ar-*H*), 7.55 (d, *J* = 8.4 Hz, 1H, Ar-*H*$_4$), 7.41 (d, *J* = 8.9 Hz, 2H, 2 × Ar-*H*), [illegible] C*H*$_3$)$_3$);

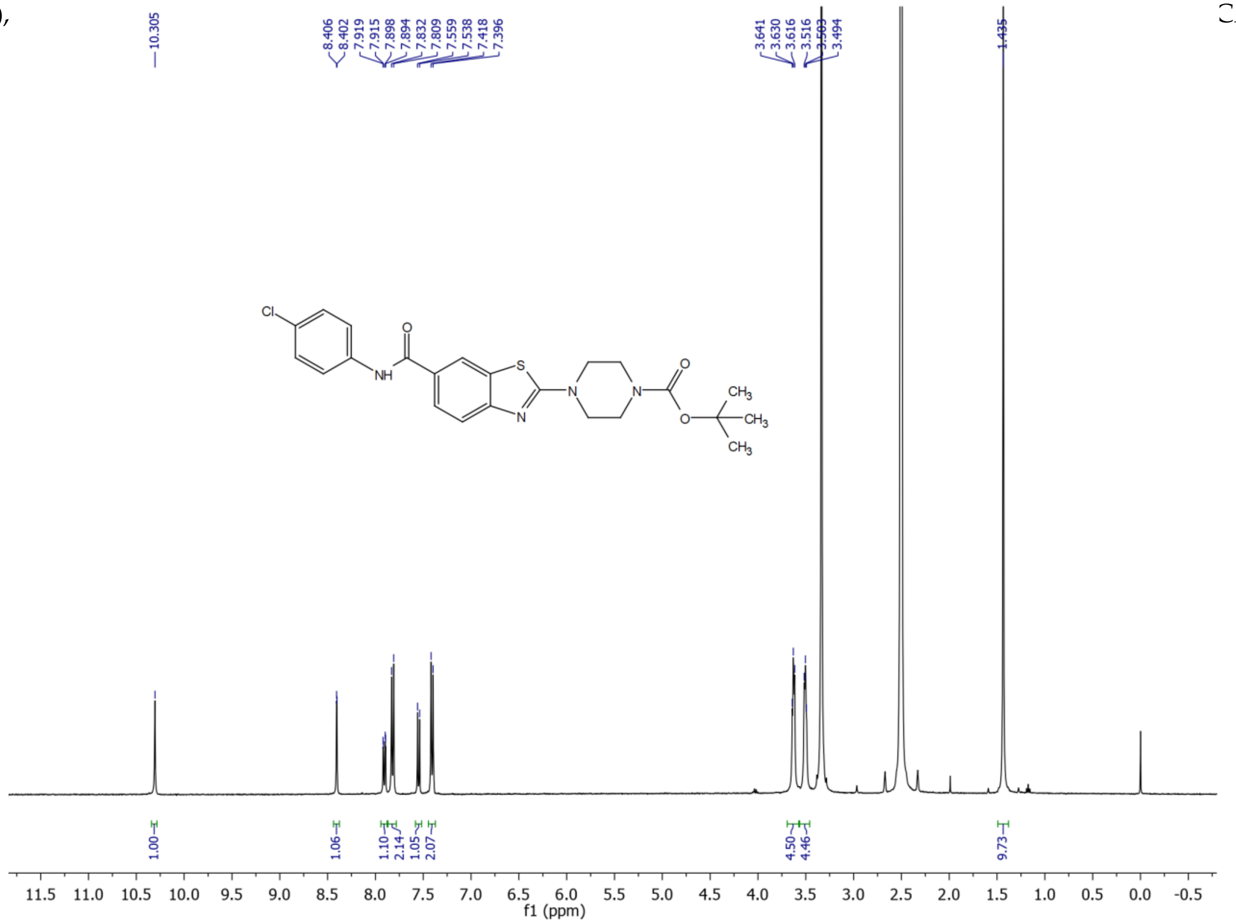

Figure S7. ^{1}H NMR spectrum of compound **4c**.

^{1}H NMR (400 MHz, [D$_6$]DMSO, 25°C, TMS): δ = 10.16 (s, 1H, Ar-N*H*-COR), 8.38 (d, *J* = 1.8 Hz, 1H, Ar-*H*$_7$), 7.90 (dd, J_1 = 8.5 Hz, J_2 = 1.8 Hz, 1H, Ar-*H*$_5$), 7.82 – 7.75 (m, 2H, 2 × Ar-*H*), 7.51 (d, *J* = 8.5 Hz, 1H, Ar-*H*$_4$), 7.39 – 7.30 (m, 2H, 2 × Ar-*H*), 7.13 – 7.04 (m, 1H, Ar-*H*), 3.58 – 3.48 (m, 4H, 2 × piperazine-C*H*$_2$), 2.87 – 2.76 ppm (m, 4H, 2 × piperazine-C*H*$_2$)

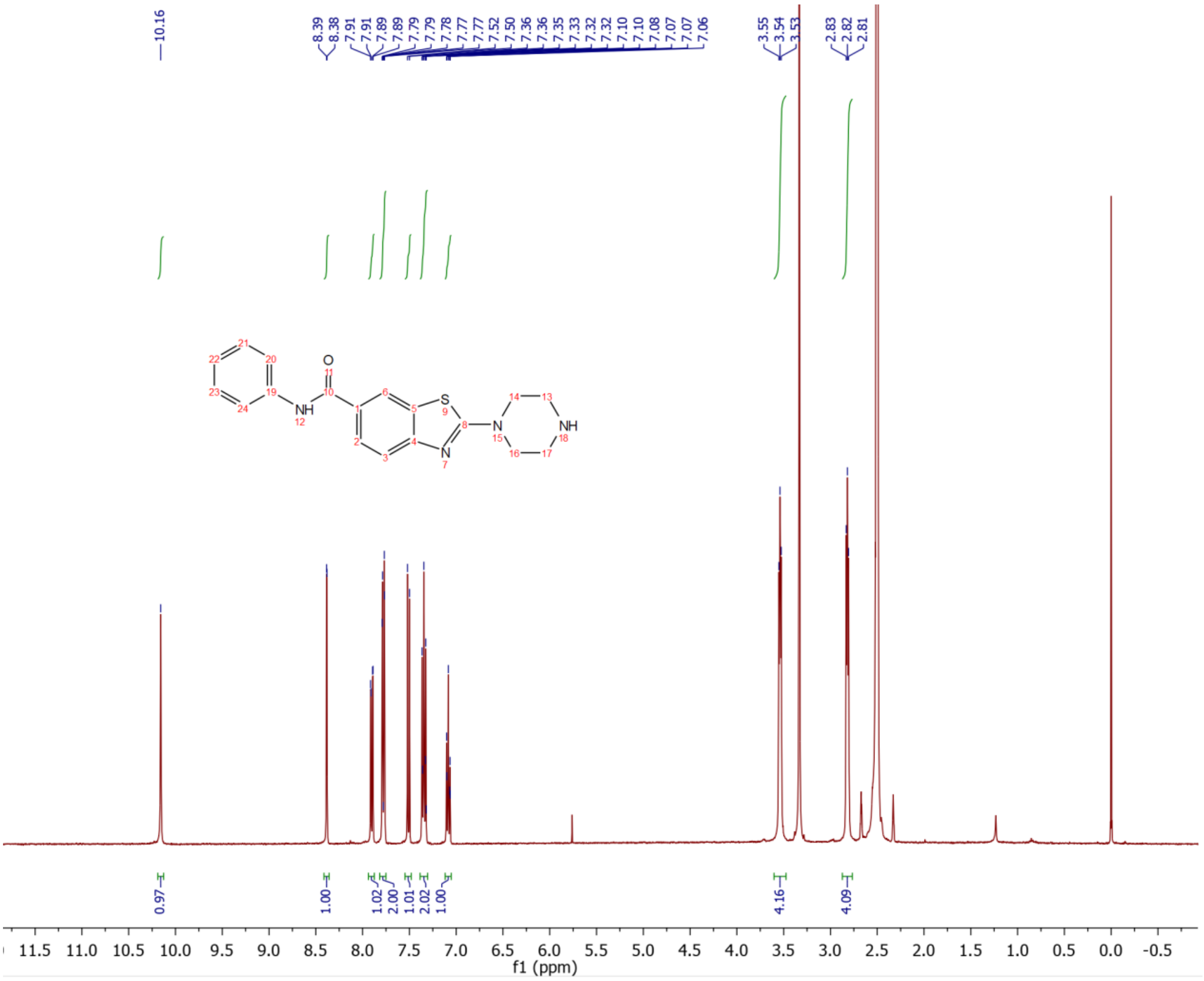

Figure S8. ^{1}H NMR spectrum of compound **5a**.

^{13}C (101 MHz, [D$_6$]DMSO, 25°C, TMS): δ = 170.6, 165.5, 155.7, 139.9, 130.6, 129.0 (2C), 127.8, 126.5, 123.9, 121.5, 120.7 (2C), 118.1, 49.9 (2C), 45.5 ppm (2C);

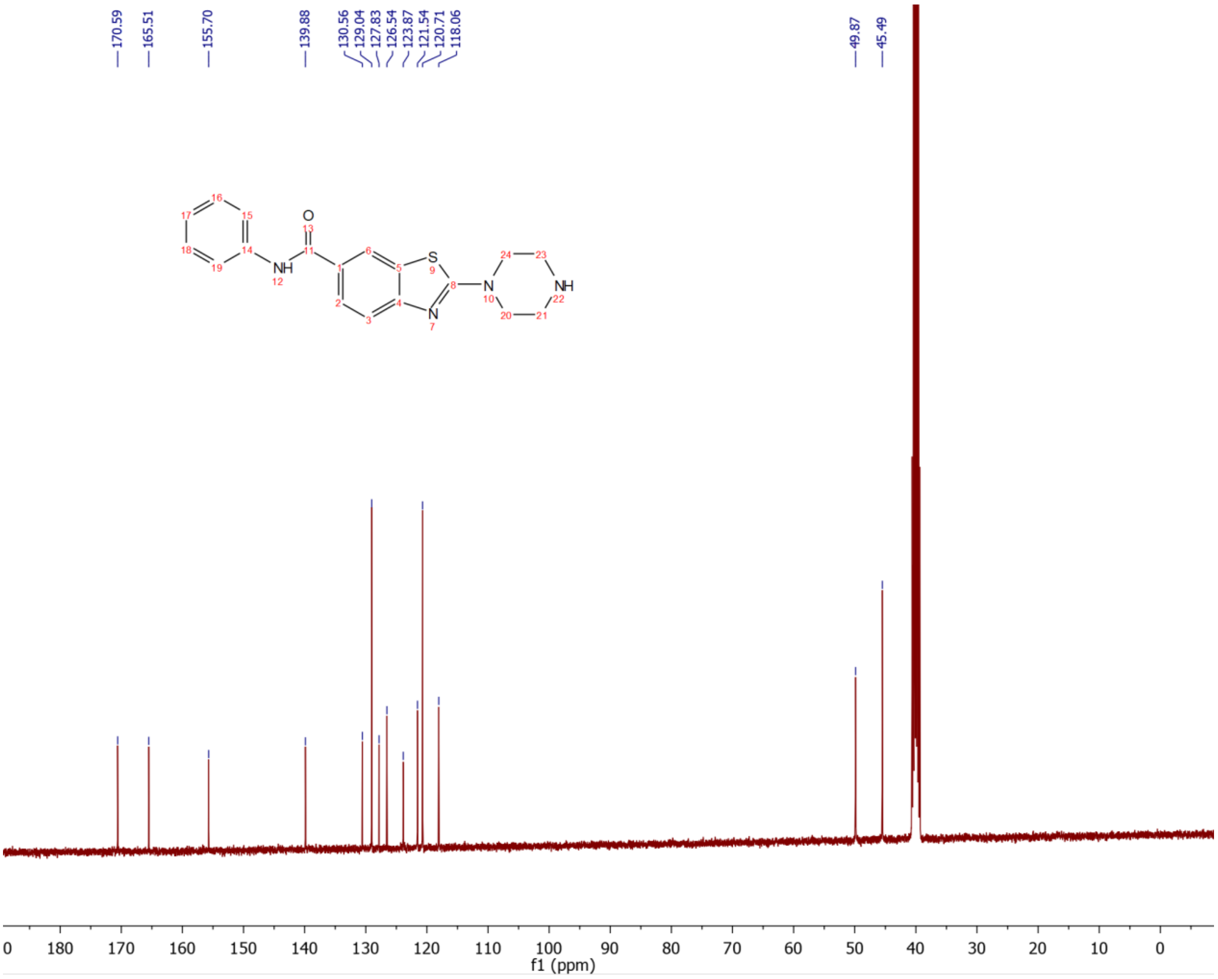

Figure S9. ^{13}C NMR spectrum of compound **5a**.

HPLC: t_r = 2.27 min (97.4 % at 254 nm)

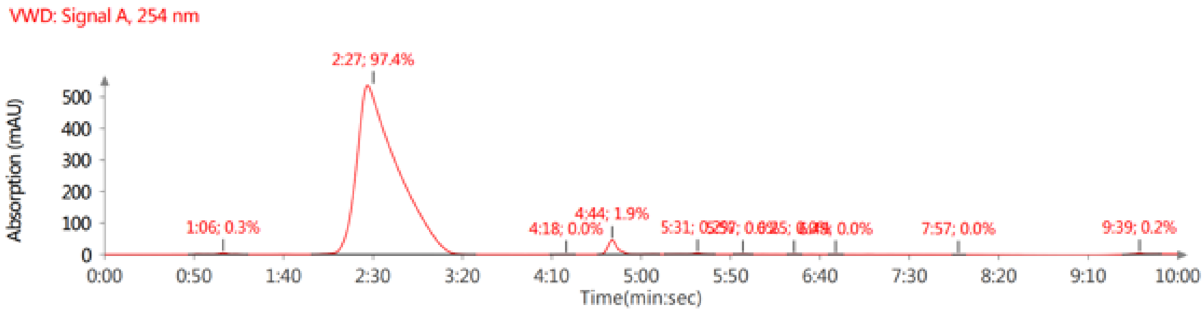

Figure S10. HPLC chromatogram for compound **5a**.

^{1}H NMR (400 MHz, [D$_6$]DMSO, 25°C, TMS): δ = 10.32 (s, 1H, Ar-N*H*-COR), 8.38 (d, *J* = 1.9 Hz, 1H, Ar-H_7), 7.98 (t, *J* = 2.0 Hz, 1H, Ar-*H*), 7.90 (dd, J_1 = 8.5 Hz, J_2 = 1.9 Hz, 1H, Ar-H_5), 7.71 (ddd, J_1 = 8.1 Hz, J_2 = 2.0 Hz, J_3 = 0.9 Hz, 1H, Ar-*H*), 7.52 (d, *J* = 8.5 Hz, 1H, Ar-H_4), 7.38 (t, *J* = 8.1 Hz, 1H, Ar-*H*), 7.14 (ddd, J_1 = 8.1 Hz, J_2 = 2.0 Hz, J_3 = 0.9 Hz, 1H, Ar-*H*), 3.58 – 3.51 (m, 4H, 2 × piperazine-CH_2), 2.85 – 2.79 ppm (m, 4H, 2 × piperazine-CH_2), not visible (N*H*)

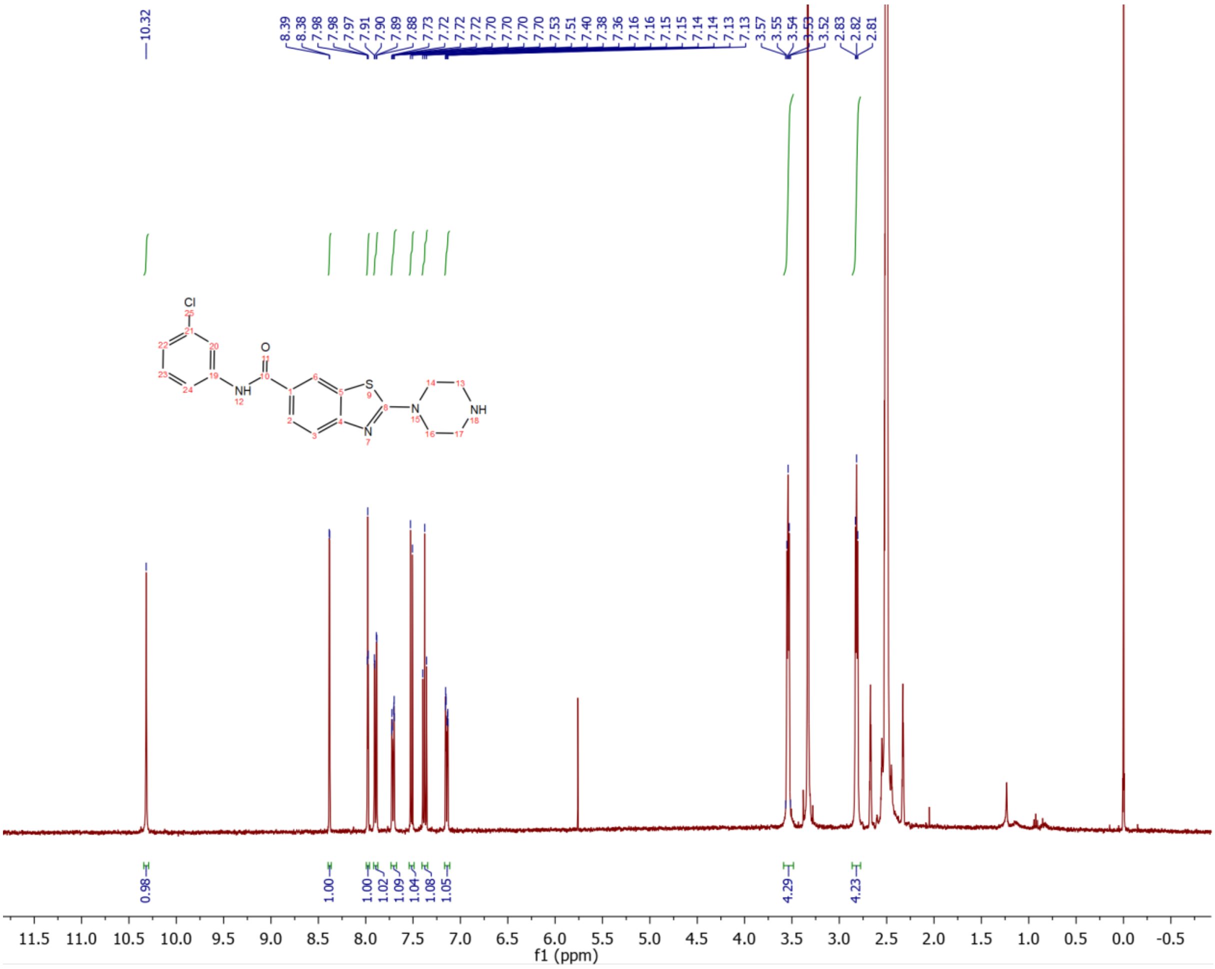

Figure S11. ^{1}H NMR spectrum of compound **5b**.

^{13}C (101 MHz, [D$_6$]DMSO, 25°C, TMS): δ = 170.7, 165.8, 155.9, 141.4, 133.4, 130.8, 130.6, 127.3, 126.6, 123.5, 121.7, 120.0, 119.0, 118.1, 49.9 (2C), 45.5 ppm (2C)

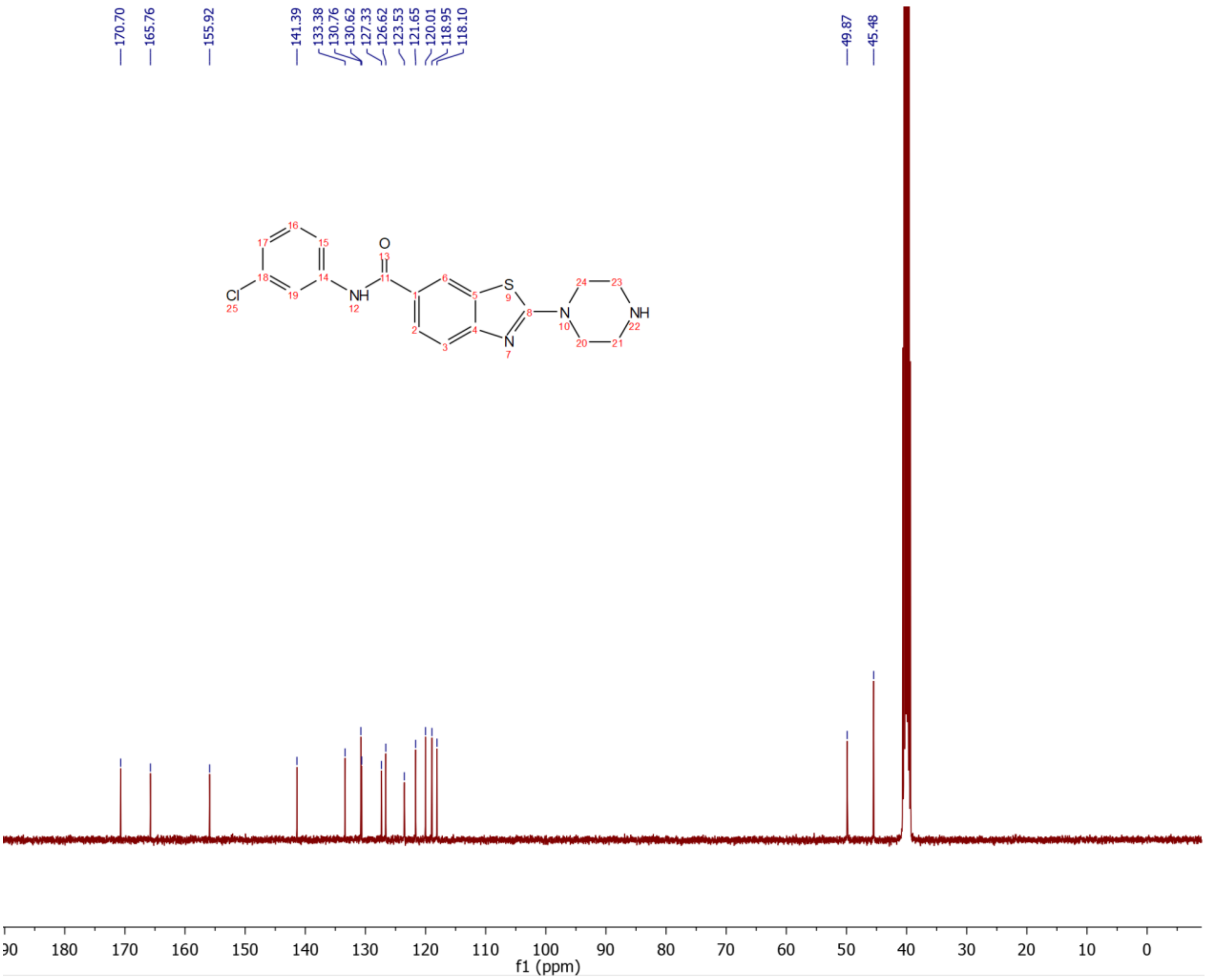

Figure S12. ^{13}C NMR spectrum of compound **5b**.

HPLC: t_r = 5.31 min (95.5 % at 254 nm)

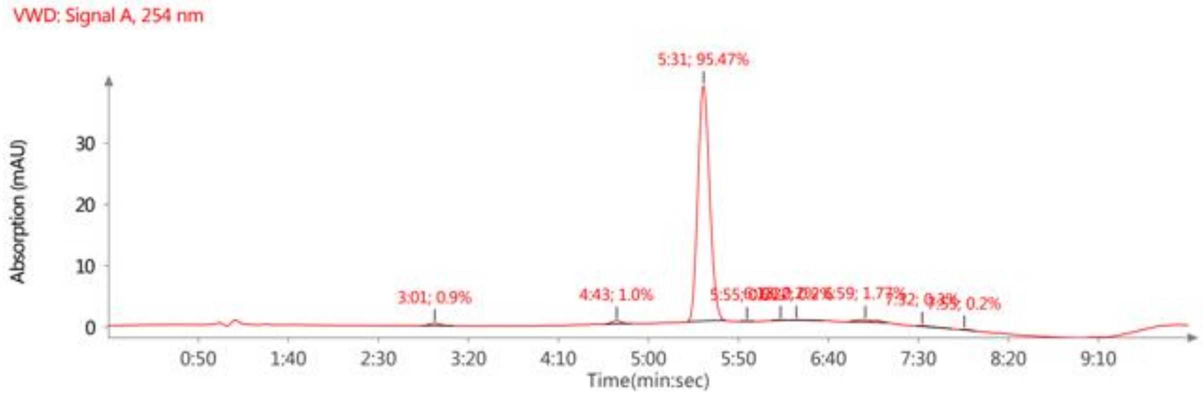

Figure S13. HPLC chromatogram of compound **5b**.

^{1}H NMR (400 MHz, [D$_6$]DMSO, 25°C, TMS): δ = 10.29 (s, 1H, Ar-N*H*-COR), 8.38 (d, *J* = 1.8 Hz, 1H, Ar-*H*$_7$), 7.89 (dd, J_1 = 8.5, J_2 = 1.8 Hz, 1H, Ar-*H*$_5$), 7.85 – 7.78 (m, 2H, 2 × Ar-*H*), 7.51 (d, *J* = 8.5 Hz, 1H, Ar-*H*$_4$), 7.44 – 7.37 (m, 2H, 2 × Ar-*H*), 3.57 – 3.50 (m, 4H, 2 × piperazine-*C*H_2), 2.84 – 2.78 ppm (m, 4H, 2 × piperazine-*C*H_2), not visible (N*H*);

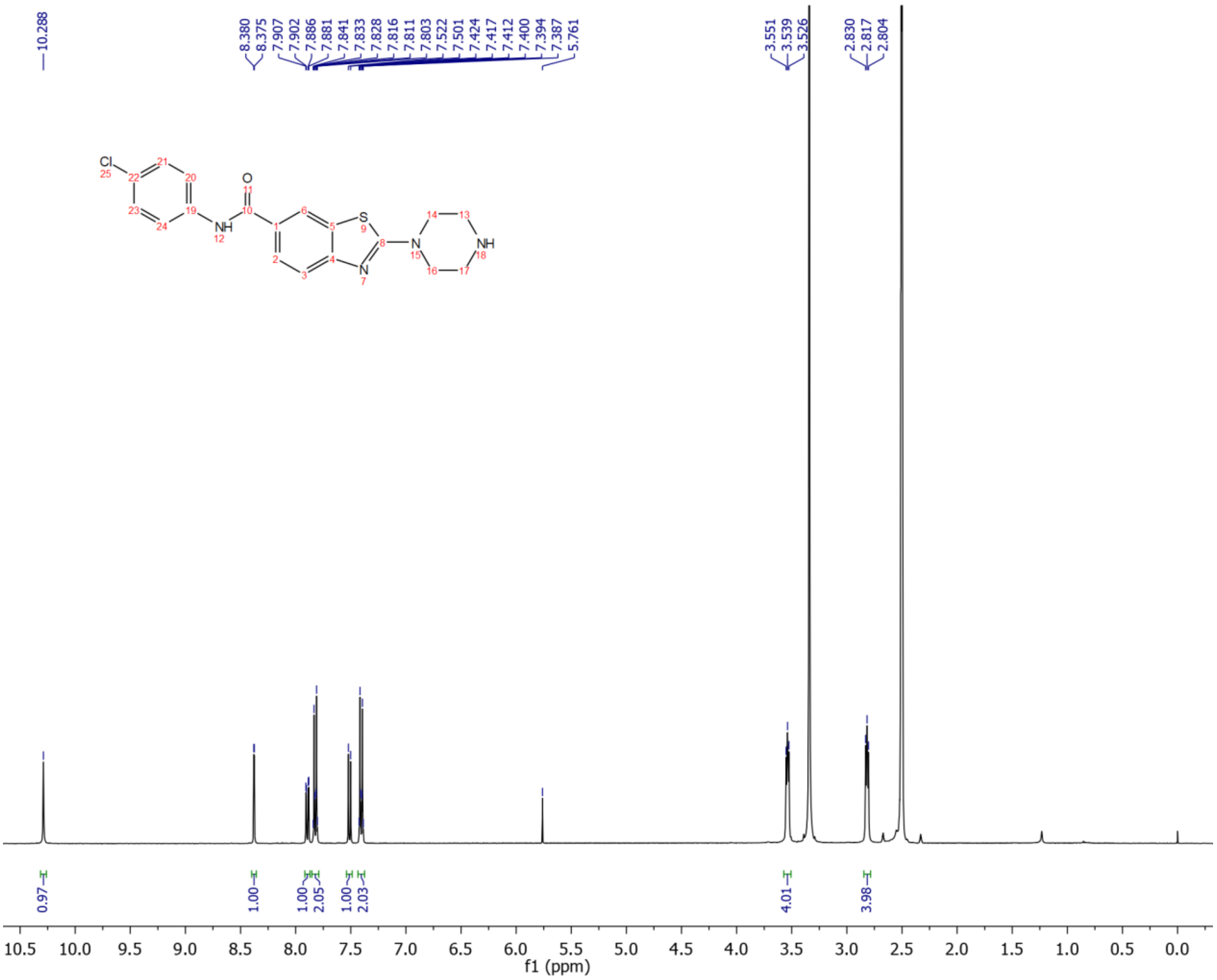

Figure S14. ^{1}H NMR spectrum of compound **5c**.

^{13}C (101 MHz, [D$_6$]DMSO, 25°C, TMS): δ = 170.7, 165.6, 155.8, 138.9, 130.6, 129.0 (2C), 127.5, 127.4, 126.6, 122.2 (2C),

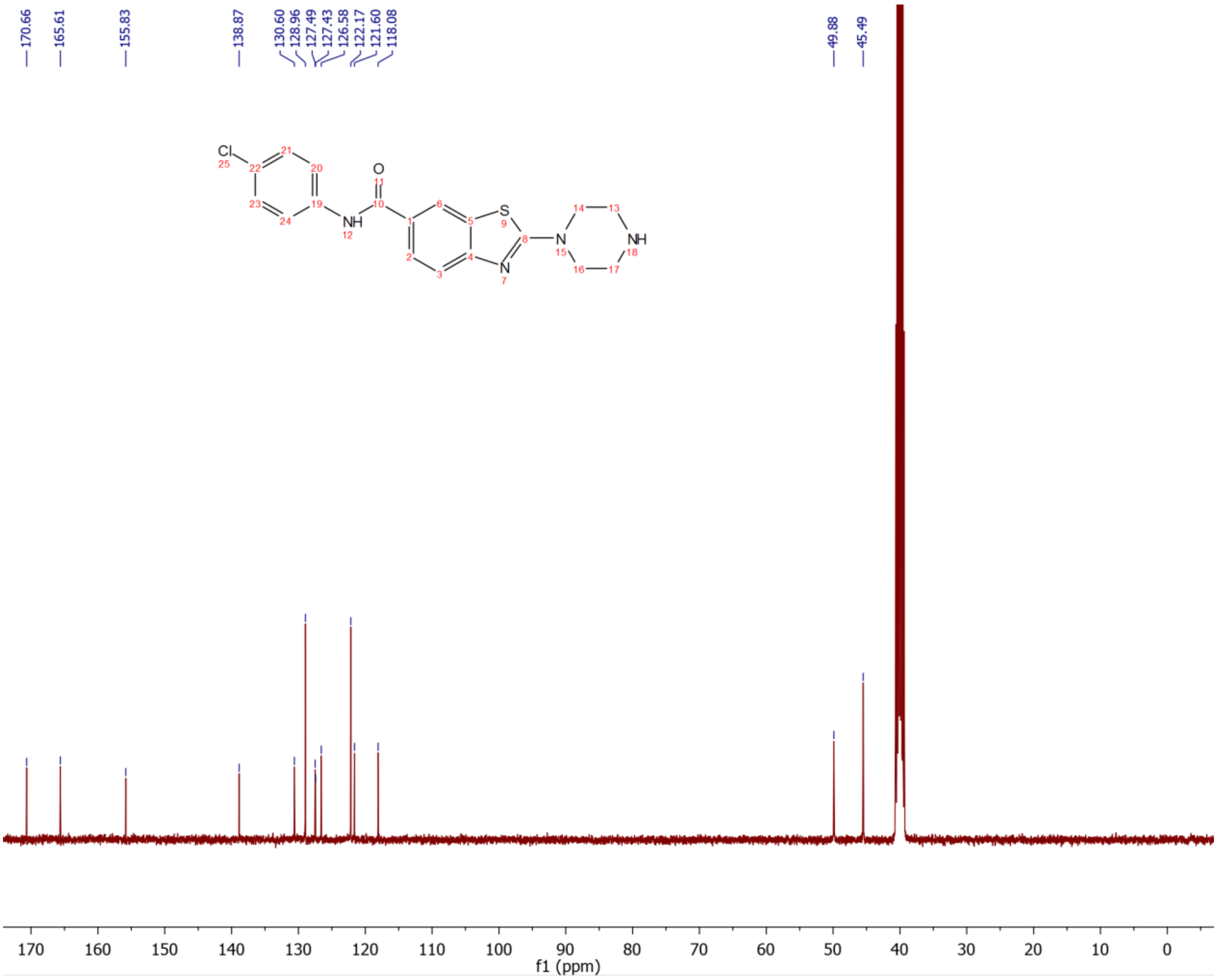

Figure S15. ^{13}C NMR spectrum of compound **5c**.

HPLC: t_r = 5.27 min (97.2 % at 254 nm)

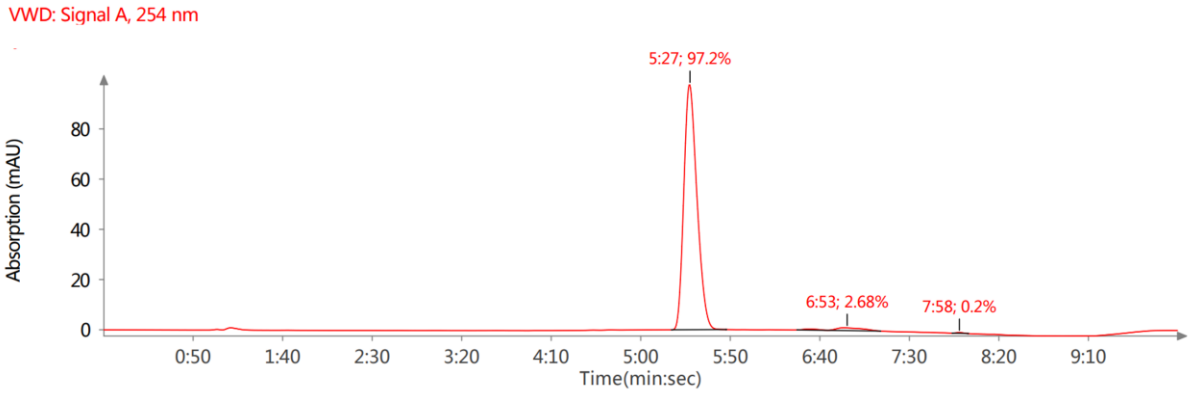

Figure S16. HPLC chromatogram of compound **5c**.

^{1}H NMR (400 MHz, $CDCl_3$, 25°C, TMS): δ = 8.20 (d, J = 1.9 Hz, 1H, Ar-H_7), 7.90 (d, J = 2.4 Hz, 1H, Ar-H_{20}), 7.79 (s, 1H, Ar-NH-COR), 7.73 (dd, J_1 = 8.5 Hz, J_2 = 1.9 Hz, 1H, Ar-H_5), 7.57 (d, J = 8.5 Hz, 1H, Ar-H_4), 7.48 (dd, J_1 = 8.7 Hz, J_2 = 2.4 Hz, 1H, Ar-H_{24}), 7.42 (d, J = 8.7 Hz, 1H, Ar-H_{23}), 3.70 – 3.65 (m, 4H, 2 × piperazine-CH_2), 3.06 – 2.99 ppm (m, 4H, 2 × piperazine-CH_2), not visible (NH)

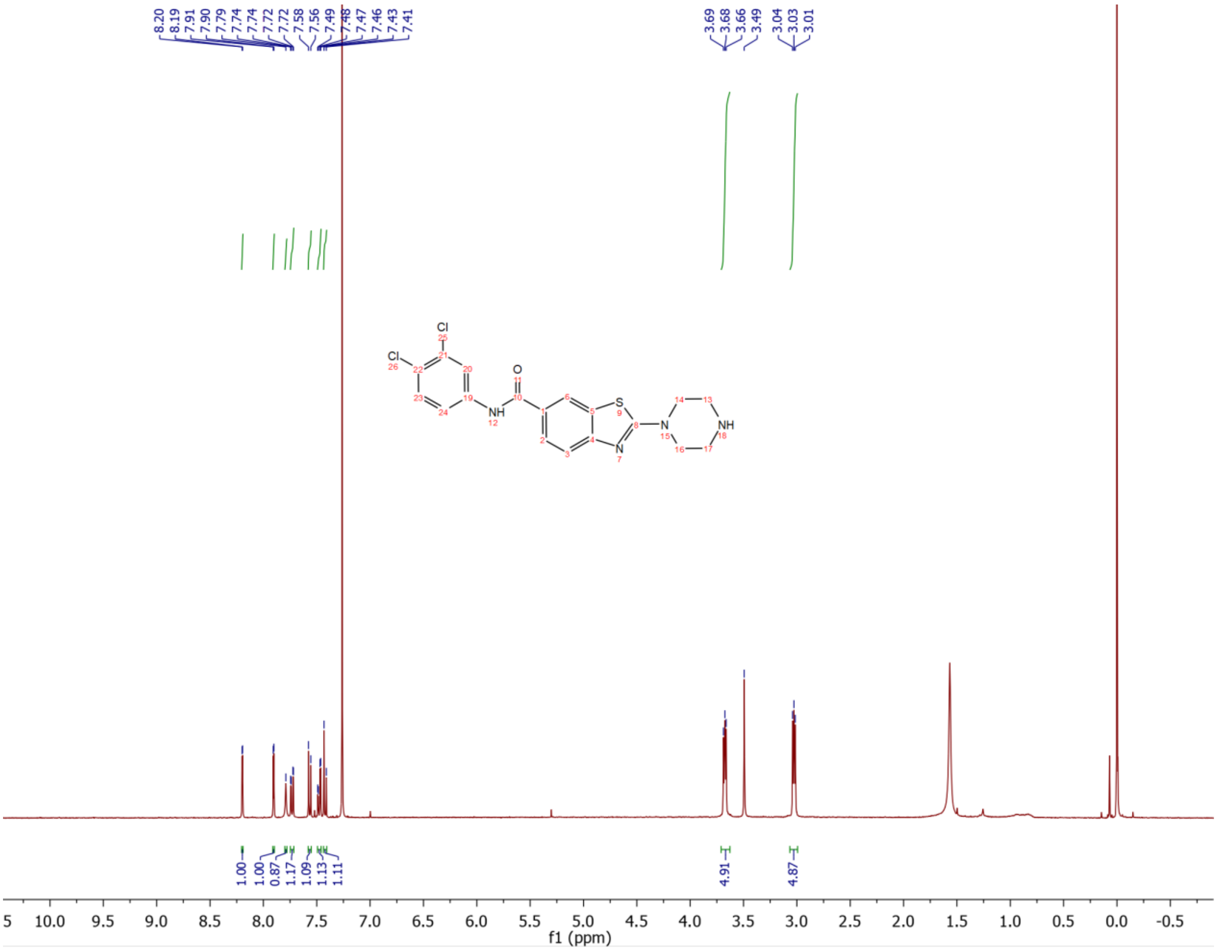

Figure S17. ^{1}H NMR spectrum of compound **5d**.

^{13}C NMR (101 MHz, [D$_6$]DMSO, 25°C, TMS): δ = 170.8, 165.8, 156.0, 140.1, 131.3, 131.0, 130.7, 127.1, 126.7, 125.2, 121.7,

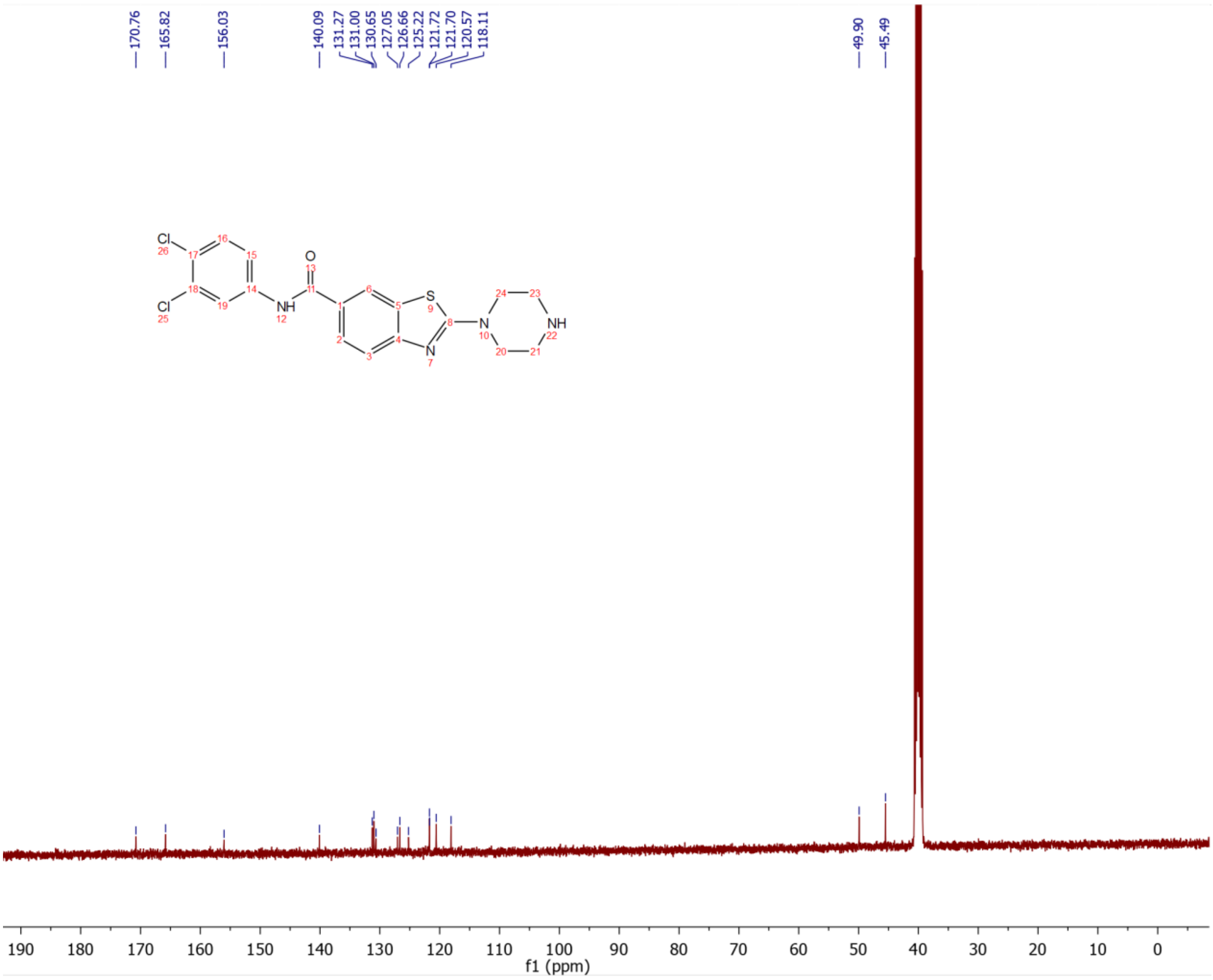

Figure S18. ^{13}C NMR spectrum of compound **5d**.

HPLC: t_r = 6.57 min (95.3 % at 254 nm)

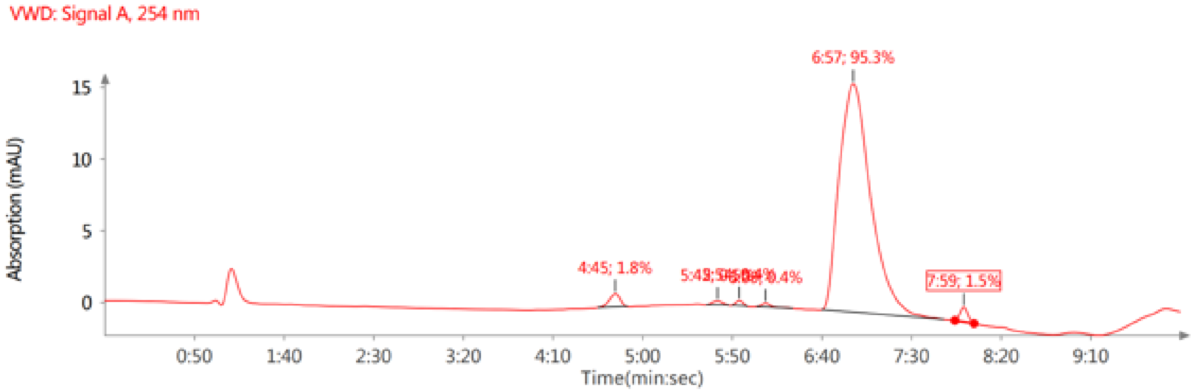

Figure S19. HPLC chromatogram of compound **5d**.

^{1}H NMR (400 MHz, [D_6]DMSO, 25°C, TMS): δ = 10.22 (s, 1H, Ar-N*H*-COR), 8.38 (d, *J* = 1.8 Hz, 1H, Ar-H_7), 7.90 (dd, J_1 = 8.5 Hz, J_2 = 1.8 Hz, 1H, Ar-H_5), 7.84 – 7.74 (m, 2H, 2 × Ar-*H*), 7.52 (d, *J* = 8.5 Hz, 1H, Ar-H_4), 7.24 – 7.14 (m, 2H, 2 × Ar-*H*), 3.59 – 3.50 (m, 4H, 2 × piperazine-CH_2), 2.83 ppm (m, 4.3 Hz, 4H, 2 × piperazine-CH_2), not visible (N*H*)

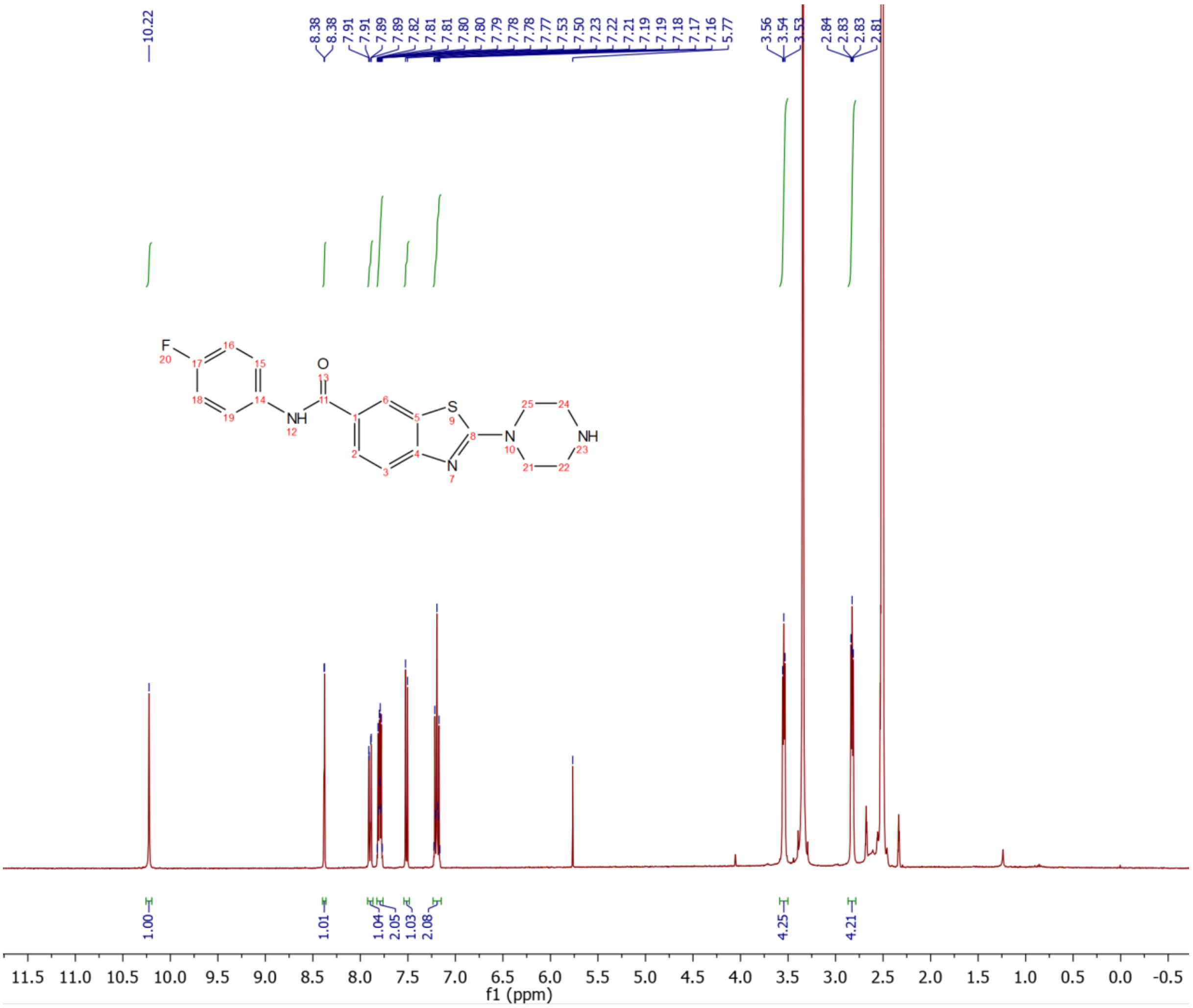

Figure S20. ^{1}H NMR spectrum of compound **5e**.

^{19}F NMR (376 MHz, [D$_6$]DMSO, 25°C, TMS): δ = -119.26 ppm

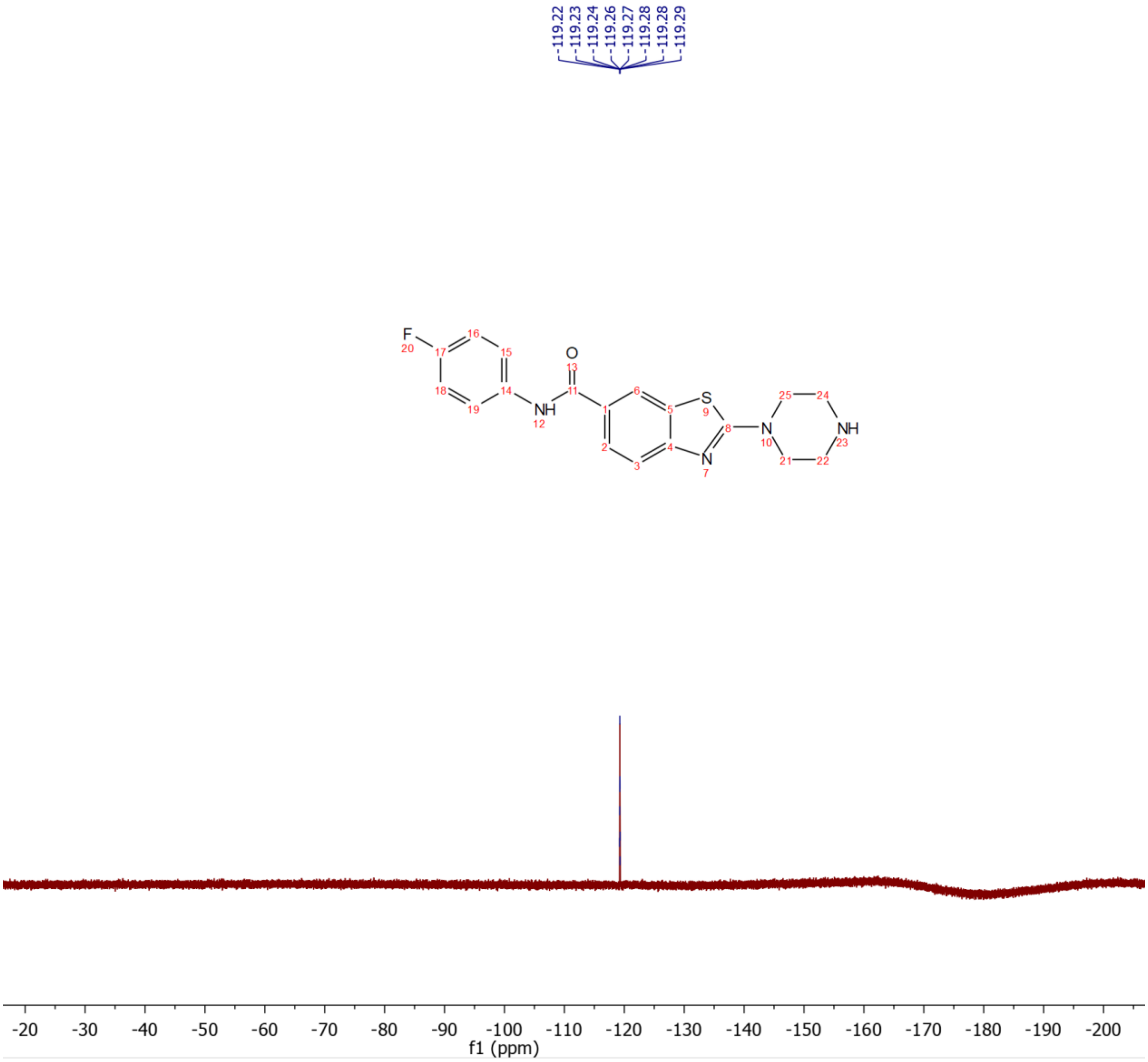

Figure S21. ^{19}F NMR spectrum of compound **5e**.

^{13}C (101 MHz, [D$_6$]DMSO, 25°C, TMS): δ = 170.6, 165.4, 158.60 (d, *J* = 239.8 Hz), 155.7, 136.2 (d, *J* = 2.6 Hz, 2C), 130.6, 127.6, 126.5, 122.5, 122.4, 121.5, 118.1, 115.7, 115.5, 49.9 (2C), 45.5 ppm (2C)

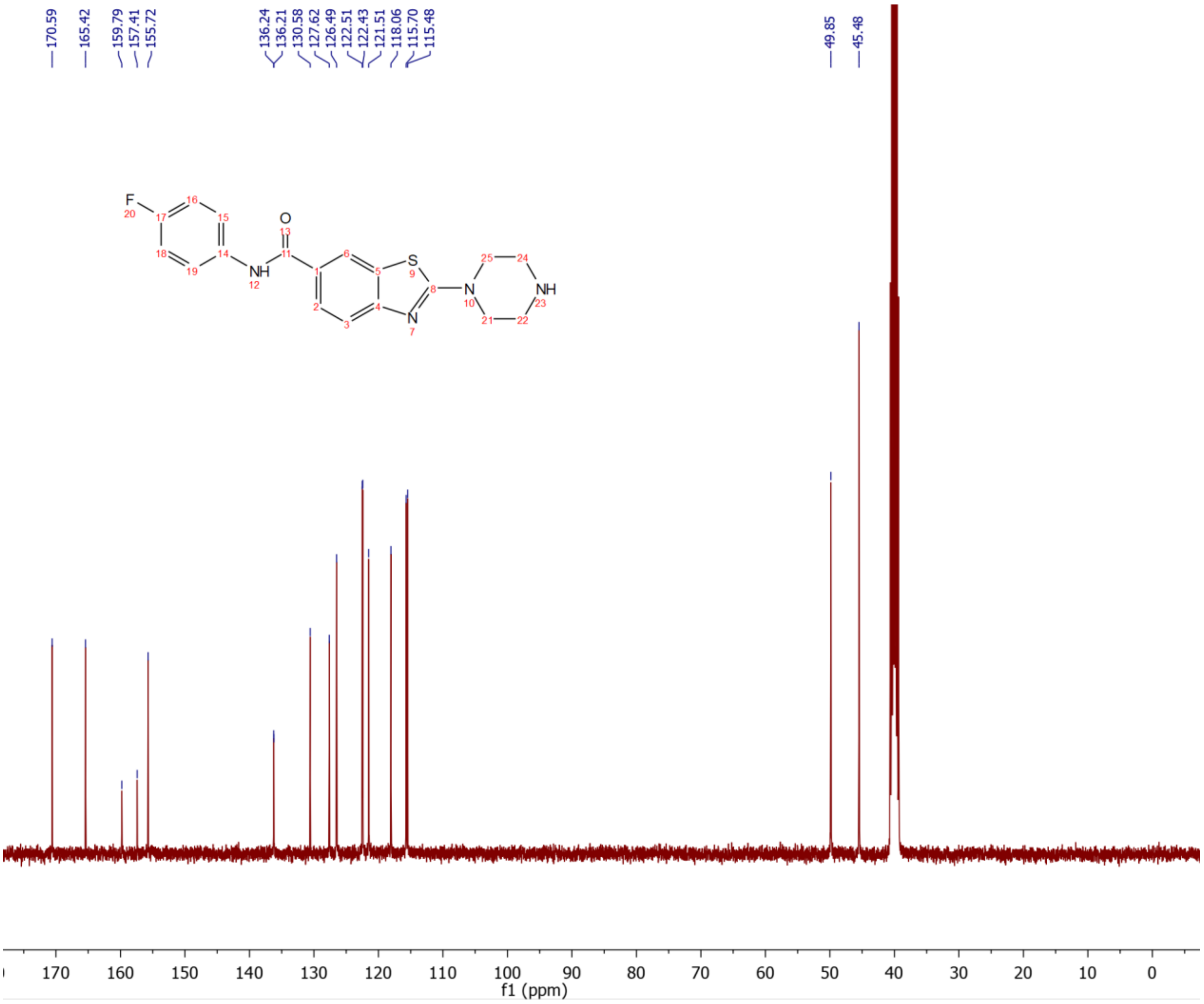

Figure S22. ^{13}C NMR spectrum of compound **5e**.

HPLC: t_r = 3.27 min (99,0 % at 254 nm)

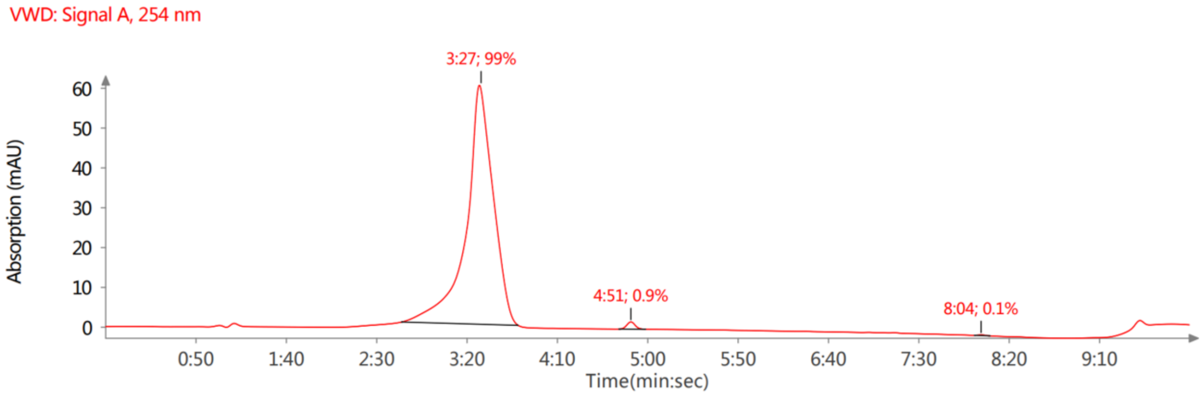

Figure S23. HPLC chromatogram of compound **5e**.

^{1}H NMR (400 MHz, [D6]DMSO, 25°C, TMS): δ = 10.28 (s, 1H, Ar-N*H*-COR), 8.38 (d, *J* = 1.8 Hz, 1H, Ar-*H*7), 7.89 (dd, J_1 = 8.5 Hz, J_2 = 1.8 Hz, 1H, Ar-*H*5), 7.79 – 7.73 (m, 2H, 2 × Ar-*H*), 7.56 – 7.47 (m, 3H, 3 × Ar-*H*), 3.59 – 3.48 (m, 4H, 2 × piperazine-C*H*2), 2.86 – 2.77 ppm (m, 4H, 2 × piperazine-C*H*2), not visible (N*H*)

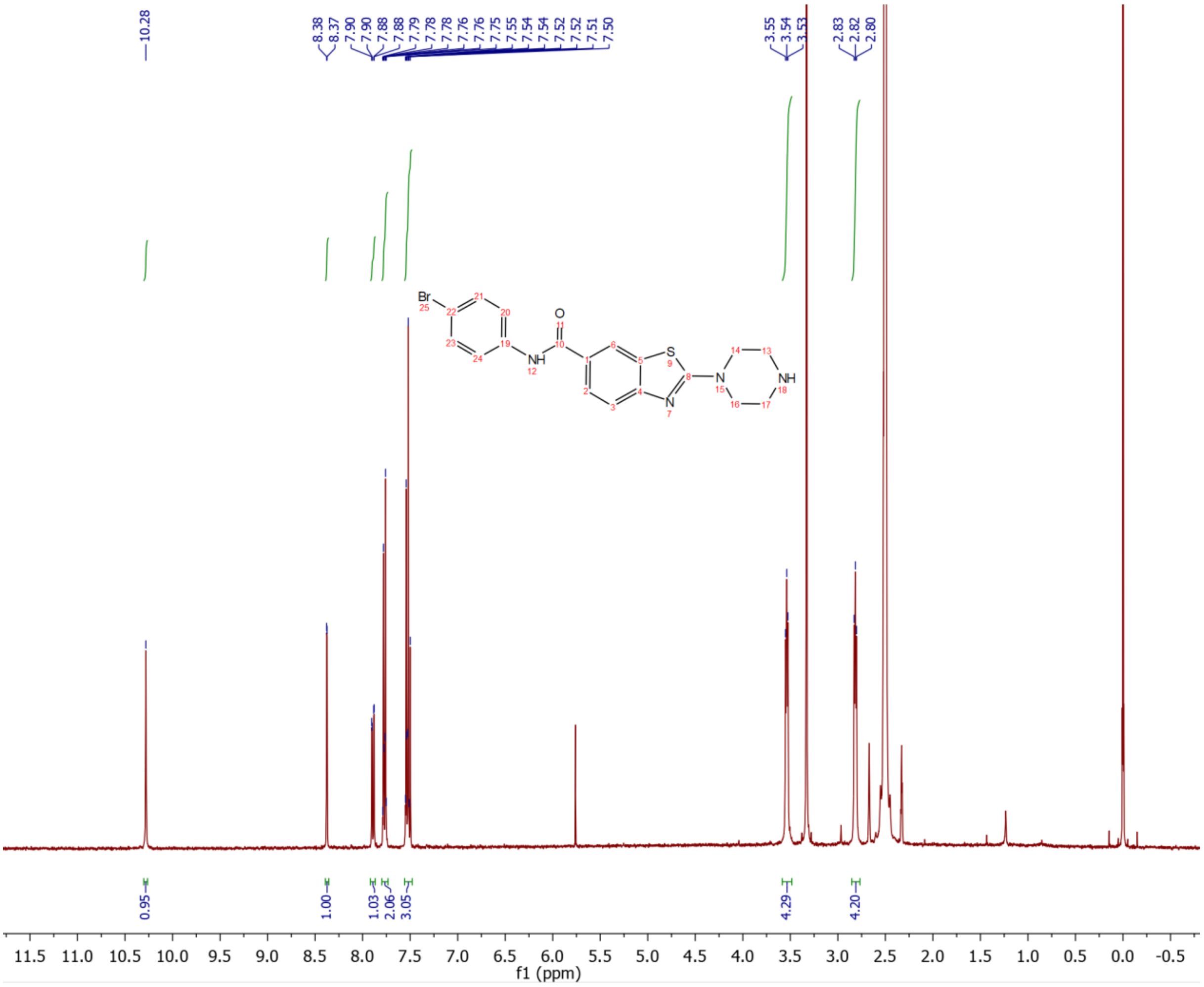

Figure S24. ^{1}H NMR spectrum of compound **5f**.

^{13}C (101 MHz, [D$_6$]DMSO, 25°C, TMS): δ = 170.7, 165.6, 155.8, 139.3, 131.9 (2C), 130.6, 127.5, 126.6, 122.6 (2C), 121.6, 118.1, 115.5, 49.9 (2C), 45.5 ppm (2C)

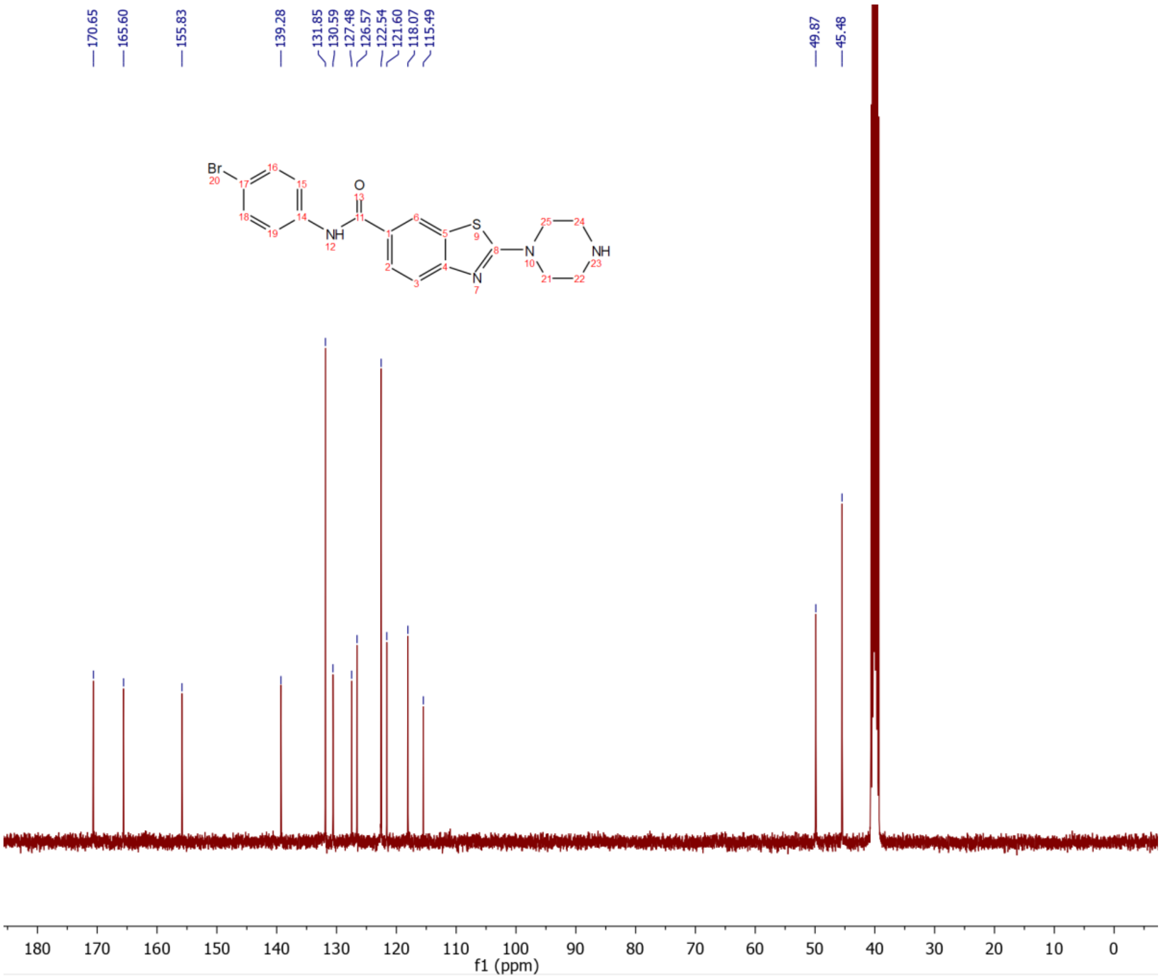

Figure S25. ^{13}C NMR spectrum of compound **5f**.

HPLC: t_r = 5.45 min (96.5 % at 254 nm)

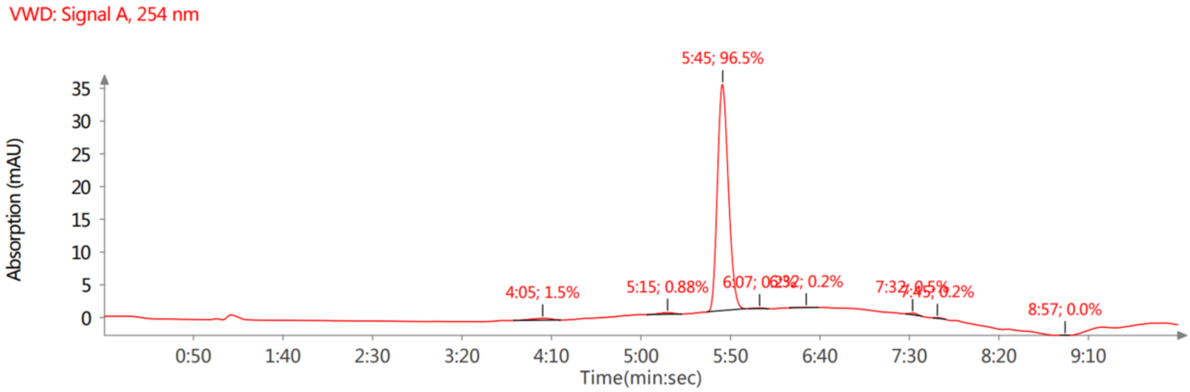

Figure S26. HPLC chromatogram of compound **5f**.

^{1}H NMR (400 MHz, [D$_6$]DMSO, 25°C, TMS): δ = 10.25 (s, 1H, Ar-N*H*-COR), 8.37 (s, 1H, Ar-*H*), 7.89 (d, *J* = 8.5 Hz, 1H, Ar-*H*), 7.71 – 7.60 (m, 4H, 4 × Ar-*H*), 7.50 (d, *J* = 8.1 Hz, 1H, Ar-*H*), 3.57 – 3.51 (m, 4H, 2 × piperazine-CH_2), 2.84 – 2.79 ppm (m, 4H, 2 × piperazine-CH_2), not visible (N*H*)

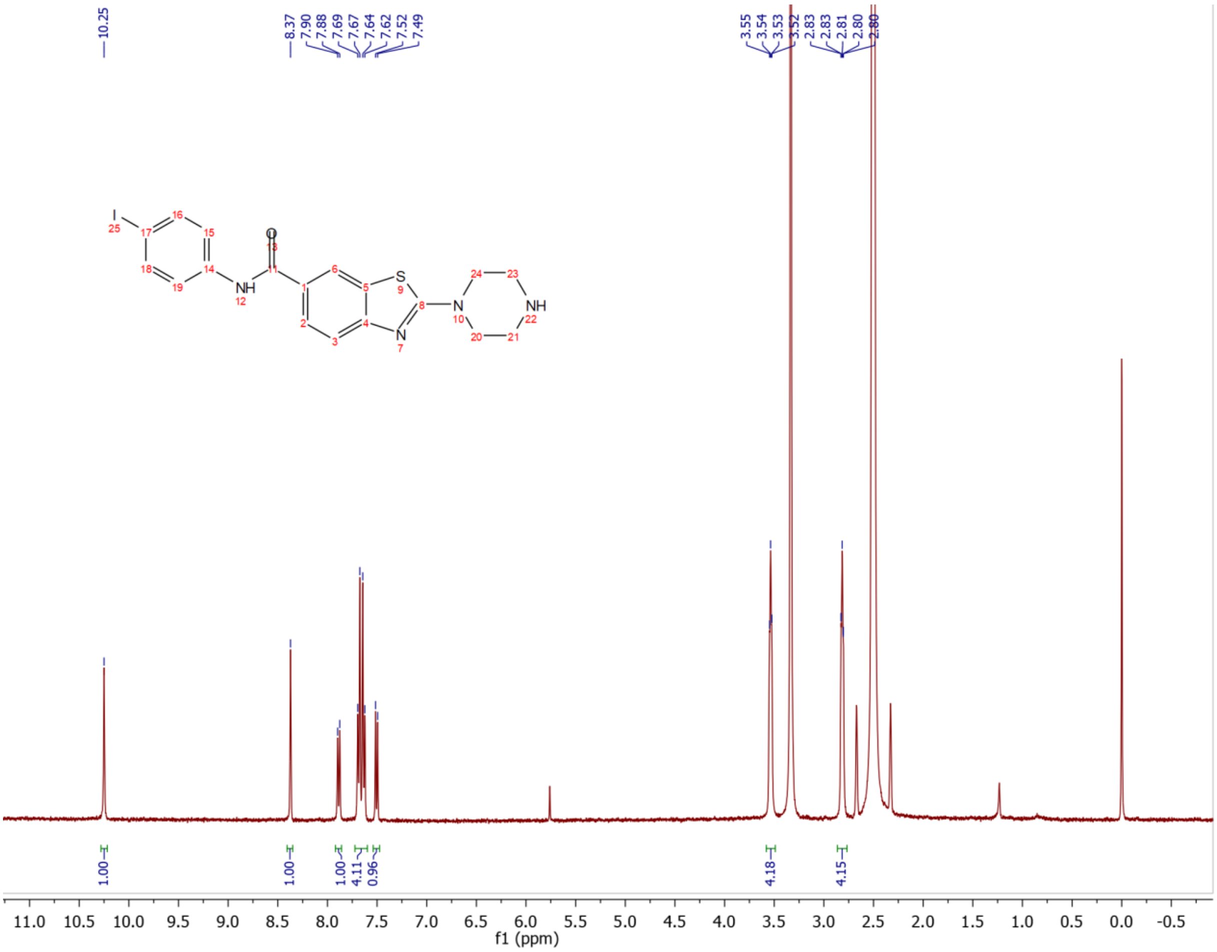

Figure S27. ^{1}H NMR spectrum of compound **5g**.

^{13}C (101 MHz, [D$_6$]DMSO, 25°C, TMS): δ = 170.6, 165.6, 155.8, 139.8, 137.7 (2C), 130.6, 127.5, 126.6, 122.8 (2C), 121.6, 118.1, 87.4, 49.8 (2C), 45.5 ppm (2C)

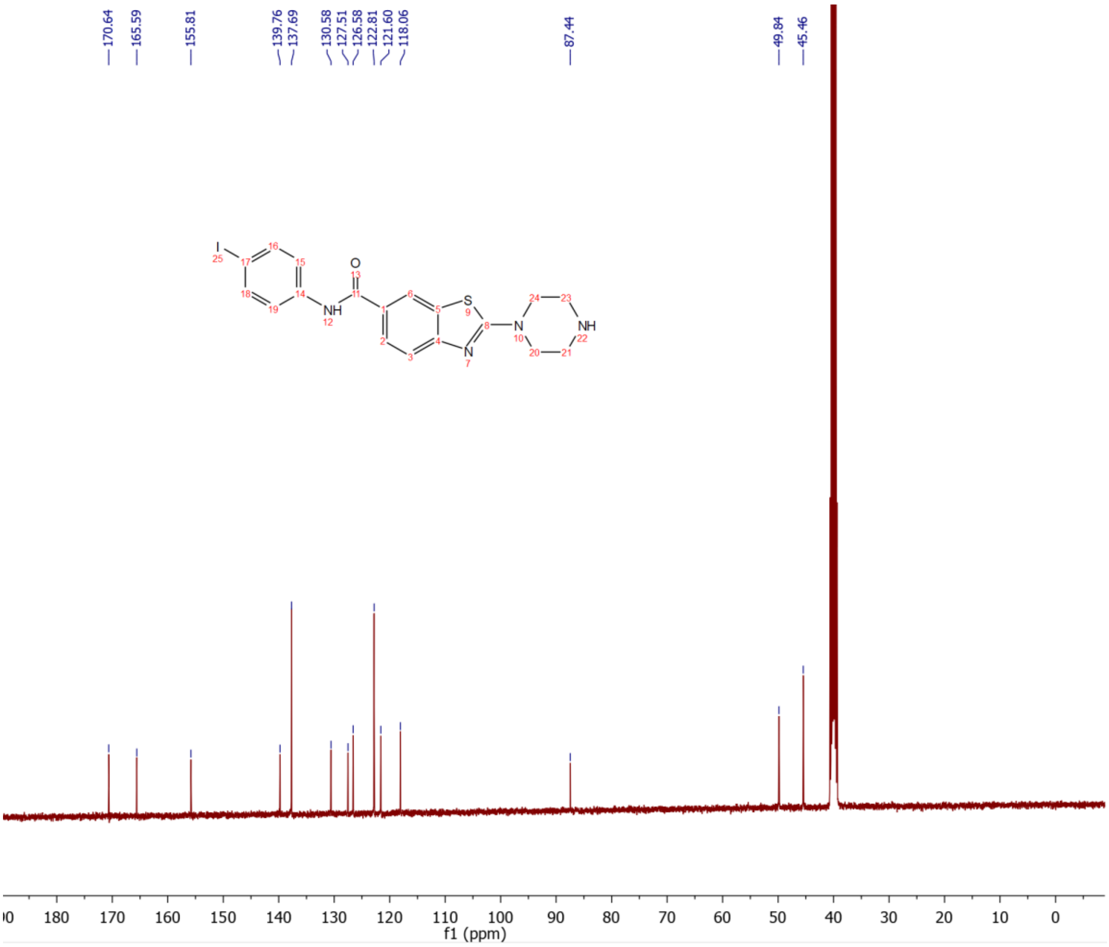

Figure S28. ^{13}C NMR spectrum of compound **5g**.

HPLC: t_r = 5.03 min (98.7 % at 254 nm)

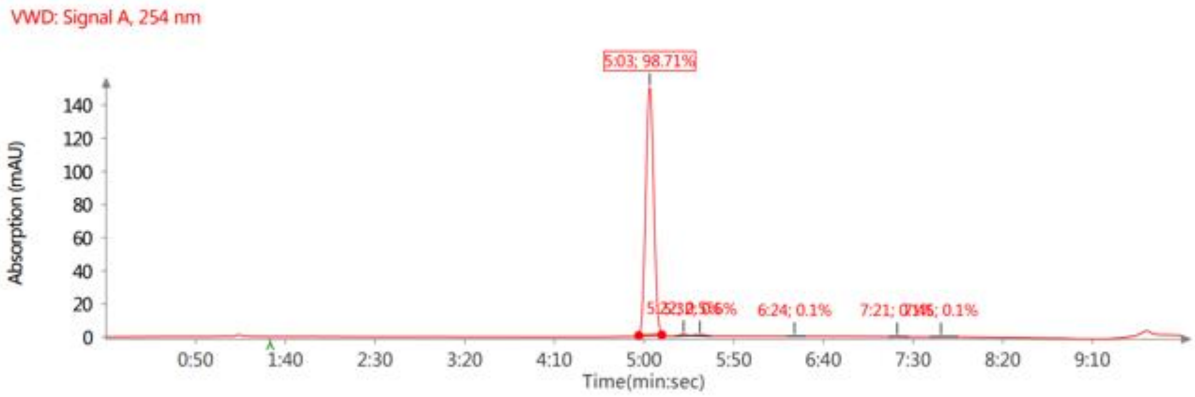

Figure S29. HPLC chromatogram of compound **5g**.

^{1}H NMR (400 MHz, [D$_6$]DMSO, 25°C, TMS): δ = 10.08 (s, 1H, Ar-N*H*-COR), 8.37 (d, *J* = 1.8 Hz, 1H, Ar-*H*$_7$), 7.89 (dd, J_1 = 8.5 Hz, J_2 = 1.8 Hz, 1H, Ar-*H*$_5$), 7.68 (d, *J* = 8.6 Hz, 2H, 2 × Ar-*H*), 7.50 (d, *J* = 8.5 Hz, 1H, Ar-*H*$_4$), 7.21 (d, *J* = 8.6 Hz, 2H, 2 × Ar-*H*), 3.58 – 3.47 (m, 4H, 2 × piperazine-C*H*$_2$), 2.90 – 2.78 (m, 5H, Ar-C*H*-(CH$_3$)$_2$ + 2 × piperazine-C*H*$_2$), 1.20 ppm (d, *J* = 6.9 Hz, 6H, Ar-CH-(C*H*$_3$)$_2$)

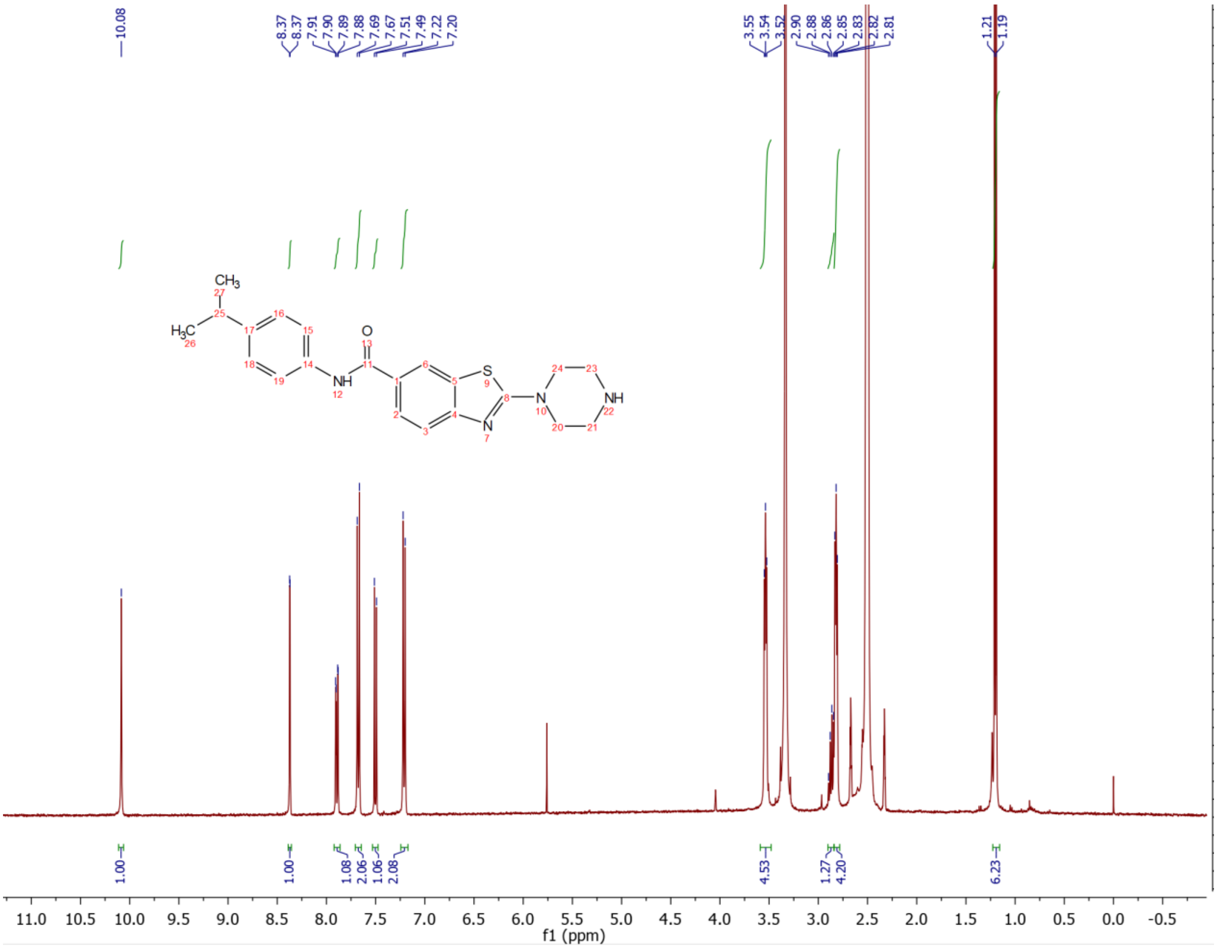

Figure S30. ^{1}H NMR spectrum of compound **5h**.

^{13}C (101 MHz, [D6]DMSO, 25°C, TMS): δ = 170.5, 165.3, 155.6, 143.9, 137.6, 130.5, 127.9, 126.7 (2C), 126.5, 121.5, 120.8 (2C), 118.0, 49.9 (2C), 45.5 (2C), 33.4, 24.5 ppm (2C)

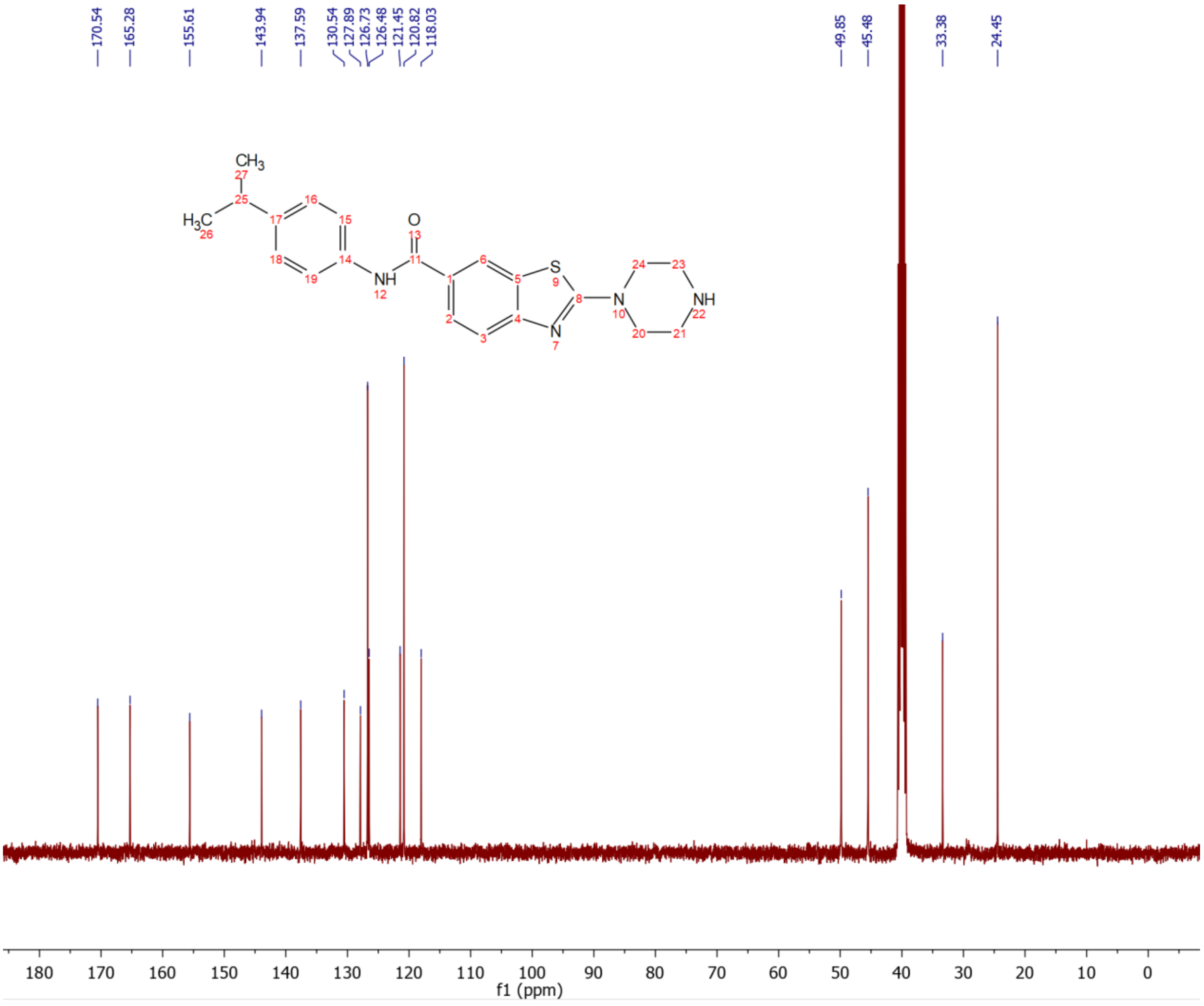

Figure S31. ^{13}C NMR spectrum of compound **5h**.

HPLC: t_r = 5.12 min (99,0 % at 254 nm)

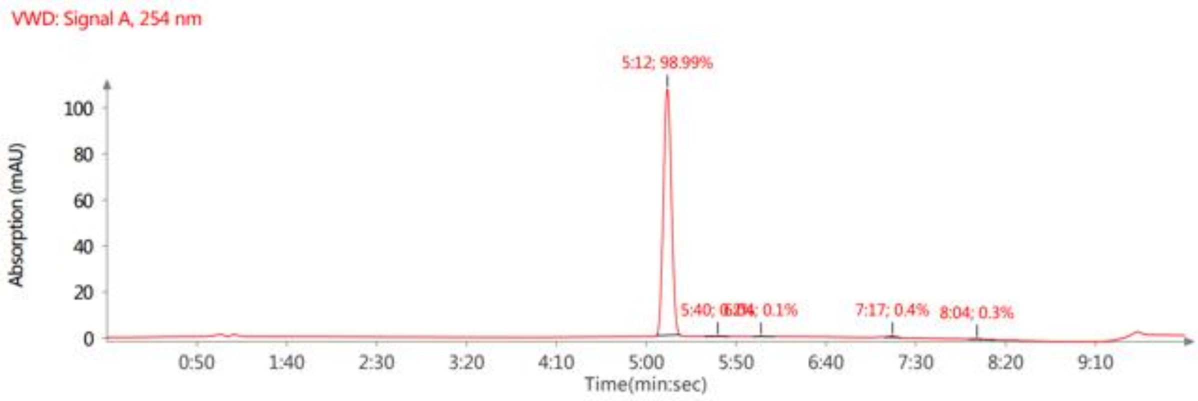

Figure S32. HPLC chromatogram of compound **5h**.

^{1}H NMR (400 MHz, [D$_6$]DMSO, 25°C, TMS): δ = 10.04 (s, 1H, Ar-N*H*-COR), 8.36 (d, *J* = 1.8 Hz, 1H, Ar-*H$_7$*), 7.89 (dd, *J$_1$* = 8.5 Hz, *J$_2$* = 1.8 Hz, 1H, Ar-*H$_5$*), 7.71 – 7.63 (m, 2H, 2 × Ar-*H*), 7.50 (d, *J* = 8.5 Hz, 1H, Ar-*H$_4$*), 6.95 – 6.89 (m, 2H, 2 × Ar-*H*), 3.74 (s, 3H, Ar-O-C*H$_3$*), 3.58 – 3.48 (m, 4H, 2 × piperazine-C*H$_2$*), 2.85 – 2.78 ppm (m, 4H, 2 × piperazine-C*H$_2$*), not visible (N*H*)

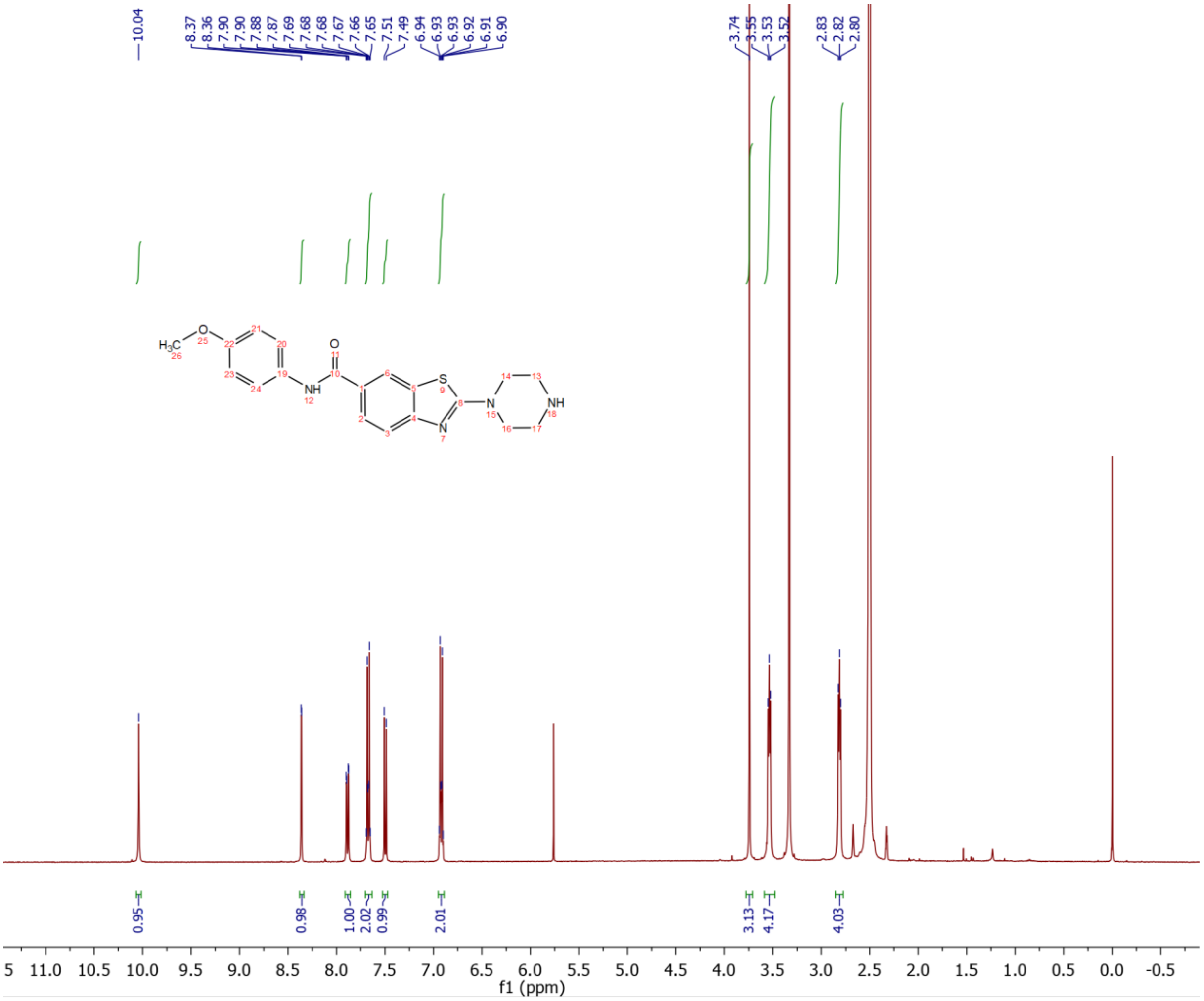

Figure S33. ^{1}H NMR spectrum of compound **5i**.

^{13}C (101 MHz, [D$_6$]DMSO, 25°C, TMS): δ = 170.5, 165.1, 155.9, 155.6, 132.9, 130.6, 128.0, 125.9, 122.3 (2C), 121.4, 118.0, 114.2 (2C), 55.6, 49.9 (2C), 45.5 ppm (2C)

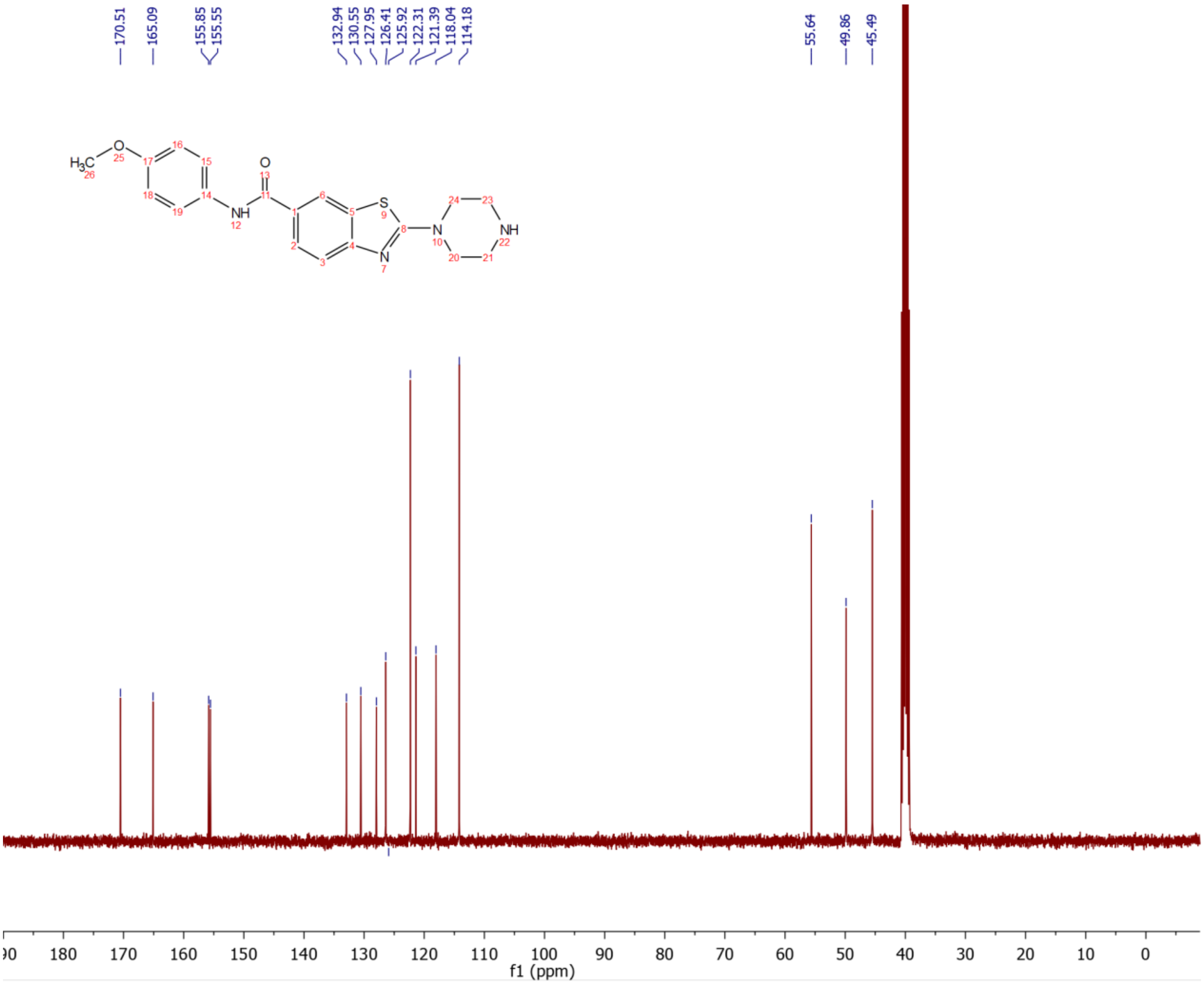

Figure S34. ^{13}C NMR spectrum of compound **5i**.

HPLC: t_r = 3.42 min (95.0 % at 254 nm)

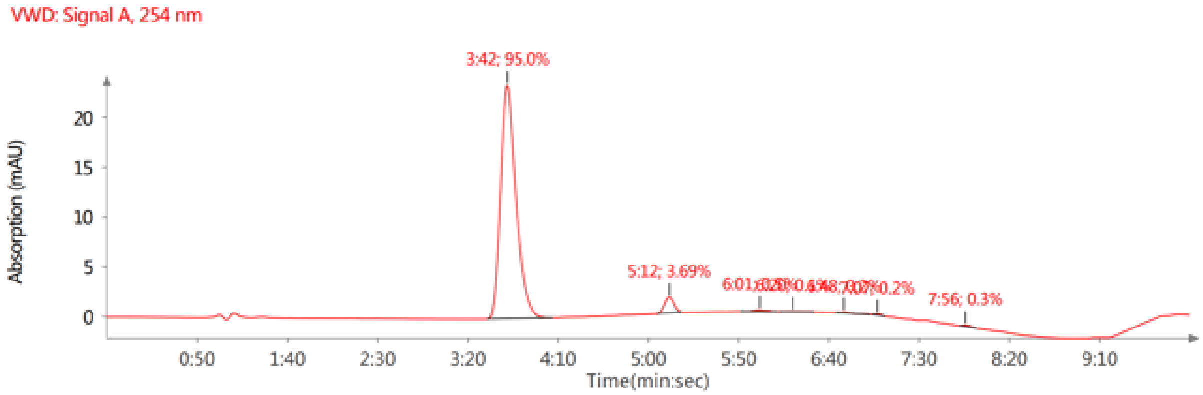

Figure S35. HPLC chromatogram of compound **5i**.

^{1}H NMR (400 MHz, [D_6]DMSO, 25°C, TMS): δ = 8.95 (t, *J* = 6.0 Hz, 1H, Ar-CH_2-N*H*-COR), 8.30 (d, *J* = 1.8 Hz, 1H, Ar-H_7), 7.83 (dd, J_1 = 8.5 Hz, J_2 = 1.8 Hz, 1H, Ar-H_5), 7.45 (d, *J* = 8.5 Hz, 1H, Ar-H_4), 7.35 – 7.29 (m, 4H, 4 × Ar-*H*), 7.26 – 7.20 (m, 1H, Ar-*H*), 4.48 (d, *J* = 6.0 Hz, 2H, Ar-CH_2-NHCO), 3.56 – 3.48 (m, 4H, 2 × piperazine-CH_2), 2.84 – 2.76 ppm (m, 4H, 2 × piperazine-CH_2), not visible (N*H*)

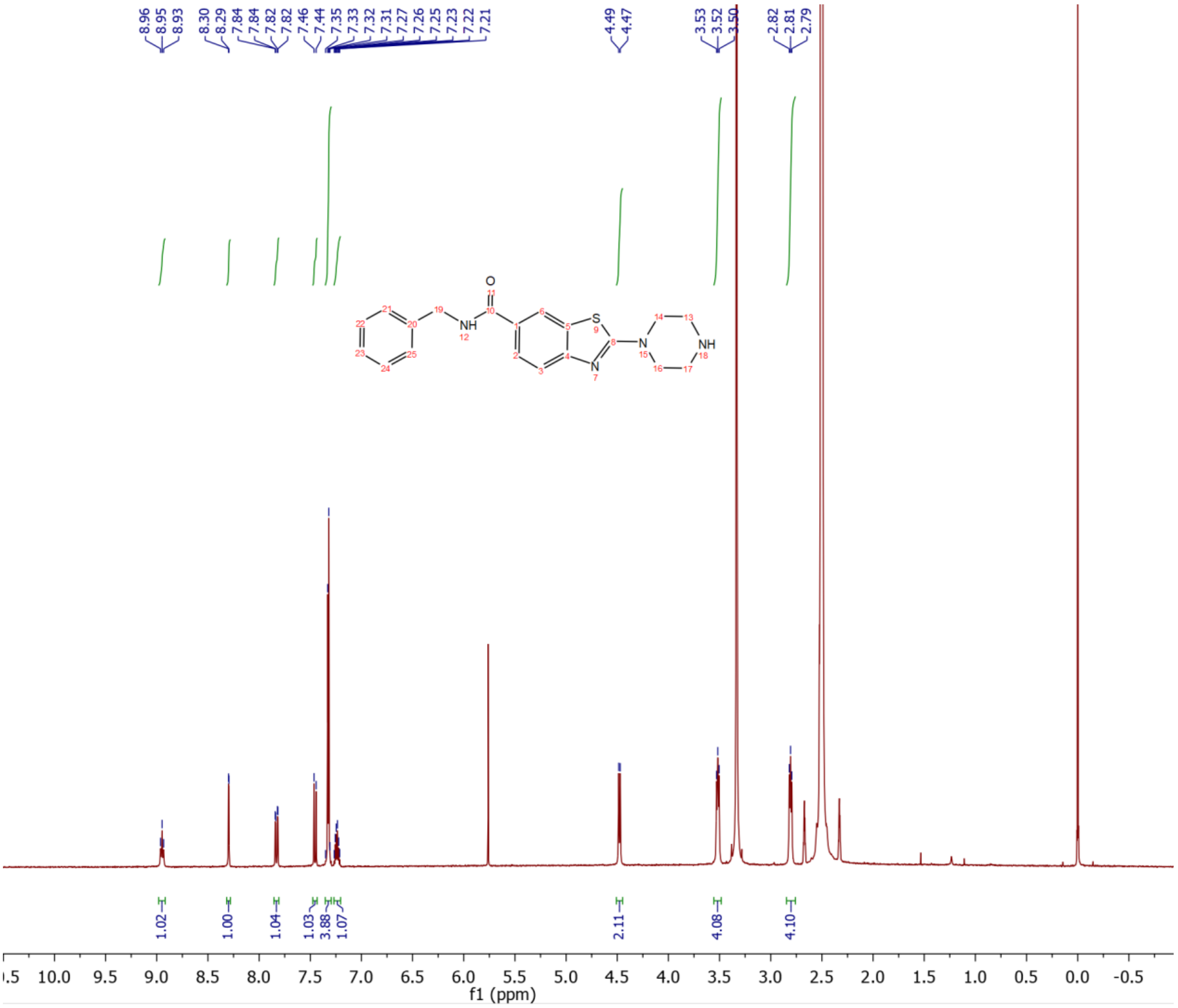

Figure S36. ^{1}H NMR spectrum of compound **5j**.

^{13}C (101 MHz, [D$_6$]DMSO, 25°C, TMS): δ = 170.3, 166.3, 155.3, 140.3, 130.6, 128.7 (2C), 127.7 (2C), 127.6, 127.2, 126.0, 121.1, 118.1, 49.3 (2C), 45.1 (2C), 43.1 ppm

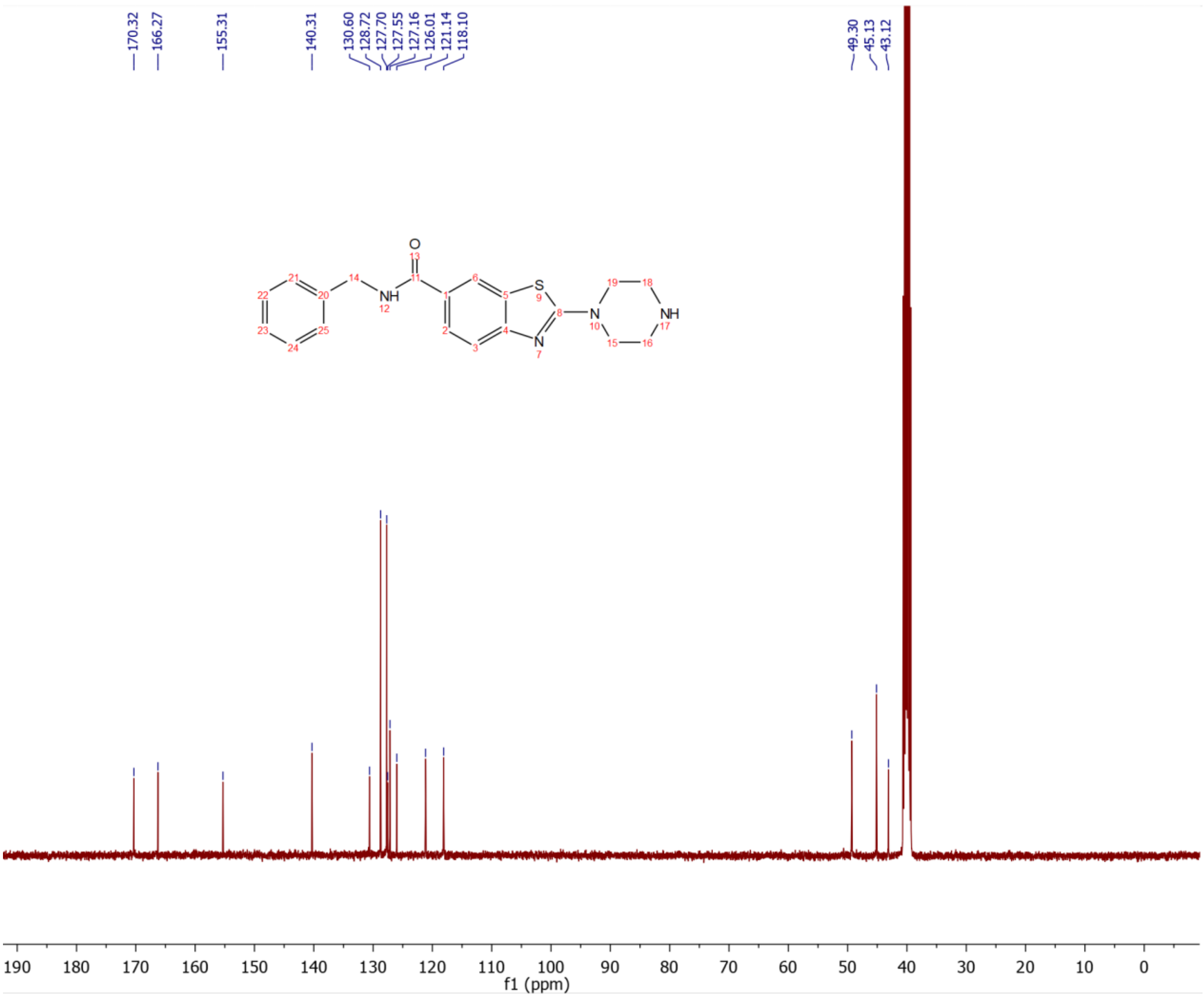

Figure S37. ^{13}C NMR spectrum of compound **5j**.

HPLC: t_r = 3.43 min (95.3 % at 254 nm)

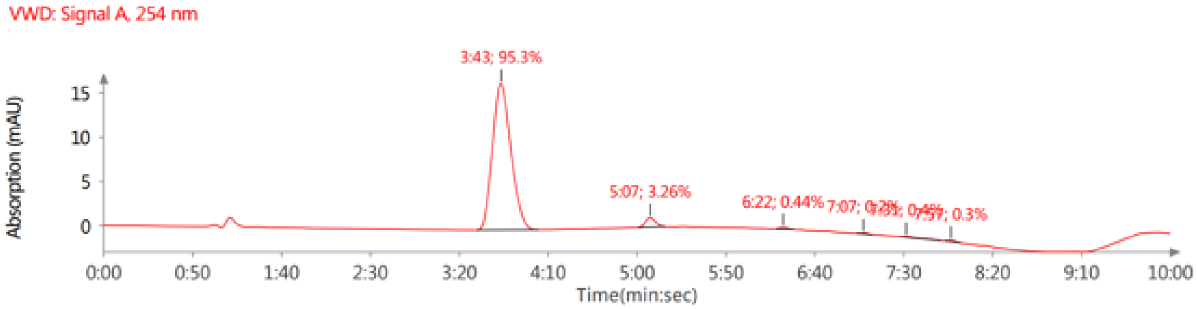

Figure S38. HPLC chromatogram of compound **5j**.

^{1}H NMR (400 MHz, [D$_6$]DMSO, 25°C, TMS): δ = 8.99 (t, *J* = 6.0 Hz, 1H, CH$_2$-N*H*-COR), 8.30 (d, *J* = 1.9 Hz, 1H, Ar-*H*$_7$), 7.82 (dd, *J*$_1$ = 8.5 Hz, *J*$_2$ = 1.9 Hz, 1H, Ar-*H*$_5$), 7.46 (d, *J* = 8.5 Hz, 1H, Ar-*H*$_4$), 7.39 – 7.33 (m, 2H, 2 × Ar-*H*), 7.33 – 7.27 (m, 2H, 2 × Ar-*H*), 4.47 (d, *J* = 6.0 Hz, 2H), 3.56 – 3.48 (m, 4H, 2 × piperazine-C*H*$_2$), 2.85 – 2.76 ppm (m, 4H, 2 × piperazine-C*H*$_2$), not visible (N*H*)

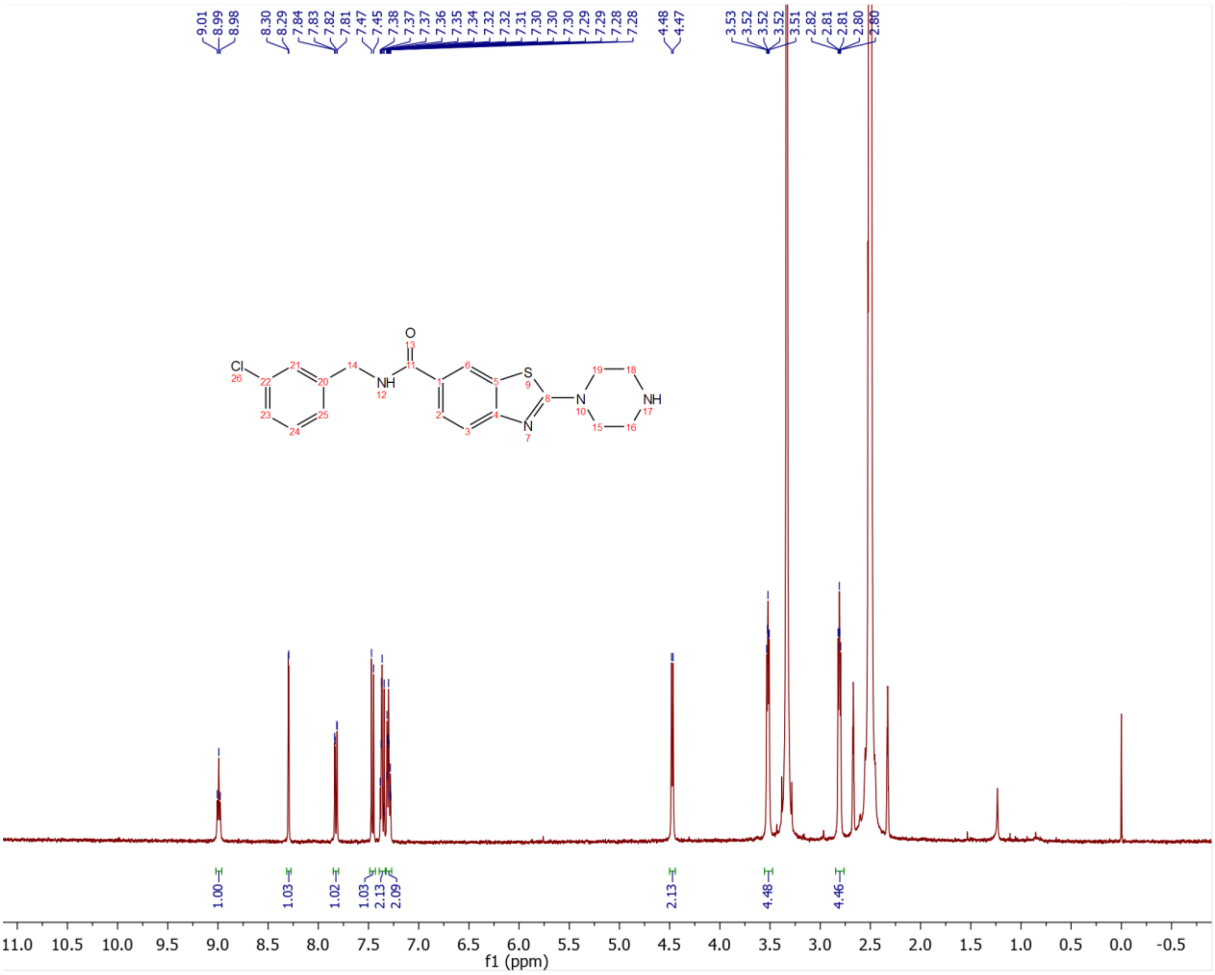

Figure S39. ^{1}H NMR spectrum of compound **5k**.

^{13}C (101 MHz, [D$_6$]DMSO, 25°C, TMS): δ = 170.4, 166.4, 155.5, 142.9, 133.4, 130.6, 130.6, 127.5, 127.2, 127.1, 126.4, 126.0, 121.1, 118.1, 49.8 (2C), 45.5 (2C), 42.7 ppm

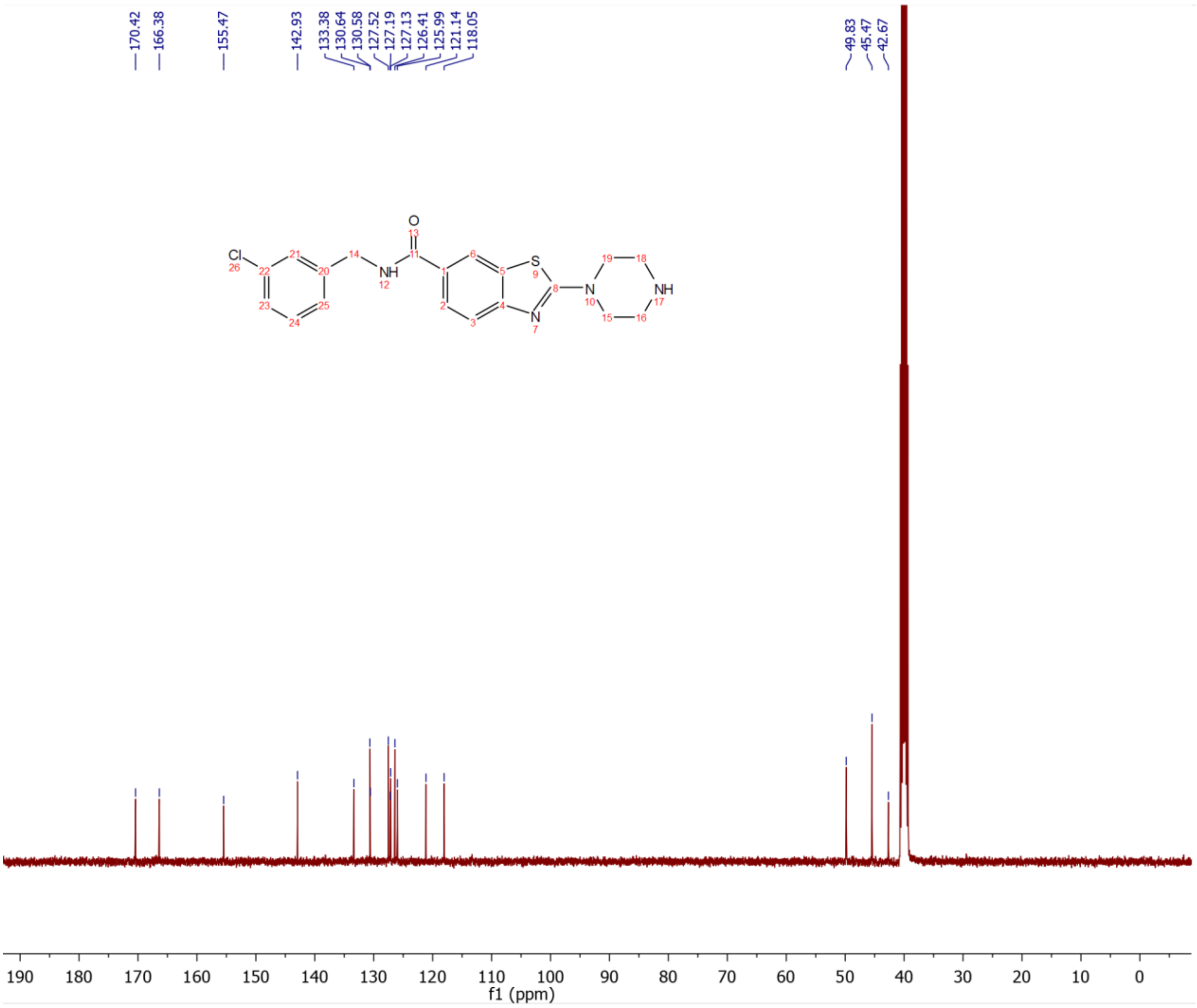

Figure S40. ^{13}C NMR spectrum of compound **5k**.

HPLC: t_r = 4.26 min (98.0 % at 254 nm).

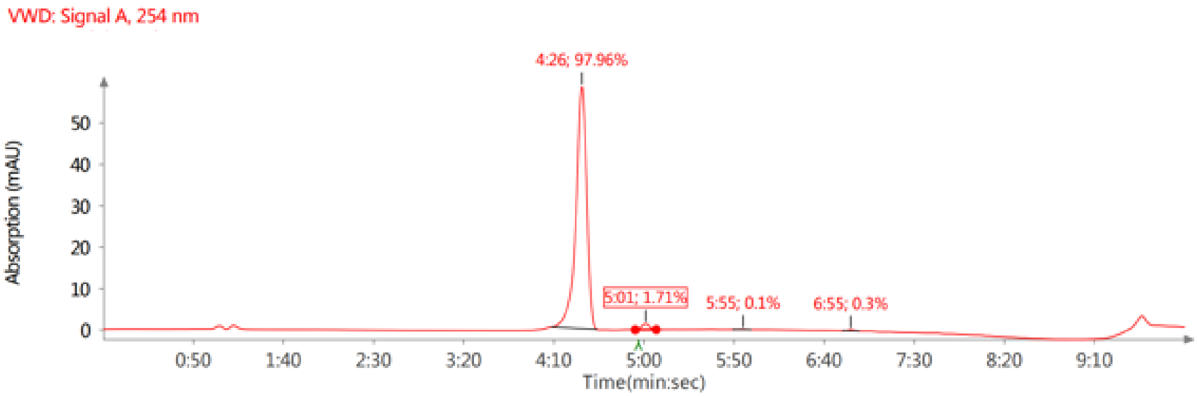

Figure S41. HPLC chromatogram of compound **5k**.

^{1}H NMR (400 MHz, [D$_6$]DMSO, 25°C, TMS): δ = 8.98 (t, *J* = 6.0 Hz, 1H, t, *J* = 5.9 Hz, 1H, CH_2-N*H*-COR), 8.29 (d, *J* = 1.8 Hz, 1H, Ar-*H*$_7$), 7.82 (dd, J_1 = 8.5 Hz, J_2 = 1.8 Hz, 1H, Ar-*H*$_5$), 7.46 (d, *J* = 8.5 Hz, 1H, Ar-*H*$_4$), 7.41 – 7.30 (m, 4H, 4 × Ar-*H*), 4.46 (d, *J* = 6.0 Hz, 2H, RCO-NH-C*H*$_2$-Ph), 3.57 – 3.46 (m, 4H, 2 × piperazine-C*H*$_2$), 2.86 – 2.77 (m, 4H, 2 × piperazine-C*H*$_2$), 2.77 – 2.69 ppm (m, 1H, N*H*)

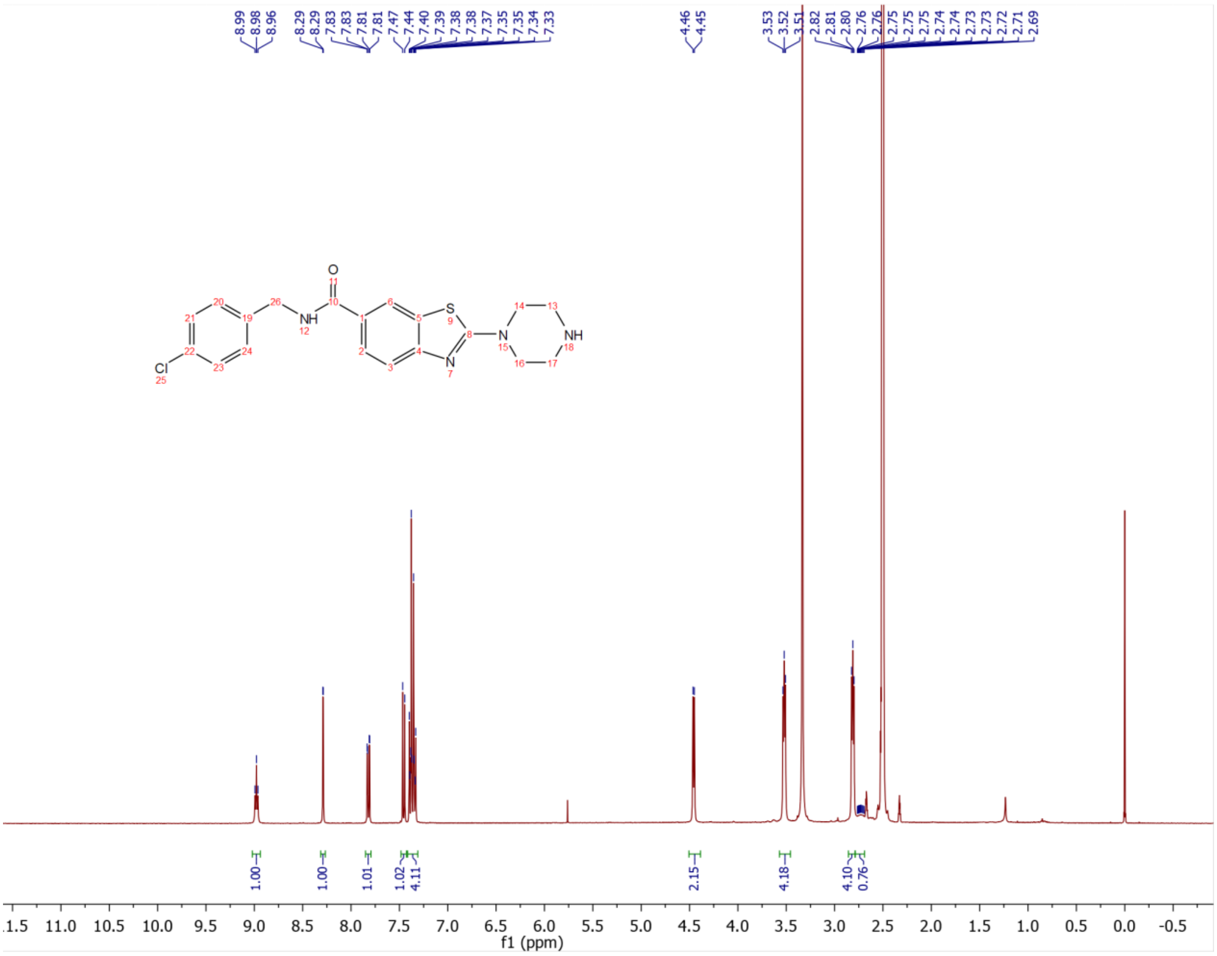

Figure S42. ^{1}H NMR spectrum of compound **5l**.

^{13}C (101 MHz, [D$_6$]DMSO, 25°C, TMS): δ = 170.4, 166.3, 155.4, 139.4, 131.7, 130.6, 129.6 (2C), 128.7 (2C), 127.3, 126.0, 121.1, 118.0, 49.7 (2C), 45.4 (2C), 42.5 ppm

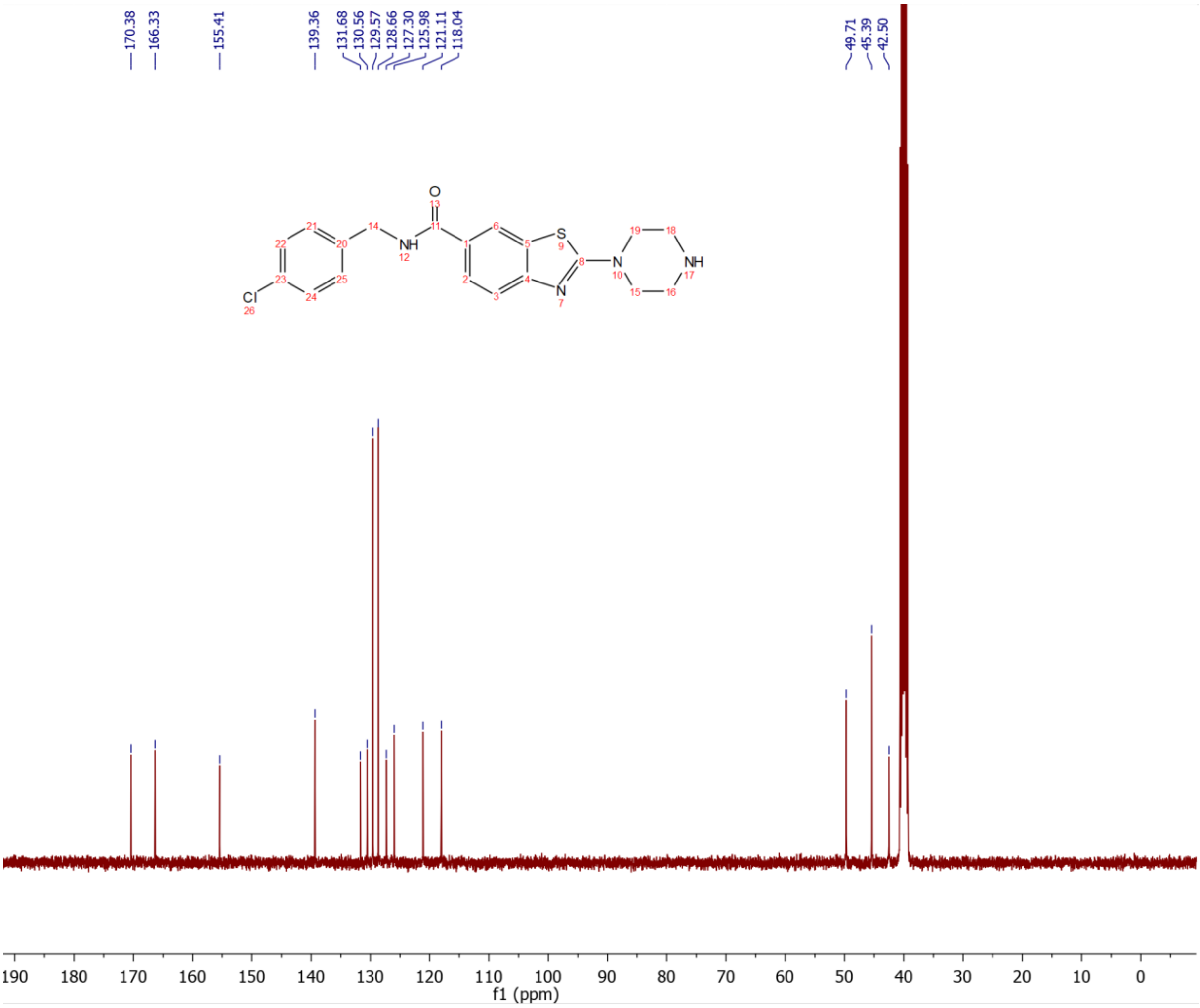

Figure S43. ^{13}C NMR spectrum of compound **5l**.

HPLC: t_r = 4.15 min (97.7 % at 254 nm)

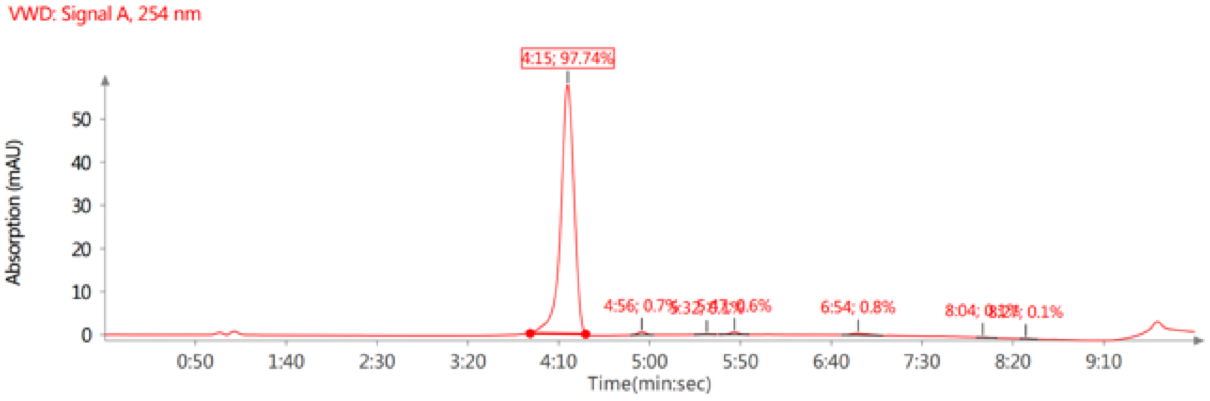

Figure S44. HPLC chromatogram of compound **5l**.

^{1}H NMR (400 MHz, [D$_6$]DMSO, 25°C, TMS): δ = 8.66 (d, *J* = 8.3 Hz, 1H, CH-N*H*-CO), 8.32 (d, *J* = 1.7 Hz, 1H, Ar-*H*$_7$), 7.86 (dd, *J*$_1$ = 8.5 Hz, *J*$_2$ = 1.7 Hz, 1H, Ar-*H*$_5$), 7.44 (d, *J* = 8.5 Hz, 1H, Ar-*H*$_4$), 7.29 – 7.14 (m, 4H, 4 × Ar-*H*), 5.56 (q, *J* = 8.3 Hz, 1H, indane-*H*), 3.55 – 3.48 (m, 4H, 2 × piperazine-C*H*$_2$), 3.00 (ddd, *J*$_1$ = 15.6 Hz, *J*$_2$ = 8.8 Hz, *J*$_3$ = 2.9 Hz, 1H, indane-*H*), 2.90 – 2.77 (m, 5H, 2 × piperazine-C*H*$_2$, and indane-*H*), 2.47 – 2.40 (m, 1H), 2.05 – 1.92 ppm (m, 1H, indane-*H*), not visible (N*H*)

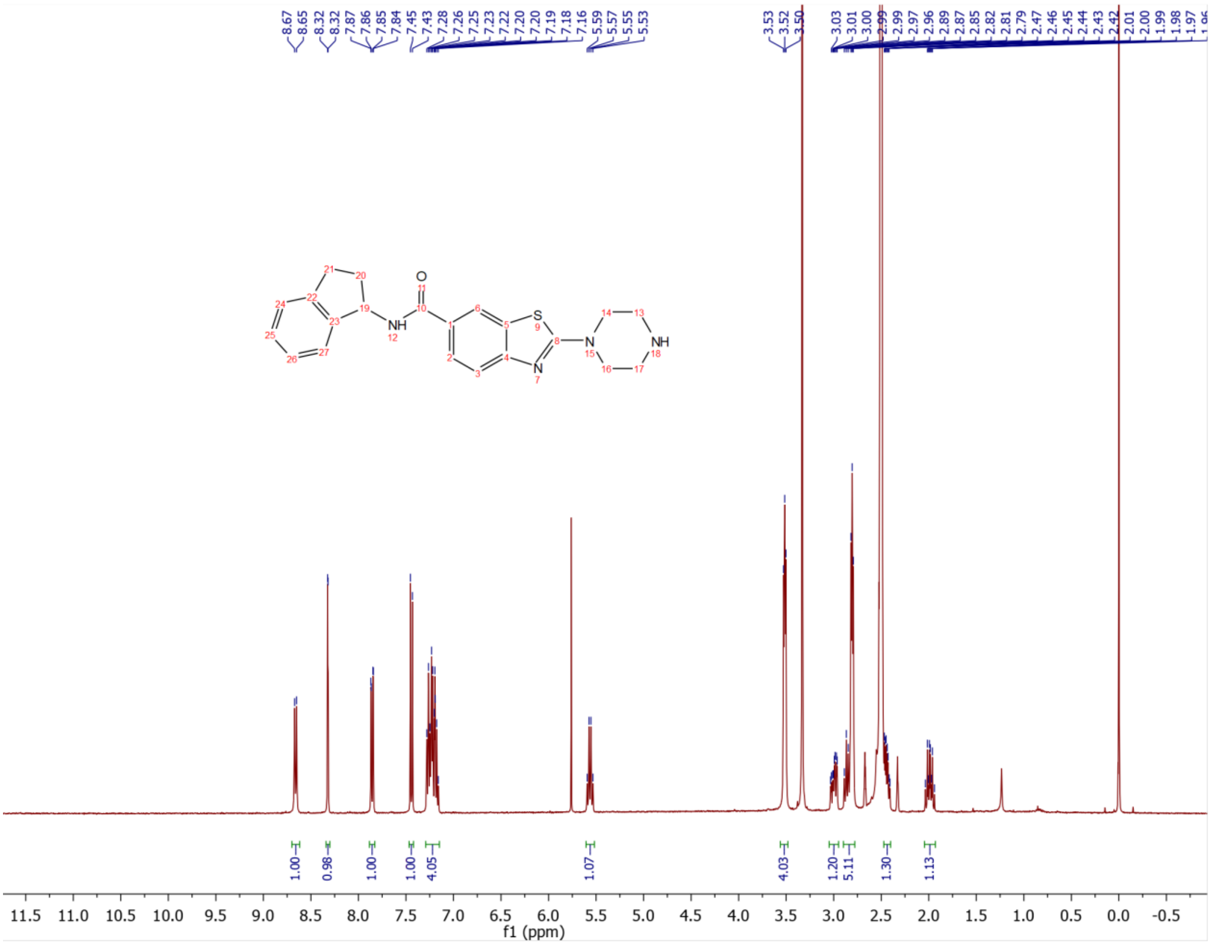

Figure S45. ^{1}H NMR spectrum of compound **5m**.

^{13}C (101 MHz, [D$_6$]DMSO, 25°C, TMS): δ = 170.3, 166.2, 155.3, 144.8, 143.4, 130.4, 127.8, 127.5, 126.8, 126.2, 124.9, 124.5, 121.2, 118.0, 54.7, 49.8 (2C), 45.5 (2C), 33.2, 30.3 ppm

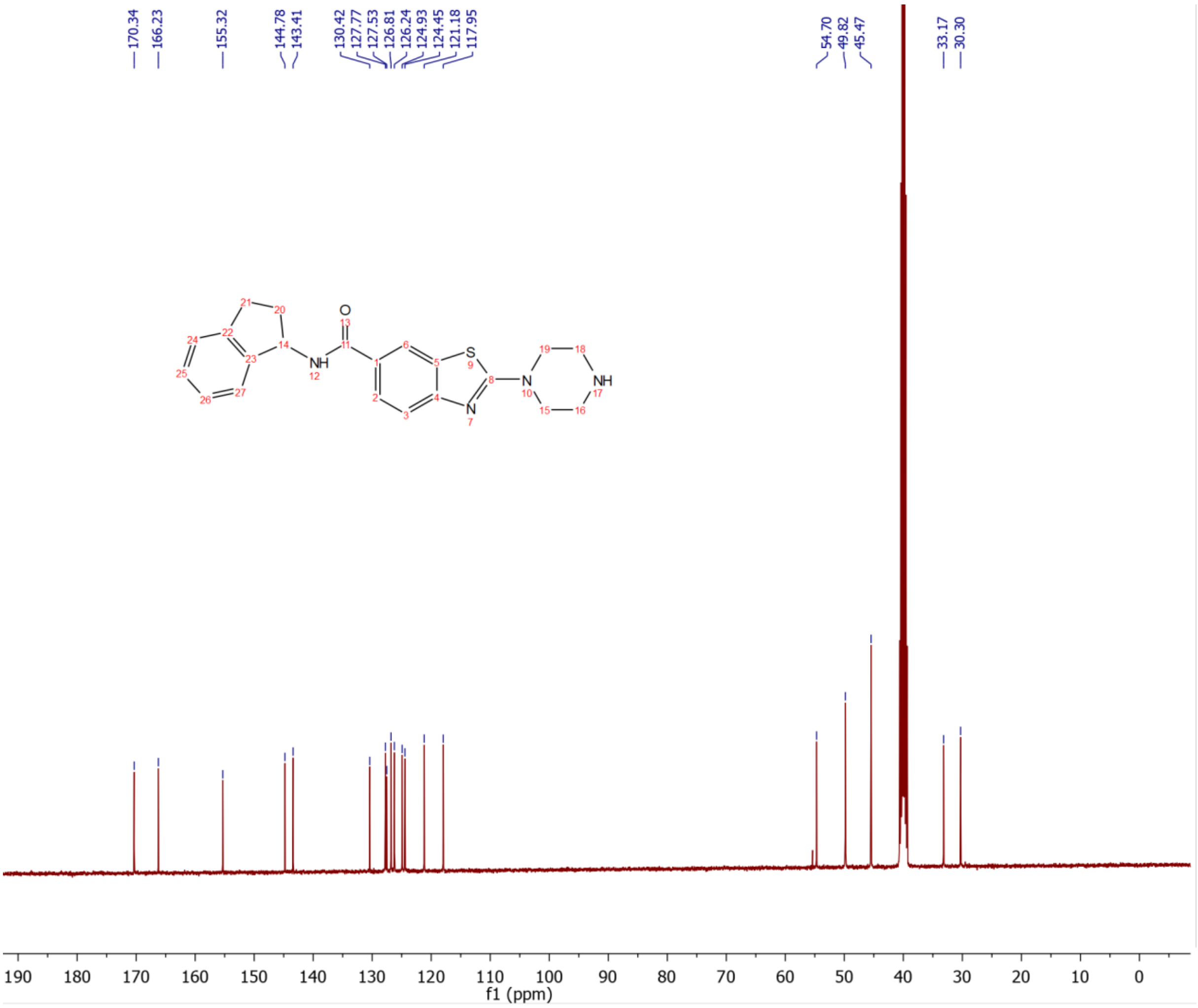

Figure S46. ^{13}C NMR spectrum of compound **5m**.

HPLC: t_r = 4.22 min (96.6 % at 254 nm)

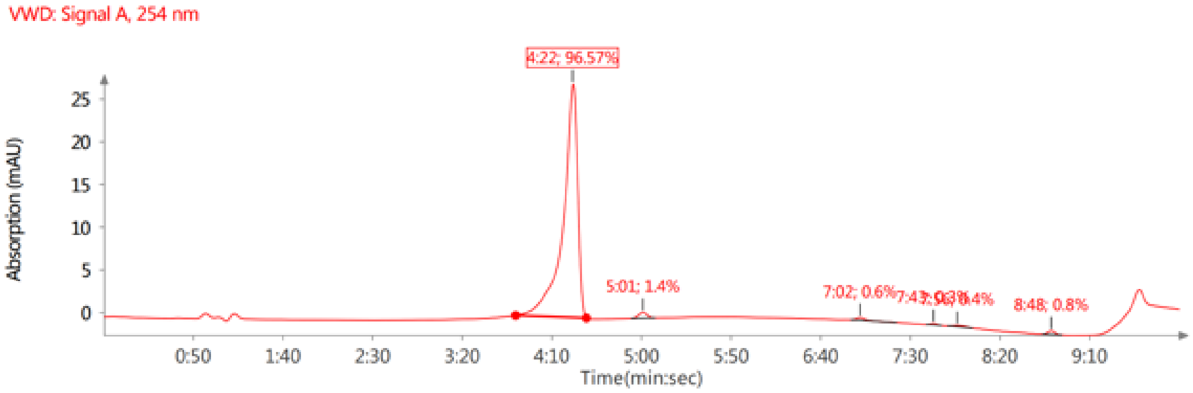

Figure S47. HPLC chromatogram of compound **5m**.

^{1}H NMR (400 MHz, [D$_6$]DMSO, 25°C, TMS): δ = 10.25 (s, 1H, Ar-N*H*-COR) 8.29 (d, *J* = 1.7 Hz, 1H, Ar-N*H*-COR), 7.87 – 7.80 (m, 5H, 5 × Ar-*H*), 7.45 – 7.37 ppm (m, 3H, Ar-*H* and N*H*$_2$);

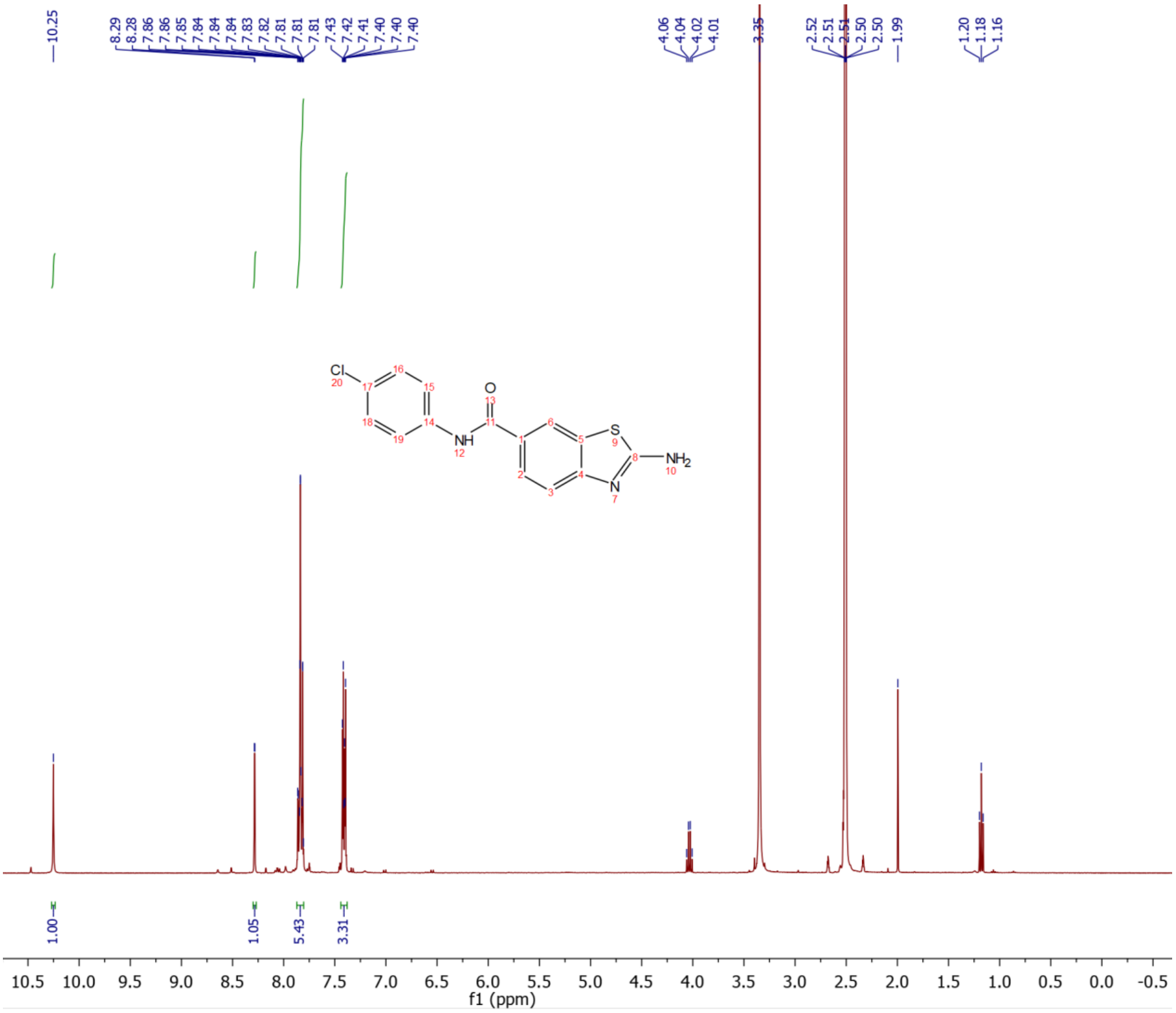

Figure S48. ^{1}H NMR spectrum of compound **6**.

^{13}C (101 MHz, [D_6]DMSO, 25°C, TMS): δ = 169.3, 165.7, 156.2, 138.9, 131.4, 129.0 (2C), 127.4; 127.4, 126.2, 122.1 (2C), 121.3, 117.4 ppm

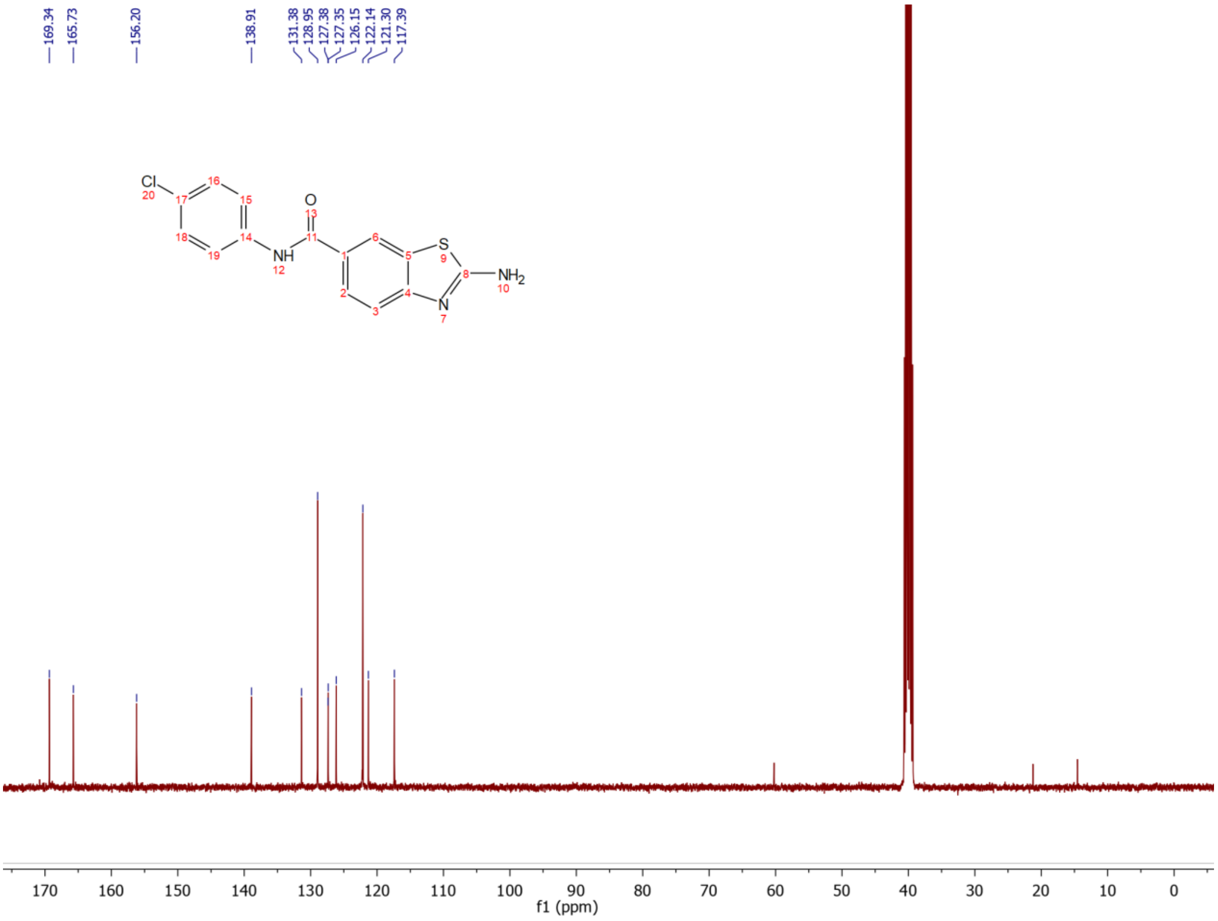

Figure S49. ^{13}C NMR spectrum of compound **6**.

^{1}H NMR (400 MHz, [D$_6$]DMSO, 25°C, TMS): δ = 10.58 (s, 1H, Ar-N*H*-COR), 8.73 – 8.70 (m, 1H, Ar-*H*), 8.16 – 8.12 (m, 1H, Ar-*H*), 8.09 (m, 1H, Ar-*H*), 7.87 – 7.80 (m, 2H, 2 × Ar-*H*), 7.48 – 7.40 ppm (m, 2H, 2 × Ar-*H*);

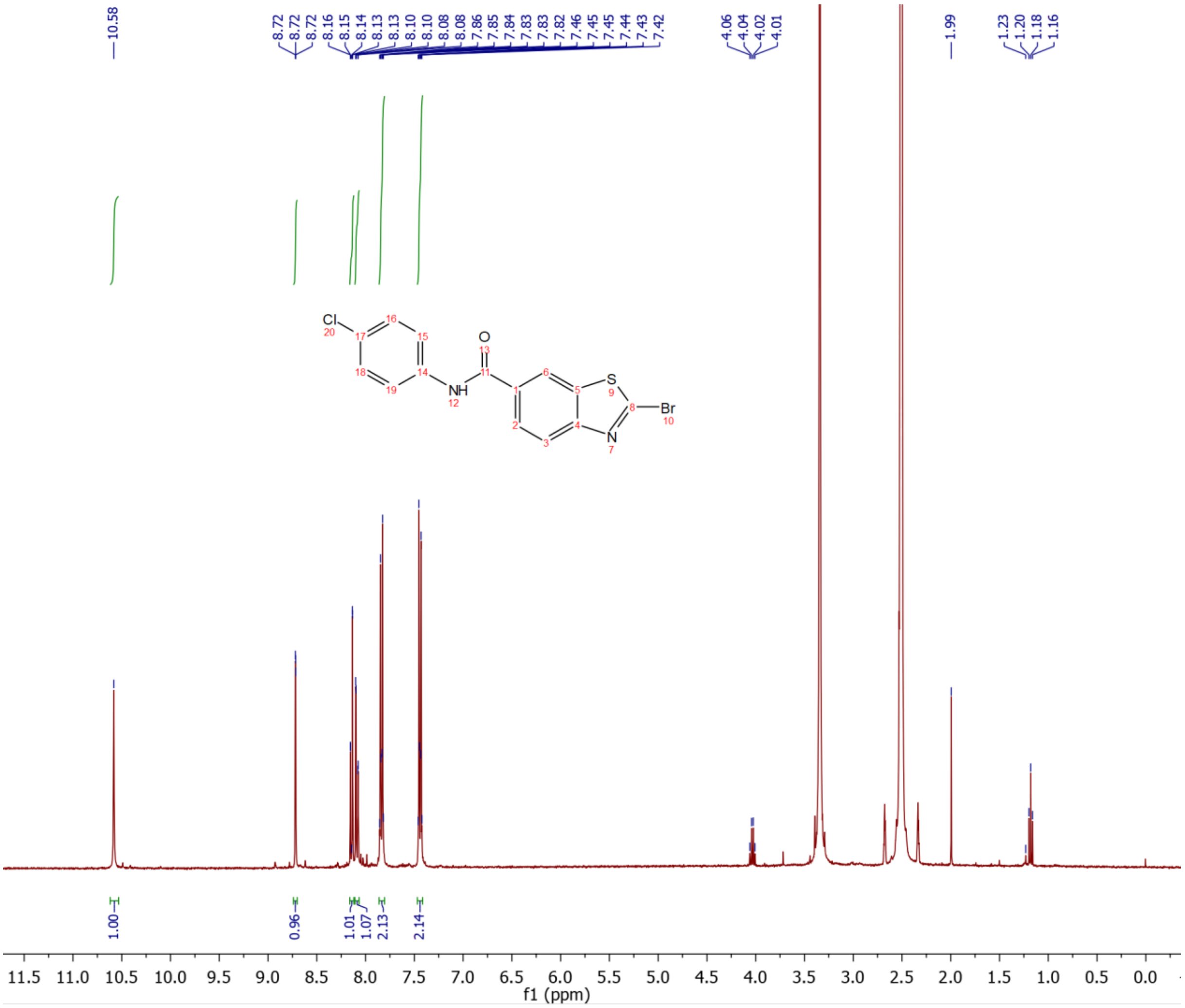

Figure S50. ^{1}H NMR spectrum of compound **7**.

^{13}C (101 MHz, [D6]DMSO, 25°C, TMS): δ = 165.4, 154.2, 143.2, 138.5, 137.4, 132.6, 129.1 (2C), 127.9, 126.8, 122.7, 122.5, 122.3 ppm (2C)

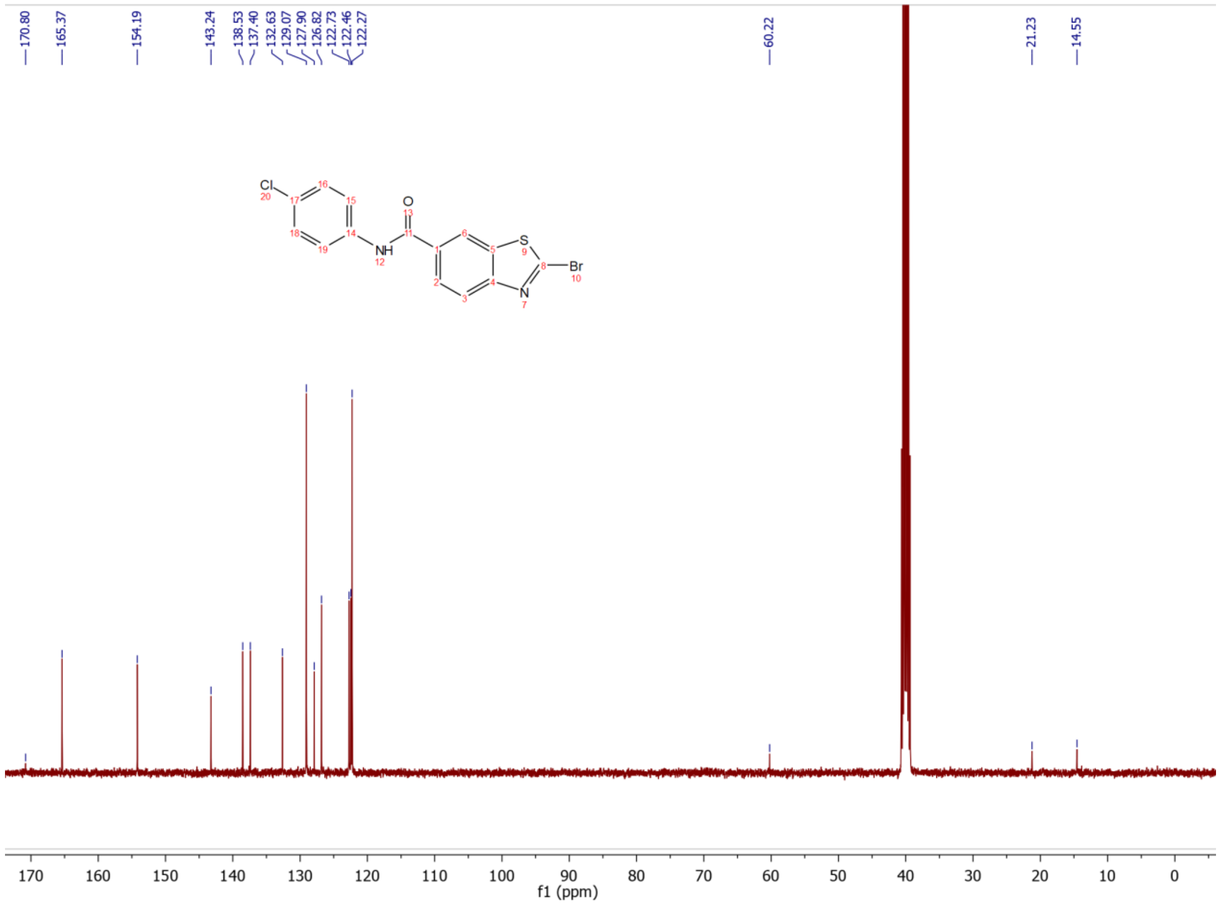

Figure S51. ^{13}C NMR spectrum of compound **7**.

^{1}H NMR (400 MHz, [D$_6$]DMSO, 25°C, TMS): δ = 10.28 (s, 1H, Ar-N*H*-COR), 8.37 (d, *J* = 1.9 Hz, 1H, Ar-*H$_7$*), 7.89 (dd, *J$_1$* = 8.5 Hz, *J$_2$* = 1.9 Hz, 1H, Ar-*H$_5$*), 7.86 – 7.78 (m, 2H, 2 × Ar-*H*), 7.51 (d, *J* = 8.5 Hz, 1H, Ar-*H$_4$*), 7.43 – 7.37 (m, 2H, 2 × Ar-*H*), 6.95 (d, *J* = 7.9 Hz, 1H, CH-N*H*-Boc), 4.02 (d, *J* = 13.5 Hz, 2H, 2 × piperidine-*H*), 3.58 (s, 1H, piperidine-*H*), 3.27 (m, 2H, 2 × piperidine-*H*), 1.87 (m, 2H, 2 × piperidine-*H*), 1.50 – 1.41 (m, 2H, 2 × piperidine-*H*), 1.39 ppm (s, 9H, COC(C*H$_3$*)$_3$)

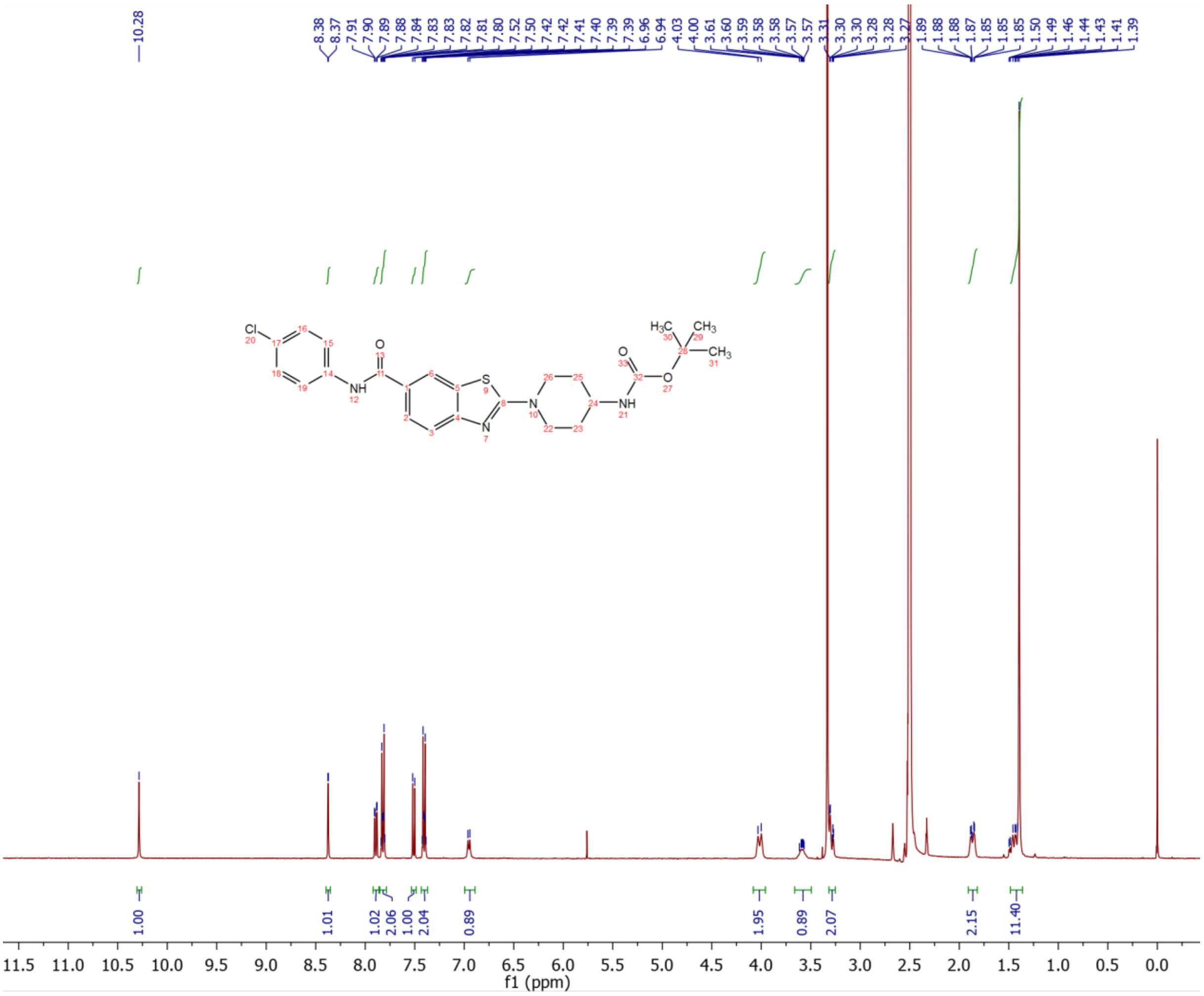

Figure S52. ^{1}H NMR spectrum of compound **8i**.

^{13}C (101 MHz, [D_6]DMSO, 25°C, TMS): δ = 168.9, 164.5, 154.9, 154.2, 137.8, 129.9, 127.9 (2C), 126.4, 126.4, 125.5, 121.1 (2C), 120.5, 116.9, 77.1, 46.6 (2C), 46.2, 30.5 (2C), 27.6 ppm (3C)

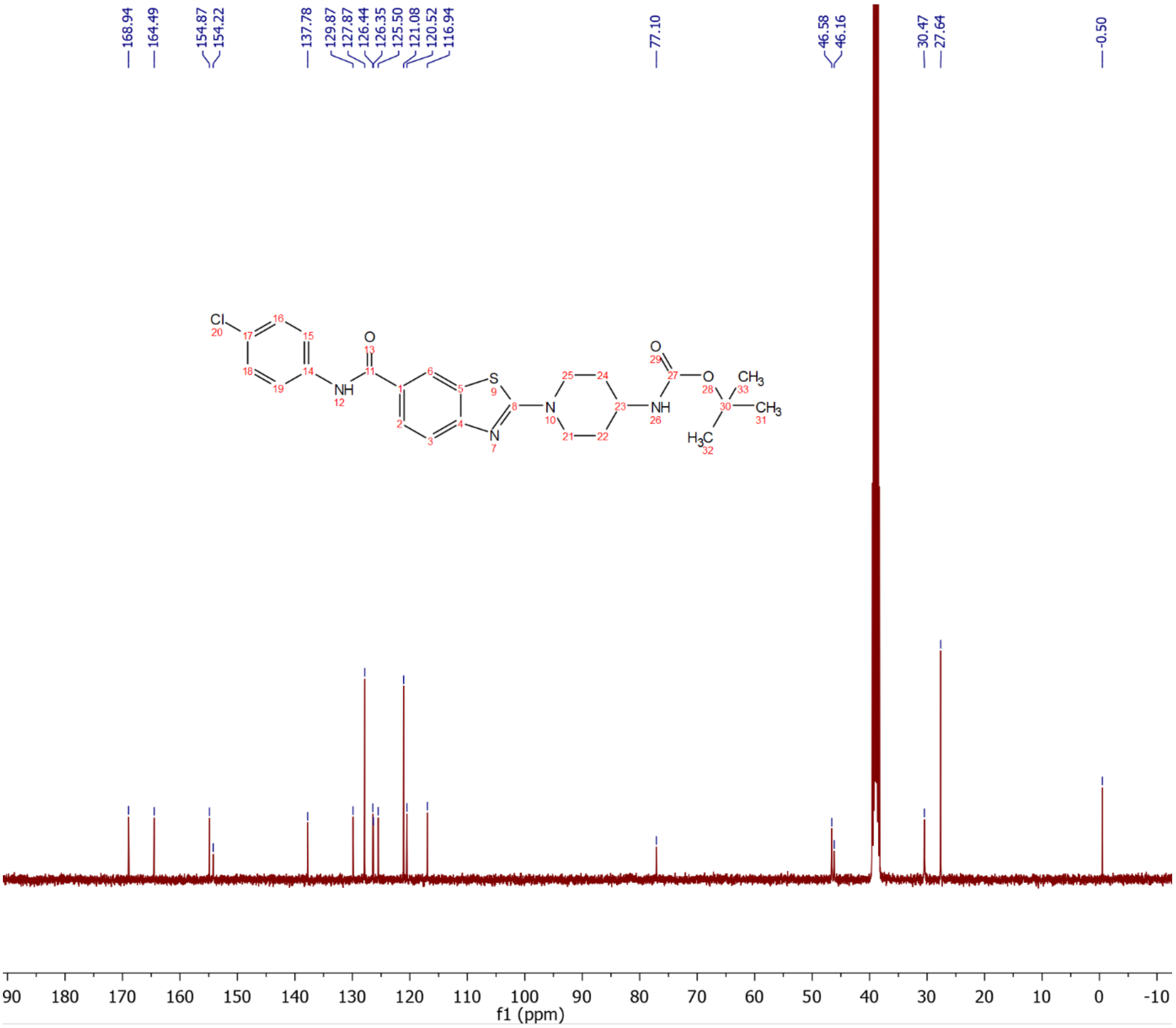

Figure S53. ^{13}C NMR spectrum of compound **8i**.

^{1}H NMR (400 MHz, [D$_6$]DMSO, 25°C, TMS): δ = 10.29 (s, 1H, Ar-N*H*-COR), 8.38 (d, *J* = 1.9 Hz, 1H, Ar-*H$_7$*), 7.90 (dd, *J$_1$* = 8.5 Hz, *J$_2$* = 1.9 Hz, 1H, Ar-*H$_5$*), 7.85 – 7.79 (m, 2H, 2 × Ar-*H*), 7.52 (d, *J* = 8.5 Hz, 1H, Ar-*H$_4$*), 7.43 – 7.38 (m, 2H, 2 × Ar-*H*), 7.36 (s, 1H, CH-CON*H$_a$*), 6.87 (s, 1H, CH-CON*H$_b$*), 4.08 (d, *J* = 13.0 Hz, 2H, 2 × piperidine-*H*), 3.29 – 3.21 (m, 2H, 2 × piperidine-*H*), 2.45 – 2.39 (m, 1H, piperidine-*H*), 1.90 – 1.79 (m, 2H, 2 × piperidine-*H*), 1.68 – 1.56 ppm (m, 2H, 2 × piperidine-*H*)

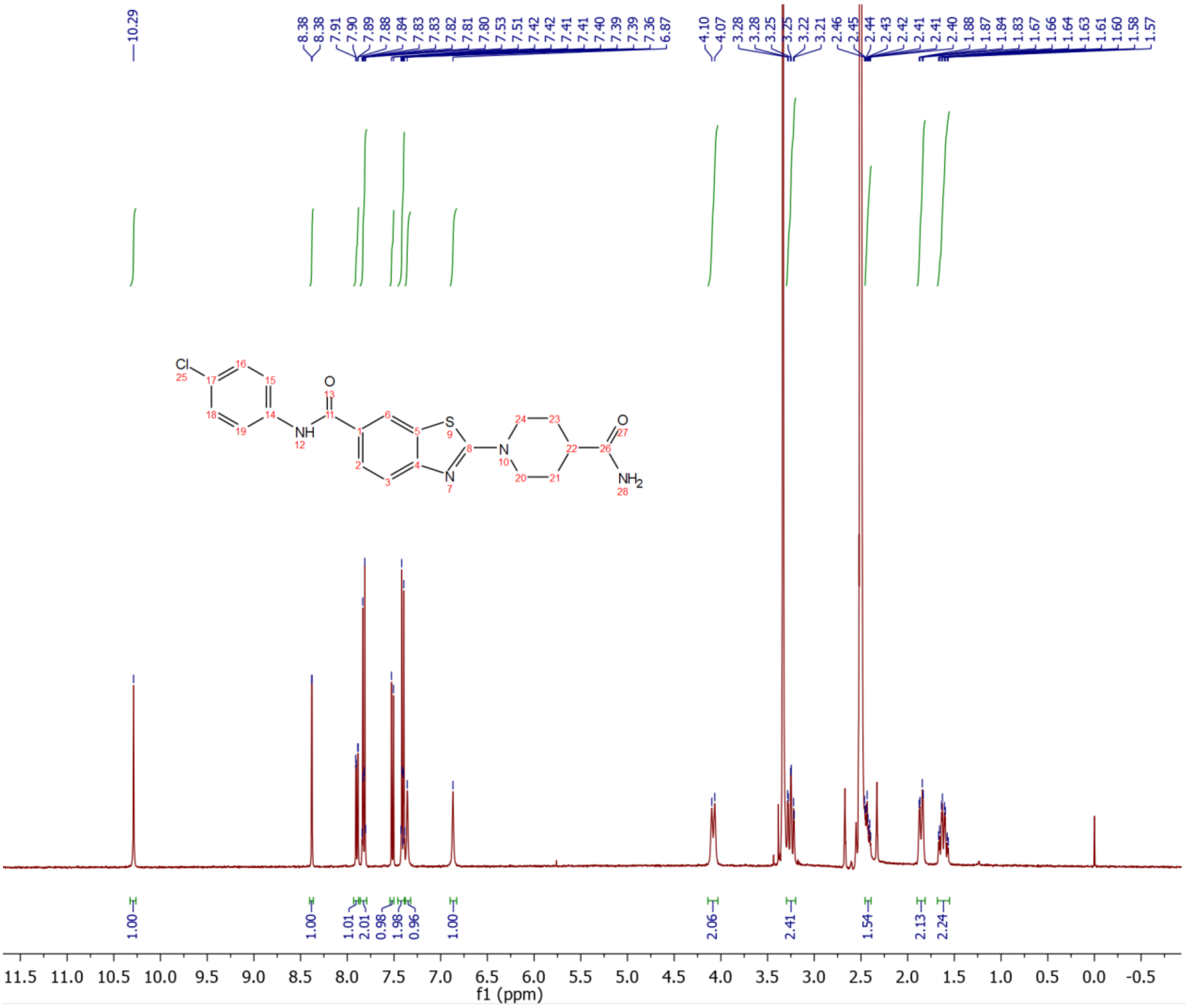

Figure S54. ^{1}H NMR spectrum of compound **8k**.

^{13}C (101 MHz, [D$_6$]DMSO, 25°C, TMS): δ = 176.1, 170.2, 165.6, 155.9, 138.9, 130.8, 129.0 (2C), 127.5, 127.4, 126.6, 122.2 (2C), 121.6, 118.0, 48.4, 41.5 (2C), 28.2 ppm (2C)

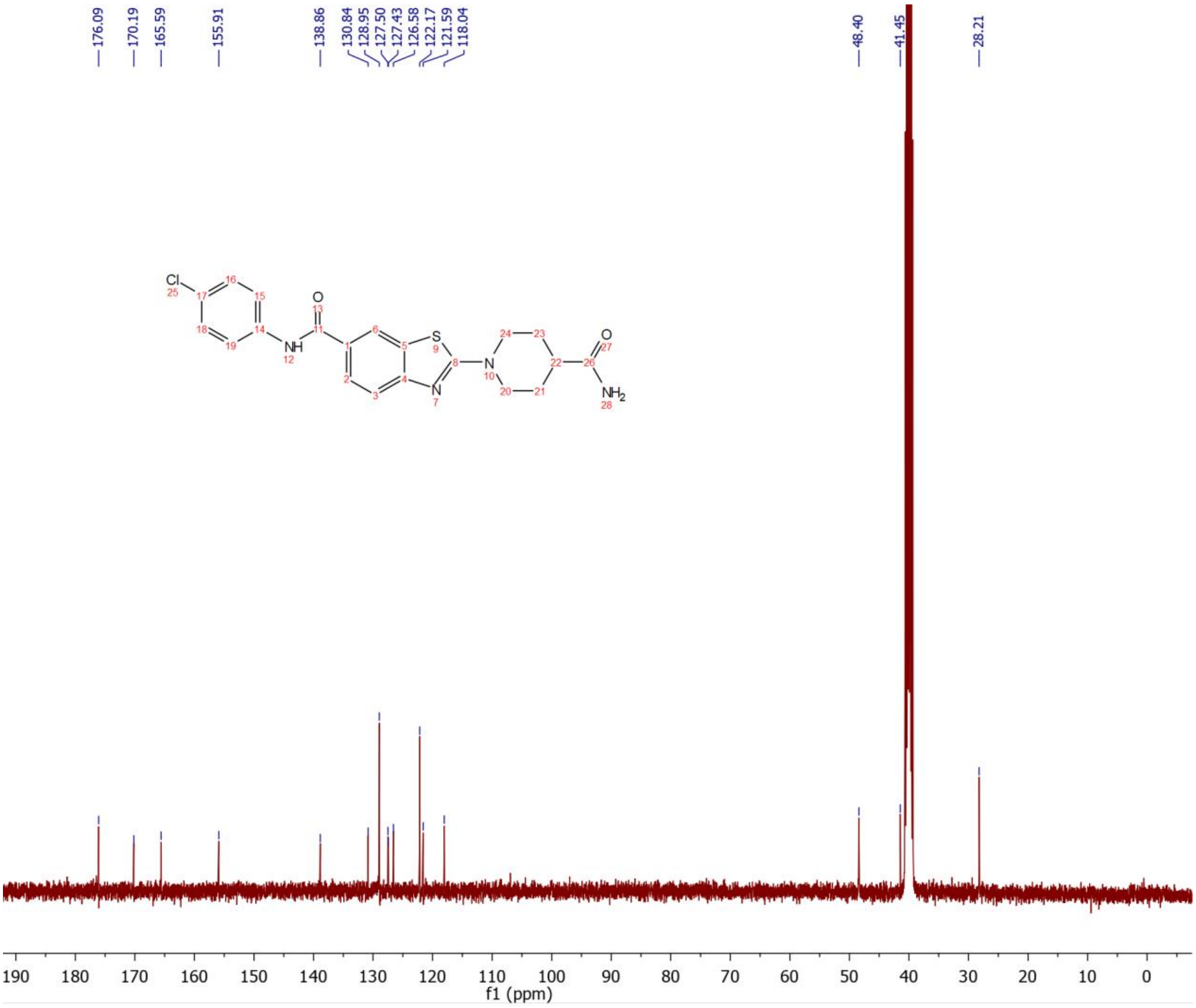

Figure S55. ^{13}C NMR spectrum of compound **8k**.

HPLC: t_r = 5.41 min (99.0 % at 254 nm)

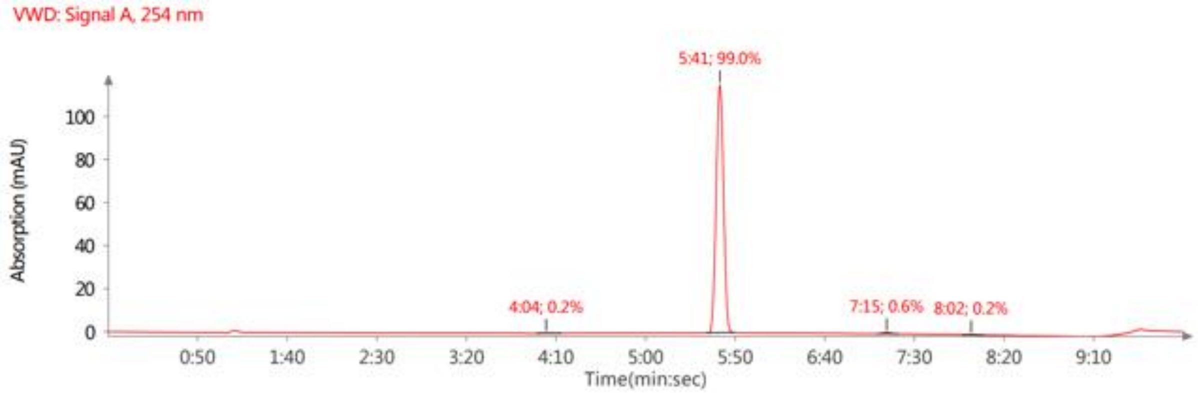

Figure S56. HPLC chromatogram of compound **8k**.

^{1}H NMR (400 MHz, [D$_6$]DMSO, 25°C, TMS): δ = 10.29 (s, 1H, Ar-N*H*-COR), 8.38 (s, 1H, Ar-*H*), 7.90 (d, *J* = 8.5 Hz, 1H, Ar-*H*), 7.82 (d, *J* = 8.4 Hz, 2H, 2 × Ar-*H*), 7.52 (d, *J* = 8.5 Hz, 1H, Ar-*H*), 7.46 (s, 1H, CO-NH_a), 7.41 (d, *J* = 8.4 Hz, 2H, 2 × Ar-*H*), 6.98 (s, 1H, CO-NH_b), 4.15 – 4.07 (m, 1H, piperidine-*H*), 4.00 – 3.93 (m, 1H, piperidine- *H*), 3.30 – 3.19 (m, 2H, 2 × piperidine-*H*), 2.00 – 1.92 (m, 1H, piperidine- *H*), 1.85 – 1.77 (m, 1H, piperidine- *H*), 1.71 – 1.50 ppm (m, 3H, 3 × piperidine-*H*)

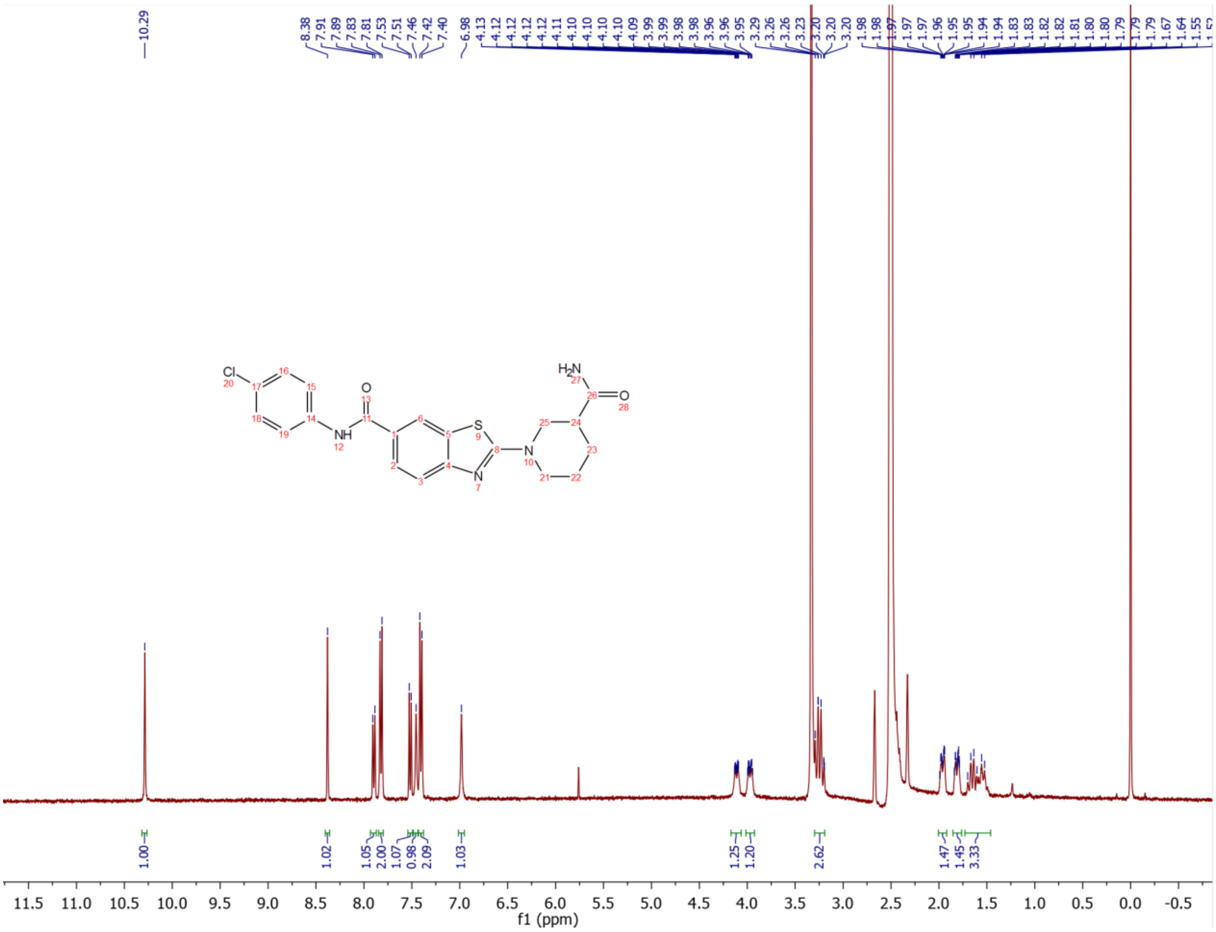

Figure S57. ^{1}H NMR spectrum of compound **8l**.

^{13}C (101 MHz, [D$_6$]DMSO, 25°C, TMS): δ = 174.8, 170.2, 165.6, 155.9, 138.9, 130.8, 129.0 (2C), 127.5, 127.4, 126.6, 122.2 (2C), 121.6, 118.0, 51.0, 49.2, 41.8, 27.8, 24.3 ppm

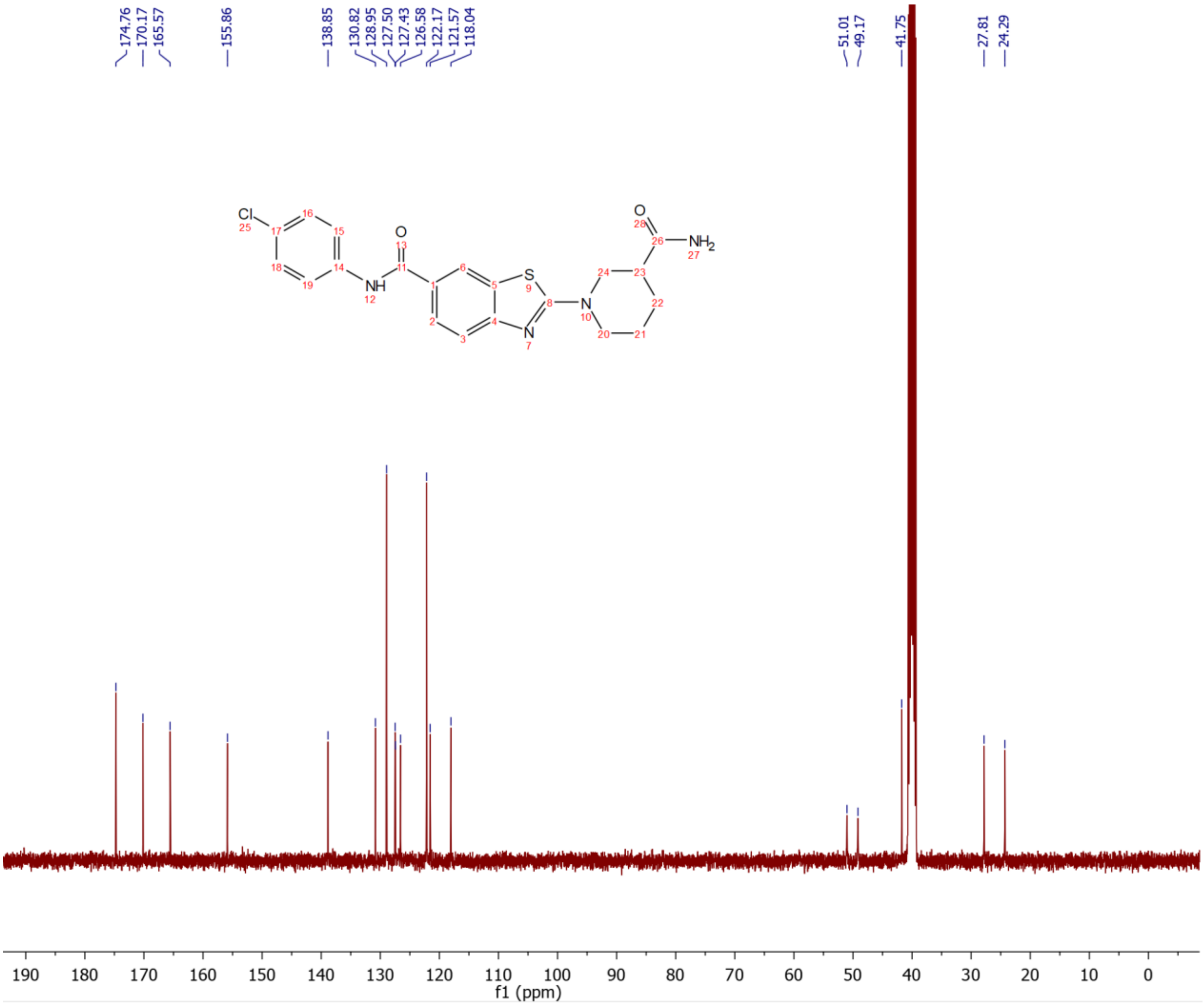

Figure S58. ^{13}C NMR spectrum of compound **8l**.

HPLC: t_r = 5.54 min (99.9 % at 254 nm)

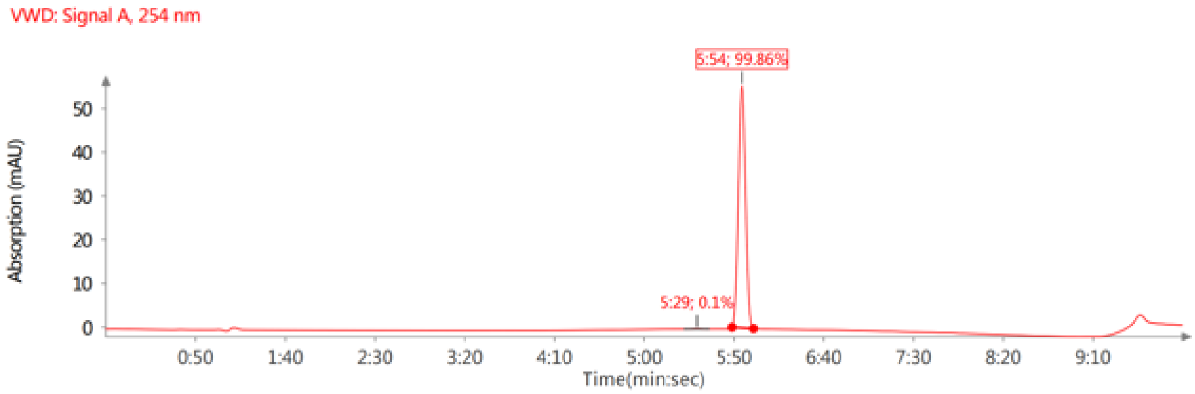

Figure S59. HPLC chromatogram of compound **8l**.

^{1}H NMR (400 MHz, [D$_6$]DMSO, 25°C, TMS): δ = 10.28 (s, 1H, Ar-N*H*-COR), 8.37 (d, *J* = 1.8 Hz, 1H, Ar-*H*$_7$), 7.89 (dd, J_1 = 8.5 Hz, J_2 = 1.8 Hz, 1H, Ar-*H*$_5$), 7.86 – 7.79 (m, 2H, 2 × Ar-*H*), 7.50 (d, *J* = 8.5 Hz, 1H, Ar-*H*$_4$), 7.44 – 7.37 (m, 2H, 2 × Ar-*H*), 4.88 (d, *J* = 4.1 Hz, 1H, CH-O*H*), 3.94 – 3.83 (m, 2H, 2 × piperidine-*H*), 3.84 – 3.76 (m, 1H, piperidine-*H*), 3.46 – 3.37 (m, 2H, 2 × piperidine-*H*), 1.93 – 1.80 (m, 2H, 2 × piperidine-*H*), 1.56 – 1.41 ppm (m, 2H, 2 × piperidine-*H*)

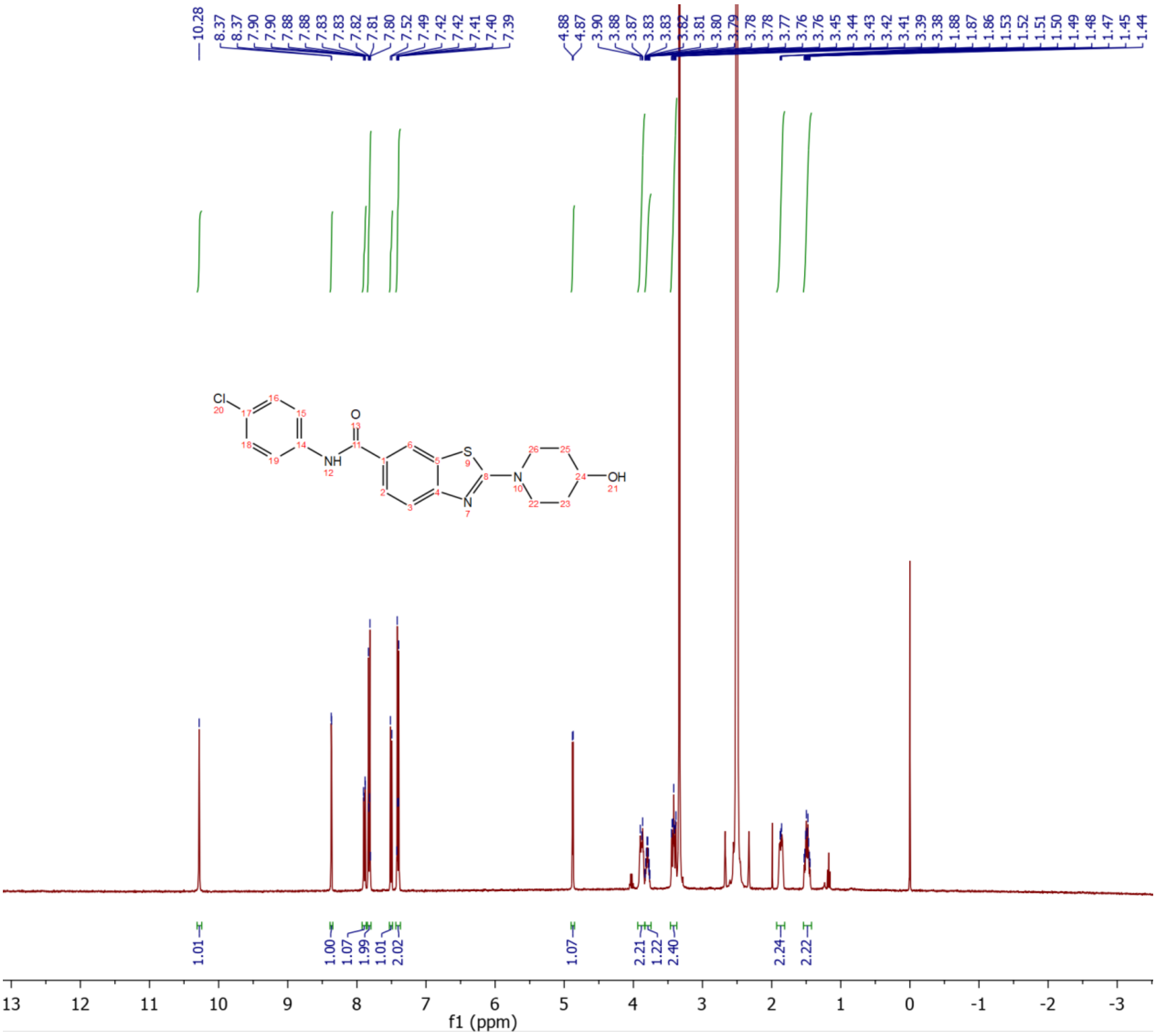

Figure S60. ^{1}H NMR spectrum of compound **8m**.

^{13}C (101 MHz, [D6]DMSO, 25°C, TMS): δ = 170.1, 165.6, 156.0, 138.9, 130.9, 129.0 (2C), 127.4, 126.6, 122.2 (2C), 121.6, 118.0, 65.5, 46.41 (2C), 33.81 (2C) ppm

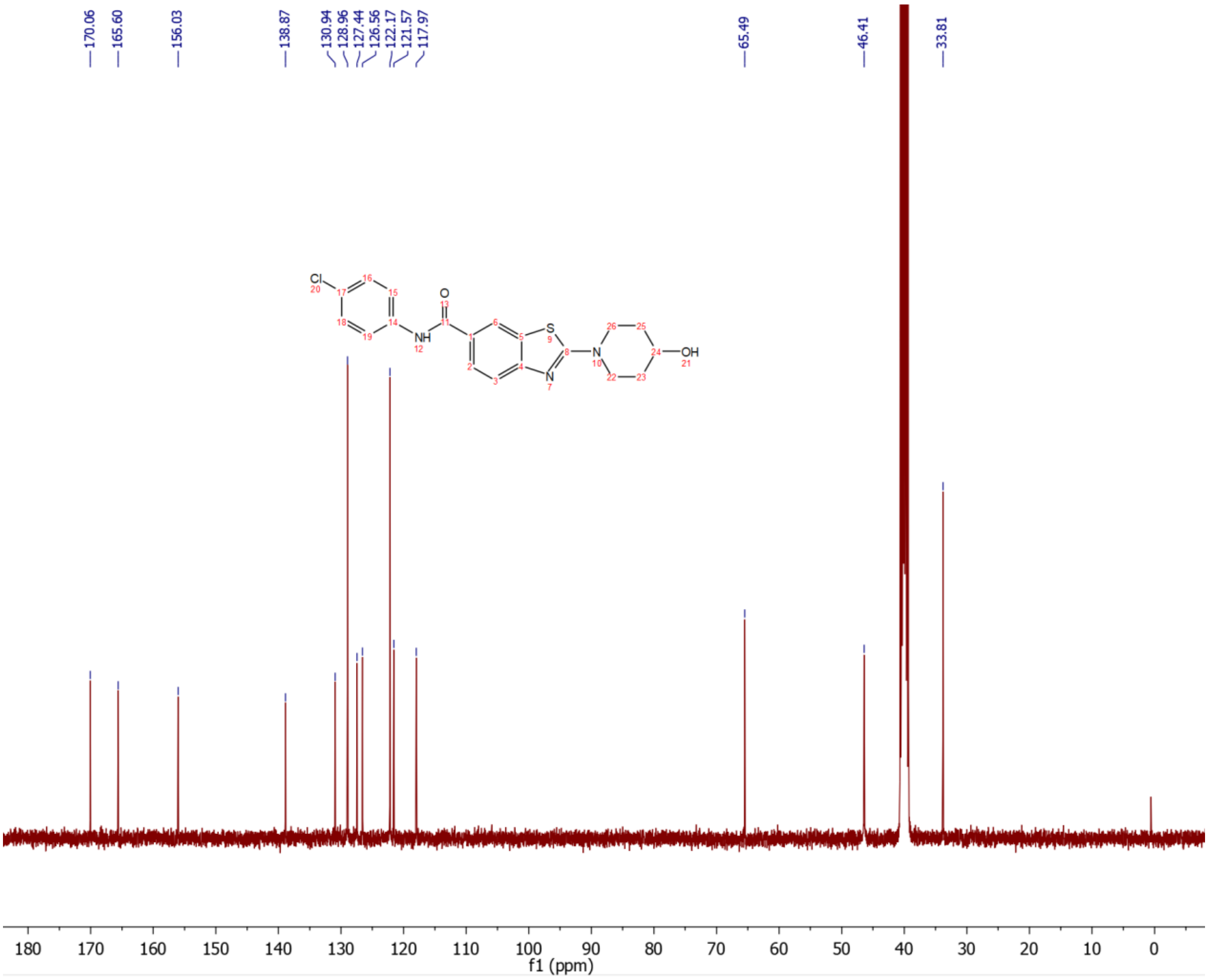

Figure S61. ^{13}C NMR spectrum of compound **8m**.

HPLC: t_r = 6.00 min (96.2 % at 254 nm)

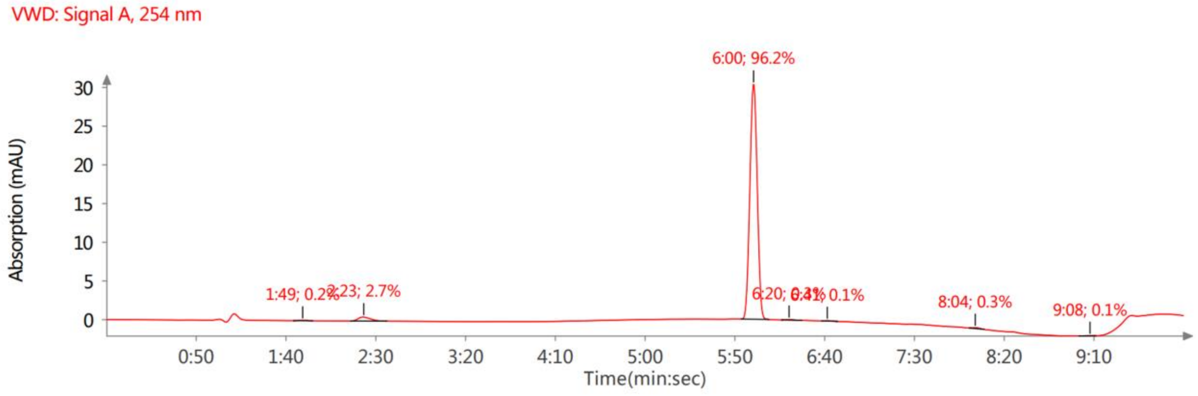

Figure S62. HPLC chromatogram of compound **8m**.

^{1}H NMR (400 MHz, [D$_6$]DMSO, 25°C, TMS): δ = 10.28 (s, 1H, Ar-N*H*-COR), 8.37 (d, *J* = 1.9 Hz, 1H, Ar-*H*$_7$), 7.89 (dd, *J*$_1$ = 8.5 Hz, *J*$_2$ = 1.9 Hz, 1H, Ar-*H*$_5$), 7.85 – 7.78 (m, 2H, 2 × Ar-*H*), 7.50 (d, *J* = 8.5 Hz, 1H, Ar-*H*$_4$), 7.44 – 7.38 (m, 2H, 2 × Ar-*H*), 4.11 – 4.02 (m, 2H, 2 × piperidine-*H*), 3.25 – 3.17 (m, 2H, 2 × piperidine-*H*), 1.80 – 1.62 (m, 3H, 3 × piperidine-*H*), 1.28 – 1.14 (m, 2H, 2 × piperidine-*H*), 0.96 ppm (d, *J* = 6.4 Hz, 3H, C*H*$_3$)

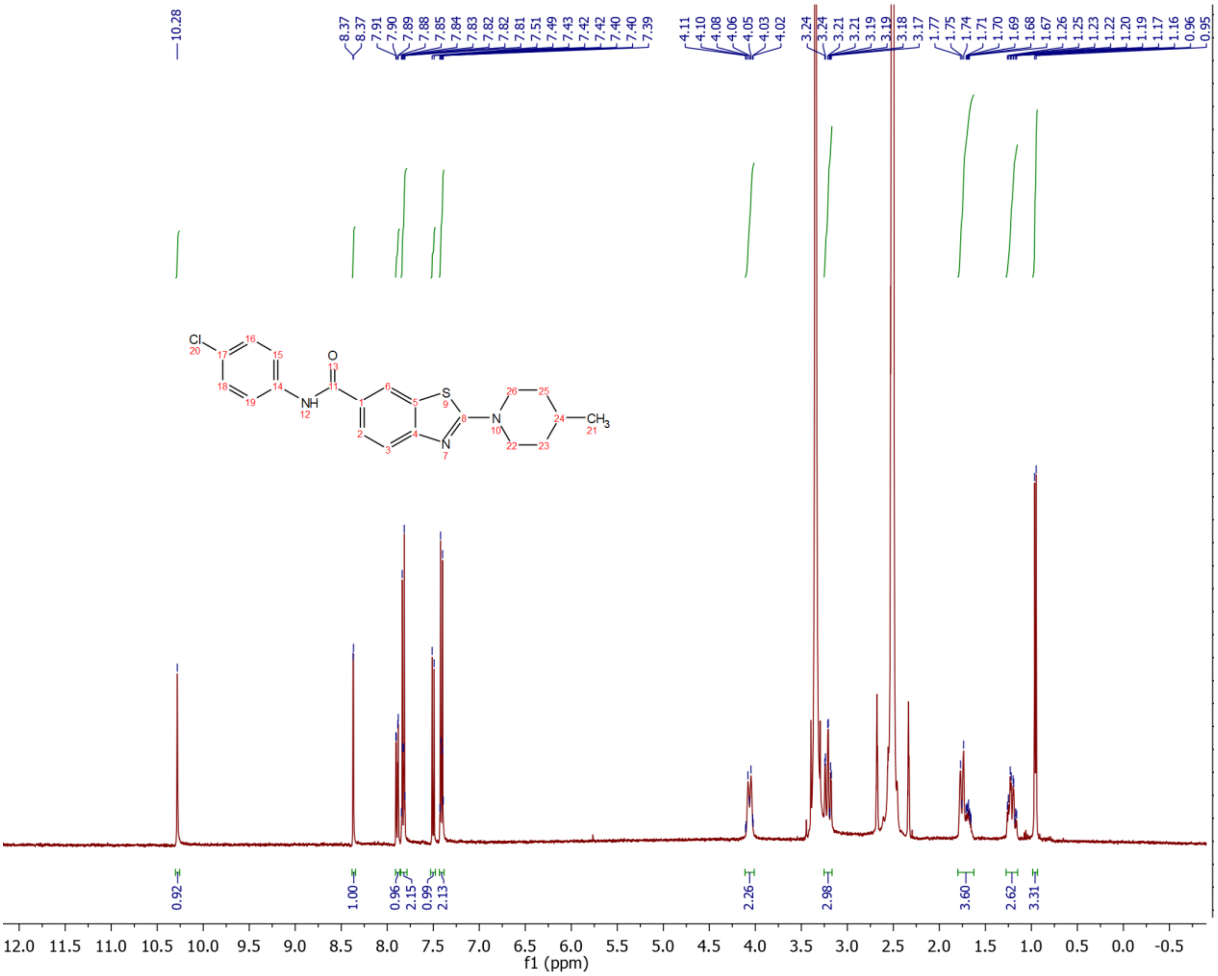

Figure S63. ^{1}H NMR spectrum of compound **8n**.

^{13}C (101 MHz, [D$_6$]DMSO, 25°C, TMS): δ = 170.1, 165.6, 156.0, 138.9, 130.8, 128.9 (2C), 127.4, 127.4, 126.6, 122.2 (2C), 121.5, 117.9, 49.0 (2C), 33.5 (2C), 30.5, 22.0 ppm

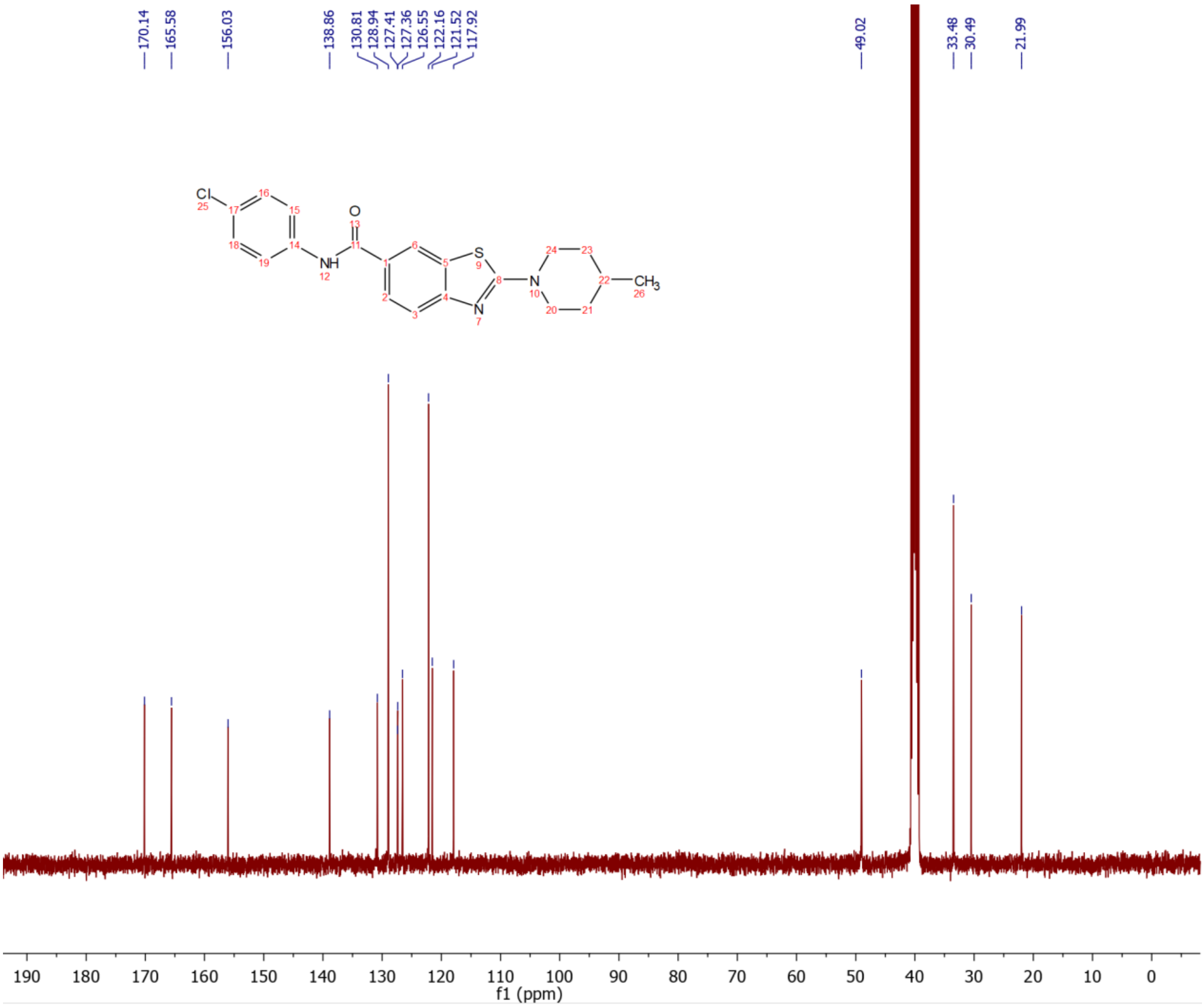

Figure S64. ^{13}C NMR spectrum of compound **8n**.

HPLC: t_r = 7.49 min (99,4 % at 254 nm)

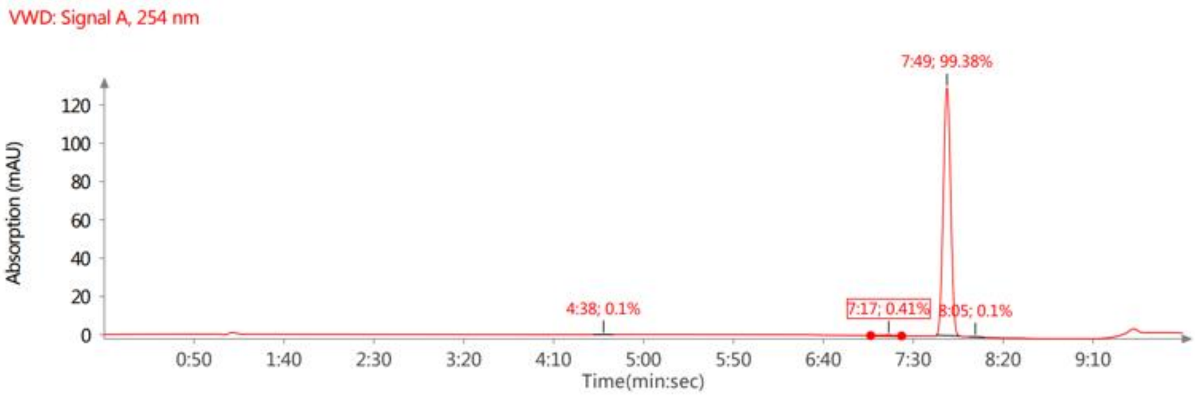

Figure S65. HPLC chromatogram of compound **8n**.

^{1}H NMR (400 MHz, [D$_6$]DMSO, 25°C, TMS): δ = 10.25 (s, 1H, Ar-N*H*-COR), 8.28 (d, *J* = 1.8 Hz, 1H, Ar-*H*), 7.88 – 7.78 (m, 3H, 3 × Ar-*H*), 7.48 – 7.36 (m, 3H, 3 × Ar-*H*), 3.44 (t, *J* = 6.8 Hz, 2H, N-C*H*$_2$-CH$_2$), 2.63 (t, *J* = 6.8 Hz, 2H, N-C*H*$_2$-CH$_2$), 1.66 ppm (p, *J* = 6.8 Hz, 2H), not visible (N*H* and N*H*$_2$);

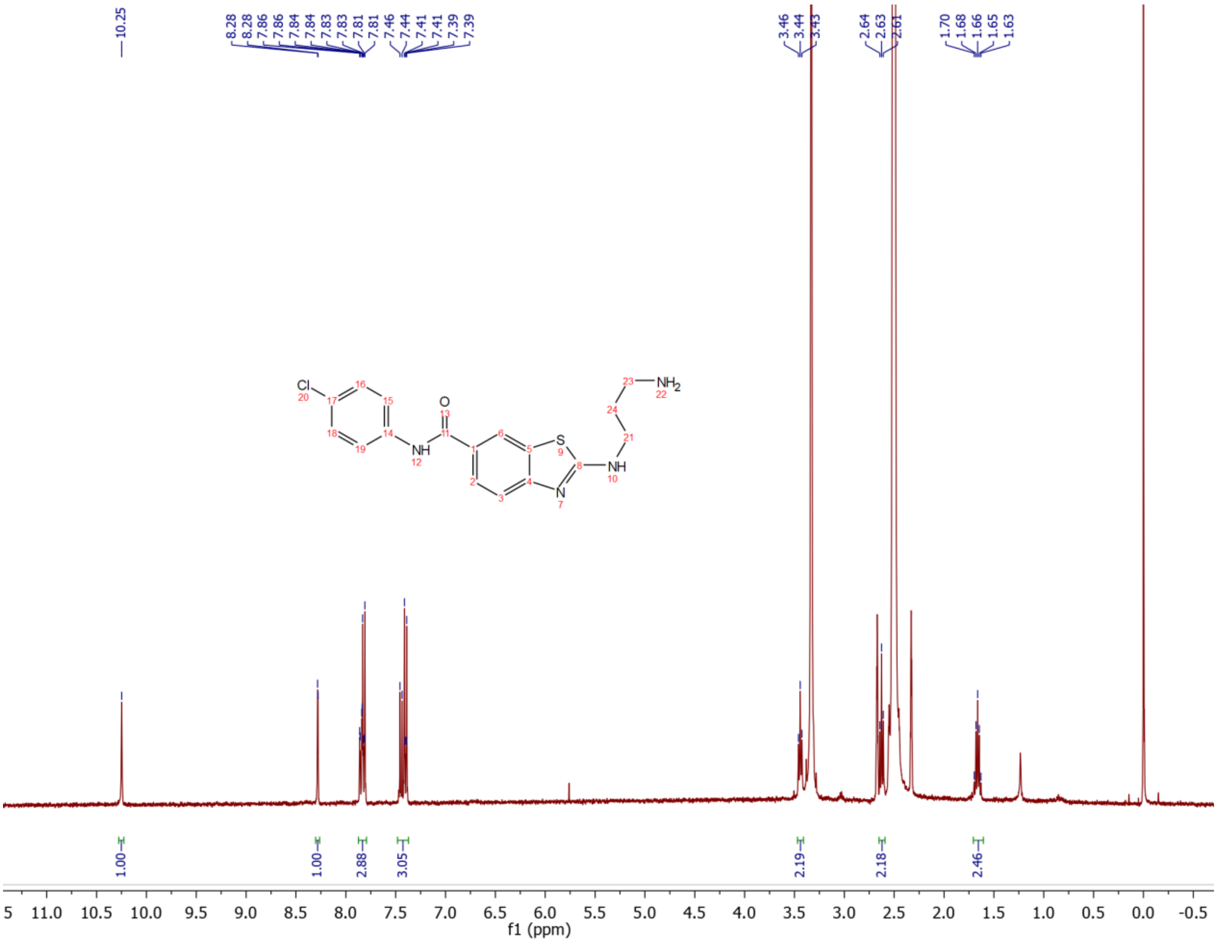

Figure S66. ^{1}H NMR spectrum of compound **9a**.

^{13}C (101 MHz, [D$_6$]DMSO, 25°C, TMS): δ = 168.8, 165.7, 156.0, 138.9, 130.7, 128.9 (2C), 127.4, 127.2, 126.2, 122.1 (2C), 121.3, 117.5, 42.4 (2C), 33.0 ppm

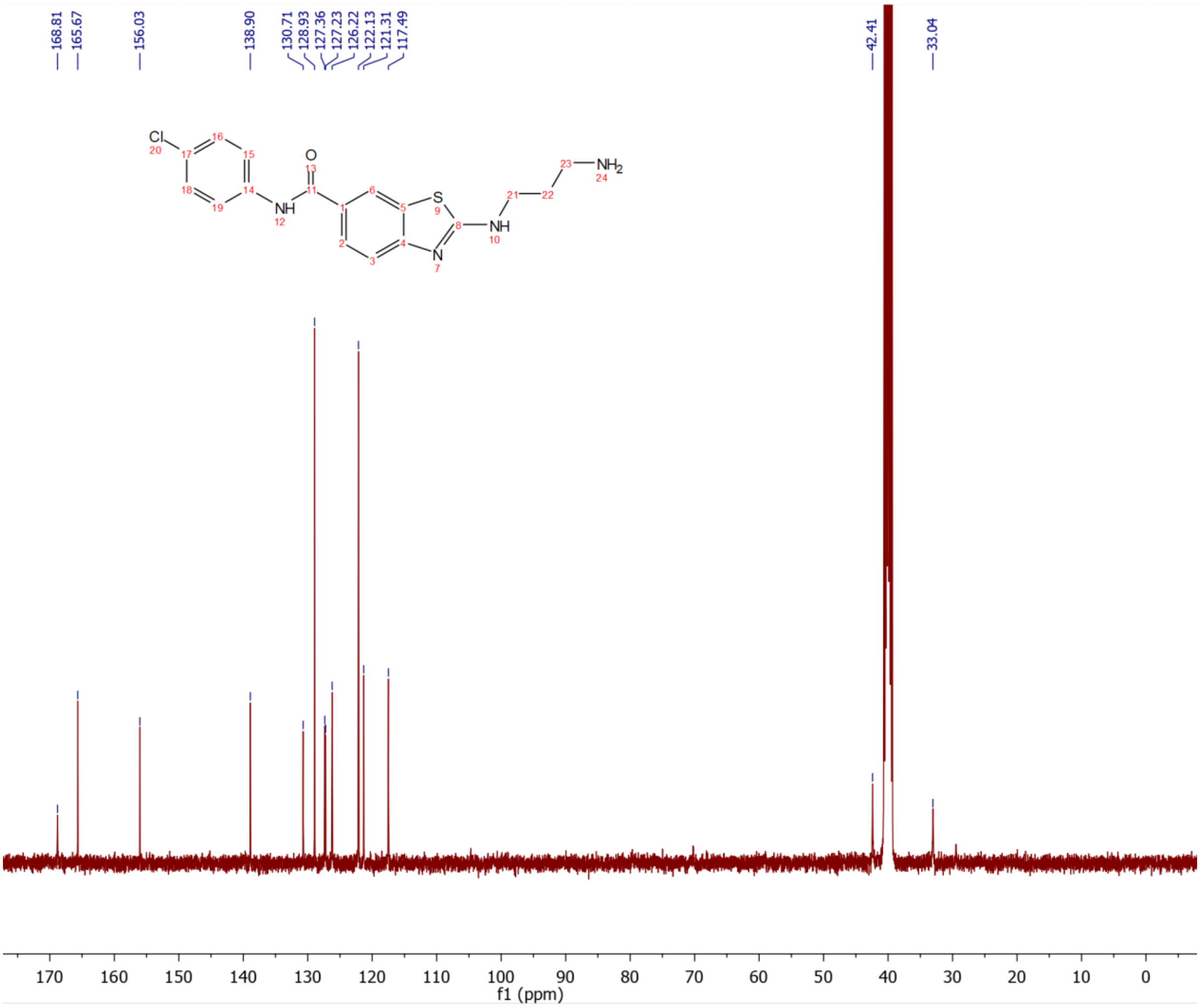

Figure S67. ^{13}C NMR spectrum of compound **9a**.

HPLC: t_r = 4.40 min (99.8 % at 254 nm).

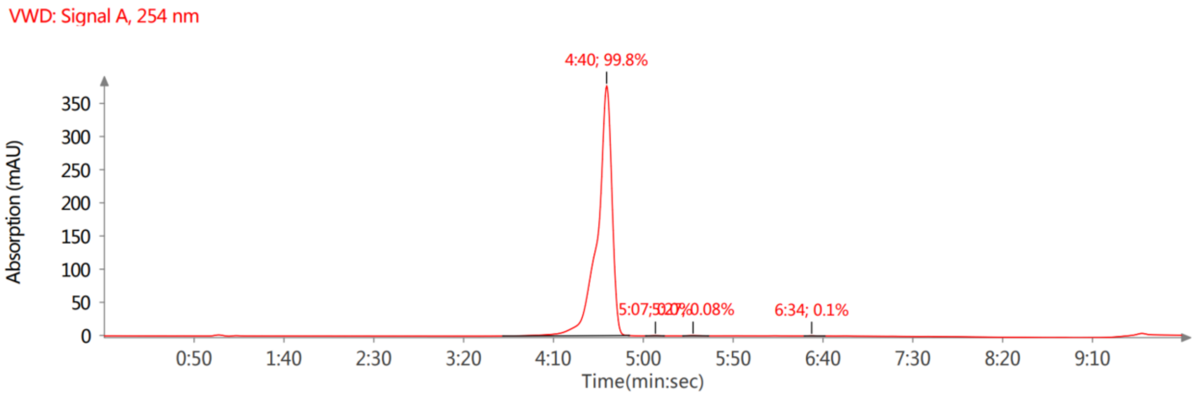

Figure S68. HPLC chromatogram of compound **9a**.

^1H NMR (400 MHz, [D_6]DMSO, 25°C, TMS): δ = 10.27 (s, 1H, Ar-N*H*-COR), 8.36 (d, *J* = 1.8 Hz, 1H, Ar-H_7), 7.89 (dd, J_1 = 8.5 Hz, J_2 = 1.8 Hz, 1H, Ar-H_5), 7.85 – 7.78 (m, 2H, 2 × Ar-*H*), 7.49 (d, *J* = 8.5 Hz, 1H, Ar-H_4), 7.43 – 7.38 (m, 2H, 2 × Ar-*H*), 3.95 (dd, J_1 = 29.1 Hz, J_2 = 12.8 Hz, 2H, CH-NH_2), 3.23 – 3.14 (m, 1H, piperidine-*H*), 2.93 – 2.86 (m, 1H, piperidine-*H*), 2.79 – 2.70 (m, 1H, piperidine-*H*), 1.93 – 1.84 (m, 1H, piperidine-*H*), 1.83 – 1.70 (m, 3H, 3 × piperidine-*H*), 1.60 – 1.46 (m, 1H, piperidine-*H*), 1.34 – 1.21 ppm (m, 1H, piperidine-*H*)

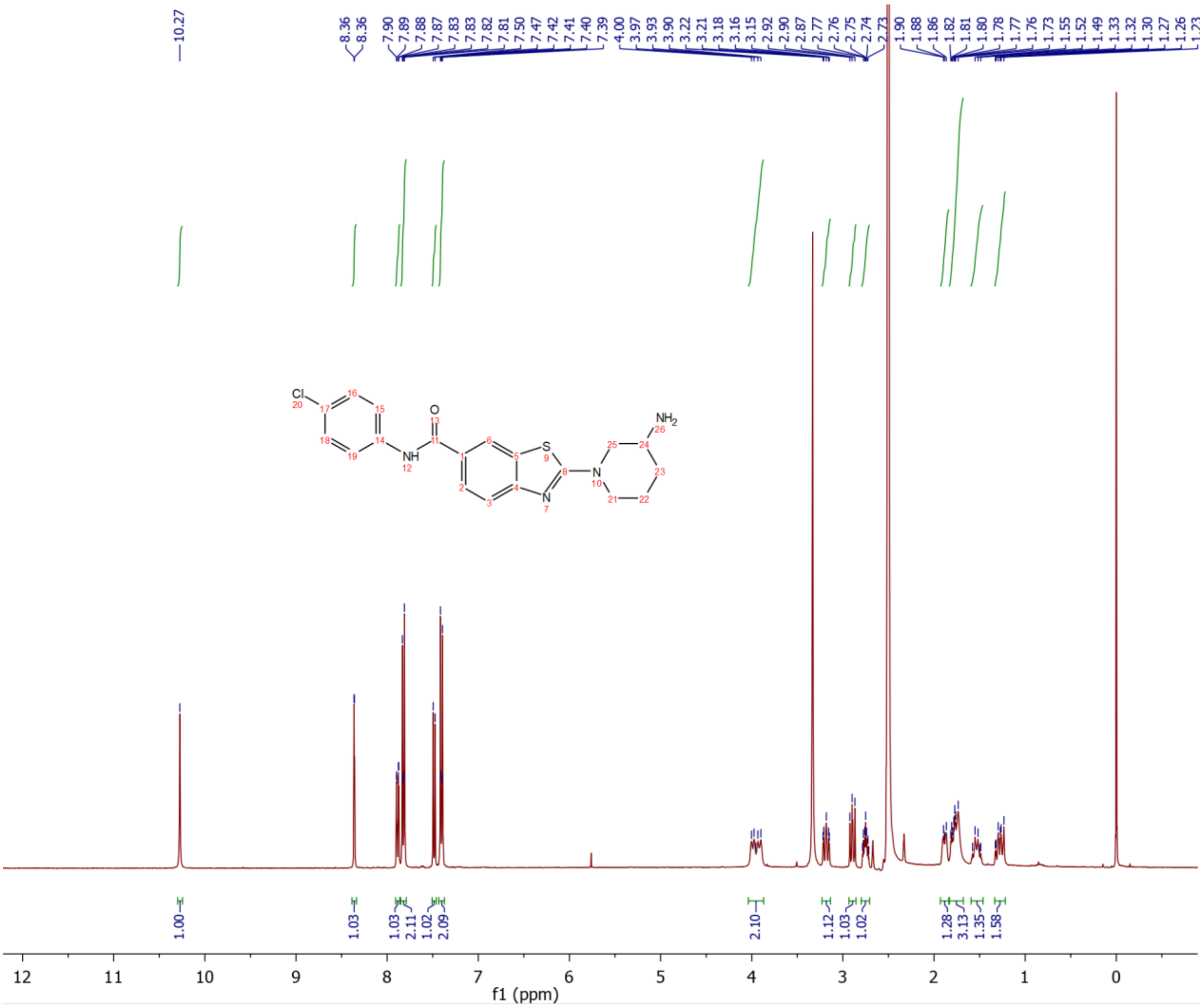

Figure S69. ^1H NMR spectrum of compound **9b**.

^{13}C (101 MHz, [D$_6$]DMSO, 25°C, TMS): δ = 170.2, 165.6, 156.0, 138.9, 130.8, 129.0 (2C), 127.4, 127.3, 126.6, 122.2 (2C), 121.5, 117.9, 57.1, 48.9, 47.9, 33.7, 23.7 ppm

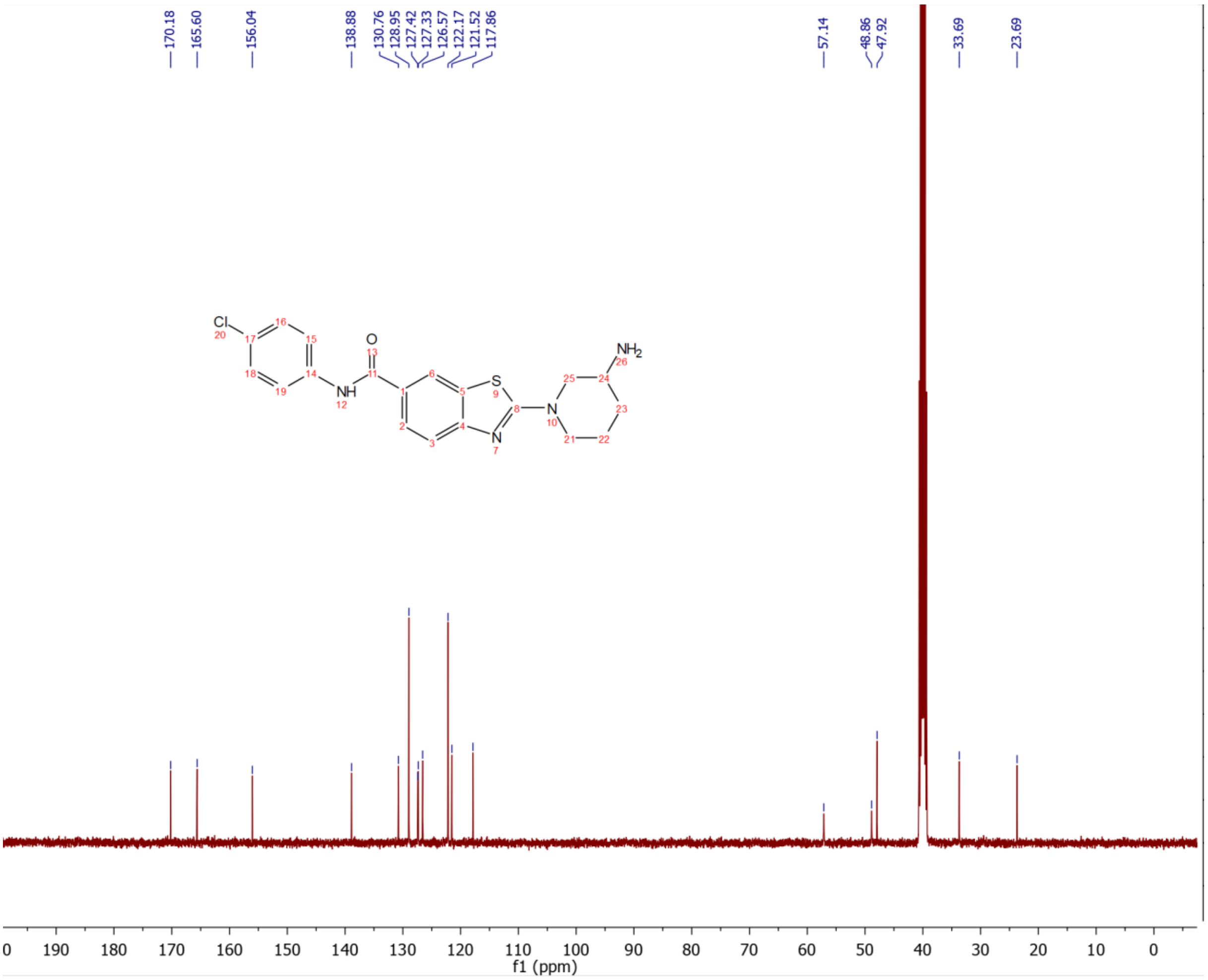

Figure S70. ^{13}C NMR spectrum of compound **9b**.

HPLC: t_r = 5.43 min (97.9 % at 254 nm)

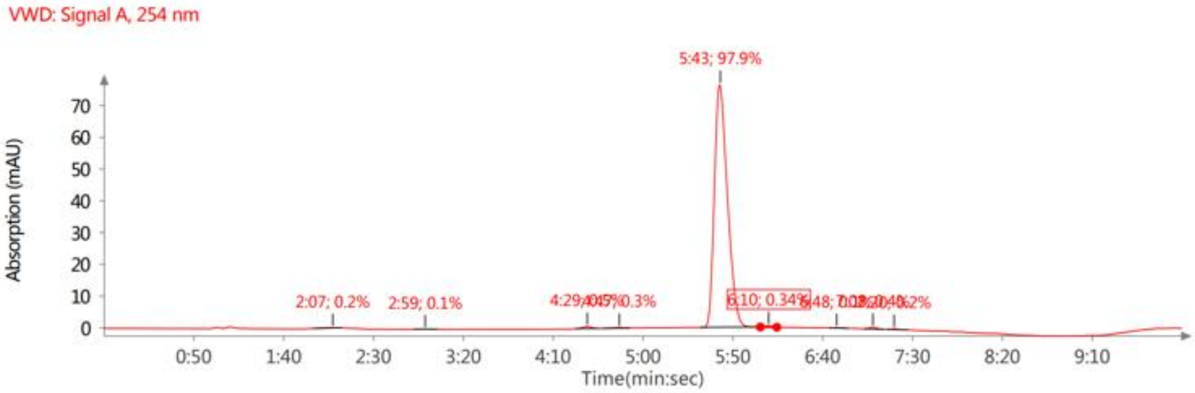

Figure S71. HPLC chromatogram of compound **9b**.

^{1}H NMR (400 MHz, [D$_6$]DMSO, 25°C, TMS): δ = 10.27 (s, 1H, Ar-N*H*-COR), 8.37 (d, *J* = 1.8 Hz, 1H, Ar-*H*$_7$), 7.88 (dd, *J*$_1$ = 8.5 Hz, *J*$_2$ = 1.9 Hz, 1H, Ar-*H*$_5$), 7.84 – 7.78 (m, 2H, 2 × Ar-*H*), 7.52 (d, *J* = 8.5 Hz, 1H, Ar-*H*$_4$), 7.44 – 7.37 (m, 2H, 2 × Ar-*H*), 3.73 – 3.46 (m, 4H, CH-N*H*$_2$ + 2 × pyrrolidine-*H*), 3.29 – 3.15 (m, 1H, pyrrolidine-*H*), 2.17 – 2.04 (m, 1H, pyrrolidine-*H*), 1.90 – 1.74 ppm (m, 3H, 3 × pyrrolidine-*H*)

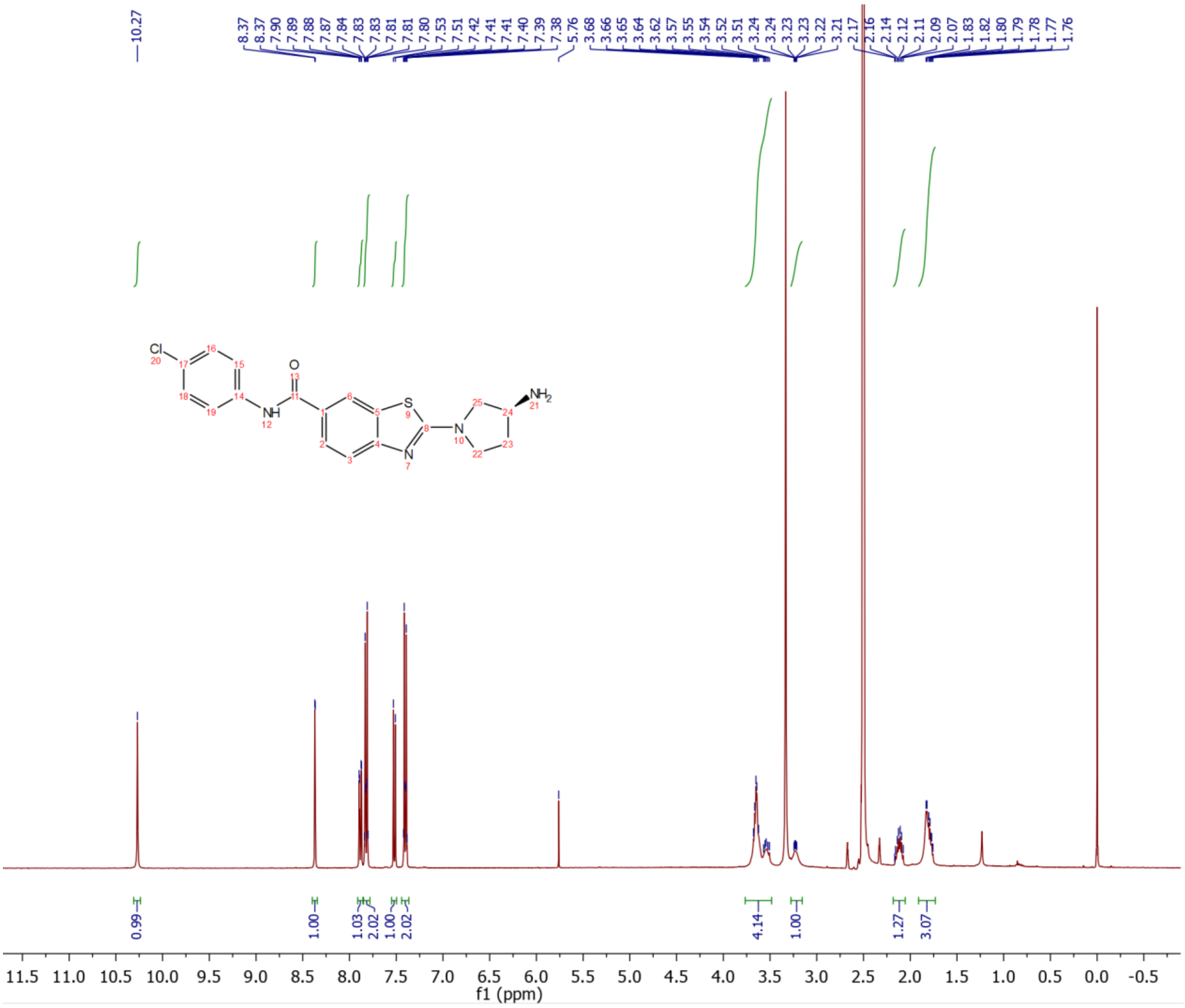

Figure S72. ^{1}H NMR spectrum of compound **9c**.

^{13}C (101 MHz, [D$_6$]DMSO, 25°C, TMS): δ = 167.0, 165.7, 156.4, 138.9, 130.9, 129.0 (2C), 127.4, 126.9, 126.5, 122.2 (2C), 121.7, 117.7, 58.3, 51.4, 48.5, 34.5 ppm

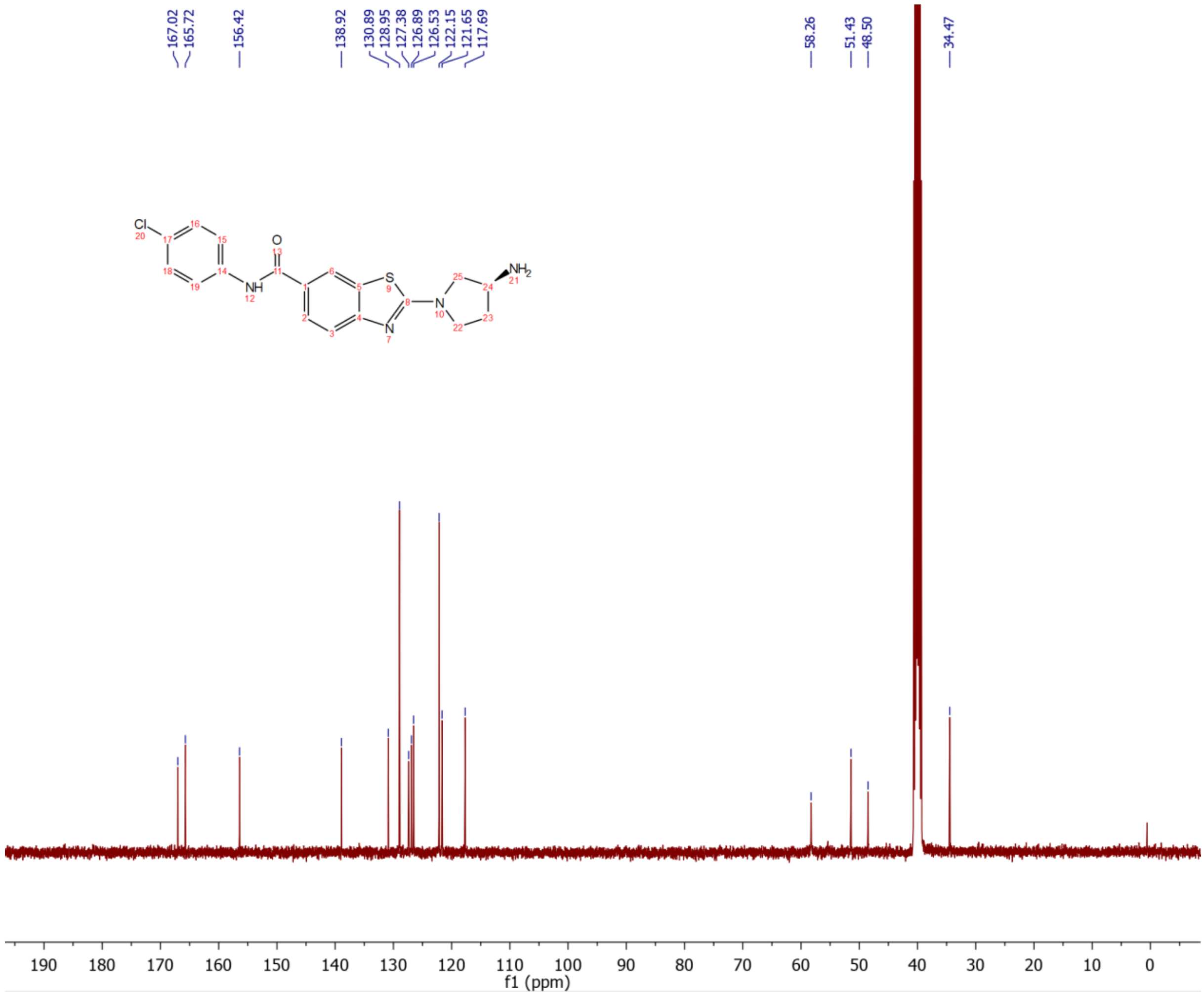

Figure S73. ^{13}C NMR spectrum of compound **9c**.

HPLC: t_r = 4.41 min (98.9 % at 254 nm).

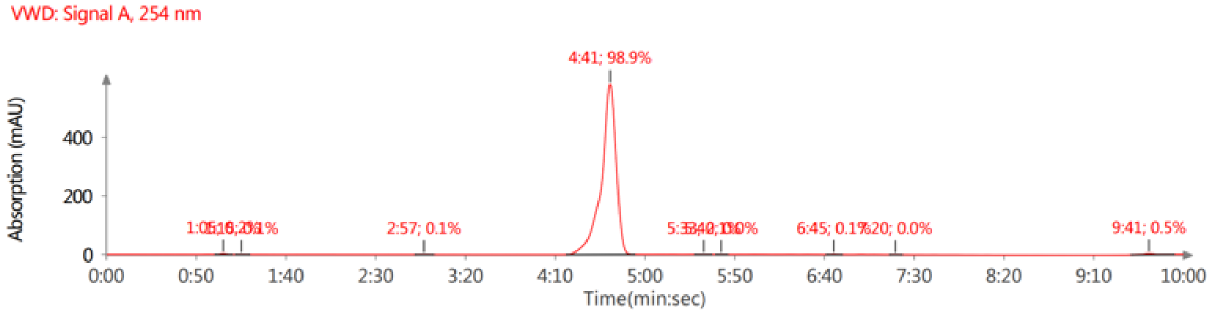

Figure S74. HPLC chromatogram of compound **9c**.

^{1}H NMR (400 MHz, [D$_6$]DMSO, 25°C, TMS): δ = 10.27 (s, 1H, Ar-N*H*-COR), 8.37 (d, *J* = 1.8 Hz, 1H, Ar-*H$_7$*), 7.88 (dd, *J$_1$* = 8.5 Hz, *J$_2$* = 1.9 Hz, 1H, Ar-*H$_5$*), 7.85 – 7.79 (m, 2H, 2 × Ar-*H*), 7.52 (d, *J* = 8.5 Hz, 1H, Ar-*H$_4$*), 7.44 – 7.37 (m, 2H, 2 × Ar-*H*), 3.73 – 3.48 (m, 4H, CH-N*H$_2$* and 2 × pyrrolidine-*H*), 3.27 – 3.18 (m, 1H, pyrrolidine-*H*), 2.16 – 2.07 (m, 1H, pyrrolidine-*H*), 1.96 – 1.74 ppm (m, 3H, 3 × pyrrolidine-*H*)

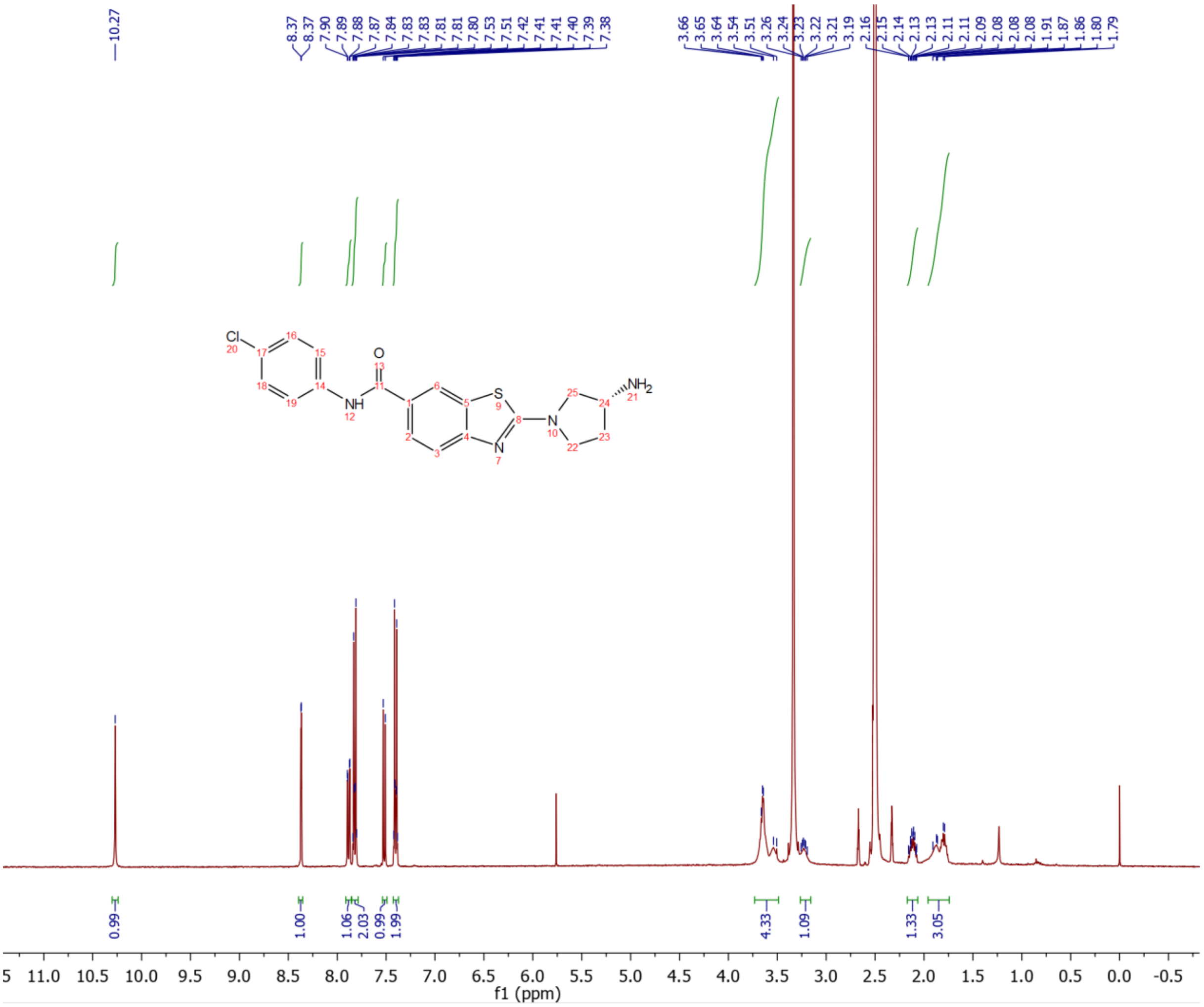

Figure S75. ^{1}H NMR spectrum of compound **9d**.

^{13}C (101 MHz, [D$_6$]DMSO, 25°C, TMS): δ = 167.0, 165.7, 156.4, 138.9, 130.9, 129.0 (2C), 127.4, 126.9, 126.5, 122.2 (2C), 121.7, 117.7, 58.2, 51.4, 48.5, 34.4 ppm

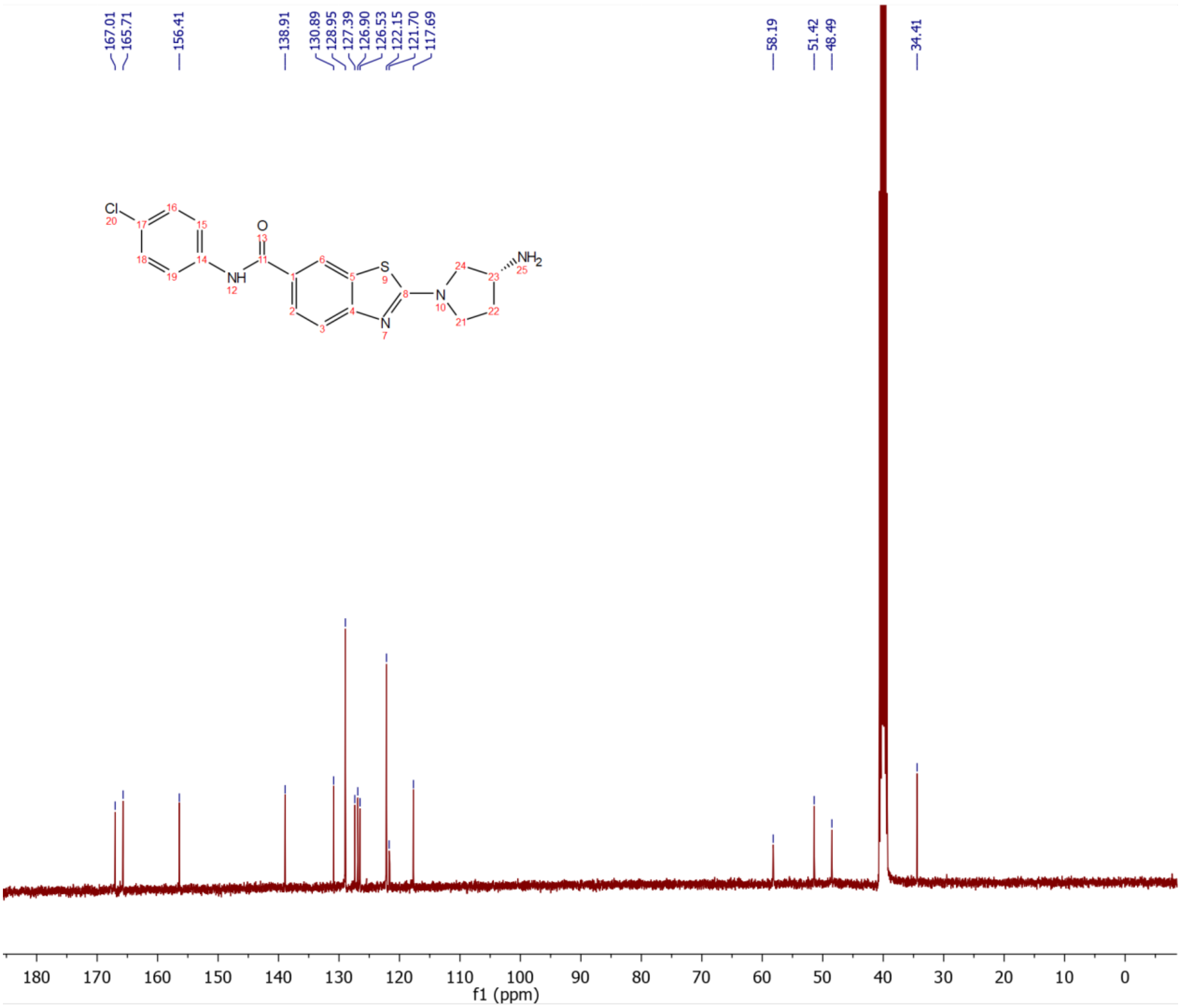

Figure S76. ^{13}C NMR spectrum of compound **9d**.

HPLC: t_r = 4.41 min (98.7 % at 254 nm)

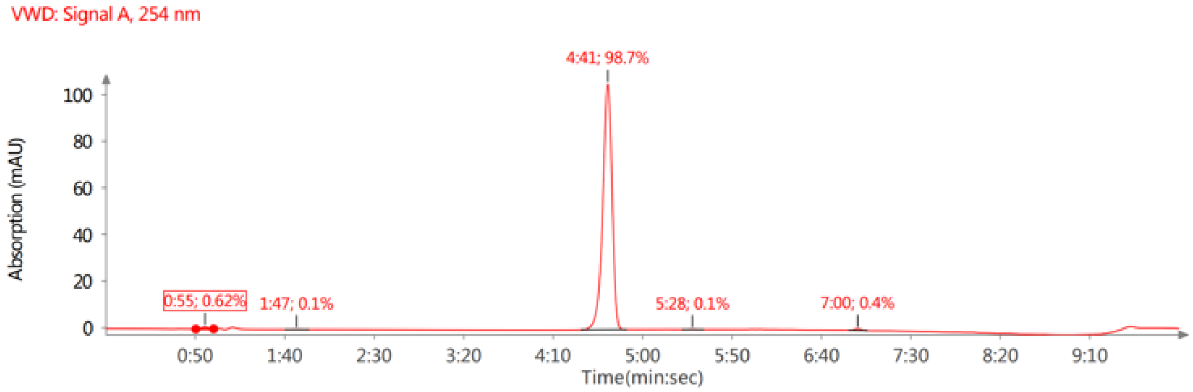

Figure S77. HPLC chromatogram of compound **9d**.

^{1}H NMR (400 MHz, [D$_6$]DMSO, 25°C, TMS): δ = 10.28 (s, 1H, Ar-N*H*-COR), 8.36 (d, *J* = 1.8 Hz, 1H, Ar-*H*$_7$), 7.89 (dd, J_1 = 8.5 Hz, J_2 = 1.8 Hz, 1H, Ar-*H*$_5$), 7.84 – 7.80 (m, 2H, 2 × Ar-*H*), 7.50 (d, *J* = 8.5 Hz, 1H, Ar-*H*$_4$), 7.43 – 7.38 (m, 2H, 2 × Ar-*H*), 4.13 – 4.05 (m, 2H, CH$_2$-N*H*$_2$), 3.19 (td, J_1 =12.7 Hz, J_2 = 2.6 Hz, 2H, CH-C*H*$_2$-NH$_2$), 2.47 – 2.44 (m, 2H, 2 × piperidine-*H*), 1.87 – 1.80 (m, 2H, 2 × piperidine-*H*), 1.59 – 1.40 (m, 3H, 3 × piperidine-*H*), 1.26 – 1.12 ppm (m, 2H, 2 × piperidine-*H*)

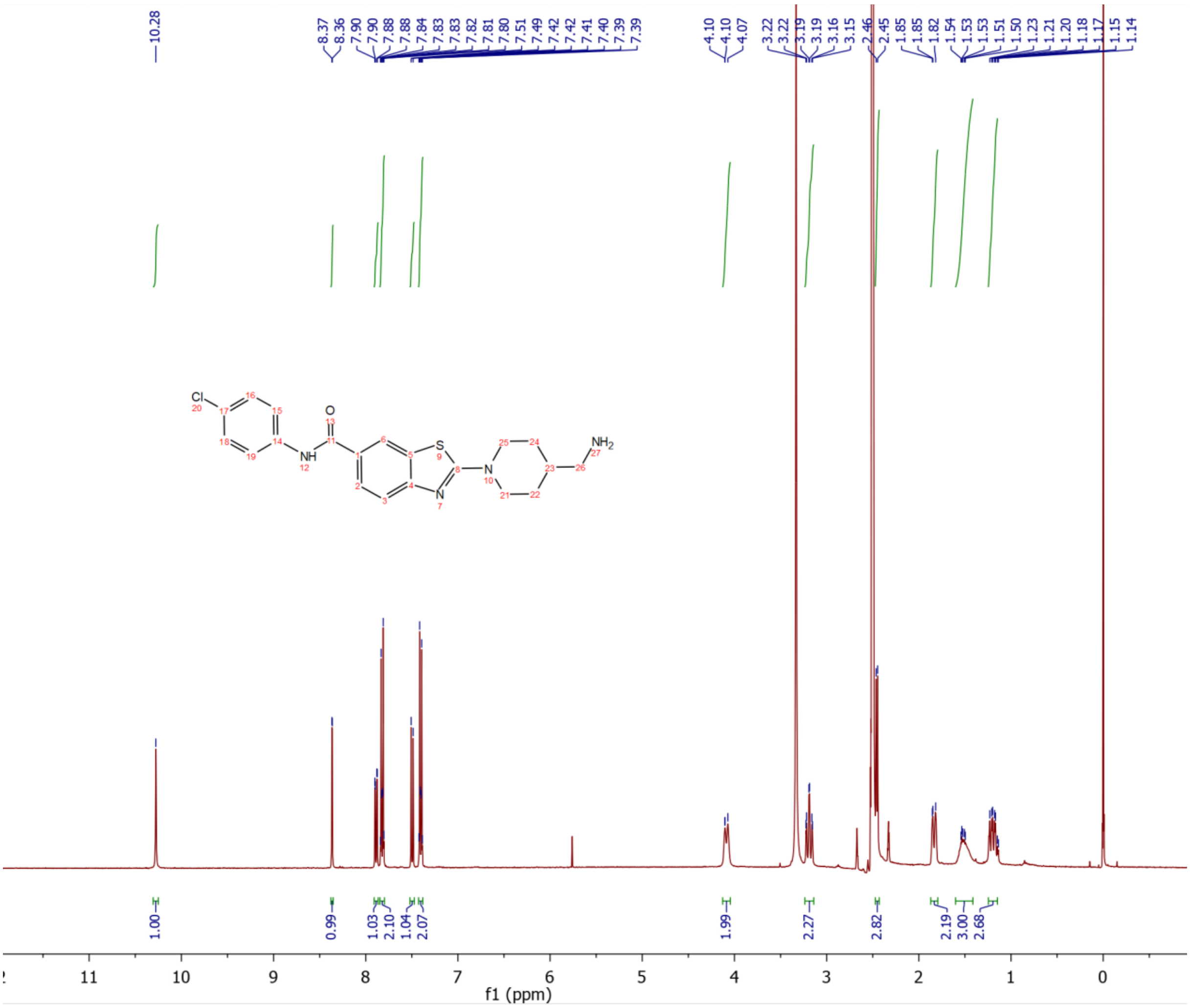

Figure S78. ^{1}H NMR spectrum of compound **9e**.

^{13}C (101 MHz, [D$_6$]DMSO, 25°C, TMS): δ = 170.2, 165.6, 156.1, 138.9, 130.8, 129.0 (2C), 127.4, 127.4, 126.6, 122.2 (2C), 121.5, 117.9, 49.0 (2C), 47.7, 29.5 ppm (3C)

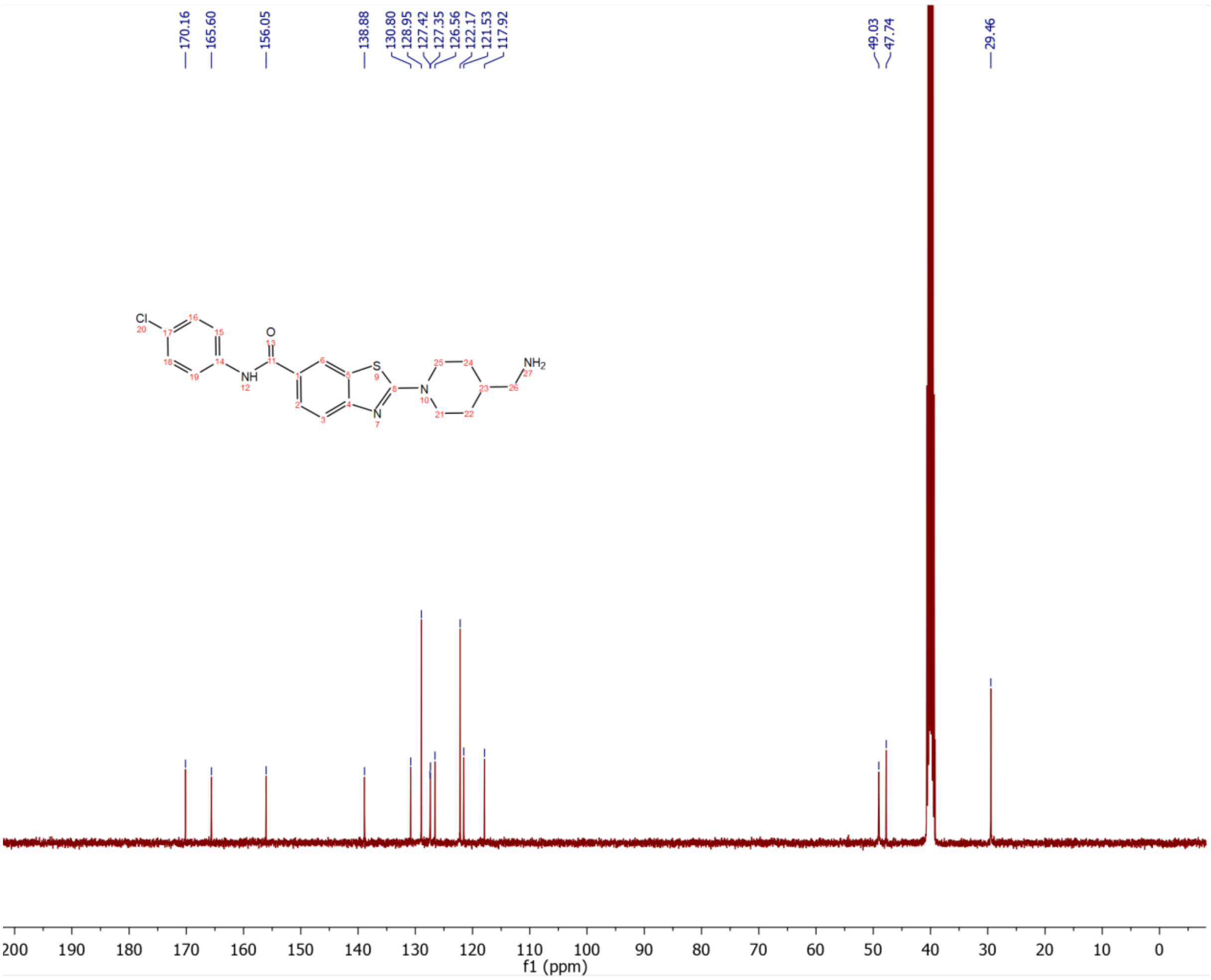

Figure S79. ^{13}C NMR spectrum of compound **9e**.

HPLC: t_r = 5.49 min (97.7 % at 254 nm)

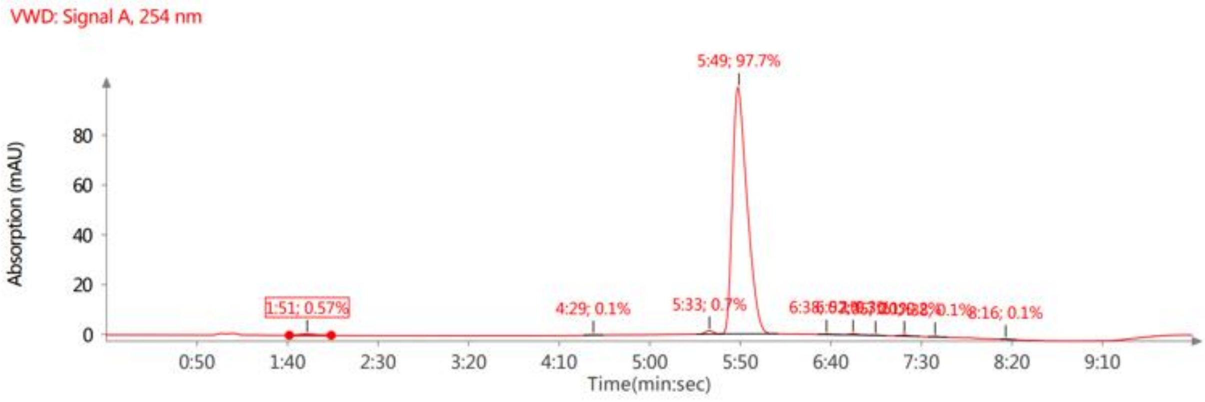

Figure S80. HPLC chromatogram of compound **9e**.

^{1}H NMR (400 MHz, [D$_6$]DMSO, 25°C, TMS): δ = 10.24 (s, 1H, Ar-N*H*-COR), 8.34 (d, *J* = 7.3 Hz, 1H, Ar-N*H*-CH), 8.27 (d, *J* = 1.7 Hz, 1H, Ar-*H*), 7.87 – 7.79 (m, 3H, 3 × Ar-*H*), 7.45 (d, *J* = 8.4 Hz, 1H, Ar-*H*), 7.42 – 7.37 (m, 2H, 2 × Ar-*H*), 3.82 – 3.76 (m, 1H, piperidine-*H*), 2.99 – 2.90 (m, 2H, 2 × piperidine-*H*), 1.98 – 1.88 (m, 2H, 2 × piperidine-*H*), 1.34 ppm (ddd, J_1 = 14.9 Hz, J_2 = 11.7 Hz, J_3 = 3.8 Hz, 2H, 2 × piperidine-*H*), not visible (2 × piperidine-*H* and N*H*)

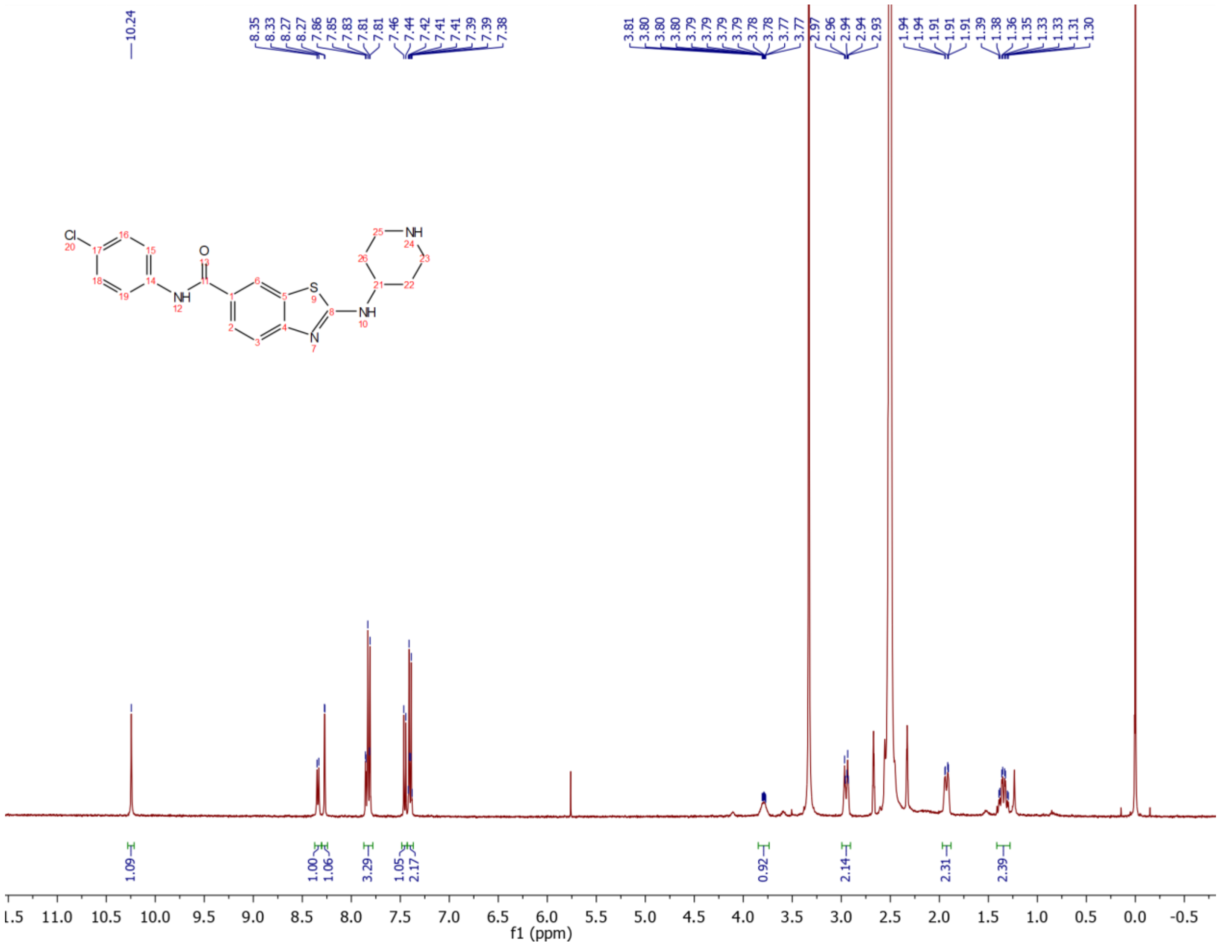

Figure S81. ^{1}H NMR spectrum of compound **9f**.

^{13}C (101 MHz, [D$_6$]DMSO, 25°C, TMS): δ = 167.6, 165.7, 156.1, 138.9, 130.7, 128.9 (2C), 127.4, 127.2, 126.2, 122.1 (2C), 121.3, 117.5, 52.5, 45.3 (2C), 33.4 ppm (2C)

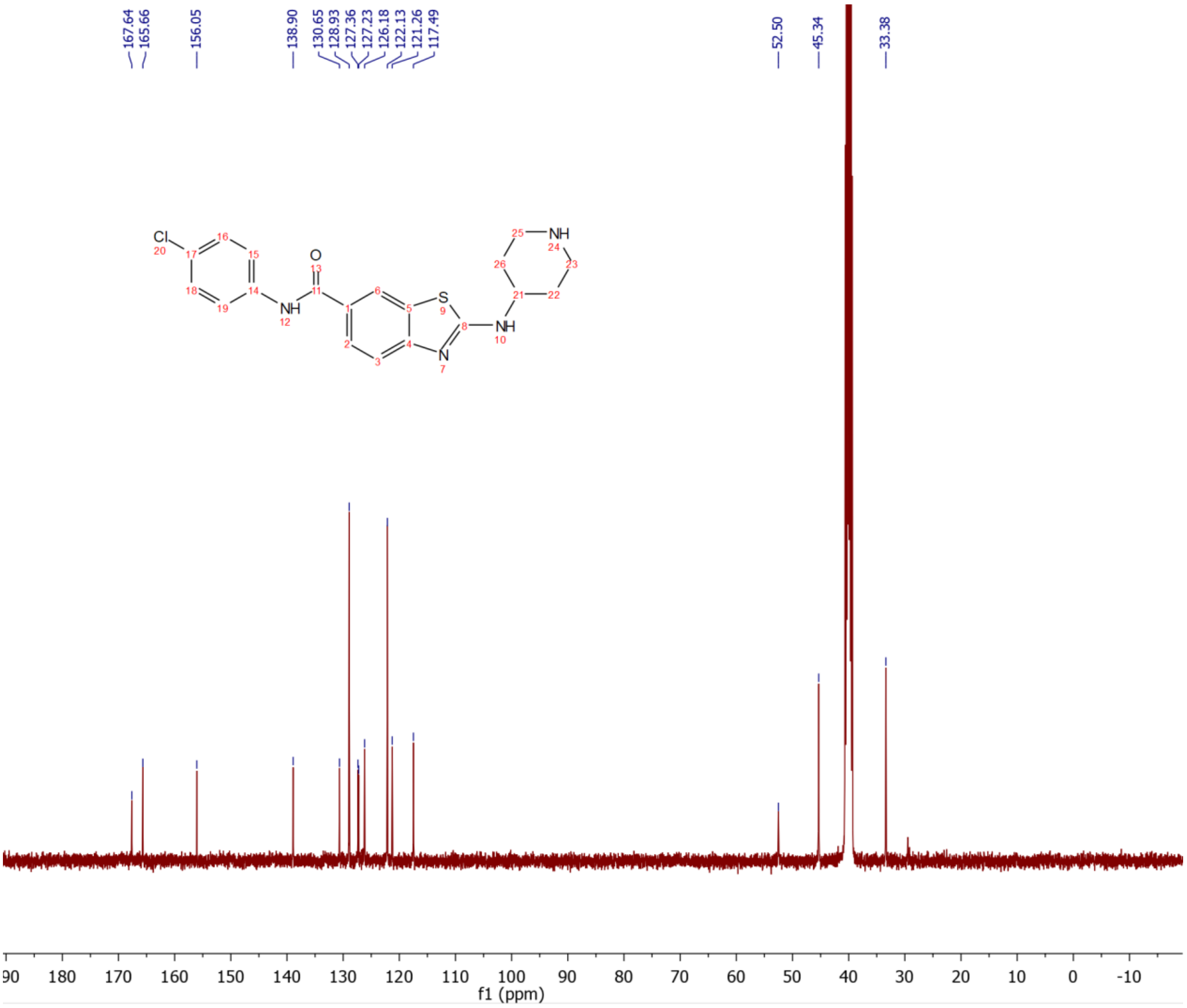

Figure S82. ^{13}C NMR spectrum of compound **9f**.

HPLC: t_r = 4.45 min (95.1 % at 254 nm)

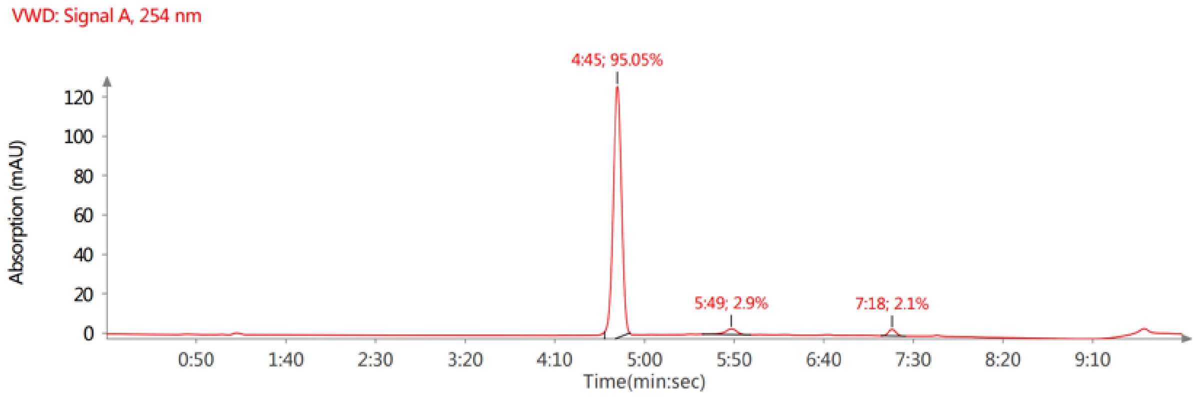

Figure S83. HPLC chromatogram of compound **9f**.

^{1}H NMR (400 MHz, [D_6]DMSO, 25°C, TMS): δ = 10.27 (s, 1H, Ar-N*H*-COR), 8.39 (d, *J* = 1.8 Hz, 1H, Ar-H_7), 7.89 (dd, J_1 = 8.5 Hz, J_2 = 1.8 Hz, 1H, Ar-H_5), 7.84 – 7.79 (m, 2H, 2 × Ar-*H*), 7.52 (d, *J* = 8.5 Hz, 1H, Ar-H_4), 7.43 – 7.37 (m, 2H, 2 × Ar-*H*), 3.69 – 3.40 (m, 4H, 2 × CH_2), 2.94 – 2.86 (m, 2H, CH_2), 2.81 – 2.70 (m, 2H, CH_2), 2.07 – 1.97 (m, 2H, CH_2), 1.81 – 1.68 ppm (m, 2H, CH_2), not visible (N*H*)

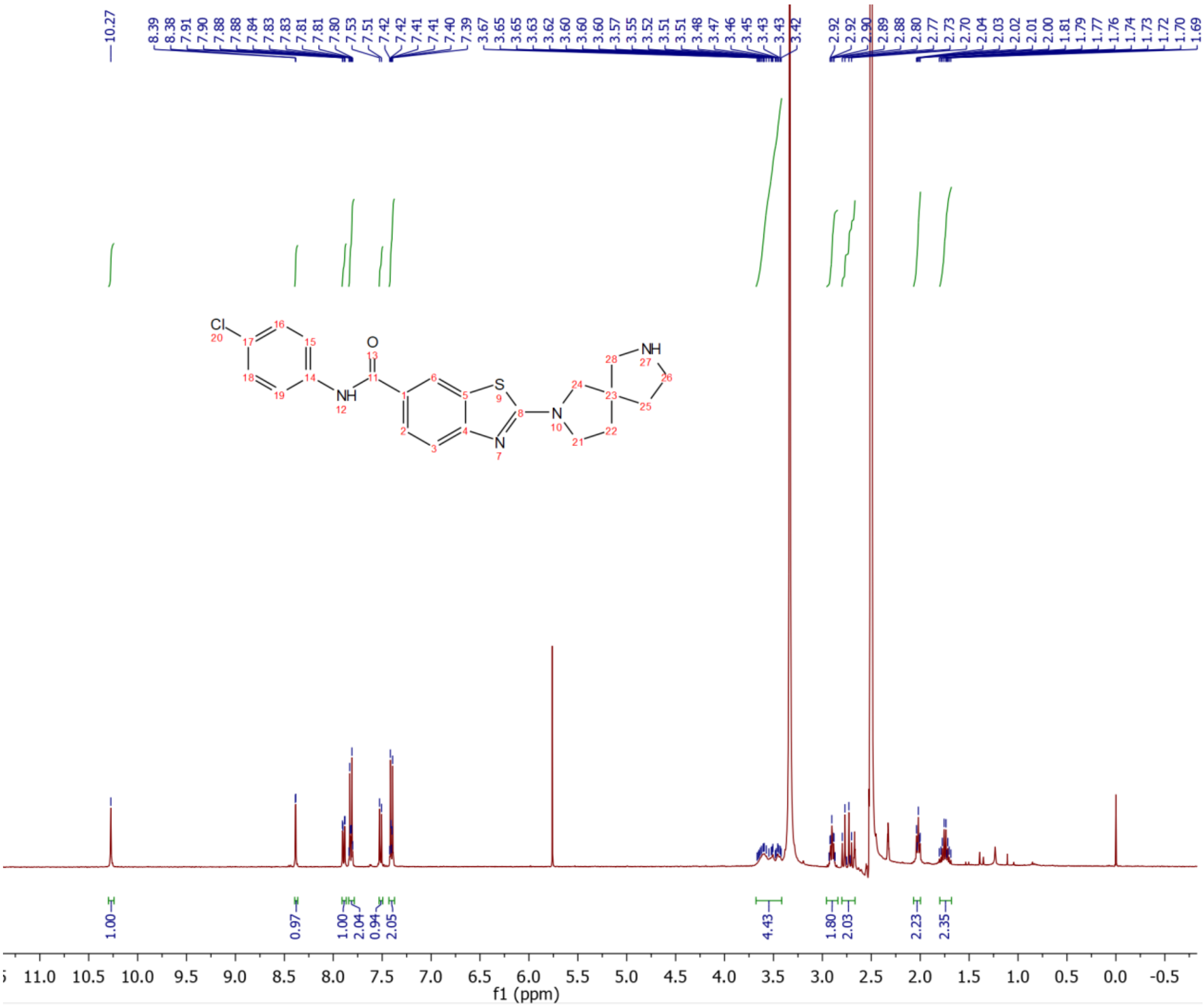

Figure S84. ^{1}H NMR spectrum of compound **9g**.

^{13}C (101 MHz, [D$_6$]DMSO, 25°C, TMS): δ = 166.9, 165.7, 156.3, 138.9, 130.9, 128.9 (2C), 127.4, 127.0, 126.6, 122.2 (2C), 121.7, 117.8, 59.3, 55.6, 49.9, 49.4, 45.8, 35.9, 35.5 ppm

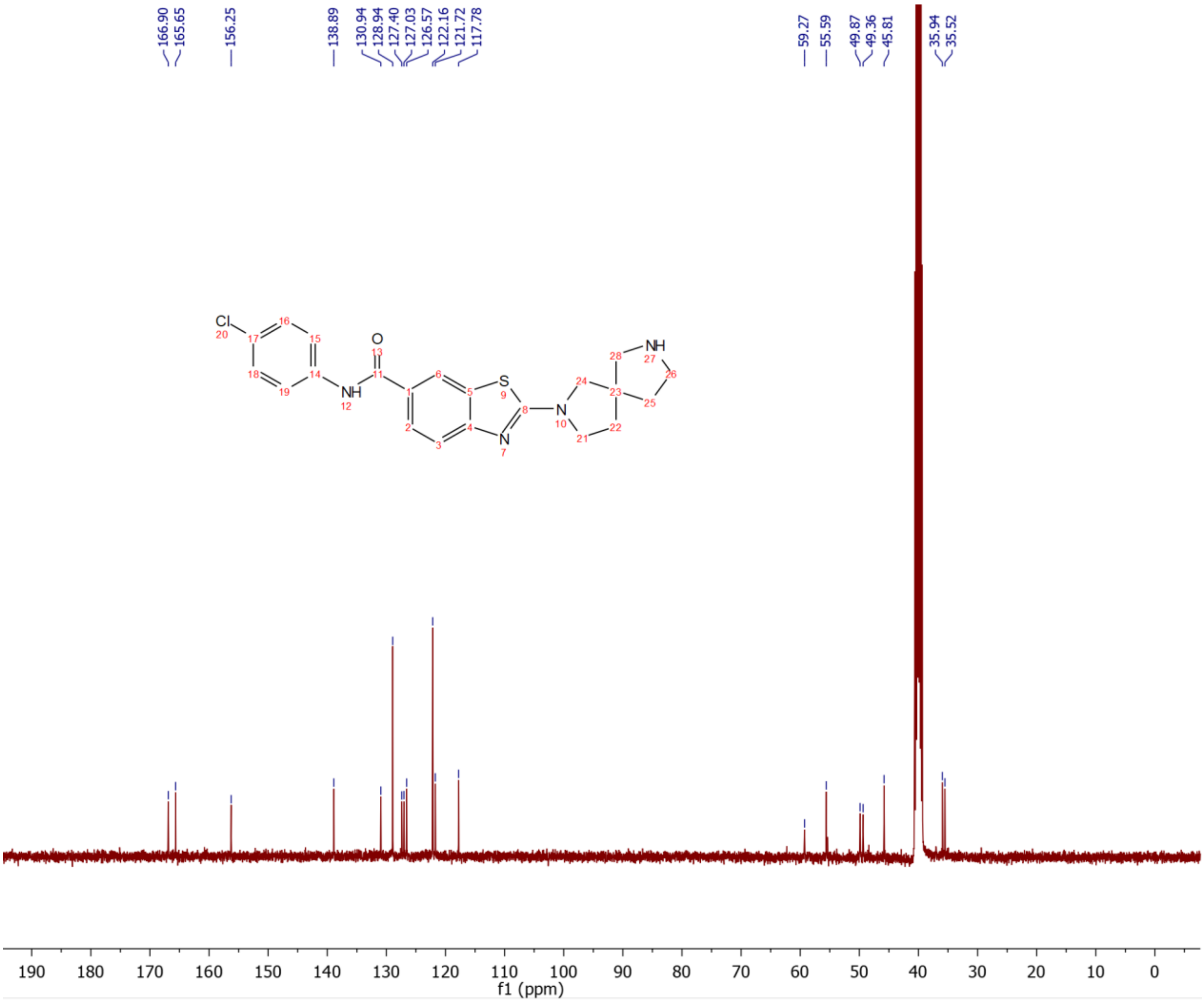

Figure S85. ^{13}C NMR spectrum of compound **9g**.

HPLC: t_r = 4.59 min (97.0 % at 254 nm)

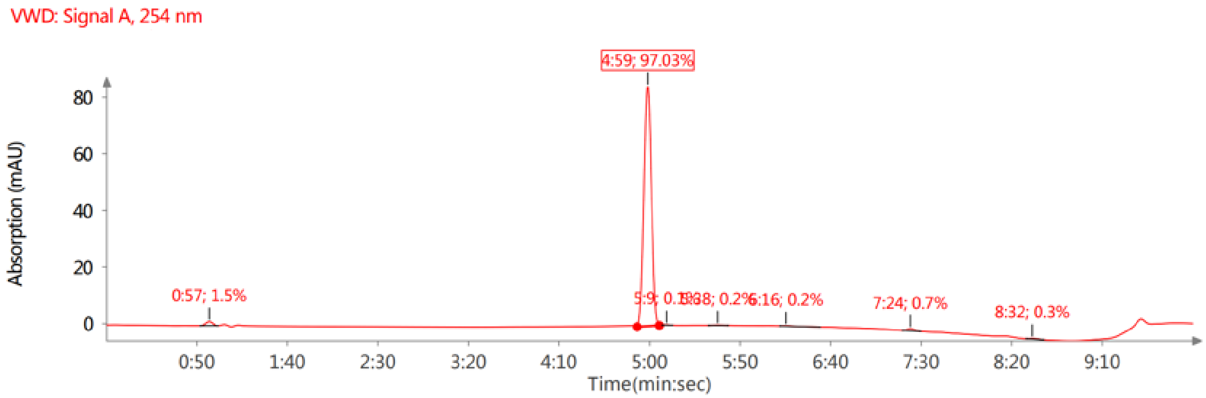

Figure S86. HPLC chromatogram of compound **9g**.

^{1}H NMR (400 MHz, [D$_6$]DMSO, 25°C, TMS): δ = 10.28 (s, 1H, Ar-N*H*-COR), 8.38 (d, *J* = 1.9 Hz, 1H, Ar-*H*$_7$), 7.89 (dd, *J*$_1$ = 8.5 Hz, *J*$_2$ = 1.9 Hz, 1H, Ar-*H*$_5$), 7.85 – 7.79 (m, 2H, 2 × Ar-*H*), 7.53 (d, *J* = 8.5 Hz, 1H, Ar-*H*$_4$), 7.43 – 7.38 (m, 2H, 2 × Ar-*H*), 3.82 – 3.75 (m, 2H, 2 × C*H*), 3.39 – 3.37 (m, 2H, 2 × C*H*), 2.96 – 2.88 (m, 4H, 4 × C*H*), 2.74 – 2.69 ppm (m, 2H, 2 × C*H*), not visible (N*H*)

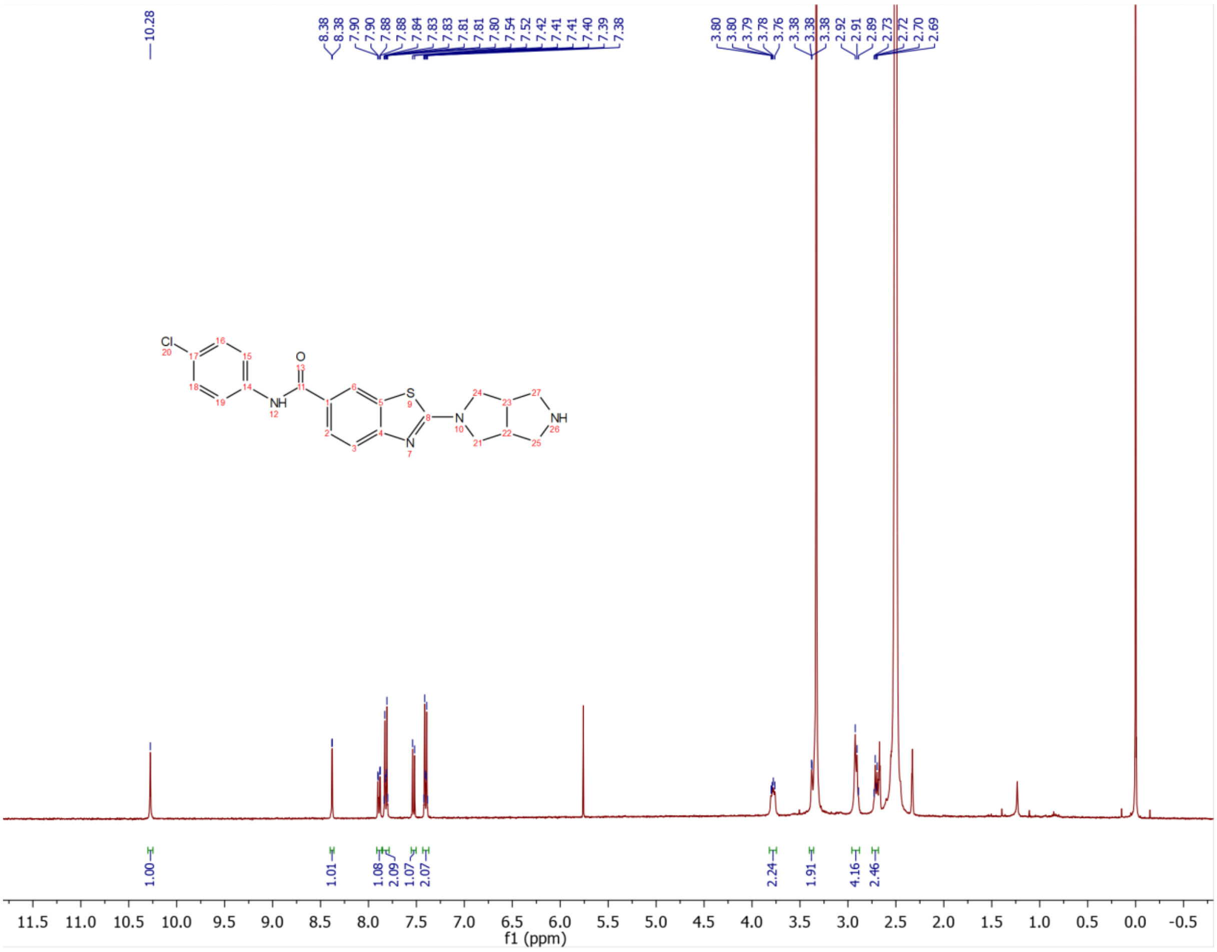

Figure S87. ^{1}H NMR spectrum of compound **9h**.

^{13}C (101 MHz, [D$_6$]DMSO, 25°C, TMS): δ = 166.6, 165.7, 156.2, 138.9, 131.1, 128.9 (2C), 127.4, 127.1, 126.5, 122.2 (2C), 121.7, 117.9, 55.3 (2C), 53.2 (2C), 43.8 ppm (2C)

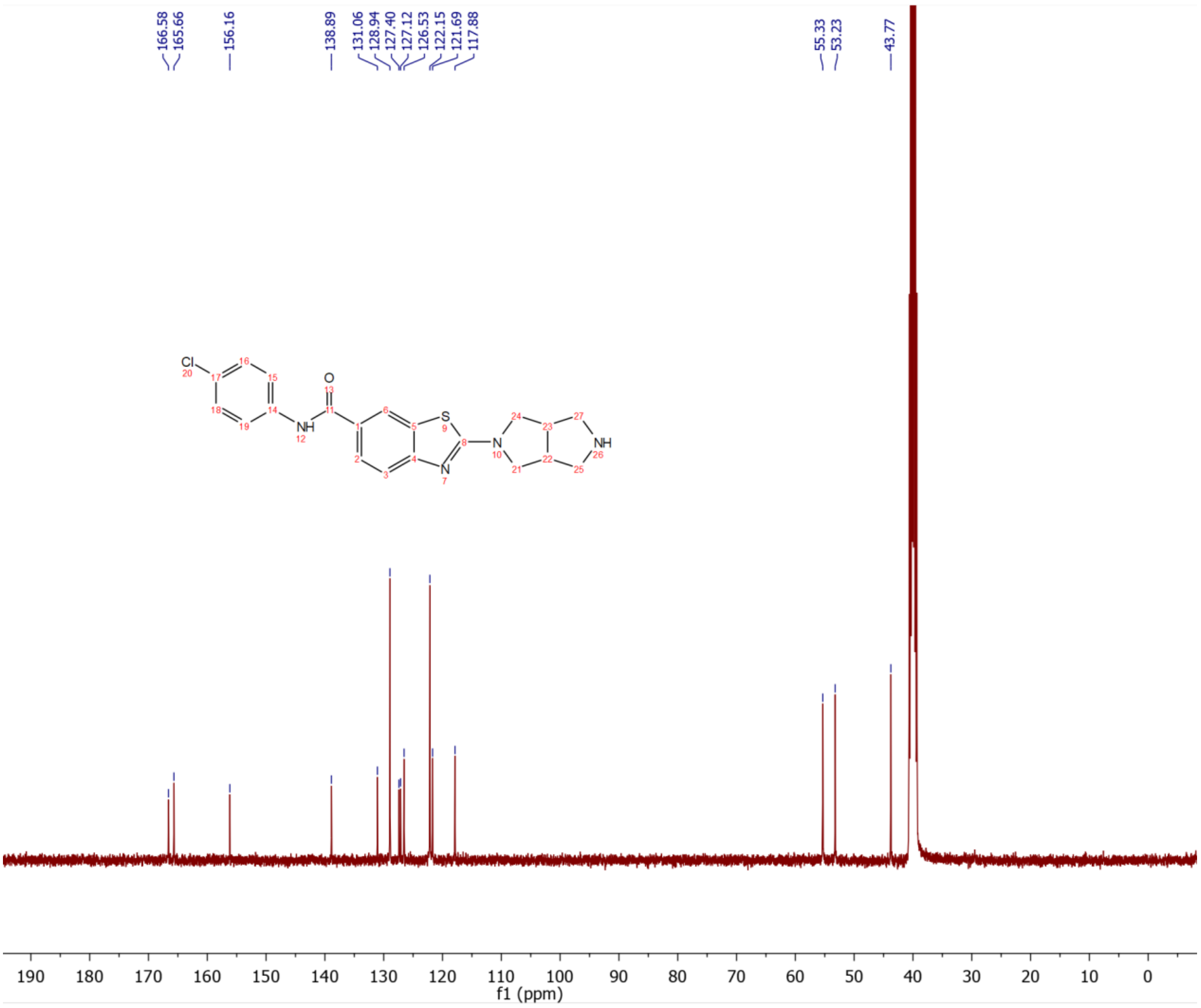

Figure S88. ^{13}C NMR spectrum of compound **9h**.

HPLC: t_r = 4.47 min (98.8 % at 254 nm)

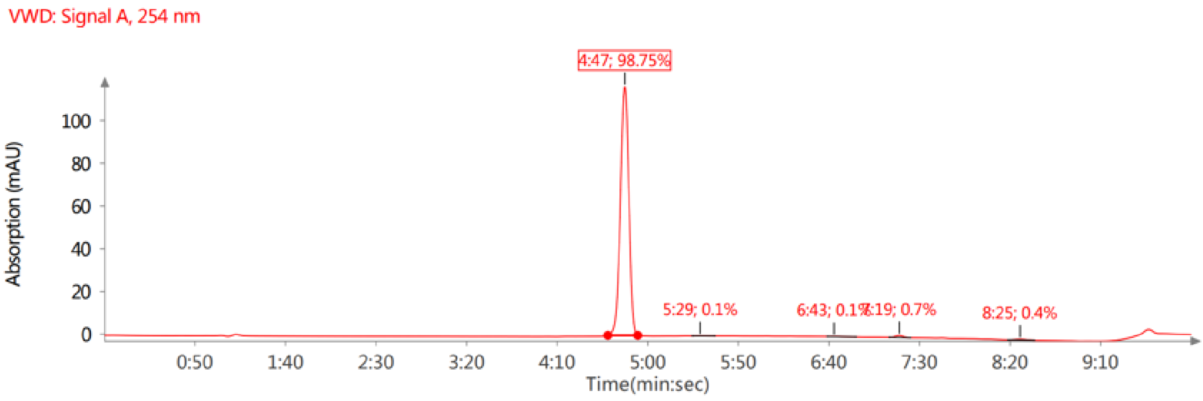

Figure S89. HPLC chromatogram of compound **9h**.

^{1}H NMR (400 MHz, [D$_6$]DMSO, 25°C, TMS): δ = 10.28 (s, 1H, Ar-N*H*-COR), 8.37 (d, *J* = 1.8 Hz, 1H, Ar-*H$_7$*), 7.89 (dd, J_1 = 8.5 Hz, J_2 = 1.8 Hz, 1H, Ar-*H$_5$*), 7.85 – 7.79 (m, 2H, 2 × Ar-*H*), 7.50 (d, *J* = 8.5 Hz, 1H, Ar-*H$_4$*), 7.43 – 7.37 (m, 2H, 2 × Ar-*H*), 3.99 (d, *J* = 13.1 Hz, 2H, CH-N*H$_2$*), 3.30 – 3.23 (m, 2H, 2 × piperidine-*H*), 2.91 – 2.83 (m, 1H, piperidine-*H*), 1.87 – 1.64 (m, 4H, 4 × piperidine-*H*), 1.37 – 1.23 ppm (m, 2H, 2 × piperidine-*H*)

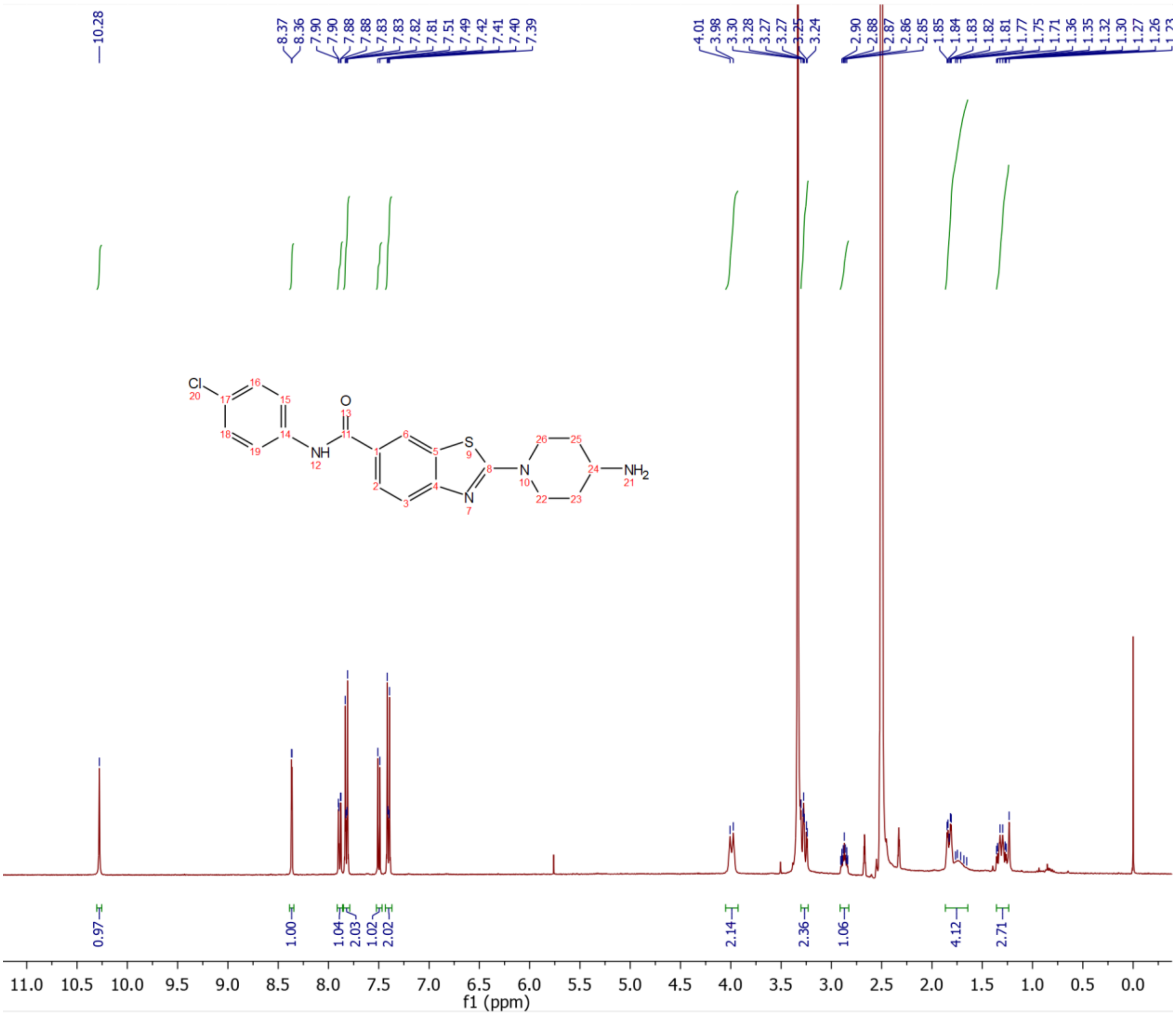

Figure S90. ^{1}H NMR spectrum of compound **9i**.

^{13}C (101 MHz, [D$_6$]DMSO, 25°C, TMS): δ = 170.1, 165.6, 156.1, 138.9, 130.9, 128.9 (2C), 127.4, 127.4, 126.6, 122.2 (2C), 121.5, 117.9, 48.0, 47.6 (2C), 34.8 ppm (2C)

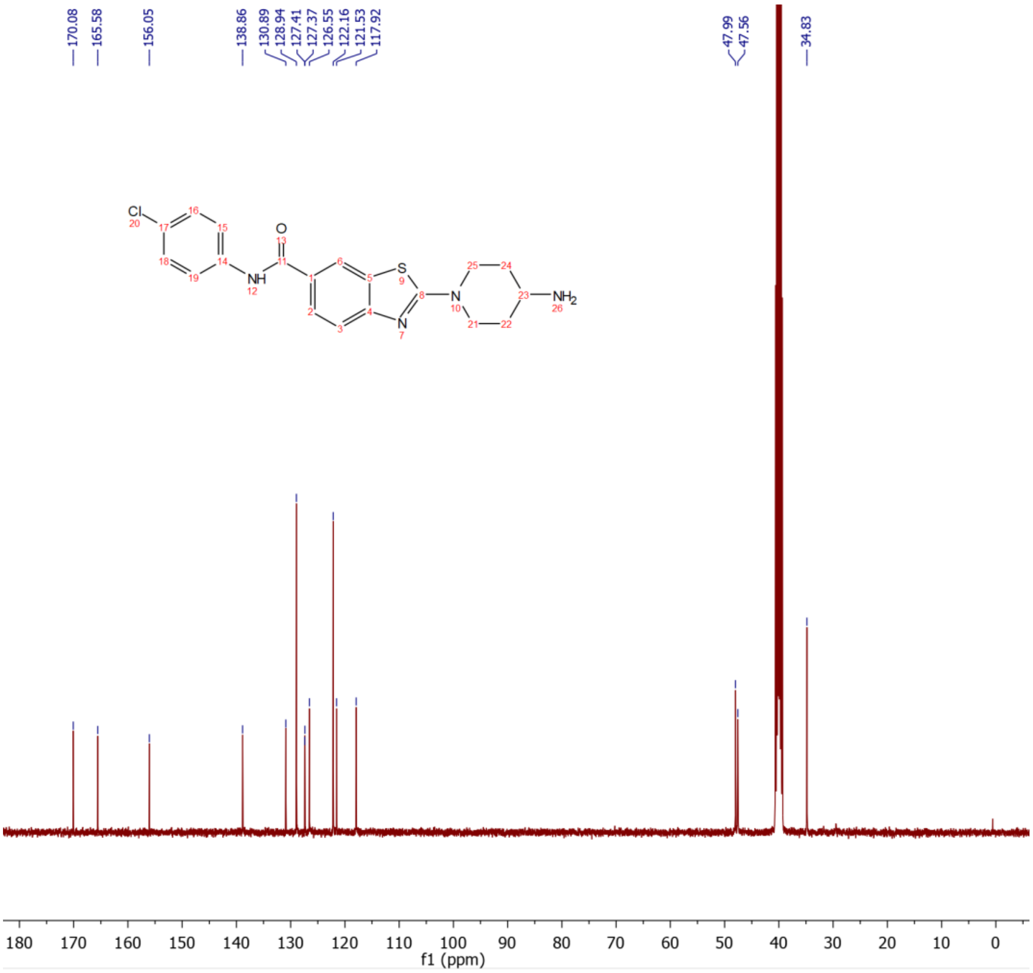

Figure S91. ^{13}C NMR spectrum of compound **9i**.

HPLC: t_r = 4.54 min (98.9 % at 254 nm)

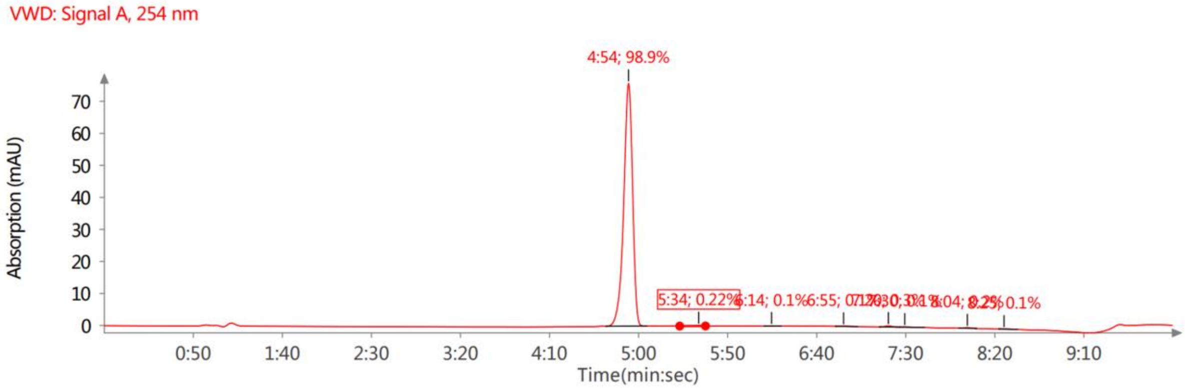

Figure S92. HPLC chromatogram of compound **9i**.

^{1}H NMR (400 MHz, [D$_6$]DMSO, 25°C, TMS): δ = 10.28 (s, 1H, Ar-N*H*-COR), 8.37 (d, *J* = 1.8 Hz, 1H, Ar-*H*$_7$), 7.89 (dd, J_1 = 8.5 Hz, J_2 = 1.8 Hz, 1H, Ar-*H*$_5$), 7.85 – 7.78 (m, 2H, 2 × Ar-*H*), 7.50 (d, *J* = 8.5 Hz, 1H, Ar-*H*$_4$), 7.44 – 7.37 (m, 2H, 2 × Ar-*H*), 3.93 – 4.00 (m, 2H, 2 × piperidine-*H*), 3.32 – 3.26 (m, 2H, 2 × piperidine-*H*), 2.63 – 2.54 (m, 1H, piperidine-*H*), 2.30 (s, 3H, NH-C*H*$_3$), 1.97 – 1.86 (m, 2H, 2 × piperidine-*H*), 1.83 – 1.67 (m, 1H, N*H*), 1.39 – 1.28 ppm (m, 2H, 2 × piperidine-*H*)

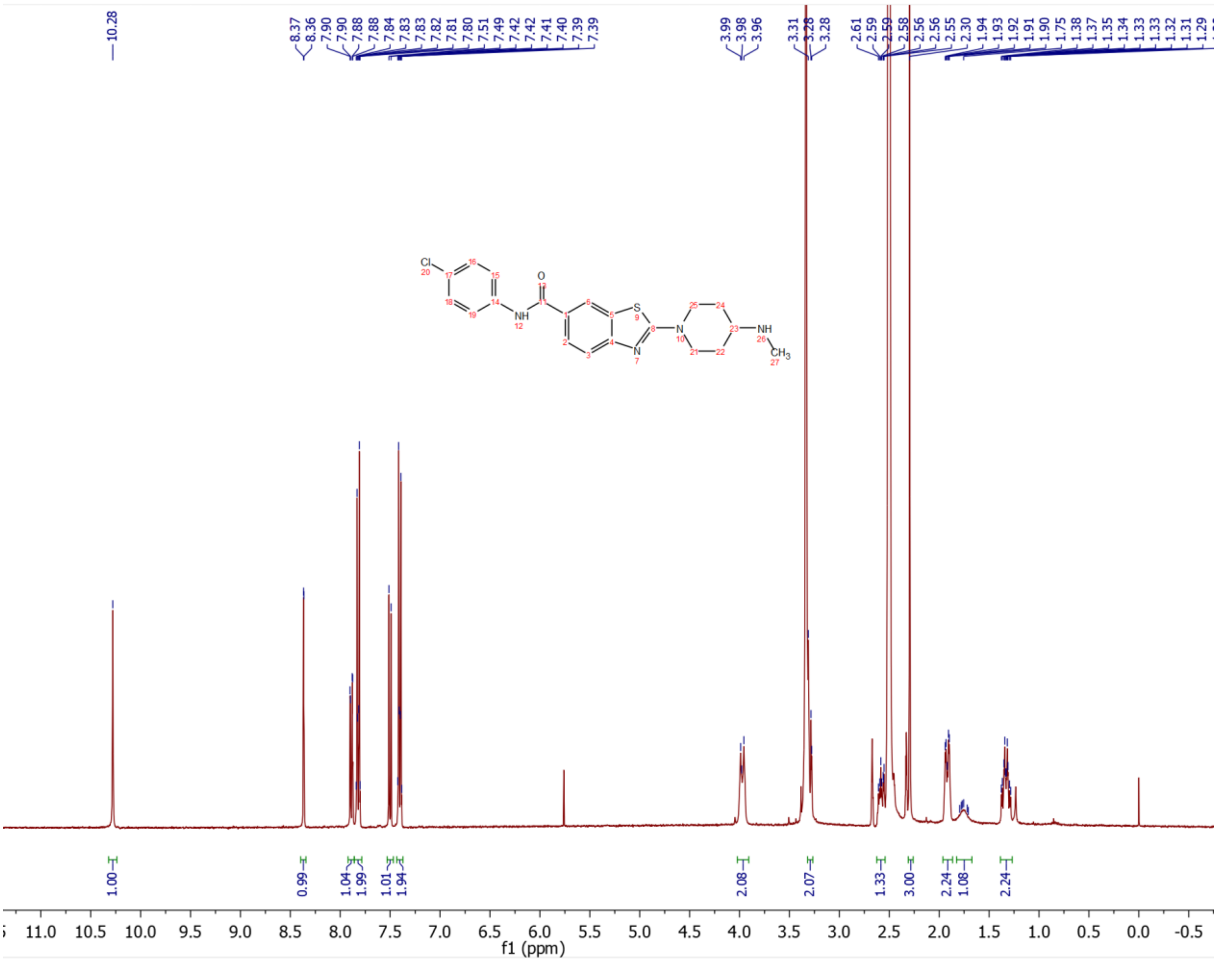

Figure S93. ^{1}H NMR spectrum of compound **9j**.

^{13}C (101 MHz, [D_6]DMSO, 25°C, TMS): δ = 170.1, 165.6, 156.0, 138.9, 130.9, 128.9 (2C), 127.4, 127.4, 126.6, 122.2 (2C), 121.5, 118.0, 55.7, 47.3 (2C), 33.7, 31.3 ppm (2C)

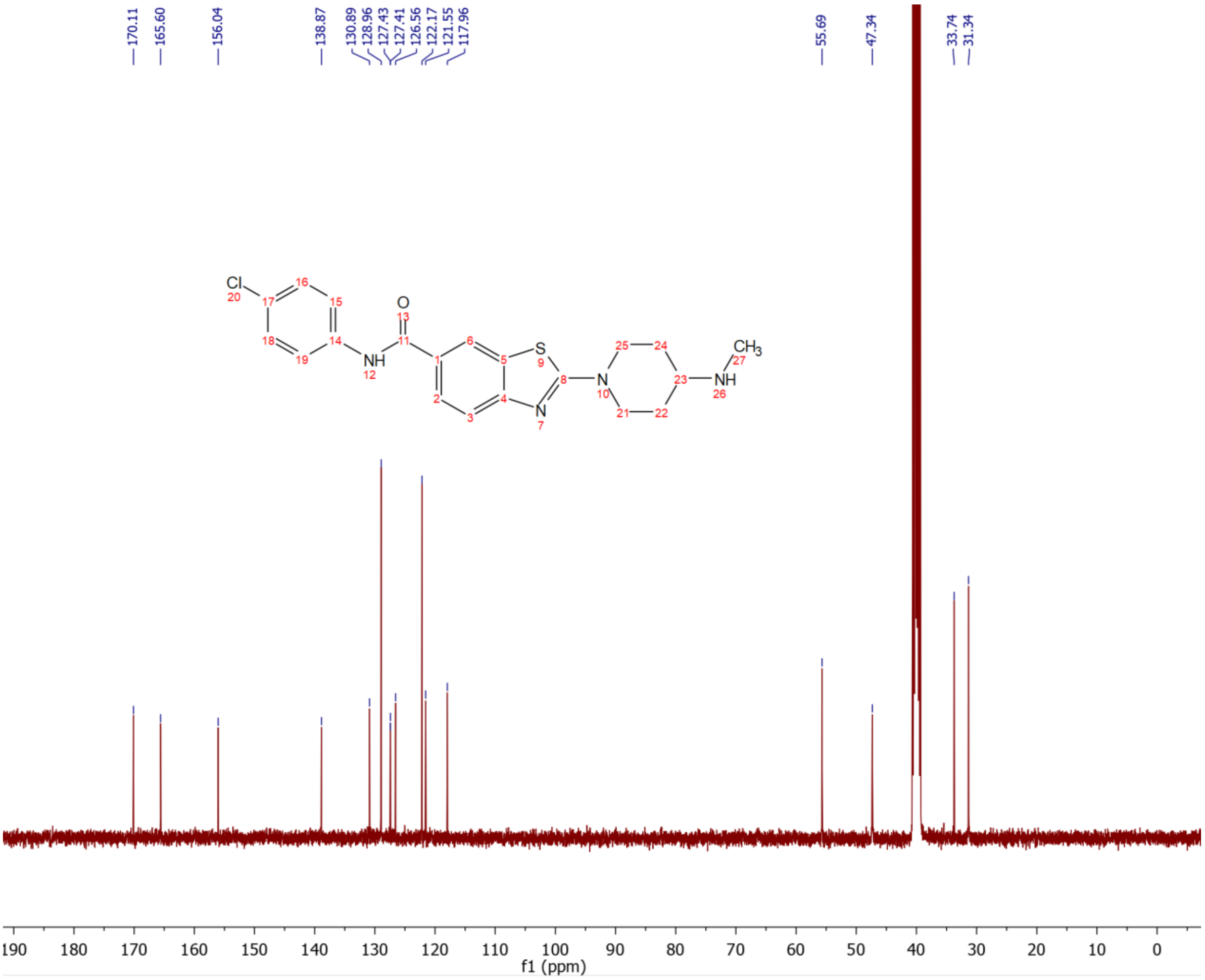

Figure S94. ^{13}C NMR spectrum of compound **9j**.

HPLC: t_r = 4.53 min (99.6 % at 254 nm)

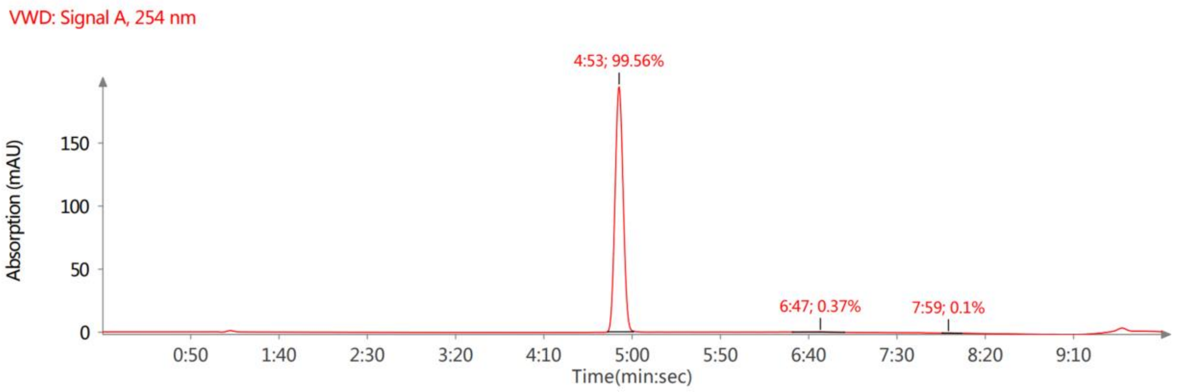

Figure S95. HPLC chromatogram of compound **9j**.

^{1}H NMR (400 MHz, [D_6]DMSO, 25°C, TMS): δ = 10.29 (s, 1H, Ar-N*H*-COR), 8.38 (d, *J* = 1.9 Hz, 1H, Ar-H_7), 7.90 (dd, J_1 = 8.5 Hz, J_2 = 1.9 Hz, 1H, Ar-H_5), 7.88 – 7.78 (m, 2H, 2 × Ar-*H*), 7.51 (d, *J* = 8.5 Hz, 1H, Ar-H_4), 7.44 – 7.37 (m, 2H, 2 × Ar-*H*), 4.08 (d, *J* = 13 Hz, 2H, 2 × piperidine-*H*), 3.29 – 3.16 (m, 2 × piperidine-*H*), 2.20 (s, 6H, N(CH_3)$_2$), 1.92 – 1.84 (m, 2H, 2 × piperidine-*H*), 1.55 – 1.42 ppm (m, 2H, 2 × piperidine-*H*), covered with solvent (1H, piperidine-*H*)

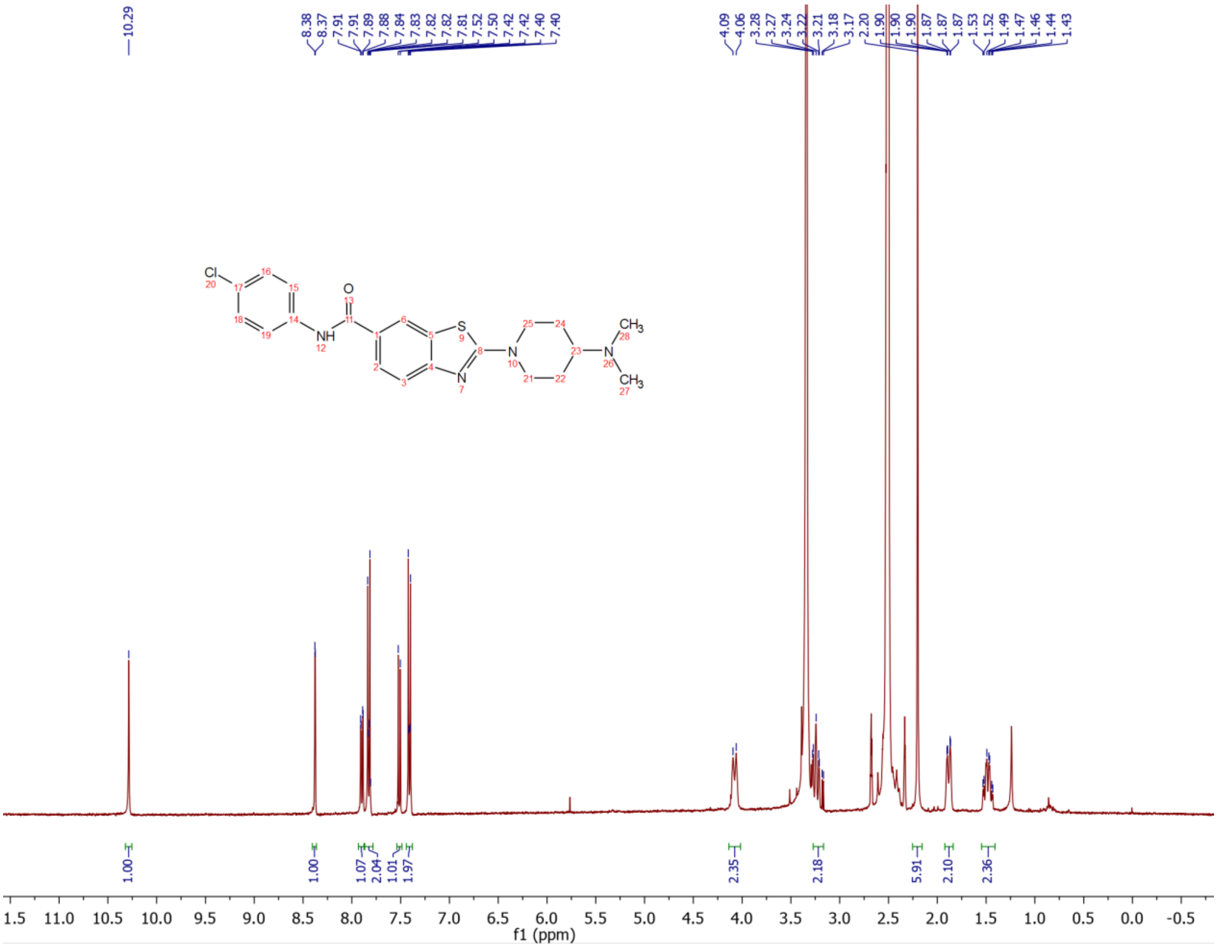

Figure S96. ^{1}H NMR spectrum of compound **10**.

^{13}C (101 MHz, [D$_6$]DMSO, 25°C, TMS): δ = 170.0, 165.6, 156.0, 138.9, 130.9, 128.9 (2C), 127.5, 127.4, 126.6, 122.2 (2C), 121.6, 118.0, 61.1, 48.0 (2C), 41.9 (2C), 27.9 ppm (2C)

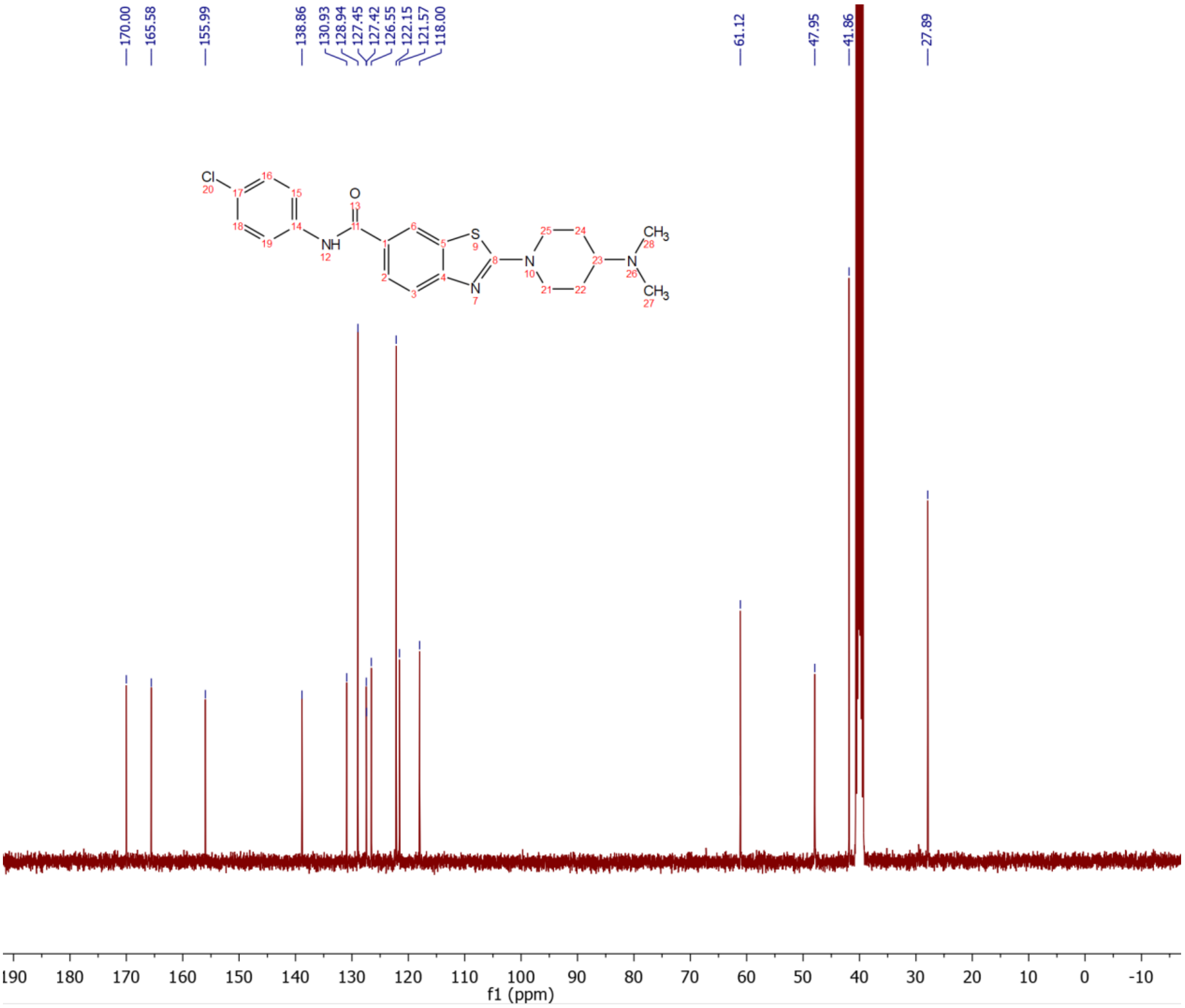

Figure S97. ^{13}C NMR spectrum of compound **10**.

HPLC: t_r = 5.02 min (96.9 % at 254 nm)

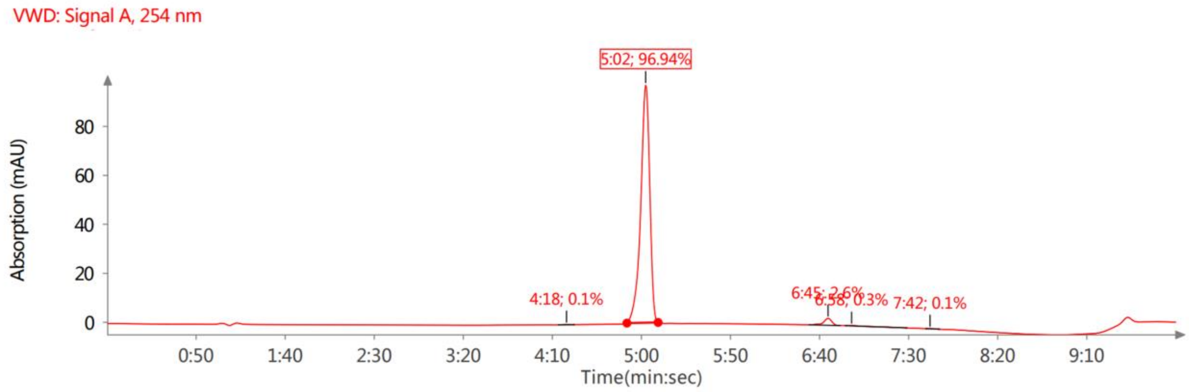

Figure S98. HPLC chromatogram of compound **10**.

^{1}H NMR (400 MHz, [D_6]DMSO, 25°C, TMS): δ = 10.47 (s, 1H, Ar-N*H*-COR), 8.40 (d, *J* = 1.8 Hz, 1H, Ar-*H*7), 8.19 (d, *J* = 2.4 Hz, 1H, Ar-*H*20), 7.91 (dd, J_1 = 8.5, J_2 = 1.8 Hz, 1H, Ar-*H*5), 7.79 (dd, *J* = 8.9, 2.4 Hz, 1H, Ar-*H*24), 7.61 (d, *J* = 8.9 Hz, 1H, Ar-*H*23), 7.50 (d, *J* = 8.5 Hz, 1H, Ar-*H*4), 3.99 (d, *J* = 13.2 Hz, 2H, CH-N*H*2), 3.33 – 3.20 (m, 2H, 2 × piperidin-*H*) 2.91 – 2.83 (m, 1H, C*H*-NH_2), 1.88 – 1.78 (m, 2H, 2 × piperidin-*H*), 1.88 – 1.58 (m, 4H, 4 × piperidin-*H*), 1.37 – 1.25 ppm (m, 2H, 2 × piperidin-*H*)

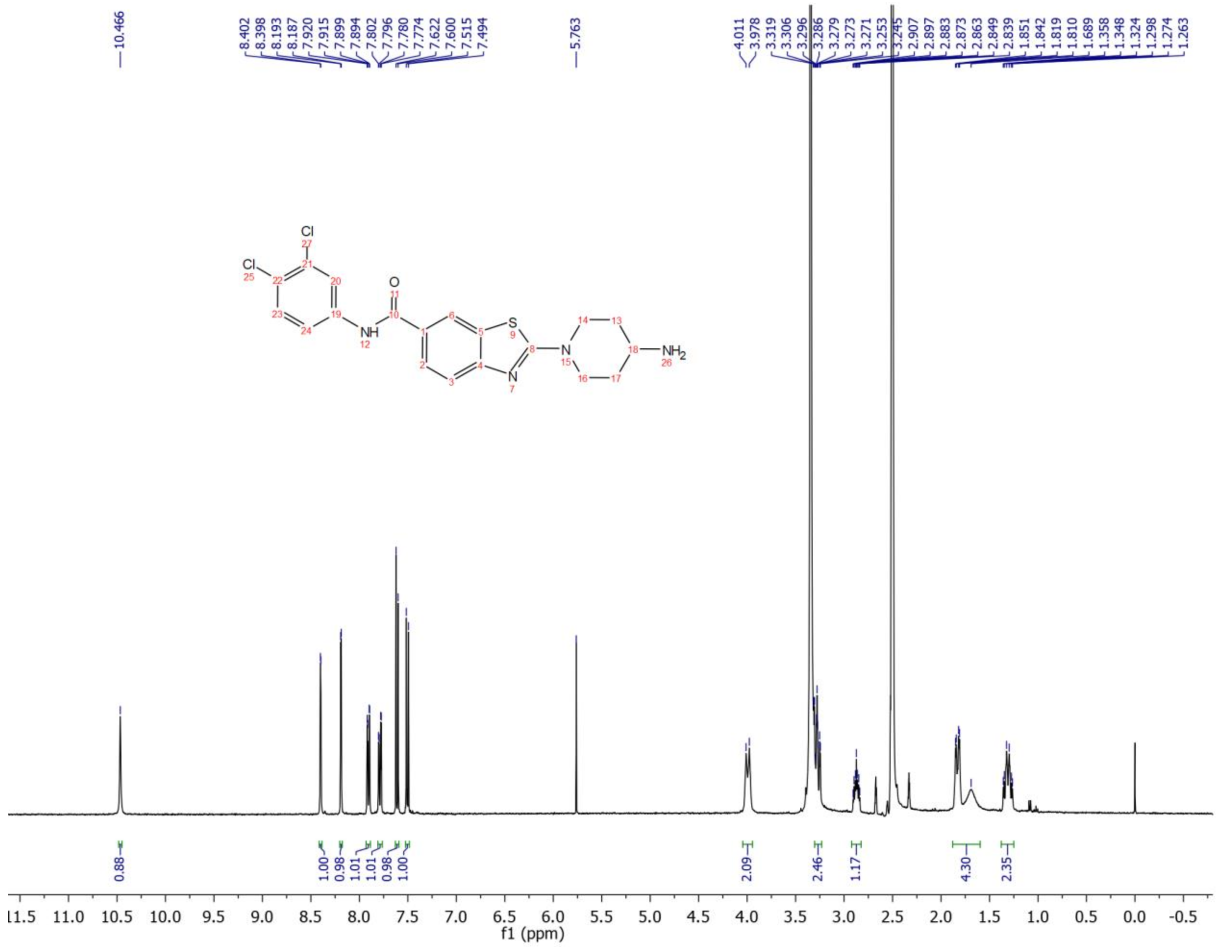

Figure S99. ^{1}H NMR spectrum of compound **14**.

^{13}C (101 MHz, [D_6]DMSO, 25°C, TMS): δ = 170.2, 165.8, 156.3, 140.2, 131.2, 130.9 (2C), 126.9, 126.8, 125.2, 121.8 (2C), 120.7, 117.9, 48.0 (2C), 47.6 (2C), 34.8 ppm

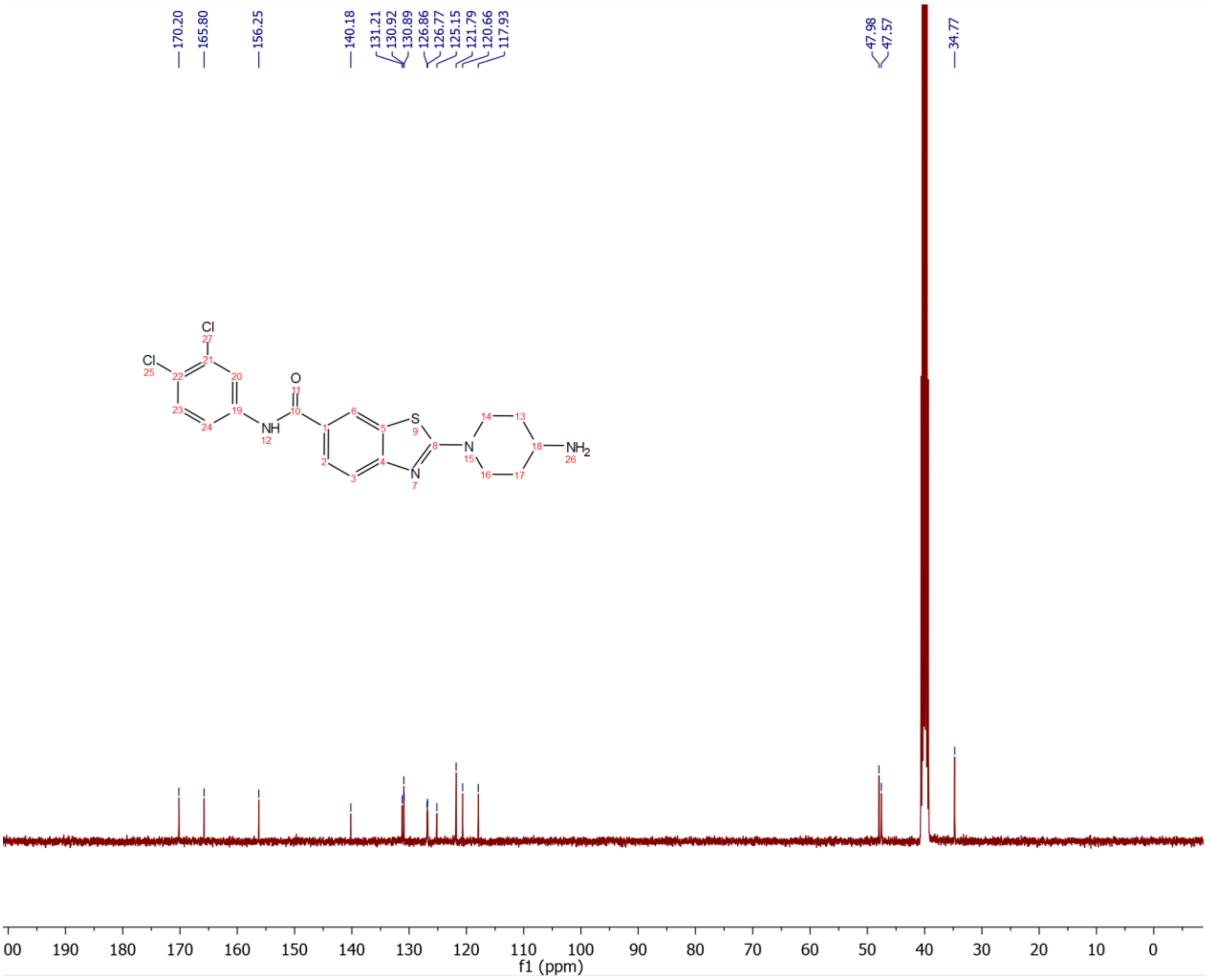

Figure S100. ^{13}C NMR spectrum of compound **14**.

HPLC: t_r = 6.31 min (95.1 % at 254 nm)

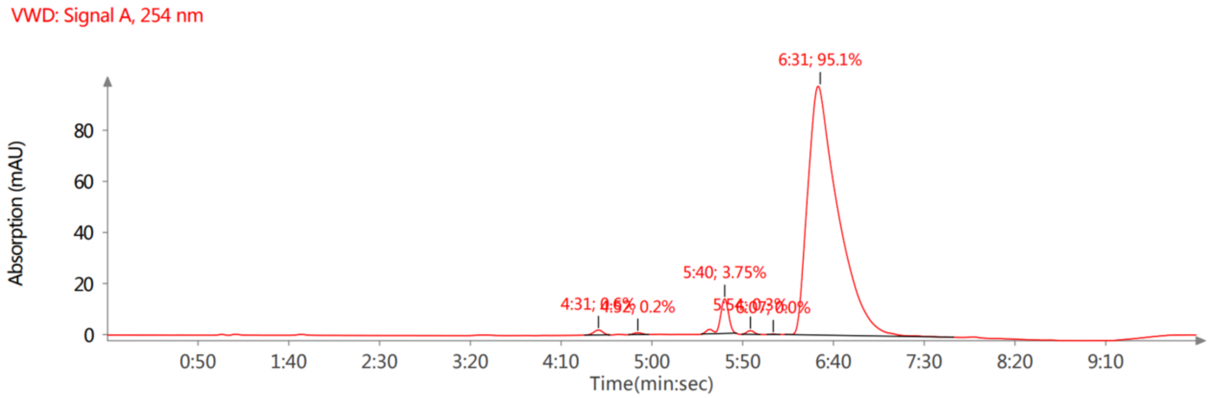

Figure S101. HPLC chromatogram of compound **14**.
